# The synthesis of brominated-boron-doped PAHs by alkyne 1,1-bromoboration: mechanistic and functionalisation studies†‡

**DOI:** 10.1039/c9sc05404a

**Published:** 2020-02-27

**Authors:** K. Yuan, R. J. Kahan, C. Si, A. Williams, S. Kirschner, M. Uzelac, E. Zysman-Colman, M. J. Ingleson

**Affiliations:** EaStCHEM School of Chemistry, University of Edinburgh Edinburgh EH9 3FJ UK michael.ingleson@ed.ac.uk; School of Chemistry, University of Manchester Manchester M13 9PL UK; Organic Semiconductor Centre, EaStCHEM School of Chemistry, University of St Andrews St Andrews KY16 9ST UK

## Abstract

The synthesis of a range of brominated-B_*n*_-containing (*n* = 1, 2) polycyclic aromatic hydrocarbons (PAHs) is achieved simply by reacting BBr_3_ with appropriately substituted alkynes *via* a bromoboration/electrophilic C–H borylation sequence. The brominated-B_*n*_-PAHs were isolated as either the borinic acids or B-mesityl-protected derivatives, with the latter having extremely deep LUMOs for the B_2_-doped PAHs (with one example having a reduction potential of *E*_1/2_ = −0.96 V *versus* Fc^+^/Fc, Fc = ferrocene). Mechanistic studies revealed the reaction sequence proceeds by initial alkyne 1,1-bromoboration. 1,1-Bromoboration also was applied to access a number of unprecedented 1-bromo-2,2-diaryl substituted vinylboronate esters directly from internal alkynes. Bromoboration/C–H borylation installs useful C–Br units onto the B_*n*_-PAHs, which were utilised in Negishi coupling reactions, including for the installation of two triarylamine donor (D) groups onto a B_2_-PAH. The resultant D–A–D molecule has a low optical gap with an absorption onset at 750 nm and emission centered at 810 nm in the solid state.

## Abstract 2

The synthesis of a range of brominated-B_*n*_-containing (*n* = 1, 2) polycyclic aromatic hydrocarbons (PAHs) is achieved simply by reacting BBr_3_ with appropriately substituted alkynes *via* a bromoboration/electrophilic C–H borylation sequence.

## Graphical abstract

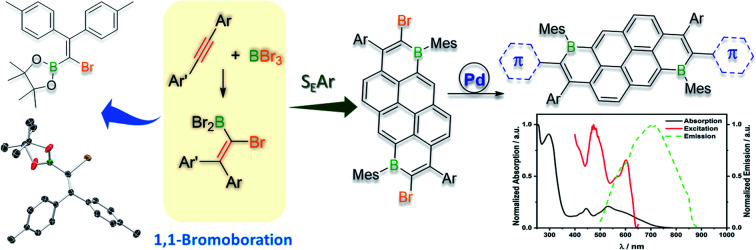

## Introduction

**Fig. 1:**
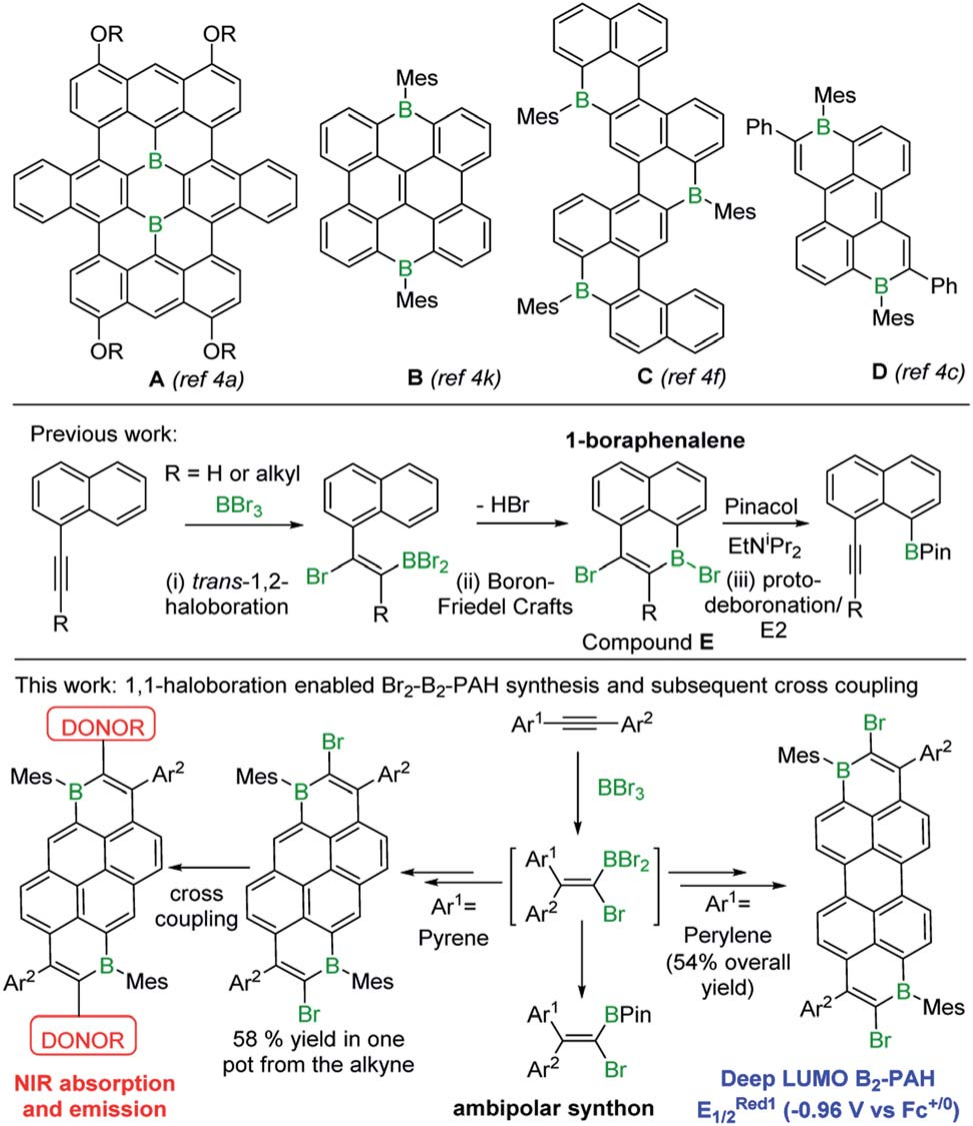
Top, select examples of some of the largest/deepest LUMO B_*n*_-PAHs (*n* > 1) containing three coordinate C_3_B units. Middle, previous work forming boraphenalenes by bromoboration/S_E_Ar. Bottom, this work accessing and utilising large, low LUMO energy dibrominated B_2_-PAHs.

The incorporation of main group elements into conjugated organic scaffolds is a powerful strategy to tune electronic properties.^1^ Recently, this approach has been extended to the introduction of three-coordinate boron atoms into polycyclic aromatic hydrocarbons (PAHs).^1,2^ In these “B-doped”-PAHs interaction between the empty p orbital on boron and the extended π system often leads to molecules with low energy LUMOs.^2^ B-doped PAHs now are being explored for a range of applications including organic light-emitting diodes, thin film transistors, solar cells, lithium batteries and electrocatalysis.^2^ However, two factors have limited the wider uptake of purely B-doped PAHs (*i.e.* PAHs without co-doping elements *e.g.* N): (i) the limited simple synthetic routes to form B_*n*-_PAHs, particularly for *n* > 1, which generally have the deeper LUMOs;^2^ (ii) the challenge associated with incorporating B_*n*_-doped-PAHs into more complex materials, *e.g.* by coupling reactions to access donor–acceptor (D–A) structures.^3^ Some notable progress has been made addressing (i), with several routes to larger B_*n*_-PAHs, (*n* ≥ 2, see **A–D**, Fig. 1) including examples with low LUMO energies, recently reported.^4,5^ However, the incorporation of B_*n*_-PAHs into more complex materials currently requires the post synthetic introduction of additional functionality onto the B_*n*_-PAH to enable subsequent steps (*e.g.* cross coupling).^6^ This has associated challenges (*e.g.* selectivity in halogenation) along with step-economy issues. Therefore, a simple route to form a range of B_*n*_-doped PAHs with low LUMO energies that concomitantly installs a second useful functional group, such as halide, would be highly attractive and facilitate access to more complex materials with desirable properties.

In our previous work, a one-pot synthesis of 1-boraphenalenes (**E**) *via* sequential bromoboration/intramolecular boron-Friedel–Crafts reaction of 1-alkynylnaphthalenes (Fig. 1, middle) was described.^7^ We envisaged that this methodology could be extended by: (i) using larger (than naphthalene) π-scaffolds to obtain B_1_ and B_2_ doped PAHs possessing deeper LUMOs; (ii) utilising the bromo substituent to access more complex materials with desirable properties *via* coupling reactions. Herein these two objectives are realised enabling access to the lowest LUMO energy ambient stable B_*n*_-doped PAH reported to date, to the best of our knowledge, and a low-optical gap D–A–D material where the acceptor unit is a B_2_-PAH. In addition, mechanistic studies on the bromoboration/S_E_Ar process revealed it proceeds by alkyne 1,1-bromoboration, a process not previously observed. Therefore 1,1-bromoboration also was applied to simple diarylalkynes demonstrating its utility for forming 1-bromo-2,2-diaryl substituted vinylboronate esters that are otherwise challenging to access.

## Results and discussion

### Synthesis of boron-doped PAHs and 1,1-bromoboration studies

**Scheme 1:**
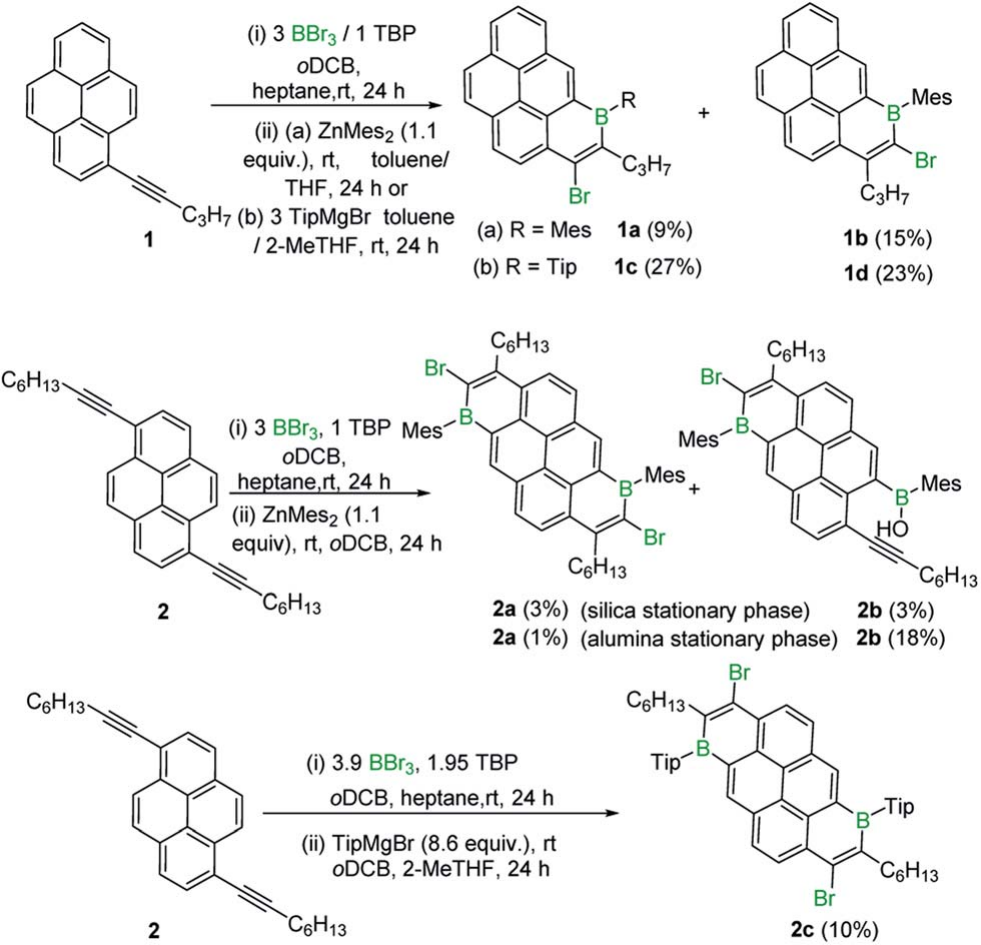
Isolated products from the reaction of alkyl-alkynyl-pyrenes with BBr_3_.

Initially, 1-(pent-1-yn-1-yl)pyrene, **1**, was combined with BBr_3_ in the presence of one equivalent of 2,4,6-tri-*tert*-butylpyridine (TBP), targeting an analogous *trans*-haloboration/S_E_Ar process that previously afforded 1-boraphenalenes.^7^ Post protection at boron by reaction with dimesitylzinc, purification afforded two isomeric compounds, **1a** and **1b** (Scheme 1) in low yield, partly due to low stability of these compounds to the column chromatography. To provide enhanced stability, the bulkier 2,4,6-triisopropylphenyl (Tip) protecting group was used instead of mesityl (Mes). Using similar reaction conditions, compounds **1c** and **1d** were isolated in higher yields relative to **1a** and **1b**.

B_2_-doped PAHs were next targeted through a two-fold bromoboration/S_E_Ar sequence starting from 1,6-di(pent-1-yn-1-yl)pyrene, **2**. However, post borylation/protection at boron, compound **2a** was the only fully fused B_2_-PAH isolable *via* column chromatography in our hands and it was obtained in a very low yield alongside **2b**. Compound **2b** presumably derives from protodeboronation of a vinyl C–B unit (analogous to that in **1a**) followed by an E2 elimination reaction as previously observed for the 1-boraphenalenes (see Fig. 1, middle right).^7^ Tip installation conditions also were applied to the product mixture derived from BBr_3_/**2**, however this led to the formation of compound **2c** in 10% yield as the only isolable product in our hands. The structures of **1a**, **2a** and **2c** were confirmed by single crystal X-ray diffraction analysis (see subsequent discussion). It should be noted that products derived from both 1,2 and 1,1-haloboration were observed (*e.g.* the B–Br analogues of **2a**/**2c**) and this may explain the complex mixtures observed starting from **2** with lower symmetry (than **2a**/**2c**) borylated products also observed spectroscopically (presumably containing one 1,1- and one 1,2-bromoborated boracycle in the same molecule).

**Scheme 2:**
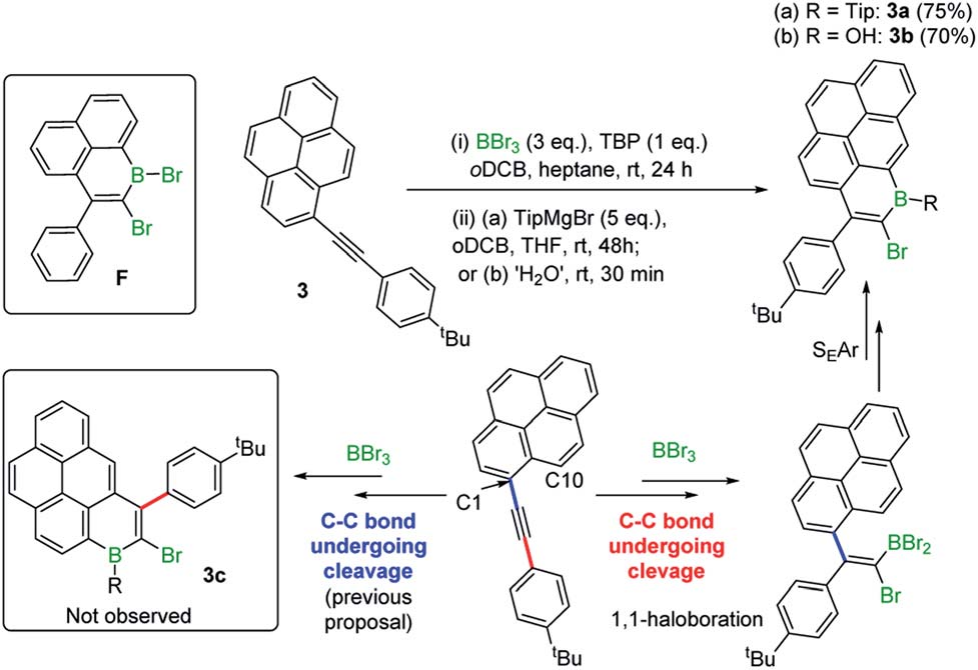
Formation of **3a** and **3b** and mechanistic implications.

In previous work 1-(arylethynyl)naphthalenes reacted with BBr_3_ to give different products to that starting from 1-(alkyl-ethynyl)naphthalenes, with the former affording products from a 1,1-bromoboration/S_E_Ar cyclisation process (*e.g.***F**, inset Scheme 2, is formed instead of an analogue of **E**, Fig. 1 middle),^7^ with no 1,2-bromoboration derived products observed. Therefore, aryl-alkynyl-pyrene derivatives (*e.g.***3**) were utilised in an attempt to avoid forming mixtures derived from competing 1,1 and 1,2-bromoboration. Starting from **3**, the desired Tip protected product **3a** was isolated in 75% yield with negligible 1,2-bromoboration products observed. In addition, the borinic acid **3b** was accessible in good yield using simple aqueous workup conditions. **3b** proved sufficiently stable for column chromatography and even towards 1 M HCl (aq.) for at least 0.5 h. Notably, all the aryl-substituted derivatives studied herein proved more resistant to protodeboronation than the alkyl congeners, with protection by mesityl also providing sufficient stabilisation for these derivatives. The NMR data for **3a**/**3b** are comparable to that previously reported for the B–OH and B-aryl 1-boraphenalenes.^7^

**Scheme 3:**
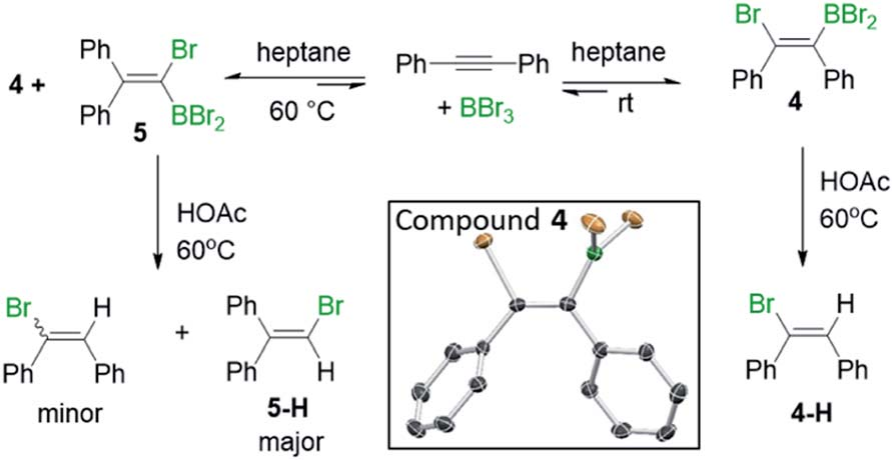
The observation of 1,1- and 1,2-bromoboration under different conditions. Inset, the solid state structure of **4**, hydrogens omitted for clarity.

Earlier studies on the bromoboration of diphenylacetylene using BBr_3_ by Lappert and co-workers suggested that only the 1,2-*cis*-bromoboration product (**4**, Scheme 3 inset) forms.^8^ Furthermore, alkyne 1,1-haloboration using boron electrophiles was to the best of our knowledge unprecedented prior to this work, with 1,2-haloboration or 1,1-carboboration the general outcomes.^9^ Thus, in our previous report on the bromoboration of 1-(arylalkynyl)-naphthalenes,^7^ a mechanism was proposed for forming **F** that avoided 1,1-bromoboration. According to that previously proposed mechanism, compound **3c** (inset Scheme 2) should be obtained starting from **3***via* cleavage of the pyrene C1–C_alkyne_ bond. However, X-ray diffraction analysis of the haloboration products **3a** and **3b** confirmed that boron was attached to C10 of pyrene and that the C1–C_alkyne_ bond remains intact. These observations indicate the reaction most likely proceeds through 1,1-bromoboration followed by electrophilic C–H borylation (Scheme 2, bottom right). Therefore, the bromoboration of other diarylalkynes, including diphenylacetylene, using BBr_3_ was revisited to probe the apparent discrepancy with earlier work.

On combining diphenylacetylene with BBr_3_ in heptane, crystals precipitated over the course of 5 minutes X-ray diffraction analysis on these revealed them to be the 1,2-*cis*-bromoboration product, **4** (inset Scheme 3). NMR studies under the same conditions revealed that only one product is observed in solution, assigned as compound **4**. Protodeboronation of this reaction mixture afforded 1-bromo-1,2-diphenylethene, **4-H** with no 1,1-bromoboration isomer (**5-H**, Scheme 3) observed. While these observations are consistent with previous work,^8^ upon heating the reaction mixture in heptane at 60 °C an additional product, assigned as **5**, was observed (by ^13^C{^1^H} and ^11^B NMR spectroscopy). Protodeboronation of this reaction mixture afforded 1-bromo-2,2-diphenylethene, **5-H**, derived from 1,1-haloboration as the major product along with minor amounts of 1-bromo-1,2-diphenylethene (**4-H**). Notably, when bromoboration was performed in DCM, both 1,2- and 1,1-bromoboration products were observed after short reaction times at 20 °C (*ca.* 10 minutes by NMR spectroscopy and by analysis of products post protodeboronation). These observations suggest that 1,1-bromoboration proceeds through a polar transition state(s), and potentially a vinyl cation type intermediate(s), more stabilised by the polar solvent DCM. Identical product distributions also were observed using 3 equiv. of BBr_3_ in place of 1.5 equiv. of BBr_3_.

**Scheme 4:**
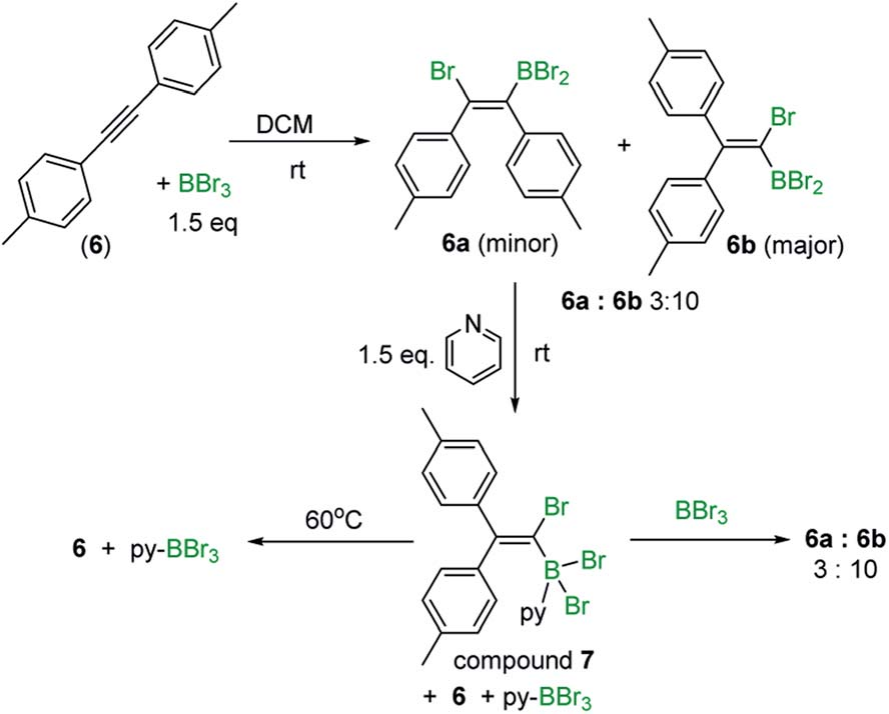
Bromoboration in DCM, and reactivity of products towards pyridine.

Similar outcomes were observed with di-*p*-tolylacetylene (**6**) and 1,2-bis(4-fluorophenyl)ethyne with the 1,1-haloboration product being the major species observed in DCM in both cases. Exact ratios of the BBr_2_ 1,1 : 1,2-haloboration (both *cis* and *trans* isomers) products in DCM are challenging to determine *in situ* by NMR spectroscopy due to uncertainty in isomer assignment. Furthermore, ratios of BPin/vinyl C–H products post work-up and isolation are not indicative of *in situ* ratios due to competing pinacol protection/protodeboronation/retrohaloboration (see ESI‡ Section 4 for further discussion). For di-*p*-tolylacetylene a mixture of bromoboration products (*ca.* 3 : 10) was formed in DCM 10 minutes after the addition of BBr_3_ at room temperature. To determine the identity of the major isomer the fact that alkyne 1,2-bromoboration products (*e.g.***4**) are converted rapidly back into the starting alkyne on adding pyridine,^8^ (concomitantly forming pyridine–BBr_3_) was exploited. Upon addition of pyridine to the 3 : 10 bromoboration reaction mixture, the minor bromoboration product, assigned as **6a** (Scheme 4), was converted into the starting alkyne and pyridine–BBr_3_, while the major bromoboration product, assigned as **6b**, formed an adduct, **7**, with pyridine at room temperature. Upon heating at 60 °C, compound **7** also converted into di-*p*-tolylacetylene and pyridine–BBr_3_. Addition of excess BBr_3_ to the mixture containing **7** to sequester all pyridine as pyridine–BBr_3_ regenerated the mixture of 1,2- and 1,1-haloboration products in an identical 3 : 10 ratio to that originally observed. Combined these results indicate that 1,1-haloboration dominates in DCM, that this reaction reaches its equilibrium position rapidly in DCM, and that both 1,2- and 1,1-bromoboration are reversible. DFT calculations (at the M06-2X/6-311G(d,p) PCM (DCM) level) revealed the 1,1- and 1,2-bromoboration products (*e.g.***4** and **5**) were effectively isoenergetic (all pairs of isomers had Δ*E* < 1.3 and Δ*G* < 0.8 kcal mol^−1^) consistent with the observation of mixtures on reaching equilibrium. Finally, 1,1-bromoboration is not limited to diarylalkynes, with 1-phenyl-1-propyne also leading to 1,1-bromoboration products.

**Scheme 5:**
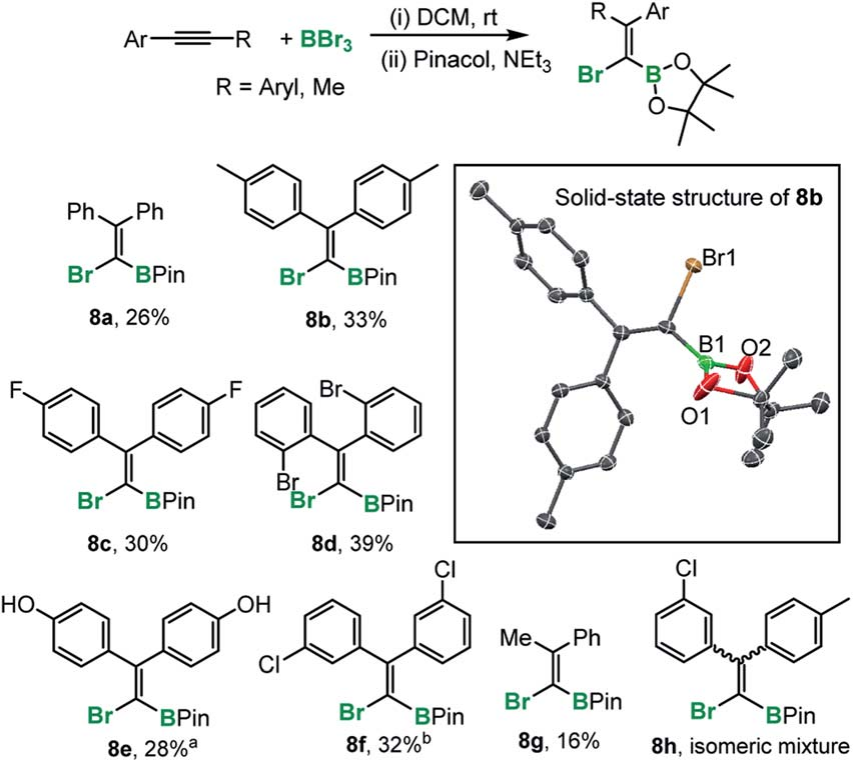
1,1-Haloboration and subsequent pinacol protection. Inset right, solid state structure of **8b**, hydrogens omitted for clarity, ellipsoids at 50% probability. a = from the *p*-MeO derivative with ether cleavage occurring concomitantly to haloboration. b = pinacol protected without Et_3_N.

The preparation of fully-substituted 1-bromo-1-alkenyl boronate esters currently is challenging (the major route to these types of compounds proceeds *via* hydroboration of alkynyl-halides thus is limited to forming tri-substituted alkenes).^10^ Indeed diaryl-derivatives (such as **8a–f**Scheme 5), have not been previously reported to the best of our knowledge. While 1,1-bromoboration represents a simple approach to these compounds to be useful protection at boron without (or with minimal) competing formation of the starting alkyne by retro-bromoboration is required. In the presence of excess triethylamine, the 1,1-bromoboration products (*e.g.***5**) were converted into the pinacol boronate esters, **8a–g** demonstrating that *ortho*, *meta* and *para* substituted aryl-alkynes are amenable to this procedure. The yields are moderate at best due to the presence of the 1,2-isomer in the reaction mixture and the propensity of the Br-vinyl-BBr_2_ isomers (*e.g.***4** and **5**) to undergo retrohaloboration in competition to pinacol protection (see ESI‡ for more details). Indeed, under these conditions all the 1,2-bromoboration products (and the mass balance of the 1,1-haloboration products) were converted into the starting alkyne and Et_3_N–BBr_3_. Attempts at pinacol installation in the absence of base led to competitive protodeboronation (presumably due to the HBr by-product from pinacol installation onto the BBr_2_ moiety) and lower yields of **8x** in most cases. The pinacol boronate esters were fully characterised and a number confirmed by X-ray diffraction analysis (*e.g.* inset Scheme 5). It should be noted that substrates containing CF_3_ were not amenable to this process due to B–F/C–Br bond formation on addition of BBr_3_. Furthermore, ether cleavage occurs concomitantly to haloboration, with substrate **8f** isolated from the 4-methoxyaryl precursor. In addition, attempts to bromoborate 2,2′-bis(2-thienyl)ethyne with BBr_3_ were successful to some extent, but protection with pinacol under a range of conditions was unsuccessful. This instead led to protodeboronation under a range of conditions. Finally an electronically biased diaryl alkyne was subjected to haloboration in DCM, however pinacol protection revealed that multiple haloborated isomers, *e.g.***8h**, had formed that could not be separated in our hands.

**Scheme 6:**
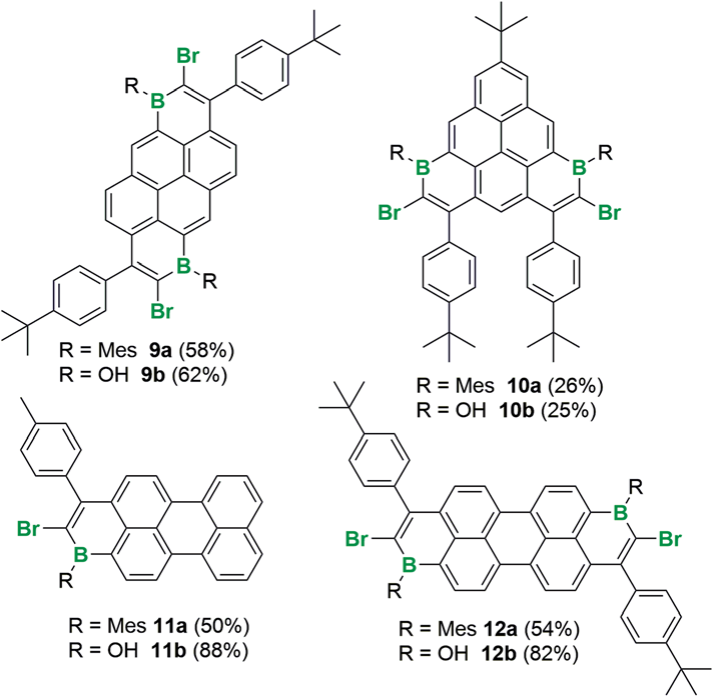
The formation of extended B_*n*_-PAHs by bromoboration/S_E_Ar.

With an understanding of the bromoboration process in hand, the extension of 1,1-haloboration/S_E_Ar to other substrates was explored. Using 1,6-bis((4-(*tert*-butyl)phenyl)ethynyl)pyrene, **9** (an analogue of **2**) and BBr_3_ this sequential transformation worked effectively and post mesityl installation afforded **9a** in 58% yield in a one-pot reaction starting from **9**. Compound **9a** proved bench stable (as **9–12a/b** all are) and was purified *via* column chromatography. The borinic acid **9b** could also be prepared from **9** in 62% yield *via* a simple hydrolytic work-up. Starting from 7-(*tert*-butyl)-1,3-bis((4-(*tert*-butyl)phenyl)-ethynyl)pyrene, **10**, both the Mes-protected product **10a** and the borinic acid **10b** also were accessible, albeit isolated in lower yields. 1,1-Haloboration/S_E_Ar also was applicable to perylene substrates. For 3-(*p*-tolylethynyl)perylene, **11**, the Tip-protected product **11a** (50%) and the borinic acid **11b** (88%) were isolated post column chromatography. The bromoboration/S_E_Ar cyclisation reaction also worked with 3,9-bis((4-(*tert*-butyl)phenyl)ethynyl)perylene, **12**, to yield the Mes-protected B_2_-PAH **12a** (54% yield) and the borinic acid **12b** (82% yield) depending on the work-up protocol (Scheme 6).

### Solid-state structures

**Fig. 2:**
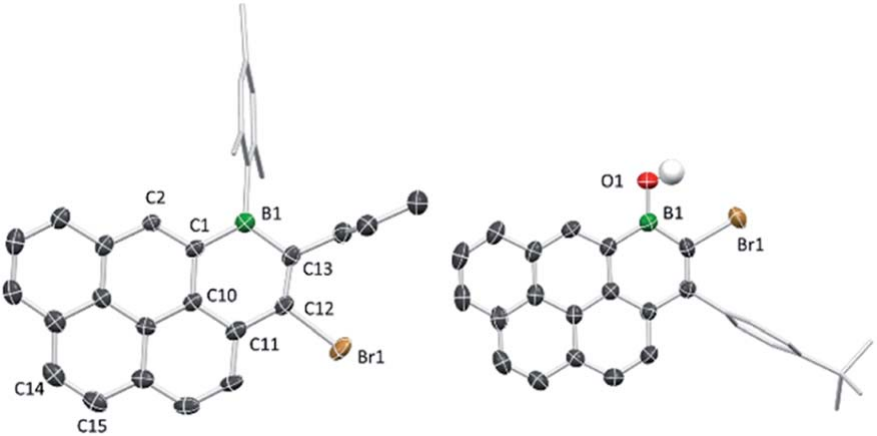
Solid-state structures of **1a** (left) and **3b** (right), most hydrogens omitted for clarity. Ellipsoids at the 50% probability level.

The solid-state structures of B_1_-PAHs **1a**, **1c** and **3b** revealed effectively planar six-membered boracycles with boron atoms adopting a trigonal planar geometry (*Σ*(C–B–C) ≈ 360°). Notably, the C12–C13 bond length (1.355(5) Å in **1a**) is shorter than the C11–C12, C1–C10 and C10–C11 bond lengths (1.462(5), 1.448(5) and 1.414(4) Å, respectively in **1a**) as is the case in **1c** and **3b**. The bond length alternation indicates the lack of significant π delocalisation within the boracycle as was observed with the 1-boraphenalenes.^7^ The fusion of a boracycle onto the PAH core does have some impact, for example on the C–C bond in the K-region of pyrene. For **1a** the bond length of C1–C2 (1.367(4) Å) is longer than that of C14–C15 (1.337(5) Å). The elongation of C1–C2 *vs.* C14–C15 may indicate some delocalisation of this π-electron density into the boron centre.^11*a*^ For compound **3b**, the B–O bond length (1.369(4) Å) is close to the reported values for other related borinic acids, indicating some multiple bond character between boron and oxygen. The Mes/Tip and *tert*-butylphenyl groups are all almost orthogonal to the boracycle, which precludes close face to face π stacking involving the boracycles in the intermolecular structure (Fig. 2).

**Fig. 3:**
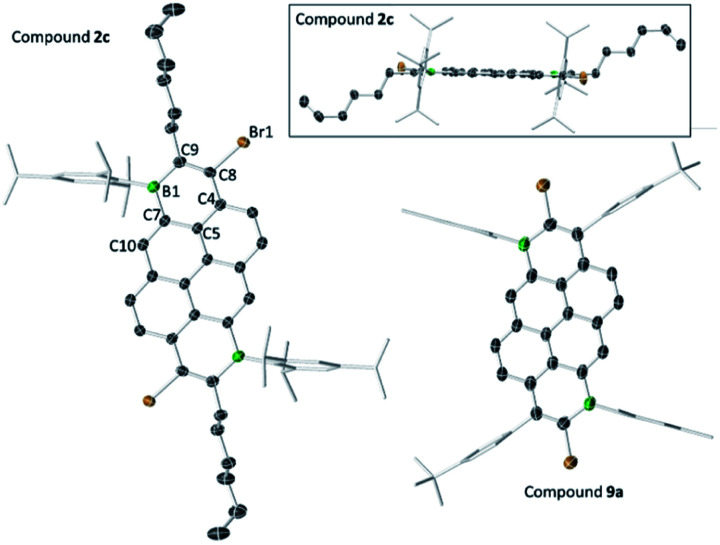
Solid state structures of **2c** (two views) and **9a**, with hydrogens omitted for clarity. Ellipsoids are at the 50% probability level.

**Fig. 4:**
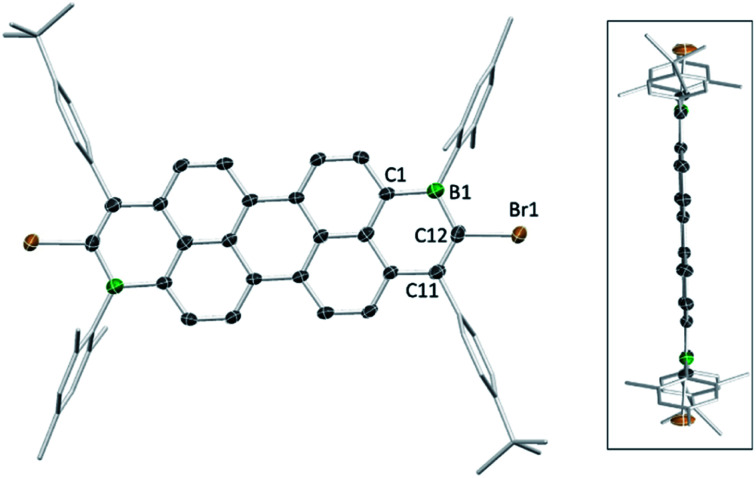
Solid state structure of **12a** (two views), with hydrogens omitted for clarity. Ellipsoids are at the 50% probability level.

The solid-state structures of the B_2_-doped PAHs **2a**, **2c**, **9a**, **10b** and **12a** also were determined (Fig. 3, 4 and ESI‡). All five compounds show planar π-conjugated cores with the peripheral substituents effectively orthogonal to the PAH core. As a result, no face to face π-stacking is observed in the extended structures for these compounds (excluding **10b**, see ESI‡). For example, the face to face intermolecular separation of the cores of two parallel molecules is found to be 6.67 Å for **2c**. The boracycle metrics are similar to the mono-borylated compounds with C–C bond length alternation within the boracycles also observed (for example a C11–C12 vinylic distance in **12a** of 1.359(7) Å and long endocyclic B–C bonds 1.534(6) and 1.543(6) Å indicate minimal π delocalisation consistent with previous NICS calculations).^7^ Notably, for the diborylated pyrene compounds, the length of the C–C bonds in the pyrene K-region is longer than that in the mono-borylated compound (*e.g.* the bond length of C7–C10 is 1.379(3) Å for compound **2c**). The elongation of C7–C10 bond length may indicate a greater delocalisation of these π-electrons into the boron centres for the di-borylated compounds relative to the mono-borylated compounds.

### Cyclic voltammetry

The first reduction potentials ranged from −1.91 V (**3b**) to −0.96 V (**12a**, Table 1). Within the scan window, the B_1_-doped PAHs showed one reversible reduction wave except for compound **11a**, for which two reduction waves were observed. All B_2_-PAHs showed two reversible reduction waves; however, no oxidation waves were observed within the solvent window for these compounds. The hydroxyl group on boron reduced the electron-accepting abilities of the boron-doped PAHs compared with the Tip/Mes group as previously observed.^4*c*^ Also as noted by Würthner and co-workers,^4*c*^ the boron doping pattern also significantly influences the redox properties with the first reduction potential of **9a** more positive than that of **10a** by 0.15 V (for **9b***vs.***10b** Δ*E*^red1^_1/2_ = 0.32 V). Notably, the reduction potential of **9a** is more positive than bis-(BMes_2_)-pyrenes (4,9 isomer *E*^red1^_1/2_ = −2.31 V, 1,6 isomer *E*^red1^_1/2_ = −1.81 V)^11^ and the products from the electrophilic borylation of 1,6-(2-pyridyl)_2_-pyrene (when the boron moiety = BPh_2_*E*^red1^_1/2_ = −2.08 V)^14^ this is ascribed to greater π delocalization in **9a** as a result of planarization and three-coordinate B centres. Finally, **9a** has a more positive reduction potential than its all carbon analogue (for which *E*^red1^_1/2_ = −2.11 V),^15^ further confirming boron-doping as an effective strategy to obtain molecules with deep LUMOs due to their electron-deficient nature (B_2_-PAHs are isoelectronic to dicationic carbon analogues).

**Table 1:** Optoelectronic properties of B_*n*_-PAH compounds and a **PDI** for comparison

	*E* ^red1^_1/2_ a (V)	*E* ^red2^_1/2_ a (V)	LUMO^exp^^b^ (eV)	*λ*_abs_ c [*ε*/10^−3^ M^−1^ cm^−1^] (nm)	*λ*_PL_ c (nm)	*Φ*_PL_ d (%)	*E*_g_ e (eV)	HOMO^f^ (eV)	LUMO^f^ (eV)
**3a**	−1.66	—	−3.49	375[18.04], 395[15.12], 417[13.16], 491[17.98]	553, 592	9.2	2.26	−7.04	−2.29
**3b**	−1.91	—	−3.24	359[3.81], 383[3.62], 404[5.34], 449[7.86]	500, 532	8.7	2.51	−6.97	−2.05
**9a**	−1.03	−1.49	−4.12	345[3.23], 362[4.74], 396[2.64], 536[24.13], 567[18.00]	599, 648	2.0	2.07	−7.26	−2.98
**9b**	−1.42	−1.73	−3.73	340[2.41], 355[3.69], 390[1.56], 501[32.13],528[25.05]	557, 598	6.8	2.23	−6.97	−2.60
**10a**	−1.30	−2.11	−3.85	332[20.75], 386[21.72], 406[35.98], 435[15.45], 508[3.64], 545[4.88], 588[3.52]	614, 668, 729	5.9	2.00	−7.19	−2.67
**10b**	−1.61	−2.25	−3.54	380[34.50], 417[3.93], 443[6.04], 476[8.37], 507[11.57], 543[8.22]	560, 605, 657	46.9	2.19	−7.00	−2.43
**11a**	−1.43	−2.06	−3.72	314[57.13], 360[8.01], 405[10.53], 431[15.93], 456[26.37], 500[16.68], 534[33.55], 573[43.71]	591, 628	0.6	2.08	−6.76	−2.50
**11b**	−1.57	—	−3.58	312[28.42], 340[5.37], 410[7.71], 433[14.85], 456[13.10], 487[29.85],521[31.05]	532, 570, 618	0.4	2.31	−6.68	−2.29
**12a**	−0.96	−1.28	−4.19	336[5.91], 392[5.49], 528[18.02], 568[44.48], 614[68.41]	634, 686	1.6	1.93	−7.06	−3.08
**12b**	−1.28	−1.54	−3.87	370[1.48], 494[4.90], 530[12.52], 571[19.61]	582, 630	3.8	2.10	−6.86	−2.74
**D** g	−1.07	−1.41	−4.08	611, 417	668	74		—	—
**PDI** h	−1.02		−4.13	527	534	99		−7.21	−2.89

a Measurements carried out at 298 K in THF with 0.1 M [^*n*^Bu_4_N][PF_6_] as the supporting electrolyte. Electrochemical reduction potentials were calibrated with ferrocene as internal standard and referenced *vs.* Fc^+^/Fc.

b *E*_LUMO_ = −*E*^red^_1/2_ − 5.15 eV.^12^

c Optical measurements carried out at 298 K in toluene at 1 × 10^−5^ M.

d Under aerated conditions using [Ru(bpy)_3_](PF_6_)_2_ in CH_3_CN as the reference (*Φ*_PL_: 1.8%).

e *E*_g_ were determined at the energy corresponding to 10% of the lowest energy absorption band.

f From calculations at the M06-2X/6-311G(d,p) level.

g From ref. 4c.

h **PDI** refers to the perylene diimide with N-Dip substituents from ref. 13 (Dip = 2,6-diisopropylphenyl).

Comparing pyrene and perylene core structures the reduction potentials of the borylated perylene compounds are positively shifted relative to the pyrene substrates. For example, the monoborylated compound **11a** exhibited the first reduction wave at −1.43 V more positive than **3a** by 0.23 V. Indeed among all the compounds investigated, **12a** shows the least negative first reduction potential with *E*^red1^_1/2_ = −0.96 V, which is also much more positive than the related all carbon peropyrenes.^16^ To the best of our knowledge, compound **12a** has the least negative reduction potential of all ambient stable three coordinate at boron B-doped PAHs reported to date, with compound D being the ambient stable B_*n*_-PAH with the next least negative reduction potential (Table 1).^4*c*^

### Photophysical properties

By varying the polycyclic core and the boron substituent, a series of B_*n*_-doped PAHs (*n* = 1, 2) are obtained forming solutions with colours ranging from yellow to dark blue. UV-vis absorption and fluorescence spectra were recorded and the optical energy gap estimated (Table 1). All compounds showed well resolved absorption/emission profiles with small Stokes shifts. Consistent with the observed solution colours, the obtained spectra showed the lowest energy absorption band spanning 449 nm (**3b**, light yellow solution) to 614 nm (**12a**, dark blue solution). The doubly borylated compound **9a** showed *λ*_max_ at 567 nm, which is red-shifted by 82 nm compared with the mono-borylated compound **3a** (*λ*_max_ = 491 nm). The same trend was also observed for **11a**/**12a**, due to the extended conjugation and double boracycle annulation leading to narrower HOMO–LUMO gaps. In addition, all borinic acids exhibited hypsochromic shifts of their lowest absorption band by *ca.* 40 nm compared with the Tip/Mes protected analogues. The photoluminescence quantum yield (*Φ*_PL_) of the brominated compounds studied herein were relatively low, which might be attributed to the heavy atom effect of bromine, as related heavy atom free analogues reported by Würthner and co-workers are notably more emissive (*Φ*_PL_s up to 95%).^4*c*^

### Computational studies

**Fig. 5:**
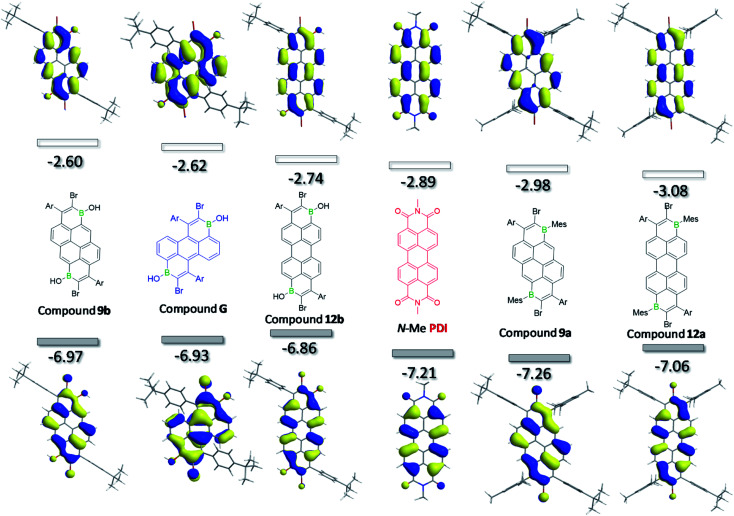
Calculated Frontier orbitals (shown at iso-values of 0.03) for several B_2_-PAHs and for comparison an N–Me substituted **PDI**. Ar = *p*-^*t*^Bu-phenyl.

To provide more insight into the trends observed in the experimental data, DFT calculations were performed at the M06-2X/6-311G(d,p) level with a polarisable continuum model (PCM) of DCM, calculations emulated the full compound (except for **2c**, see ESI‡). The trend in the LUMO energies from the computational results were consistent with the trends in the cyclic voltammetry data with compound **3b**, that has the most negative reduction potential, being calculated to have the highest LUMO energy (−2.05 eV) among the compounds studied, while compound **12a** with the least negative reduction potential was predicted to have the deepest LUMO level (−3.08 eV, see Fig. 5). It is noteworthy that the computed LUMO energy level of **12a** is lower than that of a perylene diimide (**PDI**, red, Fig. 5) calculated at the same level of theory. Indeed comparison of the CV data for **12a** and a **PDI** compound (containing all C–H and two N-DIP groups), confirms that **12a** has the deeper LUMO,^13^ while **9a** has very similar frontier orbital energies to this **PDI** (Table 1). This further indicates that both these diborylated pyrene and perylene compounds have potential as strong acceptor moieties in organic electronics.

As shown in Fig. 5 and the ESI‡, the LUMO and HOMO of all molecules are delocalised over the whole π-backbone and involve boron contributions to both. Interestingly, the frontier molecular orbital distributions of **12a** and **12b** are very similar to that of **PDI**. Furthermore, the frontier orbitals of all the B_2_-PAHs studied herein (and indeed the related compounds reported by Würthner *et al.*)^4*c*^ are related closely to the calculated HOMO and LUMO of the parent π core molecule (*e.g.* the HOMO and LUMO of pyrene and perylene)^17^ with some additional contributions from the appended B–C unit. For completeness, the anthracene-based compound **G** (blue, Fig. 5) was also calculated, which revealed that the HOMO and LUMO of this compound also was related in character to the HOMO and LUMO of anthracene. Note, attempts to synthesise **G** failed in our hands, possibly due to the additional peri C–H group inducing steric clash with the proximal aryl group (with some structural distortion observed in the calculated structure of **G**). It is notable that the trend in the relative HOMO/LUMO energy and optical gap of **9b**, **G** and **12b** mirrors that of the parent PAH core,^18^ with perylene (deepest LUMO/highest HOMO) < anthracene < pyrene (highest LUMO/deepest HOMO) for optical gap. This indicates that access to even lower LUMO energy B_2_-PAHs for this series maybe possible starting from lower energy LUMO core PAH structures.

### Functionalisation of boron doped PAHs by cross coupling

**Scheme 7:**
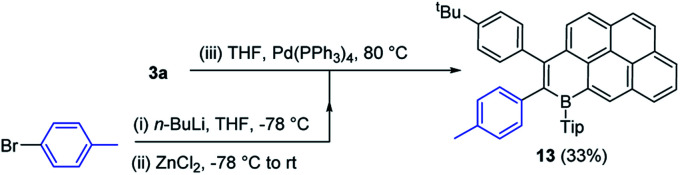
Negishi cross coupling to form compound **13**.

One attraction of the compounds reported herein is the potential utility of the bromide unit, derived from the concomitant installation of B/Br in the initial step of B_*n*_-PAH formation. While cross coupling reactions can be performed using the C–B units as the nucleophilic coupling partner,^19^ this removes the electronic effect of B-doping, thus utilising the C–Br unit in cross-coupling reactions offers a way to construct complex functional electronic materials that retain the C_3_B units that impart the deep LUMO character.^3^ Initially compound **3a** containing the sterically more demanding Tip unit, was explored and found to be amenable to Negishi cross coupling reaction conditions. Specifically, in the presence of Pd(PPh_3_)_4_, **3a** reacted with the *in situ* generated aryl-zinc reagent affording the desired product **13** (Scheme 7) in moderate 33% isolated yield. We attribute the modest yield to the highly sterically encumbered vinyl-Br position present in these B-PAHs, thus the B-Mes derivatives were explored.

**Fig. 6:**
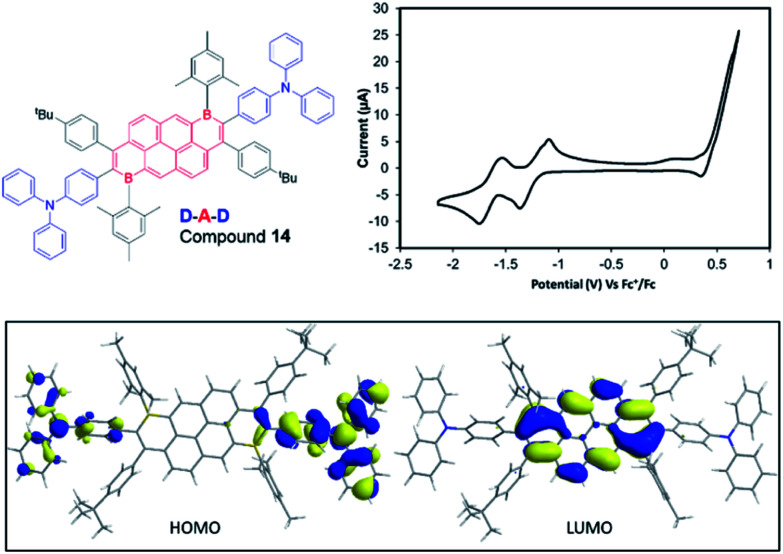
CV data, for compound **14** (1 mM solution in THF with 0.1 M [NBu_4_][PF_6_]. Inset bottom, HOMO and LUMO of compound **14** (iso-value 0.025) at the M06-2X/6-31G(d) level.

The same methodology could be applied to B-Mes derivatives to access a D–A–D molecule with a narrow HOMO–LUMO energy gap. Using the procedure established for **13**, **9a** was coupled with the organozinc compound derived *in situ* from 4-bromotriphenylamine to construct compound **14** (Fig. 6). The desired double cross coupled product, **14**, was isolated by column chromatography in 21% yield. Cyclic voltammetry measurements on compound **14** showed one oxidation wave within the solvent window (peak-current *E*_ox_ = 0.49 V *vs.* Fc/Fc^+^) and two reduction processes (*E*^red1^_1/2_ = −1.23 V, *E*^red2^_1/2_ = −1.62 V). The first reduction process is shifted cathodically by 0.2 V relative to **9a**, presumably due to the replacement of inductively electron-withdrawing bromine for the triphenylamine groups. DFT calculations (Fig. 6, bottom) were performed on compound **14** at a lower theory level due to its size (M06-2x/6-31G(d)), **9a** was calculated at the same level for direct comparison (which confirmed effectively identical HOMO/LUMO distributions for **9a** using the two basis sets). Consistent with a D–A molecule the HOMO/LUMO are spatially separated, with the HOMO being predominantly localised on the triaryl amine unit thus being 0.91 eV higher in energy than the HOMO of **9a**. The LUMO in **14** is localised on the B_2_-PAH acceptor unit and is closely related in character to the LUMO of **9a**. Notably, the calculated energy for the LUMO of **14** is 0.19 eV higher than that for **9a** in excellent agreement with the CV data; again this is attributed to the exchange of inductively withdrawing Br groups for triarylamine units.

**Fig. 7:**
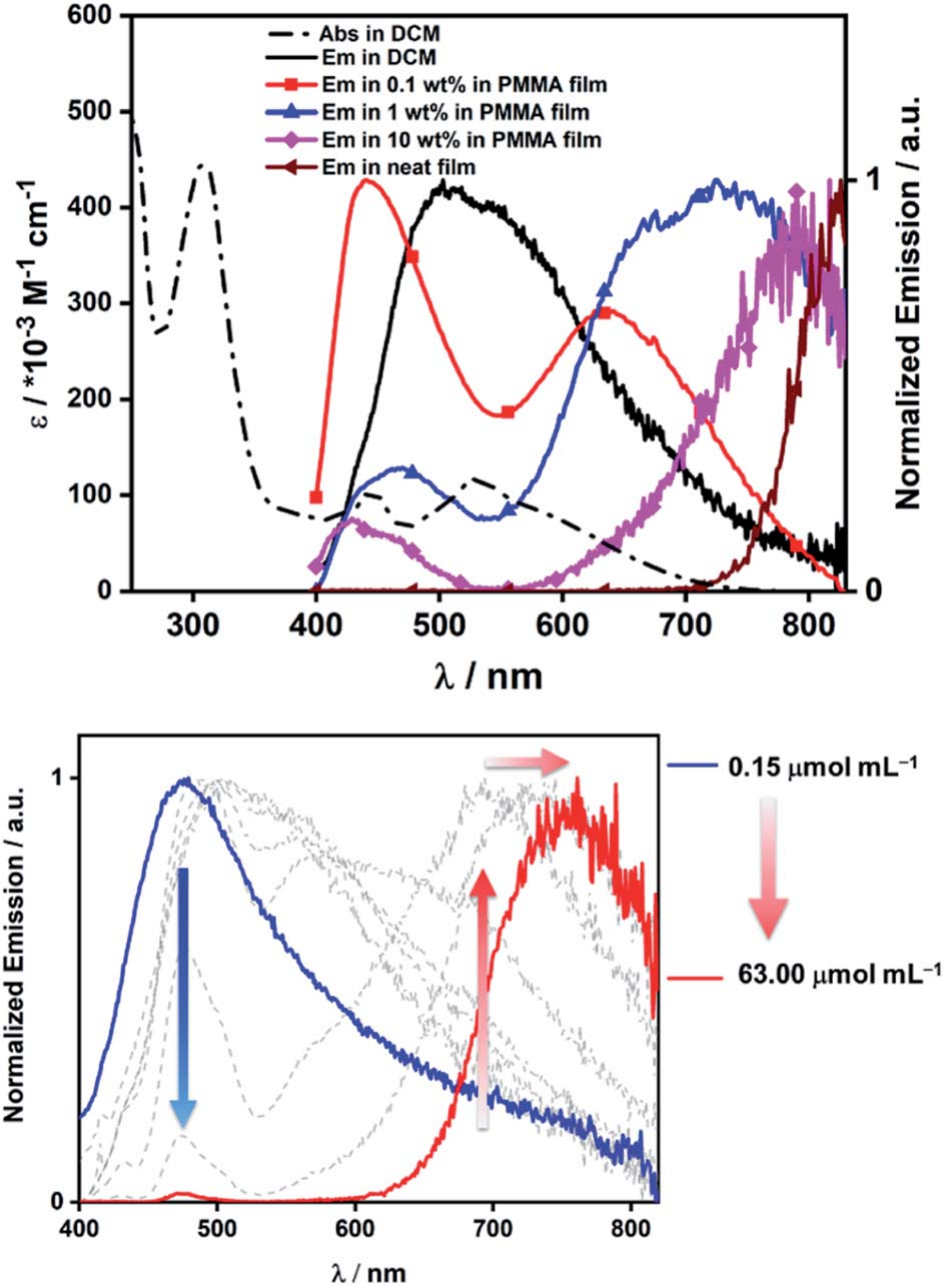
Top, photophysical data for compound **14** in DCM solution and in the solid state (neat film and dispersed in PMMA). Bottom, variation in the emission spectra on change of concentration of **14** in DCM solution. *λ*_exc_ = 370 nm.

The photophysical properties of **14** in solution, solid-state and dispersed in a PMMA film were investigated. Compound **14** displayed a broad absorption band stretching up to 750 nm in dichloromethane, with the onset corresponding to an optical gap of 1.65 eV (comparable to Δ*E*_redox_ determined by electrochemistry). Thus, the strong acceptor character of the B_2_-PAH unit combined with the triphenylamine donor units results in a small HOMO–LUMO energy gap. We employed time-dependent DFT (TDDFT) calculations using the Tamm-Dancoff approximation at the PBE0/6-31G(d,p) level of theory to assign the spectral features observed in the absorption spectrum of **14**. The lowest energy absorption features found within the tail of the band showing a maximum at 530 nm and a shoulder at 600 nm are assigned to be charge transfer (CT) states from the Ph_2_NPh group to the B_2_-PAH core (S_0_ → S_1_, S_0_ → S_2_). The principal distinguishable absorption features at 444 nm originate from mixed CT/LE (LE = locally excited) transitions, but this time the CT contribution implicates the mesityl groups as donors to the B_2_-PAH core (S_0_ → S_3_, S_0_ → S_4_, see ESI‡ for more details); the LE contribution is localized on the B_2_-PAH acceptor. Higher energy bands involve increasing contributions of LE transitions of the B_2_-PAH core. Recognizing the propensity of large acenes to aggregate in solution, even at very dilute concentrations we next investigated the effect of concentration on both the absorption and emission spectra. We note that both the *ε* values and the spectral features in the absorption spectra were independent of concentration, precluding aggregation of the molecule in its ground state in DCM at the concentrations studied (see ESI‡). In contrast, the emission in DCM was highly concentration dependent. At 0.15 μmol mL^−1^ the emission is broad and unstructured and centred at 479 nm. As the concentration is increased to 63 μmol mL^−1^, there is a decrease in intensity of this band coupled with a progressive and significant red-shift of the emission. At the highest concentrations studied, the emission narrows but remains unstructured (Fig. 7). We attribute this complex behaviour to the formation of mixtures of different excimers as the concentration increases, presumably with higher order aggregates forming at the higher concentrations.

The *Φ*_PL_ value in DCM for **14** at 0.52 μmol mL^−1^ is 4% and this value is not sensitive to oxygen. The excited state emission decays measured at both dilute (0.52 μmol mL^−1^) and concentrated (33 μmol mL^−1^) solutions are multi-exponential in nature but remain in the nanosecond regime, consistent with a fluorescence mechanism. In order to mitigate these non-radiative pathways, we next investigated the solid-state photophysical properties as thin films in PMMA at 0.1, 1 and 10 wt% doping concentrations, and as neat films. Generally, the same evolution in emission profiles is observed in the solid-state as was observed in DCM. As a 0.1 wt% PMMA doped film two unstructured emission bands are observed, one of higher relative intensity at high energy at *λ*_PL_ = 442 nm that we ascribe to the same monomer emission observed in dilute DCM, and a lower intensity and broader low energy band at *λ*_PL_ = 638 nm that we ascribe to excimeric emission. The *Φ*_PL_ of this film is 5%, which is essentially identical to that measured in DCM. This suggests that during the spin-coating process aggregation remains strong even at this low doping concentration. As a 1 wt% PMMA doped film, two unstructured emission bands remain, one at *λ*_PL_ = 472 nm and one at *λ*_PL_ = 726 nm; here, we note the large red-shift and significant enhancement in intensity of the low-energy band. The *Φ*_PL_ of this film is slightly lower at 3%. The 10 wt% doped PMMA film also shows two emission bands where the relative intensity of the high energy band at *λ*_PL_ = 434 nm is reduced further compared to the low energy band at *λ*_PL_ = 793 nm. The *Φ*_PL_ of this film is <1%. The neat film shows only a single very weak and red-shifted emission band at *λ*_PL_ = 824 nm with a *Φ*_PL_ < 1%. There is a systematic shortening of the *τ*_PL_ with increasing doping concentrations that mirrors the decrease in *Φ*_PL_ (see ESI‡). Irrespective of the complexity of the emission spectra the successful formation of the low optical gap material **14** demonstrates the utility of these dibrominated B_2_-PAHs in accessing complex organic materials.

## Conclusions

In conclusion, sequential bromoboration/intramolecular electrophilic C–H borylation enables formation of a range of brominated B_*n*_-doped PAHs in useful yields *via* an operationally simple, one-pot route from readily available precursors (alkynes and BBr_3_). Cyclic voltammetry and photophysical studies revealed that these molecules have very low LUMO energies thus are attractive acceptor units for use in organic electronic applications. In particular, compound **12a** has the least negative reduction potential among all reported ambient stable B-doped PAHs to the best of our knowledge. The C–Br units in the B_*n*_-PAHs can be utilised directly in Negishi cross-coupling reactions, which enabled formation of a donor–acceptor–donor molecule displaying solution absorption up to 750 nm. Finally, mechanistic studies on the bromoboration reaction indicated it proceeds through the 1,1-bromoboration of the diarylalkynes, a reaction not previously observed using just boron electrophiles. 1,1-Bromoboration can be applied to functionalise other diarylalkynes, enabling access to unprecedented 1-bromo-2,2-diaryl substituted vinylboronate esters direct from internal alkynes. The utility of the 1,1-bromoboration reaction is being studied further in our laboratory as are the brominated-B_2_-PAHs, particularly to access useful functional materials.

## Conflicts of interest

There are no conflicts to declare.

## Supplementary Material

SC-011-C9SC05404A-s001

SC-011-C9SC05404A-s002
