# Supplementary material for: The synthesis of brominated-boron-doped PAHs by alkyne 1,1-bromoboration: mechanistic and functionalisation studies
Source: Chem Sci. 2020 Feb 27;11(12):3258–67. doi: 10.1039/c9sc05404a (PMC8157679; doi:10.1039/c9sc05404a)
Supplement: SC-011-C9SC05404A-s001 [file SC-011-C9SC05404A-s001.pdf]

Electronic Supplementary Material (ESI) for Chemical Science.
This journal is © The Royal Society of Chemistry 2020

The Synthesis of Brominated-Boron-Doped PAHs by Alkyne 1,1-bromoboration: Mechanistic and Functionalisation Studies

K. Yuan, R. J. Kahan, C. Si, A. William, S. Kirschner, M. Uzelac, E. Zysman-Colman* and M. J. Ingleson*

Contents

S1. General Information **2**

S2. Synthesis of Precursors **3**

S3. Synthesis of Brominated-Boron-Doped PAHs **8**

S4. Bromoboration of Simple Internal Alkynes with BBr_3 .... **23**

S5. Photophysical Properties **62**

S6. Electrochemical Properties **65**

S7. NMR Spectra **68**

S8. Crystal Structures **119**

S9. Computational Studies **129**

S10. Detailed Studies on Compound 14 **186**

S1. General Information

Unless stated otherwise, all the experiments were carried out under a N_2 atmosphere using standard Schlenk techniques. Solvents were either obtained from an Inert PureSolv MD5 SPS or distilled from drying reagents: *ortho*-dichlorobenzene (CaH_2), triethylamine (CaH_2). All solvents were stored over 3 Å molecular sieves. Unless otherwise stated all chemicals were purchased from commercial sources and used as received. 3-bromoperylene[1] and 3,9-dibromoperylene[2] were prepared according to reported procedures.

^{1}H, $^{13}C\{^{1}H\}$, ^{11}B and ^{19}F NMR spectra were recorded on Bruker 400, Bruker 500 and Bruker 600 MHz spectrometers. Chemical shifts were referenced to residual solvent signals or external BF_3-etherate. Resonances of carbon atoms directly bonded to boron atoms were not always observed due to the quadrupolar relaxation effects. Note very broad background signals are observed at ca. 0 ppm in the ^{11}B NMR spectra due to borosilicate glass NMR tubes and boron containing parts in the NMR cavity, these are not coincident with the broad compound signals.

Optically dilute solutions of concentrations on the order of 10^{-5} or 10^{-6} M were prepared in HPLC grade toluene for absorption and emission analysis. UV–visible spectra were recorded at room temperature using a Shimadzu UV-1800 double beam spectrometer. Steady-state emission spectra were recorded at 298 K using a Shimadzu RF-6000 fluorimeter.

Cyclic voltammetry (CV) measurements were performed under a N_2 atmosphere using a CH-Instrument 1110C Electrochemical/Analyzer potentiostat. Experiments were conducted using a 1 mM analyte solution with 0.1 M tetrabutylammonium hexafluorophosphate as the supporting electrolyte in THF. A glassy carbon electrode was used as the working electrode with a platinum wire as the counter electrode and an Ag/$AgNO_3$ non-aqueous electrode as the reference electrode. The scan rates were varied between 100 mV s^{-1} and 150 mV s^{-1}. All potentials were calibrated against the ferrocene/ferrocenium (Fc/Fc^+) redox couple.

All exact masses were calculated using the predominant isotopes, which (for the heteroatoms) are: ^{11}B, ^{35}Cl, and ^{79}Br

[1] Maeda, H.; Nanai, Y.; Mizuno, K.; Chiba, J.; Takeshima, S.; Inouye, M. *J. Org. Chem.* **2007**, *72*. 8990.
[2] Hayashi, K; Inouye, M. *Eur. J. Org. Chem.* **2017**, 4334.

S2. Synthesis of Precursors

Synthesis of 1-(pent-1-yn-1-yl)pyrene (**1**)

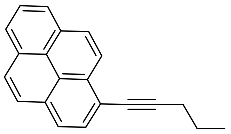

To a mixture of 1-bromopyrene (604 mg, 2.15 mmol, 1 eq.), 1-pentyne (6.0 mL, 61 mmol, 28 eq.) and copper(I) iodide (7.9 mg, 0.041 mmol, 0.02 eq.) was added dry THF (10 mL) and dry triethylamine (5 mL). Under argon flow $Pd(PPh_3)_2Cl_2$ (30.6 mg, 0.044 mmol, 0.02 eq.) was added. The mixture darkened to black within 15 minutes and was heated to 80 °C for 96 hours in a sealed vessel. Volatiles were removed *in vacuo* and the mixture was taken up in dichloromethane and filtered through a plug of silica. The remaining solids were washed with dichloromethane and the washings were combined and dried *in vacuo*. The product was purified by flash column chromatography using a DCM: petrol (5: 95) eluent. Yield: 416 mg, 1.55 mmol, 72%.

^{1}H NMR (500MHz, $CDCl_3$) δ = 8.60 (d, *J* = 9.1 Hz, 1H), 8.21 – 8.00 (m, 8H), 2.66 (t, *J* = 7.0 Hz, 2H), 1.93 – 1.73 (m, 2H), 1.22 (t, *J* = 7.37 Hz, 3H).

^{13}C{^{1}H} NMR (126MHz, $CDCl_3$) δ = 131.8, 131.2, 131.1, 130.6, 129.6, 127.9, 127.6, 127.2, 126.0, 125.6, 125.3, 125.2, 124.4, 124.4, 124.3, 118.9, 96.2, 79.7, 22.5, 21.9, 13.8.

HRMS (APCI) Calculated for $C_{21}H_{17}$ ($[M+H]^+$) 269.1317. Found 269.1325.

Synthesis of 1,6-di(oct-1-yn-1-yl)pyrene (**2**)

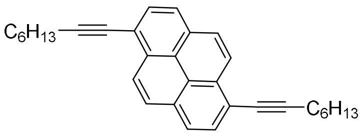

To a mixture of 1,6-dibromopyrene (1.80 g, 5.00 mmol, 1 eq.), 1-octyne (1.86 g, 16.90 mmol, 3.4 eq.) and copper(I) iodide (17 mg, 0.089 mmol, 0.018 eq.) was added dry THF (25 mL) and dry triethylamine (10 mL). Under argon flow $Pd(PPh_3)_2Cl_2$ (75 mg, 0.11 mmol, 0.022 eq.) was added. The mixture darkened to black within 15 minutes and was heated to 80 °C for 96 hours in a sealed vessel. Volatiles were removed *in vacuo* and the mixture was taken up in dichloromethane and filtered through a plug of silica. The remaining solids were washed with dichloromethane and the washings were combined and dried *in vacuo*. The product was purified by flash column chromatography using a DCM: Petrol (2: 98) eluent. Yield: 1.137 g, 2.72 mmol, 54%.

^{1}H NMR (500MHz, $CDCl_3$) δ = 8.55 (d, *J* = 9.0 Hz, 2H), 8.10 – 8.04 (m, 6H), 2.66 (t, *J* = 7.1 Hz, 4H), 1.78 (m, 4H), 1.61 (m, 4H), 1.42 (m, 8H), 0.98 (t, *J* = 6.7 Hz, 6H).

^{13}C{^{1}H} NMR (126MHz, $CDCl_3$) δ = 132.0, 130.6, 129.8, 127.7, 125.9, 124.7, 124.2, 119.2, 96.6, 79.6, 31.4, 29.0, 28.8, 22.6, 19.9, 14.1.

HRMS (APCI) Calculated for $C_{32}H_{35}$ ($[M+H]^+$) 419.2720. Found 419.2733.

Synthesis of 1-((4-(*tert*-butyl)phenyl)ethynyl)pyrene (**3**)

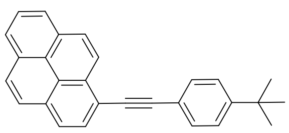

To a mixture of 1-bromopyrene (1.125 g, 4 mmol, 1eq), copper(I) iodide (76 mg, 0.4 mmol, 0.1 eq) and $Pd(PPh_3)_4$ (231 mg, 0.2 mmol, 0.05 eq) was added dry THF (14 mL), dry piperidine (7 mL) and 4-*tert*-butylphenylacetylene (1.44 mL, 8 mmol, 2 eq). The mixture was heated to 85 °C for 60 hours in a sealed vessel. Volatiles were removed *in vacuo* and the mixture was taken up in dichloromethane. The reaction mixture was washed with saturated aqueous ammonium chloride, then brine and the organic layer was collected and dried with $MgSO_4$. The product was separated as a white solid by flash column chromatography using a DCM: hexane (1:100 to 1:10) eluent. Yield: 900 mg, 2.51 mmol, 63%.

^{1}H NMR (500 MHz, $CDCl_3$) δ = 8.68 (d, *J* = 9.1 Hz, 1H), 8.25 – 8.17 (m, 4H), 8.13 (d, *J* = 8.0 Hz, 1H), 8.10 (d, *J* = 8.9 Hz, 1H), 8.07 – 8.00 (m, 2H), 7.67 (d, *J* = 8.5 Hz, 2H), 7.47 (d, *J* = 8.6 Hz, 2H), 1.38 (s, 9H).

^{13}C{^{1}H} NMR (126 MHz, $CDCl_3$) δ = 151.9, 132.0, 131.6, 131.5, 131.3, 129.7, 128.4, 128.2, 127.4, 126.4, 125.8, 125.71, 125.65, 124.69, 124.68, 124.5, 120.7, 118.3, 95.5, 88.1, 35.0, 31.4. Two carbon resonances cannot be observed due to overlap.

HRMS (EI) Calculated for $C_{28}H_{22}$ ($[M]^+$) 358.1716. Found 358.1721.

Synthesis of 1,6-bis((4-(*tert*-butyl)phenyl)ethynyl)pyrene (**9**)

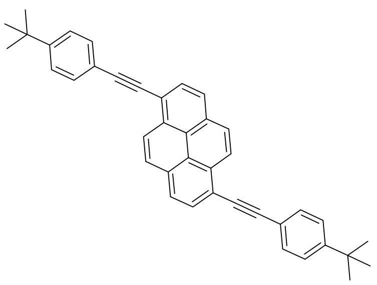

To a mixture of 1,6-dibromopyrene (1.44 g, 4 mmol, 1 eq), copper(I) iodide (76 mg, 0.4 mmol, 0.1 eq) and $Pd(PPh_3)_4$ (139 mg, 0.12 mmol, 0.03 eq) was added dry triethylamine (20 mL) and 4-*tert*-butylphenylacetylene (1.8 mL, 10 mmol, 2.5 eq). The mixture was heated to 105 °C for 60 hours in a sealed vessel. Volatiles were removed *in vacuo* and the mixture was suspended in 60 mL dichloromethane. The suspension was filtered, and the filtrate was discarded. The solid was recrystallized from toluene to yield the desired product as a yellow solid (1.15 g, 2.23 mmol, 56%).

^{1}H NMR (500MHz, $CDCl_3$) δ = 8.69 (d, *J* = 9.1 Hz, 2H), 8.22 (d, *J* = 8.0 Hz, 2H), 8.18 – 8.15 (m, 4H), 7.66 (d, *J* = 8.3 Hz, 4H), 7.46 (d, *J* = 8.3 Hz, 4H), 1.38 (s, 18H).

^{13}C{^{1}H} NMR (126MHz, $CDCl_3$) δ = 152.0, 132.2, 131.6, 131.2, 130.1, 128.4, 126.4, 125.7, 125.3, 124.5, 120.6, 118.9, 95.9, 88.1, 35.1, 31.4.

HRMS (EI) Calculated for $C_{40}H_{34}$ ($[M]^+$) 514.2655. Found 514.2649.

Synthesis of 7-(*tert*-butyl)-1,3-bis((4-(*tert*-butyl)phenyl)ethynyl)pyrene (**10**)

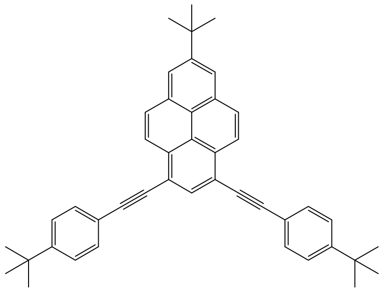

To a mixture of 1,3-dibromo-7-(*tert*-butyl)pyrene (1 g, 2.4 mmol, 1 eq), copper(I) iodide (68.4 mg, 0.36 mmol, 0.15 eq) and $Pd(PPh_3)_4$ (138 mg, 0.12 mmol, 0.05 eq) was added dry toluene (5 mL), dry triethylamine (10 mL) and 4-*tert*-butylphenylacetylene (1.8 mL, 10 mmol, 4.2 eq). The mixture was heated to 90 °C for 48 hours in a sealed vessel. Volatiles were removed *in vacuo* and the mixture was taken up in dichloromethane. The reaction mixture was washed with saturated aqueous ammonium chloride and brine and the organic layer was collected and dried with $MgSO_4$. The product was separated as a yellow solid by flash column chromatography using a hexane eluent. Yield: 810 mg, 1.42 mmol, 59%.

The analytical data is in accordance with the literature.[3]

Synthesis of 3-(*p*-tolylethynyl)perylene (**11**)

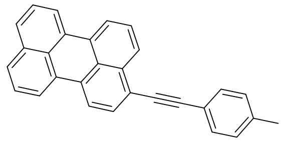

To a mixture of 3-bromoperylene (397 mg, 1.2 mmol, 1 eq.), copper(I) iodide (34 mg, 0.18 mmol, 0.15 eq) and $Pd(PPh_3)_4$ (69 mg, 0.06 mmol, 0.05 eq) was added dry THF (10 mL), dry piperidine (5 mL) and 4-ethynyltoluene (0.38 mL, 3 mmol, 2.5 eq). The mixture was heated to 85 °C for 24 hours in a sealed vessel. Volatiles were removed *in vacuo* and the mixture was taken up in dichloromethane. The reaction mixture was washed with saturated aqueous ammonium chloride and brine. The organic layer was collected and dried with $MgSO_4$. The mixture was purified by flash column chromatography using a DCM: hexane (1:100 to 1:10) eluent to yield the desired product as an orange solid (190 mg, 0.46 mmol, 39%).

^{1}H NMR (400 MHz, $CDCl_3$) δ = 8.31 (dd, *J* = 8.3, 1.0 Hz, 1H), 8.25 (dd, *J* = 7.7, 1.1 Hz, 1H), 8.23 – 8.18 (m, 2H), 8.15 (d, *J* = 7.9 Hz, 1H), 7.73 (d, *J* = 7.9 Hz, 1H), 7.71- 7.68 (m, 2H), 7.61- 7.55 (m, 3H), 7.52 – 7.47 (m, 2H), 7.22 (dt, *J* = 7.9, 0.8 Hz, 2H), 2.41 (s, 3H).

^{13}C{^{1}H} NMR (100 MHz, $CDCl_3$) δ =138.8, 134.82, 134.79, 131.8, 131.70, 131.66, 131.3, 131.04, 130.99, 129.4, 128.74, 128.68, 128.5, 128.2, 127.4, 126.83, 126.76, 126.4, 121.0, 120.9, 120.72, 120.5, 119.9, 95.8, 87.6, 21.7.

HRMS (EI) Calculated for $C_{29}H_{18}$ ($[M]^+$) 366.1403. Found 366.1415.

[3] Cheng, G.; Bonillo, B.; Sprick, R. S.; Adams, D. J.; Hasell, T.; Cooper, A. I. *Adv. Funct. Mater.* **2014**, *24*, 5219.

Synthesis of 3,9-bis((4-(*tert*-butyl)phenyl)ethynyl)perylene (**12**)

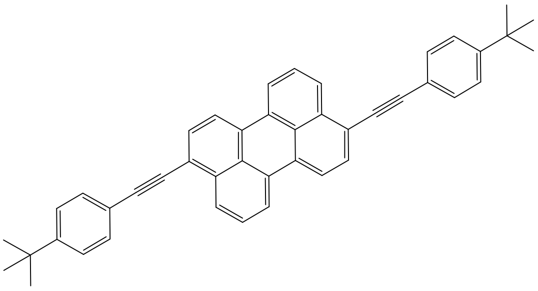

To a mixture of 3,9-dibromoperylene (244 mg, 0.6 mmol, 1 eq.), copper(I) iodide (20 mg, 0.12 mmol, 0.2 eq) and $Pd(PPh_3)_4$ (70 mg, 0.06 mmol, 0.1 eq) was added dry THF (10 mL), dry piperidine (2 mL) and 4-*tert*-butylphenylacetylene (0.43 mL, 2.4 mmol, 4 eq). The mixture was heated to 70 °C for 16 hours in a sealed vessel. Volatiles were removed *in vacuo* and to the mixture was added 18 mL toluene. The desired product was obtained via recrystallization using toluene. Yield: 250 mg, 0.44 mmol, 74%.

The analytical data is in accordance with the literature.[2]

Synthesis of 1,2-bis(3-chlorophenyl)acetylene

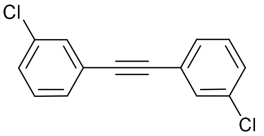

A suspension of 3-chloroethynylbenzene (0.31 mL, 2.5 mmol, 1 eq.), 3-chlorobromobenzene (0.30 mL, 2.5 mmol, 1 eq), $Pd(PPh_3)_2Cl_2$ (18 mg, 25 µmol, 0.01 eq.), PPh_3 (26 mg, 0.1 mmol, 0.04 eq.), and CuI (1 mg, 5 µmol, 0.002 eq.) in dry NEt_3 (12 mL) was degassed by subjecting it to three freeze-pump-thaw cycles. It was then heated to 100 °C for 18 h. The initially yellow suspension turned orange and large amounts of colourless solids were precipitated. After cooling to room temperature, the mixture was filtered through a plug of silica, eluting with EtOAc until the eluate was colourless. The clear filtrate was evaporated to dryness with the aid of a rotary evaporator and the desired product was purified by column chromatography using silica and an n-hexane eluent. Yield: 354 mg, 1.43 mmol, 57%. The analytical data is in accordance with the literature.[4]

^{1}H NMR (500MHz, C_6D_6): δ = 7.43 (t, J = 1.8 Hz, 2H), 7.13 (dt, J = 7.7, 1.3 Hz, 2H), 6.94 (ddd, J = 8.1, 2.2, 1.0 Hz, 2H), 6.66 (t, J = 7.9 Hz, 2H).

Synthesis of 1,2-bis(4-methoxyphenyl)acetylene

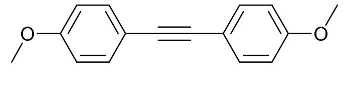

A suspension of 4-ethynylanisole (0.33 mL, 2.5 mmol, 1 eq.), 4-bromoanisole (0.32 mL, 2.5 mmol, 1 eq), $Pd(PPh_3)_2Cl_2$ (19 mg, 25 µmol, 0.01 eq.), PPh_3 (27 mg, 0.1 mmol, 0.04 eq.), and CuI (1 mg, 5 µmol, 0.002 eq.) in dry NEt_3 (12 mL) was degassed by subjecting it to three freeze-pump-thaw cycles. It was then heated to 100 °C for 18 h. The initially yellow suspension turned orange and large amounts of colourless solids were precipitated. After cooling to room temperature, the mixture was filtered through a plug of silica, eluting with EtOAc until the eluate was colourless. The clear filtrate was evaporated to dryness with the aid of a rotary evaporator and the desired product was separated by column chromatography using

[4] M. J. Mio, L. C. Kopel, J. B. Braun, T. L. Gadzikwa, K. L. Hull, R. G. Brisbois, C. J. Markworth and P. A. Grieco, *Org Lett*, 2002, **4**, 3199-3202.

silica and an n-hexane/EtOAc (95:5) eluent. Yield: 399 mg, 1.67 mmol, 67%. The analytical data is in accordance with the literature.[4]

^{1}H NMR (400 MHz, C_6D_6): δ = 7.52 (d, J = 8.9 Hz, 4H), 6.63 (d, J = 8.9 Hz, 4H), 3.19 (s, 6H).

Synthesis of 1,2-bis(2-thienyl)acetylene

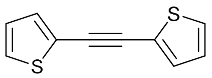

Prepared according to a slightly modified literature procedure:[1] A suspension of 2-bromothiophene (0.49 mL, 5.0 mmol, 1 eq.), ethynyltrimethylsilane (0.35 mL, 2.5 mmol, 0.5 eq.), 1,8-diazabicyclo[5.4.0]undec-7-en (DBU, 4.5 mL, 30 mmol, 6 eq.), water (0.04 mL, 2.0 mmol, 0.4 eq.), $Pd(PPh_3)_2Cl_2$ (218 mg, 300 µmol, 0.06 eq.), and CuI (95 mg, 0.5 mmol, 0.1 eq.) in dry benzene (20 mL) was degassed by subjecting it to three freeze-pump-thaw cycles. It was then heated to 100 °C for 18 h. The resulting deep black suspension was poured into HCl (10% in water, 30 mL) and the layers were separated. The aqueous layer was extracted with EtOAc (3x30 mL) and the combined organic layers, which contained a black colloidal solid, were filtered through a plug of silica, eluting with EtOAc until the eluate was virtually colourless. After washing with saturated aqueous $NaHCO_3$ solution (30 mL) and brine (30 mL), residual water was removed by drying over $MgSO_4$. After filtration and evaporation of the solvent of the filtrate with the aid of a rotary evaporator, the desired product was separated by column chromatography using silica and an n-hexane eluent. Yield: 270 mg, 1.41 mmol, 56%. The analytical data is in accordance with the literature.[4]

^{1}H NMR (400 MHz, C_6D_6): δ = 7.04 (dd, J = 3.6, 1.2 Hz, 1H), 6.63 (dd, J = 5.2, 1.2 Hz, 1H), 6.49 (dd, J = 5.2, 3.6 Hz, 1H).

Synthesis of 1-(3-chlorophenyl)-2-(4-methylphenyl)-acetylene

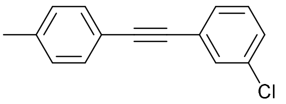

A suspension of 3-chloroethynylbenzene (0.31 mL, 2.5 mmol, 1 eq.), 4-iodotoluene (0.55 g, 2.5 mmol, 1 eq), $Pd(PPh_3)_2Cl_2$ (18 mg, 25 µmol, 0.01 eq.), and CuI (1 mg, 5 µmol, 0.002 eq.) in dry NEt_3 (12 mL) was degassed by subjecting it to three freeze-pump-thaw cycles. It was then heated to 100 °C for 3 h. The initially yellow suspension turned orange and large amounts of colourless solids precipitated. After cooling to room temperature, the mixture was filtered through a plug of silica, eluting with EtOAc until the eluate was colourless. The clear filtrate was evaporated to dryness with the aid of a rotary evaporator and the desired product was separated by column chromatography using silica and an n-hexane eluent. Yield: 452 mg, 1.99 mmol, 80%. The analytical data is in accordance with the literature.[5]

^{1}H NMR (500 MHz, $CDCl_3$): δ = 7.49 (t, J = 1.8 Hz, 1H), 7.45 – 7.39 (m, 2H), 7.38 (dt, J = 7.2, 1.5 Hz, 1H), 7.28 (dt, J = 8.1, 1.8 Hz, 1H), 7.26 – 7.22 (m, 1H), 7.15 (m, 2H), 2.36 (s, 3H).

---

[5] H. Yan, L. Lu, P. Sun, Y. Zhu, H. Yang, D. Liu, G. Rong and J. Mao, *RSC Adv.*, 2013, **3**, 377-381.

S3. Synthesis of Brominated-Boron-Doped PAHs

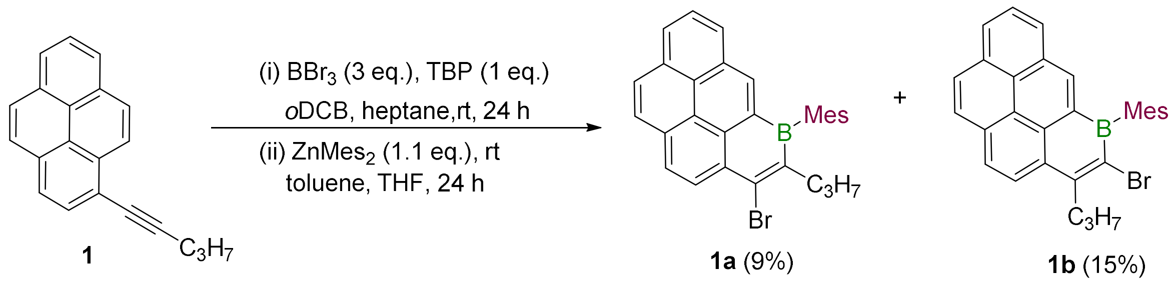

<u>Synthesis of **1a** and **1b**</u>

To an ampoule containing a solution of 1-(pent-1-yn-1-yl)pyrene (44.3 mg, 0.17 mmol, 1 eq.) and 2,4,6-tri-*tert*-butylpyridine (TBP) (50.4 mg, 0.20 mmol, 1.2 eq.) in *ortho*-dichlorobenzene (*o*DCB) (2.0 mL) was added dropwise a solution of boron tribromide (0.5 mL, 1.0 M in hexanes, 0.5 mmol, 3.0 eq.). The mixture instantly develops a dark red colour and was stirred for 24 hours. Volatiles were removed *in vacuo* and to the residue was added dimesityl zinc (56.0 mg, 0.18 mmol, 1.1 eq.). The mixture was taken up in toluene (6.0 mL) and THF (0.3 mL) to give a bright orange suspension. The mixture was stirred for 24 hours then volatiles were removed *in vacuo*. The residue was extracted in hexane and filtered through Celite filter aid to give a bright orange solution. The products were separated by flash column chromatography using silica and a DCM: Petrol (9:1) eluent to yield **1a** (7.3 mg, 9%) and **1b** (10.2 mg, 15%).

<u>1a</u>

^{1}H NMR (500MHz, C_6D_6) δ = 9.29 (d, *J* = 8.4 Hz, 1H), 9.02 (s, 1H), 7.99 (d, *J* = 8.4 Hz, 1H), 7.90 (d, *J* = 7.5 Hz, 1H), 7.80 (d, *J* = 8.8 Hz, 1H), 7.73 (d, *J* = 8.9 Hz, 1H), 7.62 (d, *J* = 7.7 Hz, 1H), 7.50 (dd, *J* = 7.7, 7.6 Hz, 1H), 7.00 (s, 2H), 3.12 – 3.01 (m, 2H), 2.34 (s, 3H), 2.18 (s, 6H), 1.81 (h, *J* = 7.4 Hz, 2H), 0.99 (t, *J* = 7.3 Hz, 3H).

^{11}B NMR (160 MHz, C_6D_6) δ = 60.1.

^{13}C{^{1}H} NMR (126MHz, C_6D_6) δ = 149.4, 149.1, 138.6, 137.2, 133.3, 132.1, 131.1, 131.0, 130.8, 130.2, 129.0, 126.5, 125.9, 125.4, 123.8, 41.0, 23.7, 23.3, 21.5, 15.0. Additional carbon resonances coincide with the benzene resonance (see HSQC).

HRMS (APCI) Calculated for $C_{30}H_{27}BBr$ ([M+H]$^{+}$) 477.1384. Found 477.1380.

The structure of **1a** was further confirmed by single crystal structure analysis (Figure 2).

<u>1b</u>

^{1}H NMR (500MHz, C_6D_6) δ = 9.13 (s, 1H), 8.47 (d, *J* = 8.4 Hz, 1H), 7.99 (d, *J* = 8.4 Hz, 1H), 7.93 (d, *J* = 7.6 Hz, 1H), 7.87 (d, *J* = 8.8 Hz, 1H), 7.79 (d, *J* = 8.9 Hz, 1H), 7.61 (d, *J* = 7.7 Hz, 1H), 7.50 (dd, *J*

= 7.7, 7.6 Hz, 1H), 7.07 (s, 2H), 3.67 – 3.49 (m, 2H), 2.39 (s, 3H), 2.34 (s, 6H), 1.94 (h, J = 7.8 Hz, 2 H), 1.12 (t, J = 7.7 Hz, 3H).

^{11}B NMR (160 MHz, C_6D_6) δ = 58.1.

^{13}C{^{1}H} NMR (126MHz, C_6D_6) δ = 159.9, 148.4, 137.5, 136.0, 132.1, 129.9, 129.8, 129.8, 129.0, 129.0, 126.5, 125.3, 124.9, 124.1, 123.3, 36.7, 22.8, 22.3, 20.4, 13.5. Additional carbon resonances coincide with the benzene resonance (see HSQC).

HRMS (APCI) Calculated for $C_{30}H_{27}BBr$ ([M+H]$^+$) 477.1384. Found 477.1383.

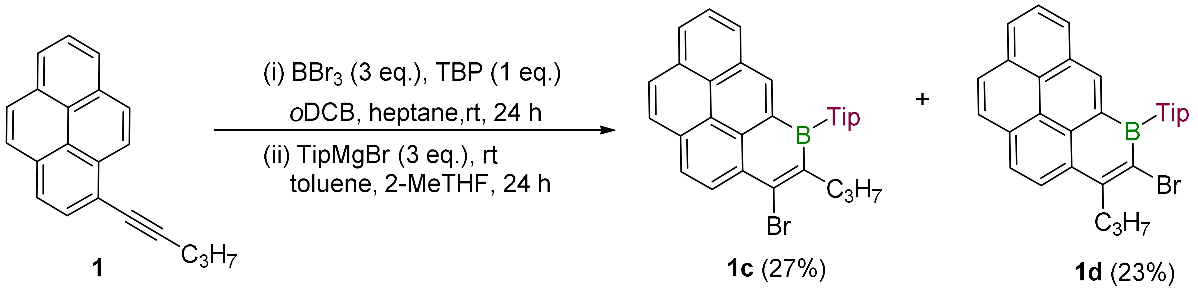

<u>Synthesis of **1c** and **1d**</u>

To an ampoule containing a solution of 1-(pent-1-yn-1-yl)pyrene (44.8 mg, 1.67 x 10^{-4} mol, 1 eq.) and 2,4,6-tri-*tert*-butylpyridine (TBP) (40.6 mg, 1.64 x 10^{-4} mol, 0.98 eq.) in *ortho*-dichlorobenzene was added dropwise a solution of boron tribromide (0.35 mL, 1.0 M in hexanes, 0.35 mmol, 2.1 eq.). The mixture instantly develops a red/brown colour and was stirred for 24 hours. The solvent volume was reduced by 50% to remove residual boron tribromide then to the solution was added 2,4,6-tri*iso*propylphenylmagnesium bromide solution (TipMgBr) solution (1.0 mL, 0.5 M in 2-methyltetrahydrofuran, 0.50 mmol, 3.0 eq.) The mixture was stirred for 24 hours then volatiles were removed *in vacuo*. The residue was extracted in hexane and filtered through Celite filter aid to give a bright orange solution. The products were separated by flash column chromatography using silica and a DCM: hexane (1:9) eluent to yield **1c** (25.1 mg, 27%) and **1d** (21.8 mg, 23%).

<u>**1c**</u>

^{1}H NMR (500MHz, CD_2Cl_2) δ = 9.14 (d, J = 8.4 Hz, 1H), 8.89 (s, 1H), 8.46 (d, J = 7.7 Hz, 1H), 8.44 (d, J = 7.6 Hz, 1H), 8.34 (dd, J = 8.4, 3.4 Hz, 1H), 8.20 (m, 2H), 8.10 (dd, J = 7.7, 7.6 Hz, 1H), 7.15 (s, 2H), 3.03 (hept, J = 6.9 Hz, 1H), 2.77 (m, 2H), 2.46 (m, 2H), 1.63 (h, J = 7.5 Hz, 2H), 1.40 (d, J = 6.9 Hz, 6H), 1.24 (d, J = 6.7 Hz, 6H), 0.94 (m, 9H).

^{11}B NMR (160 MHz, CD_2Cl_2) δ = 59.1.

^{13}C{^{1}H} NMR (126MHz, CD_2Cl_2) δ = 150.0, 149.4, 148.6, 147.6, 138.3, 135.1, 132.7, 131.1, 130.8, 130.8, 130.2, 129.6, 129.6, 128.2, 128.1, 127.7, 126.6, 125.3, 124.9, 123.0, 120.1, 112.2, 40.4, 35.6, 34.4, 24.6, 24.0, 23.4, 22.2, 14.4.

HRMS (APCI) Calculated for $C_{36}H_{39}BBr$ ($[M+H]^+$) 561.2323. Found 561.2326.

The structure of **1c** was further confirmed by single crystal structure analysis.

1d

^1H NMR (500MHz, CD_2Cl_2) δ = 8.91 (s, 1H), 8.74 (d, J = 8.4 Hz, 1H), 8.48 (d, J = 7.6 Hz, 1H), 8.46 (d, J = 7.8 Hz, 1H), 8.37 (d, J = 8.3 Hz, 1H), 8.23 (m, 2H), 8.11 (dd, J = 7.7, 7.6 Hz, 1H), 7.15 (s, 2H), 3.59 (m, 2H), 3.05 (hept, J = 6.9 Hz, 1H), 2.54 (hept, J = 6.7 Hz, 2H), 1.95 (m, 2H), 1.41 (d, J = 7.0 Hz, 6H), 1.26 (m, 9H), 0.97 (d, J = 6.7 Hz, 6H).

^{11}B NMR (160 MHz, CD_2Cl_2) δ = 57.9.

$^{13}C\{^1H\}$ NMR (126MHz, CD_2Cl_2) δ = 160.3, 150.6, 149.7, 149.0, 133.4, 131.4, 131.3, 131.0, 130.3, 130.1, 128.7, 128.4, 128.2, 128.0, 127.2, 126.0, 125.6, 124.2, 120.6, 38.2, 36.5, 34.9, 24.6, 24.5, 24.5, 24.2, 14.9.

HRMS (APCI) Calculated for $C_{36}H_{39}BBr$ ($[M+H]^+$) 561.2323. Found 561.2319.

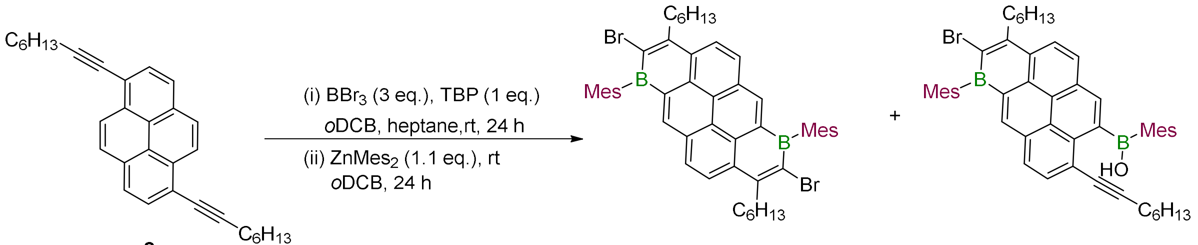

2 **2a** (3%) **2b** (2.5%)

Synthesis of **2a** and **2b**

To an ampoule containing a solution of 1,6-di(pent-1-yn-1-yl)pyrene (83.0 mg, 1.98 x 10^{-4} mol, 1 eq.) and 2,4,6-tri-*tert*-butylpyridine (TBP) (96.7 mg, 3.91 x 10^{-4} mol, 2.0 eq.) in *ortho*-dichlorobenzene (*o*DCB) (4.5 mL) was added dropwise a solution of boron tribromide (0.8 mL, 1.0 M in hexanes, 0.8 mmol, 4.0 eq.). The mixture instantly developed a dark red colour and was stirred for 24 hours. The solvent volume was reduced by 50% to remove residual boron tribromide then to the solution was added dimesityl zinc (124.0 mg, 4.08 x 10^{-4} mol, 2.1 eq.) and *ortho*-dichlorobenzene (2.0 mL). The mixture was stirred for 24 hours then volatiles were removed *in vacuo*. The residue was extracted in hexane and filtered through Celite filter aid to give a bright red solution. The products were separated by flash column chromatography using pH7 alumina and a DCM: Petrol (1:9) eluent to yield **2a** (4.3 mg, 3%) and **2b** (3.2 mg, 2.5%).

Alternative purification of the product mixture by flash column chromatography using silica furnished **2a** (0.7 mg, 1%) and **2b** (15.7 mg, 18%).

2a

The structure of **2a** was confirmed by single crystal structure analysis.

^{1}H NMR (400 MHz, C_6D_6) δ = 8.92 (s, 2H), 8.16 (d, *J* = 8.5 Hz, 2H), 7.58 (d, *J* = 8.4 Hz, 2H), 7.06 (s, 4H), 3.37 (t, *J* = 7.9 Hz, 4H), 2.38 (m, 16H), 1.82 (m, 4H), 1.49 (m, 4H), 1.31 (m, 10H), 0.89 (t, *J* = 6.7 Hz, 6H).

^{13}C{^{1}H} NMR (101 MHz, C_6D_6) Due to the low yield / instability of the compound toward standard purification procedures, we were not able to obtain sufficient quantity pure. Therefore we are unable to assign ^{13}C chemical shifts due to low yield and/or impurities.

HRMS (APCI) Calculated for $C_{50}H_{54}B_2Br_2$ ($[M]^+$) 834.2973. Found 834.2773.

2b

^{1}H NMR (400 MHz, C_6D_6) δ = 9.07 (s, 1H), 8.46 (d, *J* = 8.4 Hz, 1H), 8.13 (s, 1H), 7.96 (d, *J* = 8.0 Hz, 1H), 7.77 (d, *J* = 8.3 Hz, 1H), 7.48 (d, *J* = 8.0 Hz, 1H), 7.08 (s, 2H), 6.94 (s, 2H), 5.32 (s, br, B-O*H*), 3.55 (m, 2H), 2.69 (s, 6H), 2.40 (s, 3H), 2.38 (s, 6H), 2.22 (m, 5H), 1.92 (m, 2H), 1.54 (m, 2H), 1.33 (m, 6H), 1.18 (m, 4H), 1.09 (m, 2H), 0.87 (m, 6H).

^{11}B NMR (128 MHz, C_6D_6) δ = 52.0 (extremely broad, potentially due to two overlapping broad signals for the borinic acid and the triorganoborane).

^{13}C{^{1}H} NMR (101 MHz, C_6D_6) δ = 161.1, 148.8, 144.7, 139.6, 138.4, 136.9, 133.4, 132.3, 132.1, 131.1, 130.3, 130.3, 129.7, 129.2, 125.8, 125.4, 124.1, 123.7, 121.8, 120.8, 119.8, 110.9, 105.2, 102.3, 83.8, 35.8, 31.7, 31.3, 30.3, 29.8, 28.8, 27.8, 24.0, 23.2, 22.7, 22.5, 21.2, 21.0, 20.1, 14.0, 14.0. Additional carbon resonances coincide with the benzene resonance. (See HSQC)

HRMS (APCI) Calculated for $C_{50}H_{56}B_2OBr$ ($[M+H]^+$) 773.3692. Found 773.3695.

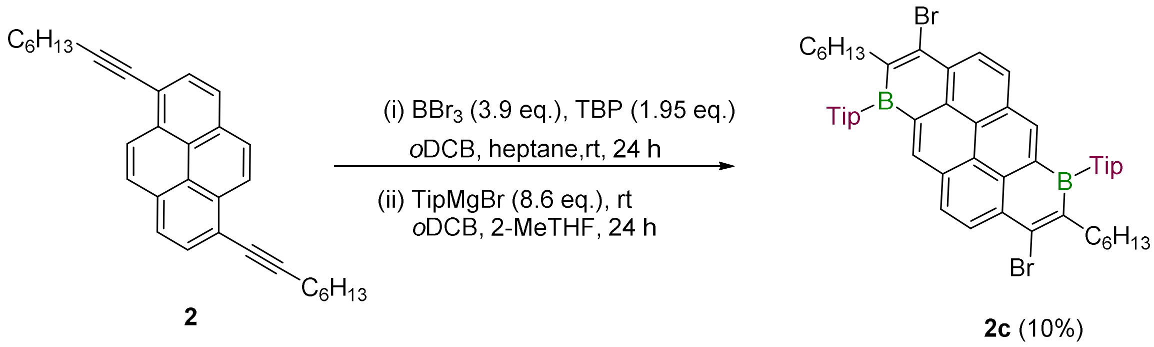

2

2c (10%)

Synthesis of **2c**

To an ampoule containing a solution of 1,6-di(oct-1-yn-1-yl)pyrene (267 mg, 0.64 mmol, 1eq.) and 2,4,6-tri-*tert*-butylpyridine (TBP) (311 mg, 1.25 mmol, 1.95 eq.) in dry *ortho*-dichlorobenzene (*o*DCB) (8 mL) was added dropwise a solution of boron tribromide (2.5 mL, 1.0 M in hexane, 2.5 mmol, 3.9 eq.). The solution turned dark red and was stirred for 24 hours. The solvent volume was reduced *in vacuo* to remove residual boron tribromide. To the remaining solution 2,4,6-tri*iso*propylphenyl

magnesium bromide (TipMgBr) (5.5 mL, 0.5 M in 2-methyltetrahydrofuran, 2.75 mmol, 4.3 eq.). The mixture was stirred for 24 hours. Volatiles were removed *in vacuo* and the residue was extracted in hexane and filtered through Celite filter aid to give a bright red solution. The product **2c** was separated by column chromatography using silica and a DCM: Petrol (1: 9) eluent. Yield: 66 mg, 10%.

^{1}H NMR (400 MHz, CD_2Cl_2) δ = 9.03 (d, *J* = 8.4 Hz, 2H), 8.73 (s, 2H), 8.41 (d, *J* = 8.4 Hz, 2H), 7.14 (s, 4H), 3.02 (sept, *J* = 6.9 Hz, 2H), 2.69-2.74 (m, 4H), 2.44 (q, *J* = 6.6 Hz, 4H), 1.50-1.56 (m, 4H), 1.38 (d, *J* = 6.9 Hz, 12H), 1.32 – 1.17 (m, 24H), 0.96 (d, *J* = 6.6 Hz, 12H), 0.81-0.85 (m, 6H).

^{11}B NMR (128 MHz, CD_2Cl_2) δ = 59.9.

^{13}C{^{1}H} NMR (101 MHz, CD_2Cl_2) δ = 150.3, 149.7, 149.4, 147.0, 134.9, 131.6, 131.2, 130.0, 129.3, 125.2, 120.6, 38.7, 36.1, 34.8, 31.6, 30.0, 28.6, 25.2, 24.4, 23.7, 22.8, 14.2.

HRMS (APCI) Calculated for $C_{62}H_{78}B_2Br_2$ (M^+) 1002.4662. Found 1002.4673.

The structure of compound **2c** was confirmed by single crystal structure analysis.

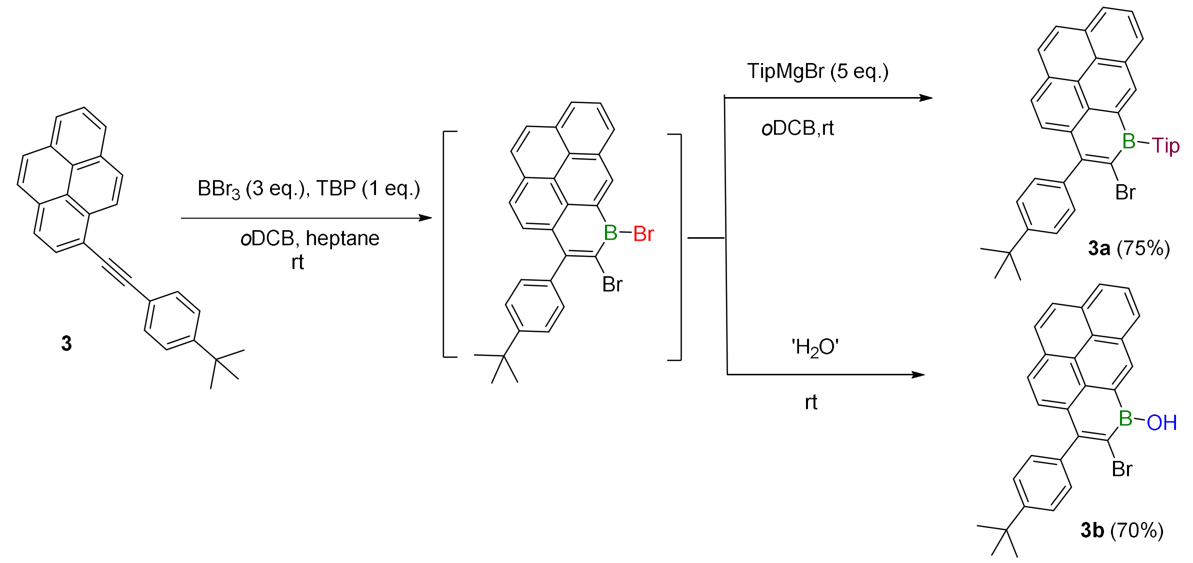

<u>Synthesis of **3a**</u>

To an ampoule containing a solution of 1-((4-(*tert*-butyl)phenyl)ethynyl)pyrene (71.6 mg, 0.2 mmol, 1 eq.) and 2,4,6-tri-*tert*-butylpyridine (TBP) (49.4 mg, 0.2 mmol, 1 eq.) in *ortho*-dichlorobenzene (*o*DCB) (3 mL) was added dropwise a solution of boron tribromide (0.6 mL, 1.0 M in heptane, 0.6 mmol, 3 eq.). The mixture instantly developed a dark red colour and was stirred for 24 hours. The solvent was removed to remove residual boron tribromide then the residue was redissolved in 3 mL *ortho*-dichlorobenzene. To the solution was added 2,4,6-tri*iso*propylphenylmagnesium bromide solution (2.0 mL, 0.5 M in THF, 1mmol, 5 eq.). The mixture was stirred for 48 hours then volatiles were removed *in* vacuo. The residue was extracted in hexane and filtered through Celite filter aid to give a red solution.

The products were separated by flash column chromatography using silica and a hexane: DCM (50:1 to 30:1) eluent to afford **3a** as a red solid. Yield: 98 mg, 0.15 mmol, 75%.

^{1}H NMR (400 MHz, $CDCl_3$) δ = 9.05 (s, 1H), 8.51 - 8.46 (m, 2H), 8.22 - 8.09 (m, 4H), 7.98 (d, 8.3 Hz, 1H), 7.61 (d, *J* = 8.3 Hz, 2H), 7.41 (d, *J* = 8.3 Hz, 2H), 7.14 (s, 2H), 3.05 (p, *J* = 6.9 Hz, 1H), 2.64 (p, *J* = 6.7 Hz, 2H), 1.47 (s, 9H), 1.41 (d, *J* = 6.9 Hz, 6H), 1.28 (d, *J* = 6.7 Hz, 6H), 1.02 (d, *J* = 6.7 Hz, 6H).

^{11}B NMR (160 MHz, $CDCl_3$) δ = 60.1.

^{13}C{^{1}H} NMR (101 MHz, $CDCl_3$) δ = 160.3, 150.6, 150.2, 149.7, 148.6, 139.3, 133.0, 131.5, 131.02 130.97, 130.9, 130.8, 130.0, 129.0, 128.3, 128.1, 127.3, 126.7, 125.8, 125.2, 124.8, 123.7, 120.1, 36.2, 34.9, 34.4, 31.7, 24.5, 24.5, 24.3.

HRMS (EI) Calculated for $C_{43}H_{44}BBr$ ($[M]^+$) 650.2719. Found 650.2700.

The identity of **3a** was confirmed by ^{1}H-^{1}H NOSEY.

<u>Synthesis of **3b**</u>

To an ampoule containing a solution of 1-((4-(*tert*-butyl)phenyl)ethynyl)pyrene (71.6 mg, 0.2 mmol, 1 eq.) and 2,4,6-tri-*tert*-butylpyridine (TBP) (49.4 mg, 0.2 mmol, 1 eq.) in *ortho*-dichlorobenzene (*o*DCB) (3 mL) was added dropwise a solution of boron tribromide (0.6 mL, 1.0 M in heptane, 0.6 mmol, 3 eq.). The mixture instantly develops a dark red colour and was stirred for 20 hours. The solvent was removed and the reaction mixture was redissolved in 5 mL 'wet' ('wet' means non-purified – i.e. used directly from the Winchester) DCM. The solution was stirred under air for 1 hour then was washed with 1 M HCl aqueous solution. The organic layer was collected and dried with $MgSO_4$. The product was purified by flash column chromatography using silica and an ethyl acetate: hexane (1:10) eluent to yield **3b** an orange solid. Yield: 65 mg, 70%.

3b: ^{1}H NMR (601 MHz, CD_2Cl_2) δ = 9.39 (s, 1H), 8.65 – 8.51 (m, 1H), 8.48 – 8.39 (m, 1H), 8.23 – 8.13 (m, 2H), 8.11 (d, *J* = 8.9 Hz, 1H), 8.06 (d, *J* = 8.2 Hz, 1H), 7.75 (d, *J* = 8.1 Hz, 1H), 7.62 (d, *J* = 8.4 Hz, 2H), 7.32 (d, *J* = 8.3 Hz, 2H), 6.20 (s, 1H, B-OH), 1.46 (s, 9H).

^{11}B NMR (160 MHz, $CDCl_3$) δ = 38.3.

^{13}C{^{1}H} NMR (101 MHz, $CDCl_3$) δ = 160.3, 150.9, 140.4, 138.4, 132.9, 131.1, 130.6, 129.83, 129.76,

129.4, 129.3, 128.6, 128.4, 128.2, 127.9, 126.6, 125.6, 125.4, 124.5, 123.9, 35.0, 31.6.

HRMS (EI) Calculated for $C_{28}H_{22}BOBr$ ($[M]^+$) 464.0947. Found 464.0923.

The identity of **3b** was confirmed by crystal structure analysis and ^{1}H-^{1}H NOSEY.

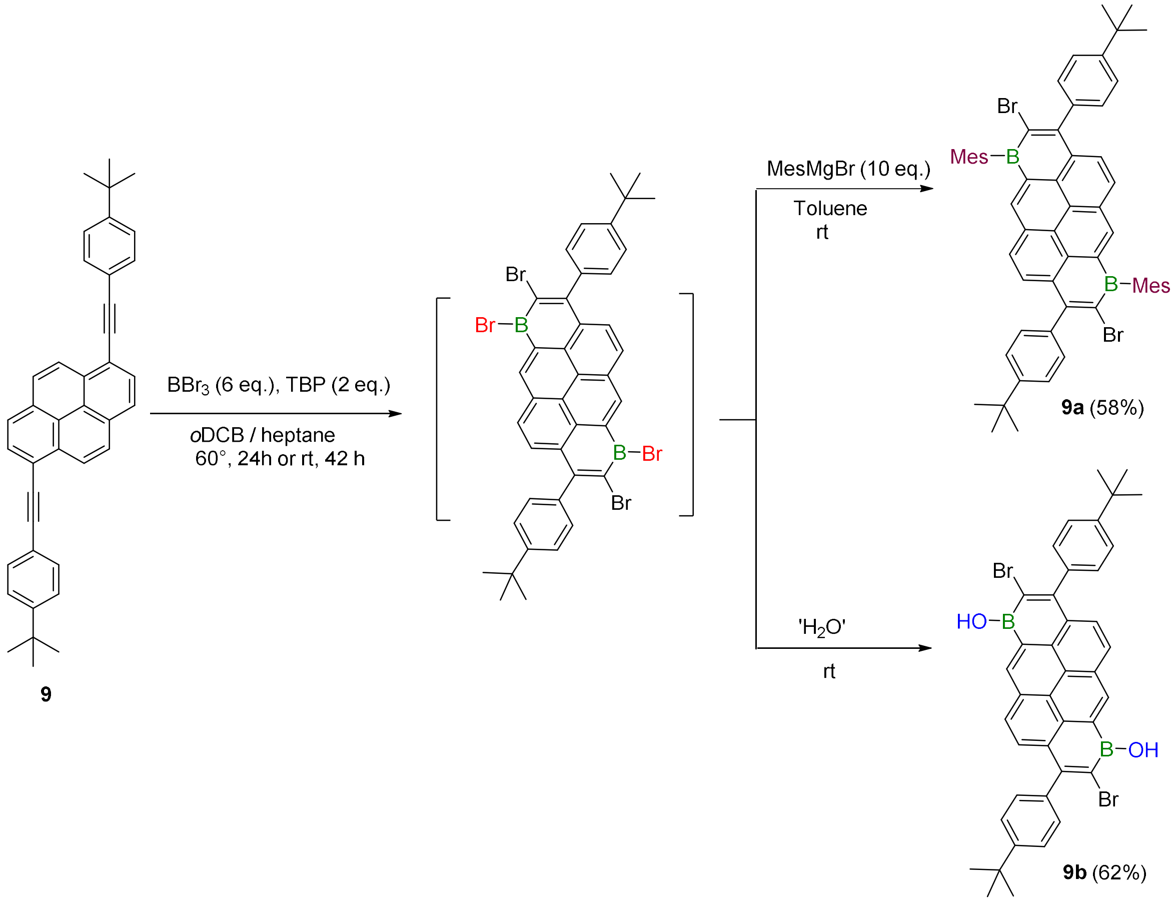

<u>Synthesis of **9a**</u>

To an ampoule containing a solution of 1,6-bis((4-(*tert*-butyl)phenyl)ethynyl)pyrene (255 mg, 0.5mmol, 1 eq.) and 2,4,6-tri-*tert*-butylpyridine (TBP) (247 mg, 1.0 mmol, 2 eq.) in *ortho*-dichlorobenzene (*o*DCB) (5 mL) was added dropwise a solution of boron tribromide (3 mL, 1.0 M in heptane, 3 mmol, 6 eq.). The mixture instantly developed a dark red colour and was stirred for 42 hours. Then the solvent was removed to get rid of residual boron tribromide and the residue was redissolved in 10 mL toluene. To the solution was added mesitylmagnesium bromide solution (MesMgBr) (5.0 mL, 1 M in Et_2O, 5 mmol, 10 eq.). The mixture was stirred for 24 hours then volatiles were removed *in vacuo*. The residue was extracted in hexane and dichloromethane and filtered through Celite filter aid to give a red solution. The product was separated by flash column chromatography using silica and a DCM: hexane (1:10 to 1:5) eluent to afford **9a** as a red solid. Yield: 270 mg, 0.29 mmol, 58%.

^{1}H NMR (500 MHz, $CDCl_3$) δ = 8.79 (s, 2H), 8.18 (d, J = 8.4 Hz, 2H), 7.84 (d, J = 8.3 Hz, 2H), 7.60 (d, J = 8.3 Hz, 4H), 7.35 (d, J = 8.3 Hz, 4H), 6.99 (s, 4H), 2.43 (s, 6H), 2.21 (s, 12H), 1.46 (s, 18H).

^{11}B NMR (160 MHz, $CDCl_3$) δ = 59.1.

^{13}C{^{1}H} NMR (126 MHz, $CDCl_3$) δ = 161.4, 151.1, 149.9, 138.7, 138.4, 137.5, 136.6, 131.4, 131.1, 130.1, 128.7, 128.2, 127.2, 126.0, 125.5, 35.0, 31.6, 23.3, 21.6.

HRMS (EI) Calculated for $C_{58}H_{54}B_2Br_2$ ($[M]^+$) 930.2778. Found 930.2773.

The structure of **9a** was further confirmed by single crystal structure analysis.

Synthesis of **9b**

To an ampoule containing a solution of 1,6-bis((4-(*tert*-butyl)phenyl)ethynyl)pyrene (154 mg, 0.3 mmol, 1 eq.) and 2,4,6-tri-*tert*-butylpyridine (TBP) (148 mg, 0.6 mmol, 2 eq.) in *ortho*-dichlorobenzene (*o*DCB) (5 mL) was added dropwise a solution of boron tribromide (1.8 mL, 1.0 M in heptane, 1.8 mmol, 6 eq.). The mixture instantly develops a dark red colour and was stirred for 42 hours. The solvent was removed, and the residue was redissolved in 5 mL 'wet' dichloromethane. The solution was stirred under air for 1 hour to form a suspension. The suspension was washed with 1 M HCl aqueous solution. The solid was collected by filtration and washed with dichloromethane to yield **9b** as a dark grey solid. Yield: 136 mg, 62%.

^{1}H NMR (400 MHz, THF-*d₈*) δ = 9.17 (s, 2H), 8.79 (s, 2H, B-OH), 8.25 (d, *J* = 8.4 Hz, 2H), 7.71 (d, *J* = 8.2 Hz, 2H), 7.63 (d, *J* = 8.5 Hz, 4H), 7.30 (d, *J* = 8.4 Hz, 4H), 1.46 (s, 18 H).

^{11}B NMR (128 MHz, THF-d_8) δ = 36.4.

$^{13}C\{^{1}H\}$ NMR (101 MHz, THF-d_8) δ = 160.8, 151.5, 140.6, 139.8, 135.2, 132.0, 130.4, 129.9, 129.6, 128.9, 126.3, 126.0, 35.6, 32.0.

HRMS (EI) Calculated for $C_{40}H_{34}B_2Br_2O_2$ ($[M]^+$) 726.1112. Found 726.1081.

The identity of **9b** was confirmed by ^{1}H-^{1}H NOSEY.

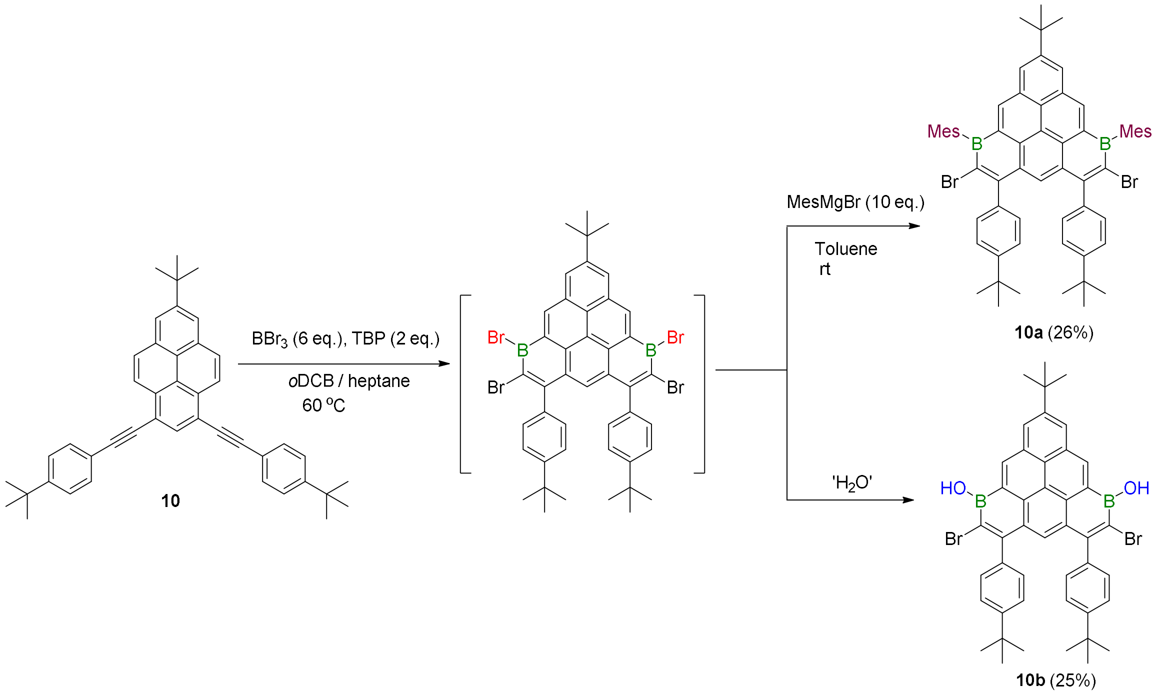

Synthesis of **10a**

To an ampoule containing a solution of 7-(*tert*-butyl)-1,3-bis((4-(*tert*-butyl)phenyl)ethynyl)pyrene (114 mg, 0.2 mmol, 1 eq.) and 2,4,6-tri-*tert*-butylpyridine (TBP) (99 mg, 0.4 mmol, 2 eq.) in *ortho*-dichlorobenzene (*o*DCB) (3 mL) was added dropwise a solution of boron tribromide (0.6 mL, 1.0 M in heptane, 0.6 mmol, 6 eq.). The solution was stirred at 60 °C for 24 hours. The mixture develops a red colour in 5 minutes and was stirred at 60 °C for 40 hours. The solvent was removed to remove residual boron tribromide then the resultant mixture was redissolved in 6 mL toluene. To the solution was added mesitylmagnesium bromide solution (3.0 mL, 1 M in Et_2O, 2 mmol, 10 eq.). The mixture was stirred for 24 hours then volatiles were removed *in vacuo*. The residue was extracted in hexane and dichloromethane and filtered through Celite filter aid to give a red solution. The product was separated by flash column chromatography using silica and a DCM: hexane (1:10 to 1:5) eluent to afford the desired product as a red solid. Yield: 50 mg, 0.051 mmol, 26%.

^{1}H NMR (400 MHz, $CDCl_3$) δ = 9.12 (s, 2H), 8.82 (s, 2H), 8.44 (s, 1H), 7.40 (d, *J* = 8.5 Hz, 4H), 7.22 (d, *J* = 8.4 Hz, 4H), 7.04 (s, 4H), 2.47 (s, 6H), 2.21 (s, 12H), 1.62 (s, 9H), 1.42 (s, 18H).

^{11}B NMR (160 MHz, $CDCl_3$) δ = 59.2.

^{13}C{^{1}H} NMR (101 MHz, $CDCl_3$) δ = 162.1, 151.0, 150.7, 149.8, 138.8, 138.4, 137.3, 135.9, 134.3, 130.9, 129.9, 128.9, 128.7, 127.2, 125.2, 124.7, 122.9, 35.7, 34.8, 31.9, 31.7, 23.3, 21.7.

HRMS (EI) Calculated for $C_{62}H_{62}B_2Br_2$ ($[M]^+$) 988.3405. Found 988.3395.

The identity of **10a** was confirmed by ^{1}H-^{1}H NOSEY.

Synthesis of **10b**

To an ampoule containing a solution of 7-(*tert*-butyl)-1,3-bis((4-(*tert*-butyl)phenyl)ethynyl)pyrene (114 mg, 0.2 mmol, 1 eq.) and 2,4,6-tri-*tert*-butylpyridine (TBP) (99 mg, 0.4 mmol, 2 eq.) in *ortho*-dichlorobenzene (*o*DCB) (3 mL) was added dropwise a solution of boron tribromide (0.6 mL, 1.0 M in heptane, 0.6 mmol, 6 eq.). The solution was stirred at 60 °C for 24 hours. The solvent was reduced to half volume to remove residual boron tribromide and then the solution was subjected to flash column chromatography directly using silica and a DCM: hexane (1:100 to 1:2) eluent to afford the desired product as a red solid. Yield: 38 mg, 0.049 mmol, 25%.

^{1}H NMR (500 MHz, $CDCl_3$) δ = 9.41 (s, 2H), 8.76 (s, 2H), 7.95 (s, 1H), 7.32 (d, *J* = 8.3 Hz, 4H), 7.05 (d, *J* = 8.2 Hz, 4H), 6.11 (s, 2H, B-OH), 1.68 (s, 9H), 1.38 (s, 18H).

^{11}B NMR (160 MHz, $CDCl_3$) δ = 38.1.

^{13}C{^{1}H} NMR (126 MHz, $CDCl_3$) δ = 160.5, 150.7, 150.5, 141.0, 137.7, 133.0, 130.8, 129.78, 129.76, 129.74, 128.4, 124.8, 124.7, 123.2, 35.5, 34.8, 32.0, 31.7.

HRMS (MALDI) Calculated for $C_{44}H_{42}B_2Br_2O_2$ ([M]$^+$) 784.1731. Found 784.1719.

The structure of **10b** was further confirmed by single crystal structure analysis.

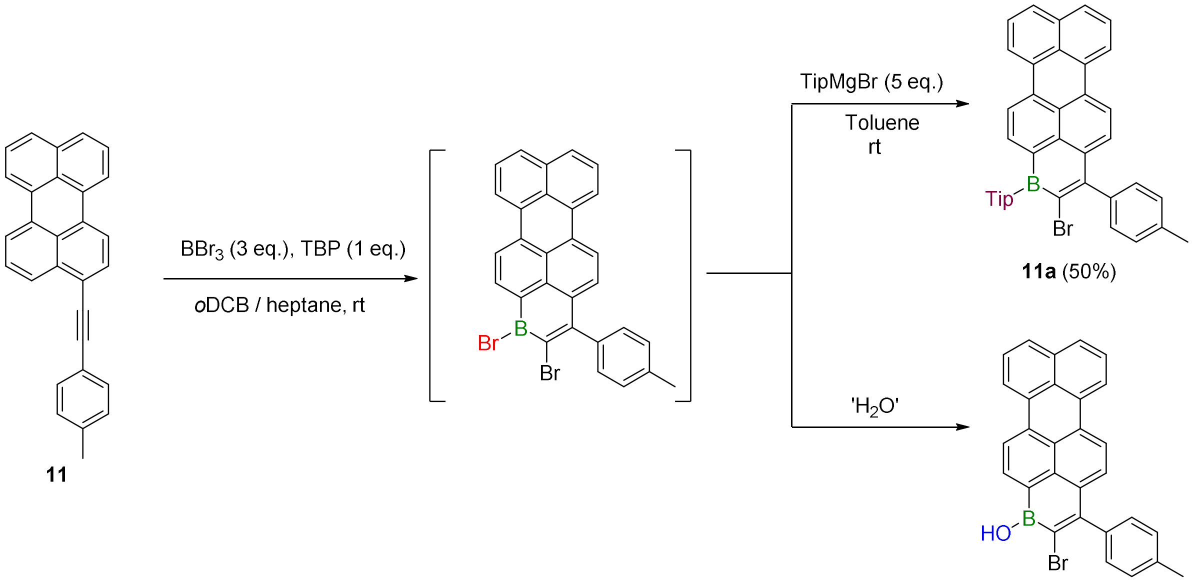

11

11a (50%)

11b (88%)

Synthesis of **11a**

To an ampoule containing a solution of 3-(*p*-tolylethynyl)perylene (37 mg, 0.1 mmol, 1 eq.) and 2,4,6-tri-*tert*-butylpyridine (TBP) (25 mg, 0.1 mmol, 1 eq.) in *ortho*-dichlorobenzene (*o*DCB) (2 mL) was added dropwise a solution of boron tribromide (0.3 mL, 1.0 M in heptane, 0.3 mmol, 3 eq.). The solution was stirred at room temperature for 24 hours. The solvent was removed, and the reaction mixture was

redissolved in 5 mL toluene. To the solution was added 2,4,6-tri*iso*propylphenylmagnesium bromide solution (1.0 mL, 0.5 M in THF, 1 mmol, 5 eq.). The mixture was stirred for 48 hours then volatiles were removed *in vacuo*. The residue was extracted in hexane and filtered through Celite filter aid to give a red solution. The products were separated by flash column chromatography using a DCM: hexane (1:100 to 1:10) eluent. eluent to afford the desired product as a dark red solid. Yield: 33 mg, 0.05 mmol, 50%.

^{1}H NMR (500 MHz, $CDCl_3$) δ = 8.54 (d, *J* = 7.5 Hz, 1H), 8.52 (d, *J* = 7.8 Hz, 1H), 8.46 (d, *J* = 7.4 Hz, 1H), 8.33-8.30 (m, 2H), 7.92-7.88 (m, 2H), 7.73 (d, *J* = 8.3 Hz, 1H), 7.65-7.60 (m, 2H), 7.39 (d, *J* = 7.8 Hz, 2H), 7.34 (d, *J* = 8.0 Hz, 2H), 7.09 (s, 2H), 3.01 (p, *J* = 6.9 Hz, 1H), 2.58 (p, *J* = 6.7 Hz, 2H), 2.52 (s, 3H), 1.38 (d, *J* = 6.9 Hz, 6H), 1.27 (d, *J* = 6.7 Hz, 6H), 1.05 (d, *J* = 6.7 Hz, 6H).

^{11}B NMR (160 MHz, $CDCl_3$) δ = 56.7.

^{13}C{H} NMR (126 MHz, $CDCl_3$) δ = 157.6, 150.1, 148.3, 144.7, 139.1, 138.5, 137.4, 134.8, 134.3, 134.1, 133.3, 130.9, 130.6, 130.3, 130.04, 129.98, 129.5, 129.0, 127.2, 127.1, 127.1, 127.0, 124.4, 123.7, 120.5, 120.0, 119.9, 35.9, 34.3, 24.5, 24.4, 24.3, 21.6.

HRMS (EI) Calculated for $C_{44}H_{40}BBr$ ($[M]^+$) 658.2401. Found 658.2394.

The identity of **11a** was confirmed by ^{1}H-^{1}H NOSEY.

Synthesis of **11b**

To an ampoule containing a solution of 3-(*p*-tolylethynyl)perylene (30 mg, 0.08 mmol, 1 eq.) and 2,4,6-tri-*tert*-butylpyridine (TBP) (20 mg, 0.08 mmol, 1 eq.) in *ortho*-dichlorobenzene (*o*DCB) (2 mL) was added dropwise a solution of boron tribromide (0.24 mL, 1.0 M in heptane, 0.24 mmol, 3 eq.). The solution was stirred at room temperature for 40 hours. The solvent was removed, and the reaction mixture was redissolved in 5 mL 'wet' DCM. The solution was stirred under air for 1 hour and then was washed with 1 M HCl aqueous solution. The organic layer was collected. The products were separated by flash column chromatography using silica and a DCM: hexane (1:2 to 2:1) eluent to afford the desired product as a dark grey solid. Yield: 34 mg, 0.07 mmol, 88%.

^{1}H NMR (400 MHz, $CDCl_3$) δ = 8.65 (d, *J* = 7.6 Hz, 1H), 8.57 – 8.41 (m, 2H), 8.33 (dd, *J* = 7.6, 1.0 Hz, 1H), 8.15 (d, *J* = 8.2 Hz, 1H), 7.84 (t, *J* = 8.3 Hz, 2H), 7.61 (t, *J* = 7.8 Hz, 1H), 7.57 (t, *J* = 7.8 Hz, 1H), 7.43 (d, *J* = 8.2 Hz, 1H), 7.40 – 7.33 (m, 2H), 7.23 (d, *J* = 8.0 Hz, 2H), 5.91 (s, 1H, B-OH), 2.51 (s, 3H).

^{11}B NMR (128 MHz, $CDCl_3$) δ = 36.0.

$^{13}C\{^1H\}$ NMR (101 MHz, $CDCl_3$) δ = 158.1, 138.2, 137.7, 136.8, 136.6, 134.3, 134.0, 132.9, 132.8, 132.6, 130.5, 130.3, 130.1, 129.7, 129.14, 129.08, 127.6, 127.4, 127.04, 127.03, 123.3, 122.6, 120.4, 119.6, 21.6.

HRMS (EI) Calculated for $C_{29}H_{18}BBrO$ ($[M]^+$) 472.0629. Found 472.0681.

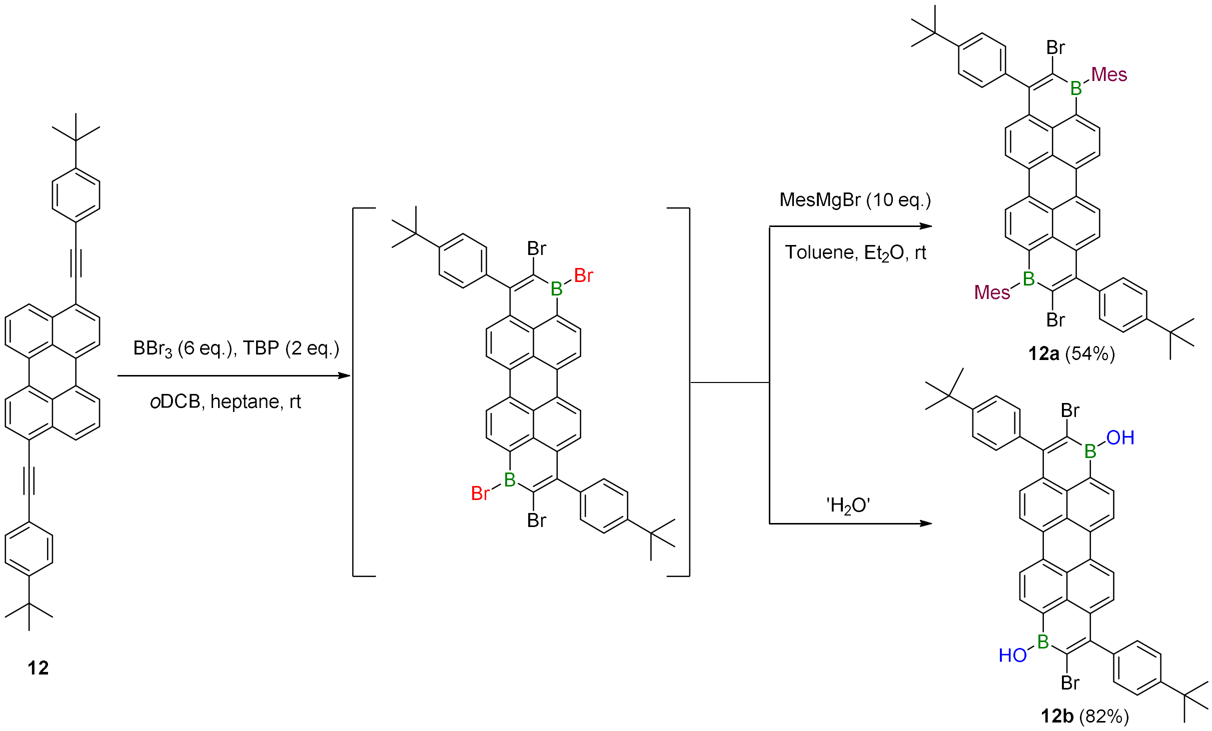

<u>Synthesis of **12a**</u>

To an ampoule containing a solution of 3,9-bis((4-(*tert*-butyl)phenyl)ethynyl)perylene (57 mg, 0.1 mmol, 1 eq.) and 2,4,6-tri-*tert*-butylpyridine (TBP) (53 mg, 0.22 mmol, 2.2 eq.) in *ortho*-dichlorobenzene (*o*DCB) (2.5 mL) was added dropwise a solution of boron tribromide (0.6 mL, 1.0 M in heptane, 0.6 mmol, 6 eq.). The mixture instantly develops a dark blue colour and was stirred at room temperature for 40 hours. Volatiles were removed in-vacuo and the residue was redissolved in 5 mL toluene. To the solution was added mesitylmagnesium bromide solution (1.0 mL, 1 M in Et_2O, 1 mmol, 10 eq.). The mixture was stirred for 24 hours then volatiles were removed *in vacuo*. The residue was extracted in hexane and dichloromethane and filtered through Celite filter aid to give a dark blue solution. The products were separated by flash column chromatography using silica and a DCM: hexane (1:10 to 1:5) eluent to afford the desired product as a dark blue solid. Yield: 53 mg, 0.054 mmol, 54%.

^1H NMR (400 MHz, CD_2Cl_2) δ = 8.55 (d, J = 7.8 Hz, 2H), 8.47 (d, J = 8.4 Hz, 2H), 8.17 (d, J = 7.7 Hz, 2H), 7.64 (d, J = 8.3 Hz, 2H), 7.59 (d, J = 8.4 Hz, 4H), 7.34 (d, J = 8.4 Hz, 4H), 6.93 (s, 4H), 2.37 (s, 6H), 2.13 (s, 12H), 1.44 (s, 18H).

^{11}B resonance was not observed despite multiple attempts using concentrated samples and a quartz NMR tube, this is presumably due to a large pwhh for this resonance.

$^{13}C\{^1H\}$ NMR (101 MHz, $CDCl_3$) δ = 159.3, 151.0, 144.3, 138.5, 138.2, 137.4, 137.3, 136.2, 135.1, 133.1, 131.0, 129.0, 127.1, 126.4, 125.2, 123.5, 123.0, 35.0, 31.6, 23.2, 21.6.

HRMS (MALDI) Calculated for $C_{62}H_{56}B_2Br_2^+$ ($[M]^+$) 982.2909. Found 982.2910.

The identity of **12a** was further confirmed by crystal structure analysis and ^1H-^1H NOSEY.

<u>Synthesis of **12b**</u>

To an ampoule containing a solution of 3,9-bis((4-(*tert*-butyl)phenyl)ethynyl)perylene (33 mg, 0.06 mmol, 1 eq.) and 2,4,6-tri-*tert*-butylpyridine (TBP) (29 mg, 0.12 mmol, 2 eq.) in *ortho*-dichlorobenzene (*o*DCB) (5 mL) was added dropwise a solution of boron tribromide (0.35 mL, 1.0 M in heptane, 0.35 mmol, 6 eq.). The solution was stirred at room temperature for 40 hours. The solvent was removed, and the residue was redissolved in 5 mL 'wet' dichloromethane. The obtained solution was stirred under air for 1 hour to form a dark grey suspension, which was filtered to obtain a dark grey solid. The solid was washed with dichloromethane and methanol to get the desired product. Yield: 38 mg, 0.05 mmol, 82%.

^1H NMR (400 MHz, THF-d_8) δ = 8.69 – 8.58 (m, 4H), 8.46 (d, J = 8.3 Hz, 2H, B-OH), 7.59 (d, J = 8.4 Hz, 4H), 7.36 (d, J = 8.2 Hz, 2H), 7.25 (d, J = 8.4 Hz, 4H), 1.73 (s, 18H).

^{11}B NMR (128 MHz, THF-d_8) δ = 35.4.

$^{13}C\{^1H\}$ NMR (101 MHz, THF-d_8) δ = 159.1, 151.4, 139.5, 137.0, 136.1, 135.7, 133.8, 133.7, 133.2, 129.8, 127.7, 126.1, 123.2, 122.7, 35.6, 32.0.

HRMS (MALDI) Calculated for $C_{44}H_{36}B_2Br_2O_2$ ($[M]^+$) 777.1183. Found 777.1211.

The identity of **12b** was confirmed by ^1H-^1H NOSEY.

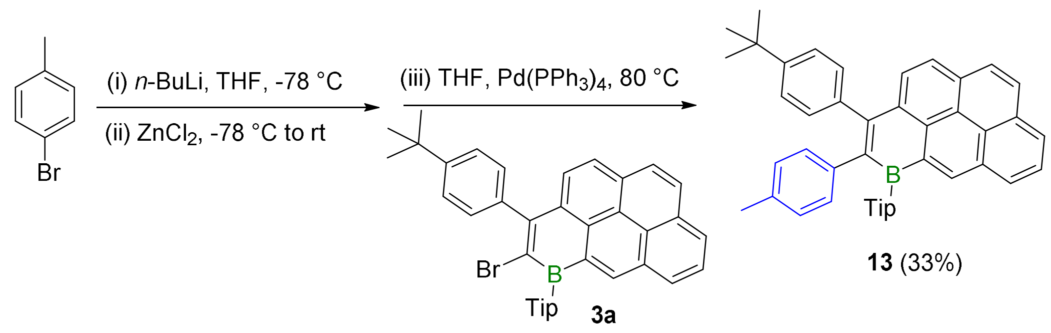

<u>Synthesis of **13**</u>

4-Bromotoluene (43 mg, 0.25 mmol, 1.7 eq.) was dissolved in 3 mL THF and the solution was cooled to -78 °C. To the solution was added dropwise *n*-BuLi (0.18 mL, 1.6 M in pentane, 0.28 mmol, 1.8 eq.). The resultant mixture was stirred at -78 °C for an hour. Then to the mixture was added $ZnCl_2$ (34 mg in 2 mL THF, 0.25 mmol, 1.7 eq.) at -78 °C. The solution was allowed to gradually warm to room temperature and stirred for an hour. To the solution was added a solution of compound **3a** (98 mg, 0.15 mmol, 1 eq.) and $Pd(PPh_3)_4$ (17 mg, 0.015 mmol, 0.1 eq.). The reaction was stirred at 80 °C for 24 hours in a sealed ampoule. The reaction was quenched with saturated aqueous ammonium chloride and then extracted with dichloromethane. The products were separated by flash column chromatography using silica and a DCM: hexane (1:20 to 1:10) eluent to afford the desired product as a red solid. Yield: 32 mg, 0.05 mmol, 33%.

^{1}H NMR (400 MHz, $CDCl_3$) δ = 8.95 (s, 1H), 8.46 – 8.42 (m , 2H), 8.29 – 8.15 (m, 4H), 8.08 (t, *J* = 7.7 Hz, 1H), 7.30 (d, *J* = 8.3 Hz, 2H), 7.23 (d, *J* = 8.3 Hz, 2H), 6.94 (s, 2H), 6.74 (d, *J* = 8.0 Hz, 2H), 6.66 (d, *J* = 8.0 Hz, 2H), 2.95 (p, *J* = 6.9 Hz, 1H), 2.56 (p, *J* = 6.6 Hz, 2H), 2.12 (s, 3H), 1.35 (s, 9H), 1.33 (d, *J* = 6.9 Hz, 6H), 0.96 – 0.92 (m, 12H).

^{11}B NMR (128 MHz, $CDCl_3$) δ = 58.8.

^{13}C{^{1}H} NMR (101 MHz, $CDCl_3$) δ = 157.6, 149.9, 149.4, 148.4, 148.1, 141.0, 138.5, 133.4, 132.5, 131.8, 131.0, 131.0, 130.7, 130.14, 130.12, 130.07, 130.0, 128.1, 127.9, 127.7, 127.2, 126.4, 125.9, 124.4, 124.2, 123.5, 119.7, 35.6, 34.7, 34.4, 31.6, 25.2, 24.4, 23.6, 21.1.

HRMS (EI) Calculated for $C_{50}H_{51}B^{+}$ ([M]$^{+}$) 662.4078. Found 662.4078.

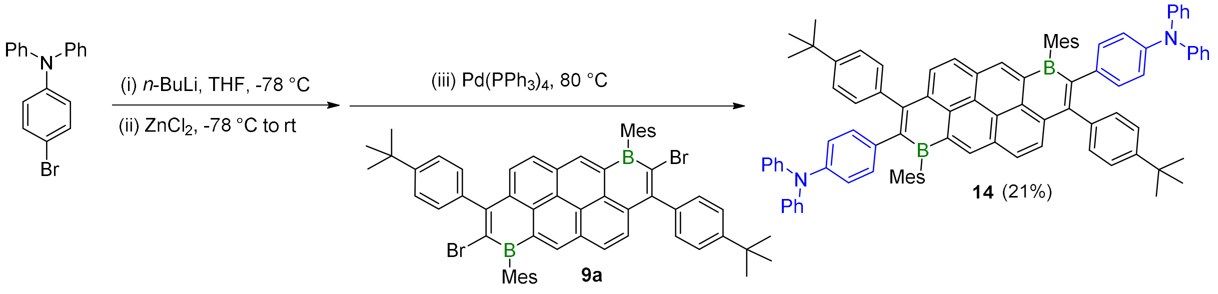

<u>Synthesis of **14**</u>

4-Bromo-*N*,*N*-diphenylaniline (115 mg, 0.354 mmol, 3 eq.) was dissolved in 3 mL THF and the solution was cooled to -78 °C. To the solution was added dropwise *n*-BuLi (0.24 mL, 1.6 M in pentane, 0.39 mmol, 3.3 eq.). The resultant mixture was stirred at -78 °C for an hour. Then to the mixture was added $ZnCl_2$ (49 mg in 1.5 mL THF, 0.354 mmol, 3 eq.) at -78 °C. The reaction was allowed to gradually warm to room temperature and stirred for an hour. To the solution was added compound **9a** (110 mg, 0.12 mmol, 1 eq.) and $Pd(PPh_3)_4$ (17 mg, 0.015 mmol, 0.1 eq.) in THF. The reaction was stirred at 80 °C for 17 hours in a sealed ampoule. The reaction was quenched with saturated aqueous ammonium chloride and extracted with dichloromethane. Compound **14** was isolated by flash column

chromatography using silica and a hexane: DCM (5:1) eluent. Compound **14** was further purified by washing with hexane. Yield: **14**: 32 mg, 0.025 mmol, 21%.

^{1}H NMR (500 MHz, $CDCl_3$) δ = 8.84 (s, 2H), 8.25 (d, *J* = 8.4 Hz, 2H), 8.12 (d, *J* = 8.3 Hz, 2H), 7.34 (d, *J* = 8.4 Hz, 4H), 7.22 (d, *J* = 8.3 Hz, 4H), 7.17 – 7.11 (m, 8H), 6.91 (t, *J* = 7.3 Hz, 4H), 6.87 (d, *J* = 7.6 Hz, 8H), 6.80 (s, 4H), 6.65 (d, *J* = 8.7 Hz, 4H), 6.61 (d, *J* = 8.7 Hz, 4H), 2.35 (s, 7H), 2.09 (s, 12H), 1.37 (s, 18H).

^{11}B NMR (160 MHz, $CDCl_3$) δ = 55.7.

^{13}C{^{1}H} NMR (126 MHz, $CDCl_3$) δ = 158.3, 150.0, 148.3, 148.0, 144.0, 139.7, 138.5, 137.6, 136.6, 136.3, 131.4, 130.8, 130.7, 130.3, 129.5, 129.0, 128.6, 126.8, 125.9, 124.5, 123.7, 123.3, 121.9, 34.8, 31.6, 23.6, 21.4.

HRMS (MALDI) Calculated for $C_{94}H_{82}B_2N_2$ ($[M]^+$) 1261.6707. Found 1261.6698.

S4. Bromoboration of Simple Internal Alkynes with BBr_3

S4.1.1 Bromoboration of diphenylacetylene in heptane at room temperature

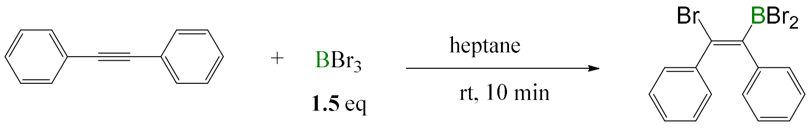

To an ampoule charged with diphenylacetylene (36 mg, 0.2 mmol, 1 eq) was added a solution of boron tribromide (0.3 mL, 1 M in heptane, 0.3 mmol, 1.5 eq.). A clear light-yellow solution was formed immediately upon gently swirling the ampoule. On standing without stirring, crystals suitable for single crystal X-ray diffraction studies formed within 5 minutes. The crystal structure indicated the formation of the 1,2-*cis*-bromoboration product **4** (for crystal structure see Scheme 3). NMR experiments under the exact same conditions confirmed the formation of a single haloboration product in 10 minutes (Figure S1 and Figure S2). The identity of compound **4** was further confirmed by protodeboronation reactions (See **S4.1.3**).

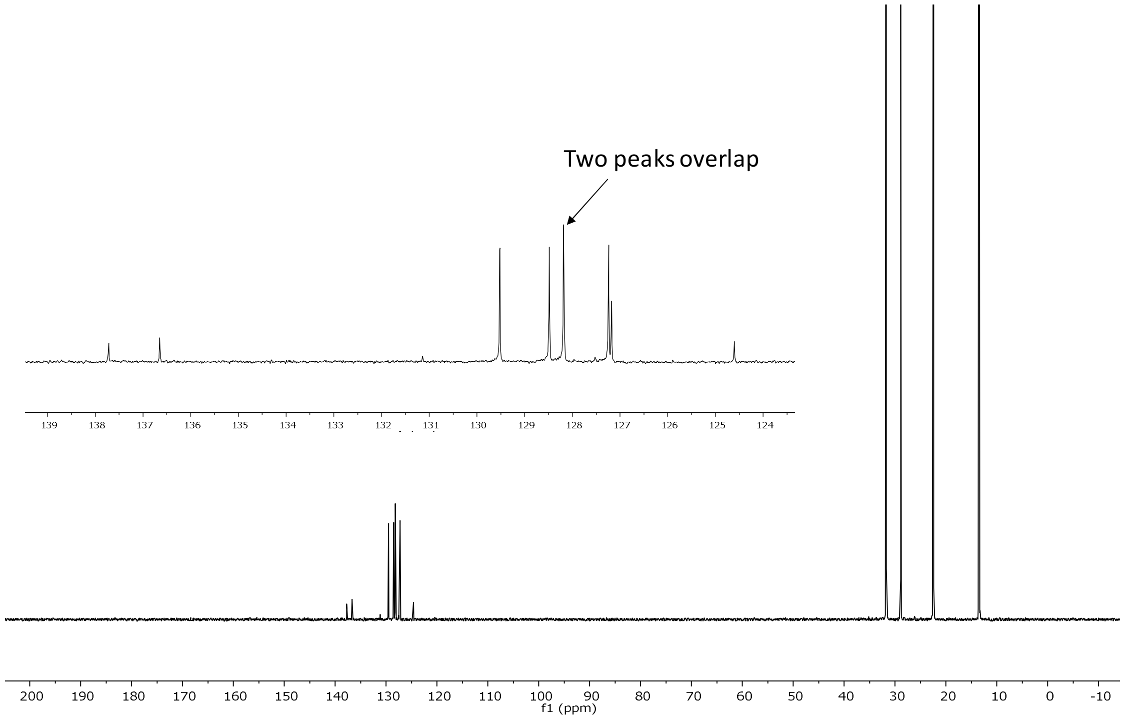

Figure S1. $^{13}C\{^1H\}$ NMR spectrum of bromoboration of diphenylacetylene with BBr_3 in heptane after 10 minutes. The spectrum suggests the full consumption of diphenylacetylene and the formation of one single product assigned as **4**.

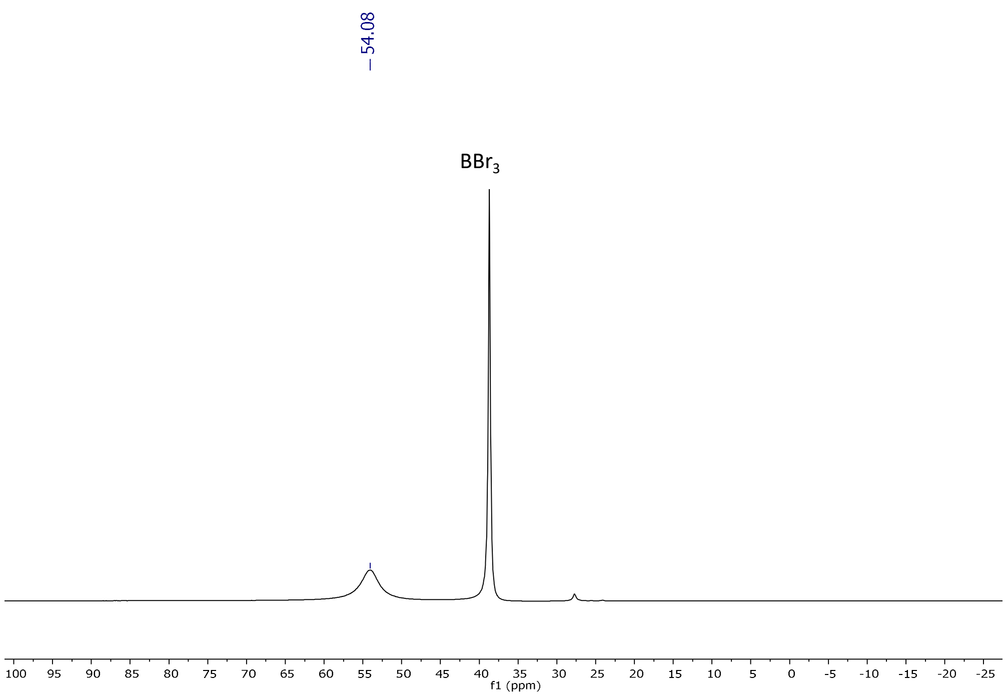

Figure S2. ^{11}B NMR spectrum of the combination of diphenylacetylene and BBr_3 in heptane after 10 minutes.

S4.1.2 NMR monitoring of the bromoboration of diphenylacetylene with BBr_3 in different solvents

(i) In hexane-heptane mixed solvents

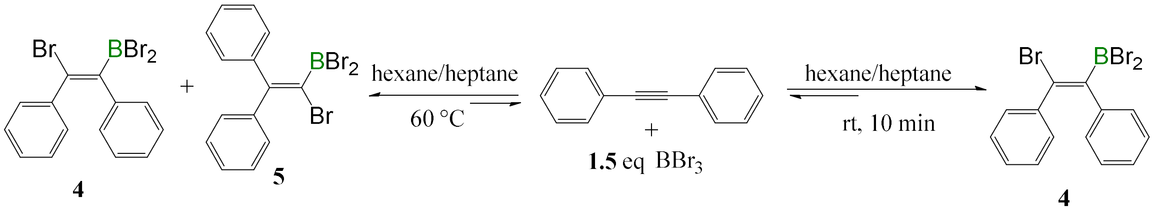

To a J-Young NMR tube charged with diphenylacetylene (36 mg, 0.2 mmol, 1 eq) was added 0.6 mL hexane to form a clear solution. Then 0.3 mL of boron tribromide solution (1 M in heptane, 0.3 mmol, 1.5 eq.) was added to the mixture which produced a light-yellow solution immediately. NMR spectroscopy after 10 minutes indicated diphenylacetylene was fully consumed and product **4** was formed cleanly based on the $^{13}C\{^1H\}$ and ^{11}B NMR spectra. On keeping the reaction mixture at room temperature for 20 hours, NMR studies indicated the formation of a minor new species. Upon heating at 60 °C $^{13}C\{^1H\}$ and ^{11}B NMR spectra show reduction of initially formed product **4** and the production of a new compound, which was assigned as 1,1-bromoboration product **5**. The reaction reached its equilibrium position after 20 hours at 60 °C. The formation of compound **4** and compound **5** was confirmed by protodeboronation reactions (See **S4.1.3**).

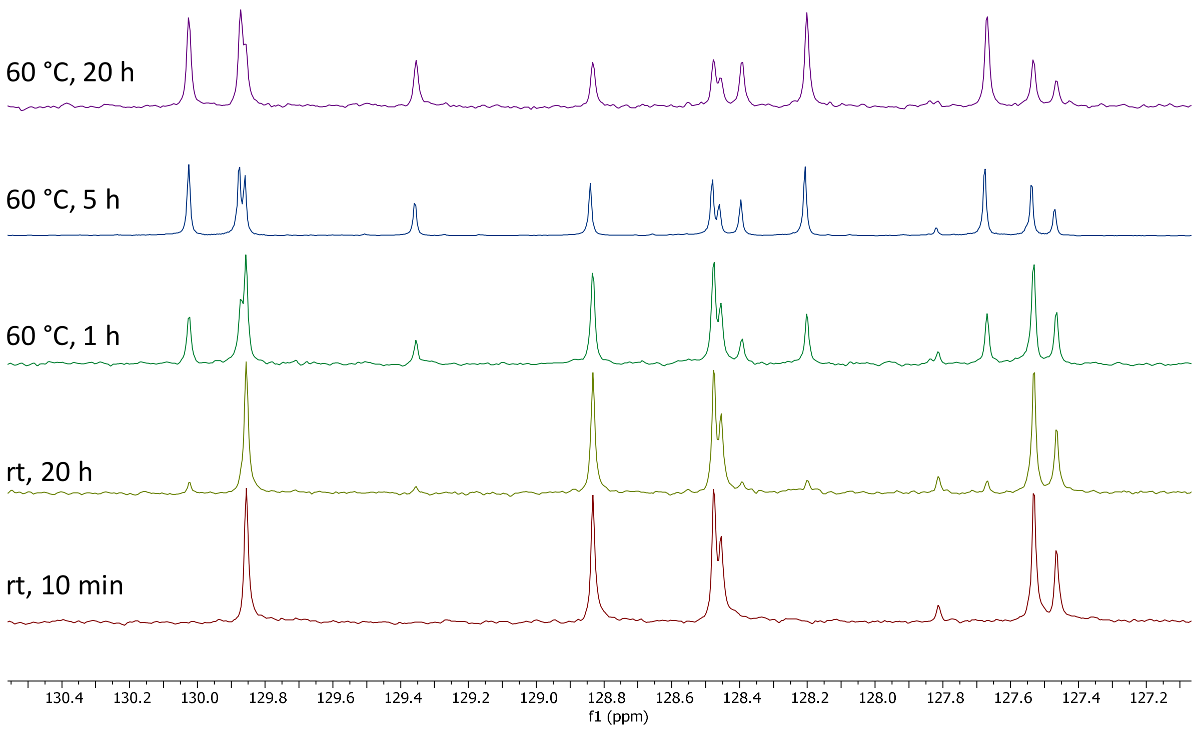

Figure S3. Stacked $^{13}C\{^{1}H\}$ NMR spectra showing the bromoboration reaction of diphenylacetylene with BBr_3 in heptane / hexane mixed solvents evolving over time.

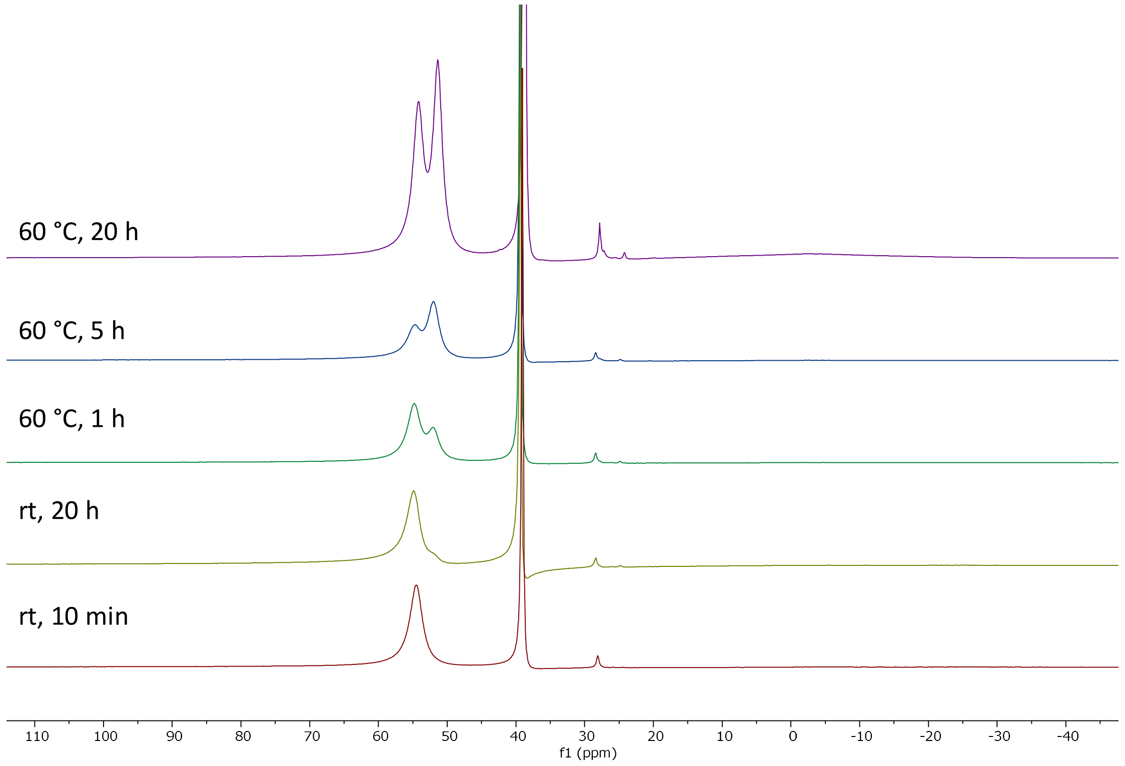

Figure S4. Stacked ^{11}B NMR spectra showing the bromoboration reaction of diphenylacetylene with BBr_3 in heptane / hexane mixed solvents evolving over time.

(ii) In dichloromethane-heptane mixed solvents

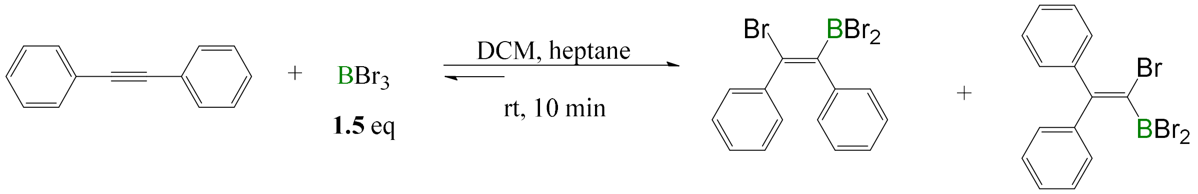

To a J-Young NMR tube charged with diphenylacetylene (36 mg, 0.2 mmol, 1 eq) was added 0.6 mL dichloromethane to form a clear solution. Then 0.3 mL of boron tribromide solution (1 M in heptane, 0.3 mmol, 1.5 eq.) was added to the mixture and a light-yellow solution was formed immediately. NMR experiments after 10 minutes indicated diphenylacetylene was fully consumed and more than one bromoboration product was formed as indicated by $^{13}C\{^1H\}$ and ^{11}B NMR spectra (Figure S5 and Figure S6). Protodeboronation of the reaction mixture indicated the formation of compound **4** and compound **5** (See **S 4.1.3**).

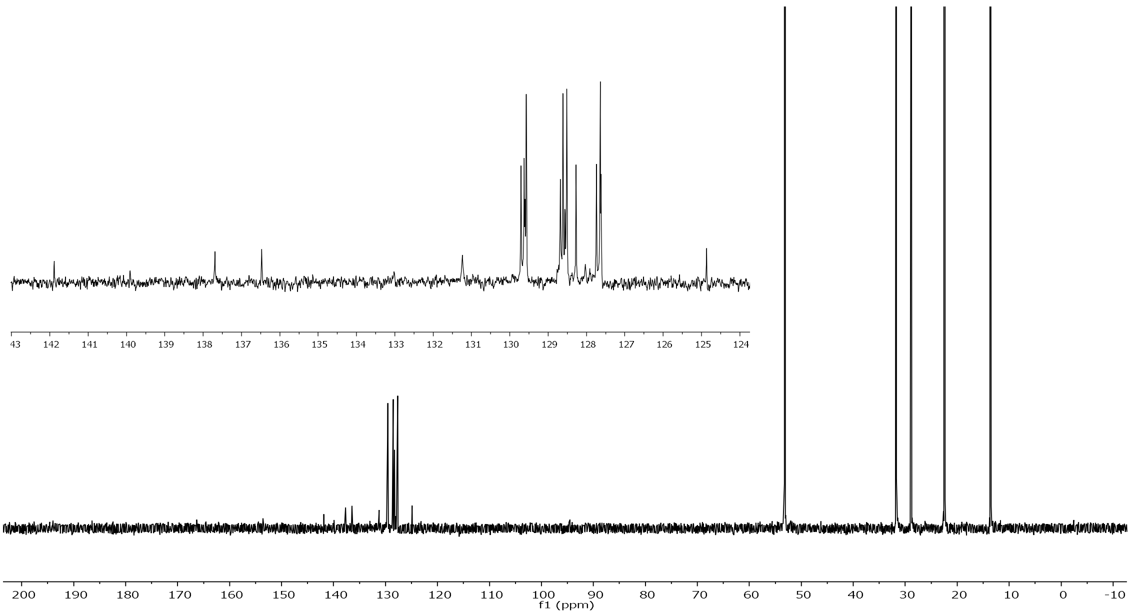

Figure S5. $^{13}C\{^1H\}$ NMR spectrum of the bromoboration of diphenylacetylene with BBr_3 in heptane / dichloromethane mixed solvents after 10 minutes. The spectrum clearly indicates the full consumption of diphenylacetylene and the formation of more than one product.

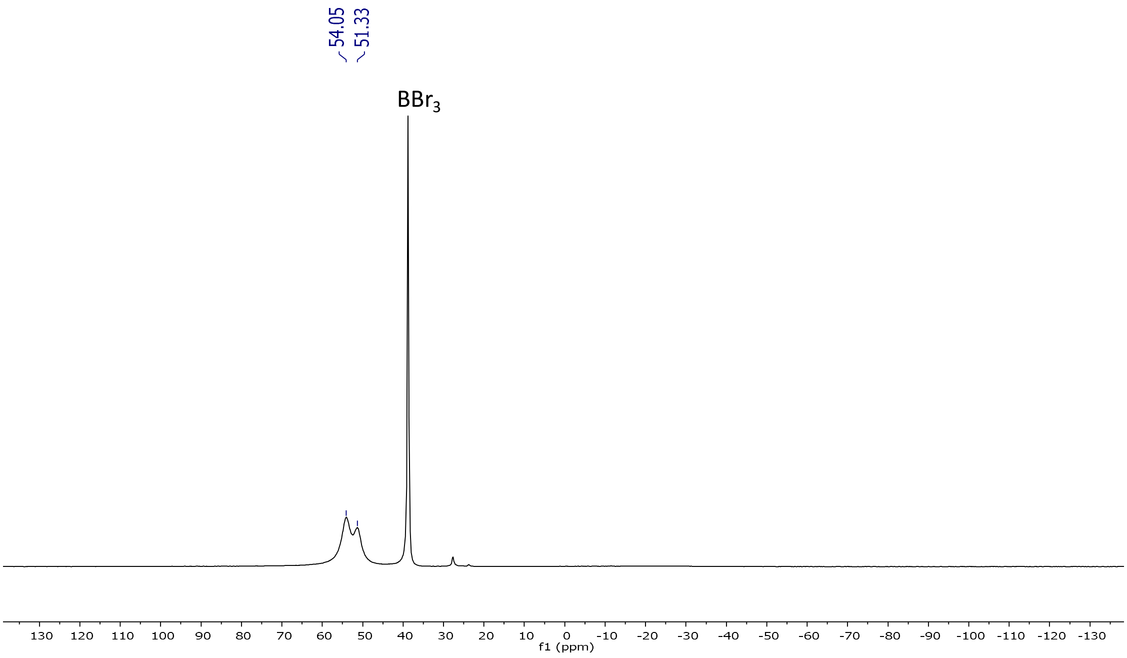

Figure S6. ^{11}B NMR spectrum of the bromoboration of diphenylacetylene with BBr_3 in heptane and dichloromethane mixed solvents after 10 minutes. The spectrum indicates the formation of more than one vinylBBr_2 product.

S 4.1.3 Bromoboration and Protodeboronation

Although NMR studies revealed that the *cis*-1,2-bromoboration reaction occurred under these reaction conditions alongside other transformations, it was difficult to confirm the exact structures of the products by NMR spectroscopy on the vinyl-BBr_2 products. As a result, we carried out protodeboronation reactions on the bromoboration products to help identify the products. It should be noted that different vinylBBr_2 isomers displayed different propensity to undergo protodeboronation, thus mixtures showing **I**, **II**, **III** along with boronic acids (from B-Br hydrolysis to B-OH) were observed. Furthermore, **II** also can be generated from addition of HBr (from B-Br hydrolysis) to the alkyne (formed on retrohaloboration). Therefore, it is not possible to provide exact ratios of haloboration products due to these challenges, but combined the information allows us to indicate the major/minor products present in-situ. The bromoboration and protodeboronation outcomes under different conditions are summarised in Table S1.

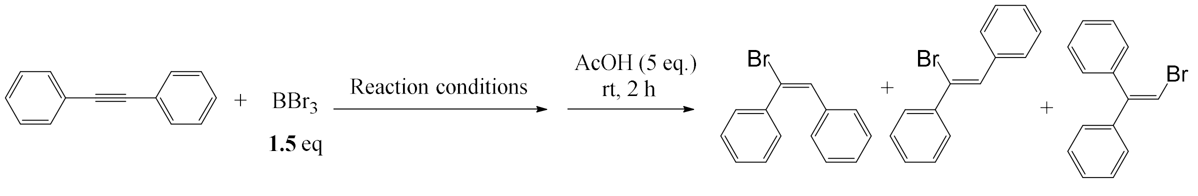

Table S1. Summary of the bromoboration/ protodeboronation of diphenylacetylene outcomes under various conditions.

Entry	BBr_3	Reaction conditions	Observed Products	
			**I** and **II**	**III**
**A**	1 M in heptane	Hexane, rt, 10 min	Major	Not observed
**B**	1 M in heptane	Hexane, rt, 20 h	Major	Minor
**C**	1 M in heptane	Hexane, 60 °C, 30 h	Minor	Major
**D**	1 M in dichloromethane	Dichloromethane, rt, 10 min	Minor	Major
**E**	1 M in dichloromethane	Dichloromethane, rt, 20 h	Minor	Major

Conditions A: To an ampoule charged with diphenylacetylene (36 mg, 0.2 mmol, 1 eq) was added 0.6 mL hexane to form a clear solution. Then 0.3 mL of boron tribromide (1 M in heptane, 0.3 mmol, 1.5 eq.) was added to the solution. The solution was kept stirring for 10 minutes, then to the reaction mixture was added acetic acid (2.5 mL, 0.4 M in hexane, 1 mmol, 5 eq.), a white precipitate was formed instantly. The mixture was stirred at room temperature for 2 hours. The protodeboronation products were separated by flash column chromatography using silica and a hexane:ethyl acetate (hexane then hexane:ethyl acetate 1:1) eluent. The ^{1}H NMR spectrum reveals the formation of *Z*-1-bromo-1,2-diphenylethene **II**, *E*-1-bromo-1,2-diphenylethene **I** and diphenylacetylene. Yield: 13 mg, 25%. We also isolated 30 mg borylated products which we were not able to determine the exact structures. However, considering that under the same bromoboration condition we only generate the 1,2-bromoboration product, we tentative assigned the product as corresponding boronic acid.

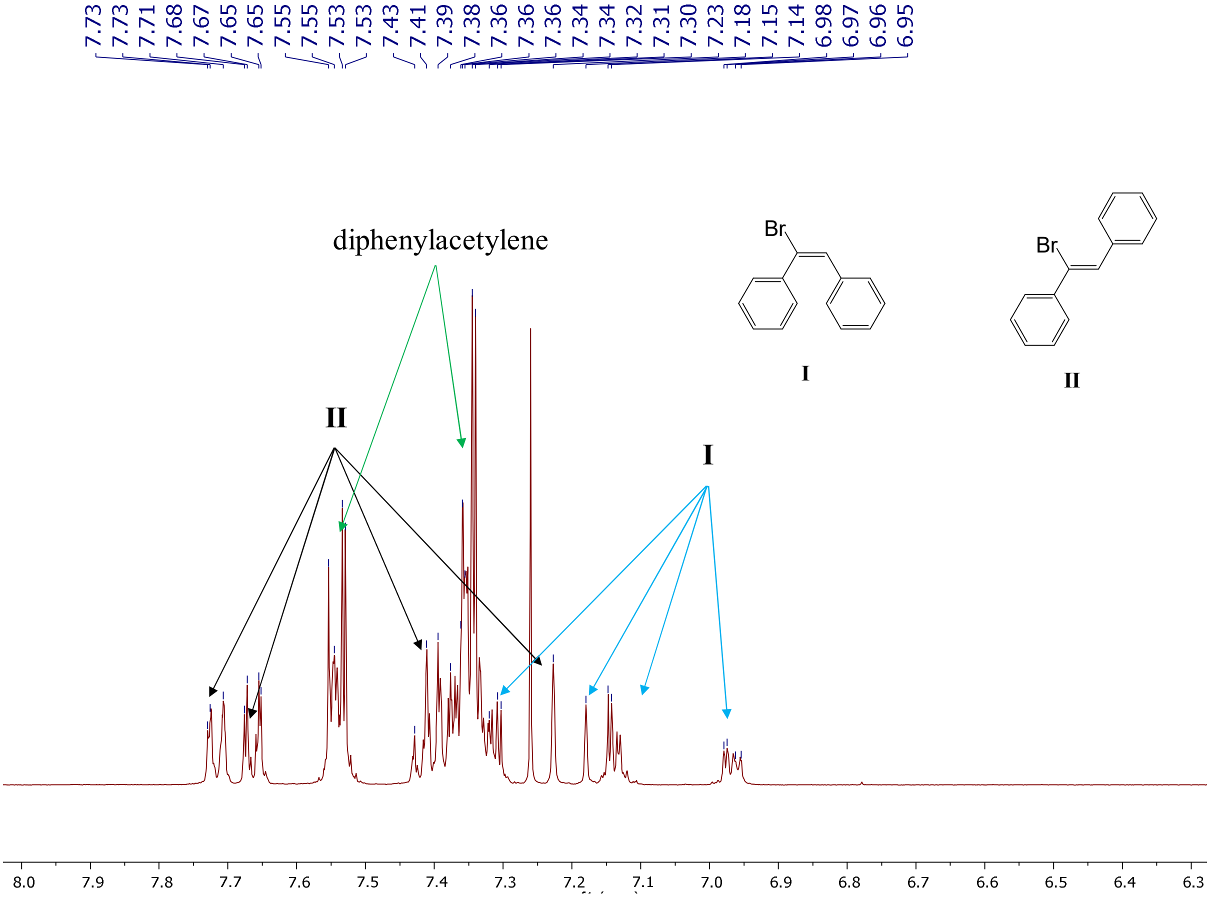

Figure S7. ^{1}H NMR spectrum showing the formation of both *Z*-1-bromo-1,2-diphenylethene **II** and *E*-1-bromo-1,2-diphenylethene **I** (Conditions A).

Conditions B: To an ampoule charged with diphenylacetylene (36 mg, 0.2 mmol, 1 eq) was added 0.6 mL hexane to form a clear solution. Then 0.3 mL of boron tribromide (1 M in heptane, 0.3 mmol, 1.5

eq.) was added to the solution. The solution was kept stirring for 20 hours, then to the reaction mixture was added acetic acid (2.5 mL, 0.4 M in hexane, 1 mmol, 5 eq.), and a white precipitate was formed instantly. The generated mixture was stirred at room temperature for 2 hours. The protodeboronation products were separated by flash column chromatography using silica and a hexane:ethyl acetate (hexane then hexane:ethyl acetate 1:1) eluent. The ^{1}H NMR spectrum reveals that aside from the formation of *Z*-1-bromo-1,2-diphenylethene **II**, *E*-1-bromo-1,2-diphenylethene **I** and diphenylacetylene, a small amount of 1-bromo-2,2-diphenylethylene **III** was also obtained. Combined yield: 9 mg, 17%. We also isolated 32 mg borylated products which we were not able to determine the exact structures.

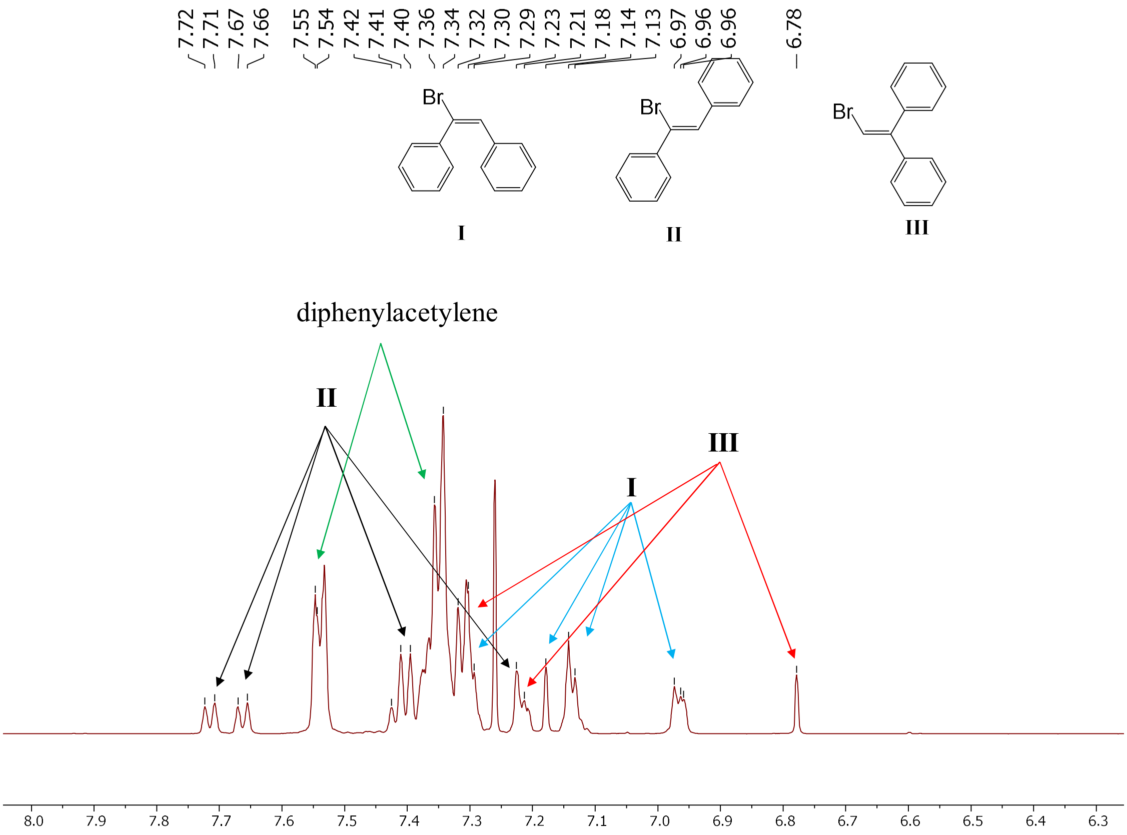

Figure S8. ^{1}H NMR spectrum showing the formation of *Z*-1-bromo-1,2-diphenylethene **II**, *E*-1-bromo-1,2-diphenylethene **I** and 1-bromo-2,2-diphenylethylene **III** (Conditions B)**.**

Conditions C: To a J-Young NMR tube charged with diphenylacetylene (36 mg, 0.2 mmol, 1 eq) was added 0.6 mL dichloromethane to form a clear solution. Then 0.3 mL of boron tribromide (1 M in dichloromethane, 0.3 mmol, 1.5 eq.) was added to the solution. The solution was heated at 60 °C for 30 hours after which no further changes were observed in the $^{13}C\{^1H\}$ and ^{11}B NMR spectra. The solution was transferred to an ampoule and to this was added acetic acid (2.5 mL, 0.4 M in hexane, 1 mmol, 5 eq.). The reaction was stirred at room temperature for 2 hours. The protodeboronation products were separated by flash column chromatography using silica and a hexane eluent. The ^1H NMR spectrum reveals the formation of 1-bromo-2,2-diphenylethylene as the major product along with small amounts of *Z*-1-bromo-1,2-diphenylethene and *E*-1-bromo-1,2-diphenylethene. Combined yield: 34 mg, 66%.

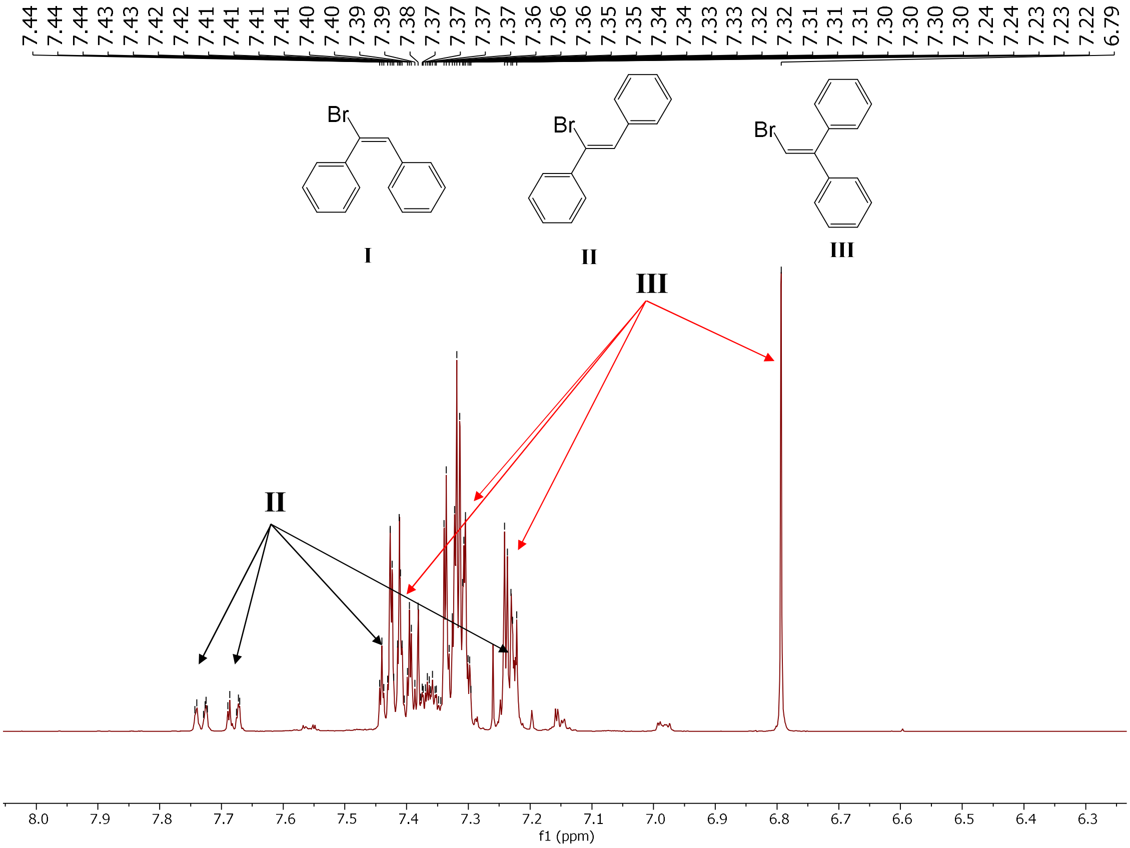

Figure S9. ^1H NMR spectrum showing the formation of 1-bromo-2,2-diphenylethylene **III** as the major product along with small amount of *Z*-1-bromo-1,2-diphenylethene **II** and *E*-1-bromo-1,2-diphenylethene **I** (Conditions C).

Conditions D: To an ampoule charged with diphenylacetylene (36 mg, 0.2 mmol, 1 eq) was added 0.6 mL dichloromethane to form a clear solution. Then 0.3 mL of boron tribromide (1 M in dichloromethane, 0.3 mmol, 1.5 eq.) was added to the solution and a light-yellow colour developed immediately. The

solution was kept stirring for 10 minutes, then to the reaction mixture was added acetic acid (2.5 mL, 0.4 M in hexane, 1 mmol, 5 eq.). The generated mixture was stirred at room temperature for 2 hours. The protodeboronation products were separated by flash column chromatography using silica and a hexane eluent. The ^{1}H NMR spectrum reveals the formation of 1-bromo-2,2-diphenylethylene **III** as the major product along with small amounts of *Z*-1-bromo-1,2-diphenylethene **I** and *E*-1-bromo-1,2-diphenylethene **III**. Combined yield: 33 mg, 64%.

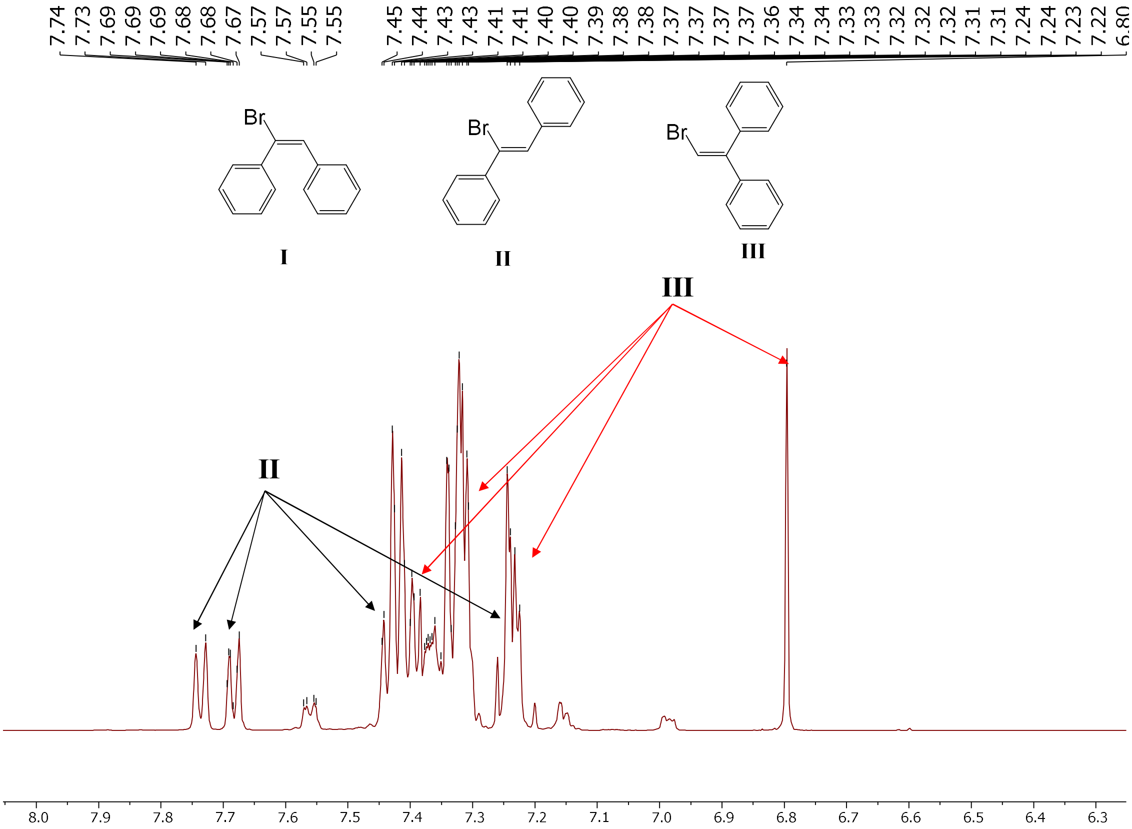

Figure S10. ^{1}H NMR spectrum showing the formation of 1-bromo-2,2-diphenylethylene **III** as the major product along with *Z*-1-bromo-1,2-diphenylethene **II** and small amount of *E*-1-bromo-1,2-diphenylethene **I** (Conditions D).

Conditions E: To an ampoule charged with diphenylacetylene (36 mg, 0.2 mmol, 1 eq) was added 0.6 mL dichloromethane to form a clear solution. Then 0.3 mL of boron tribromide (1 M in dichloromethane, 0.3 mmol, 1.5 eq.) was added to the solution and a light-yellow colour developed immediately. The solution was kept stirring for 20 hours, then to the reaction mixture was added acetic acid (2.5 mL, 0.4 M in hexane, 1 mmol, 5 eq.). The generated mixture was stirred at room temperature for 2 hours. The protodeboronation products were separated by flash column chromatography using silica and a hexane eluent. The ^{1}H NMR spectrum suggests the formation of 1-bromo-2,2-diphenylethylene as the major

product along with of *Z*-1-bromo-1,2-diphenylethene and *E*-1-bromo-1,2-diphenylethene. Combined yield: 44 mg, 85%.

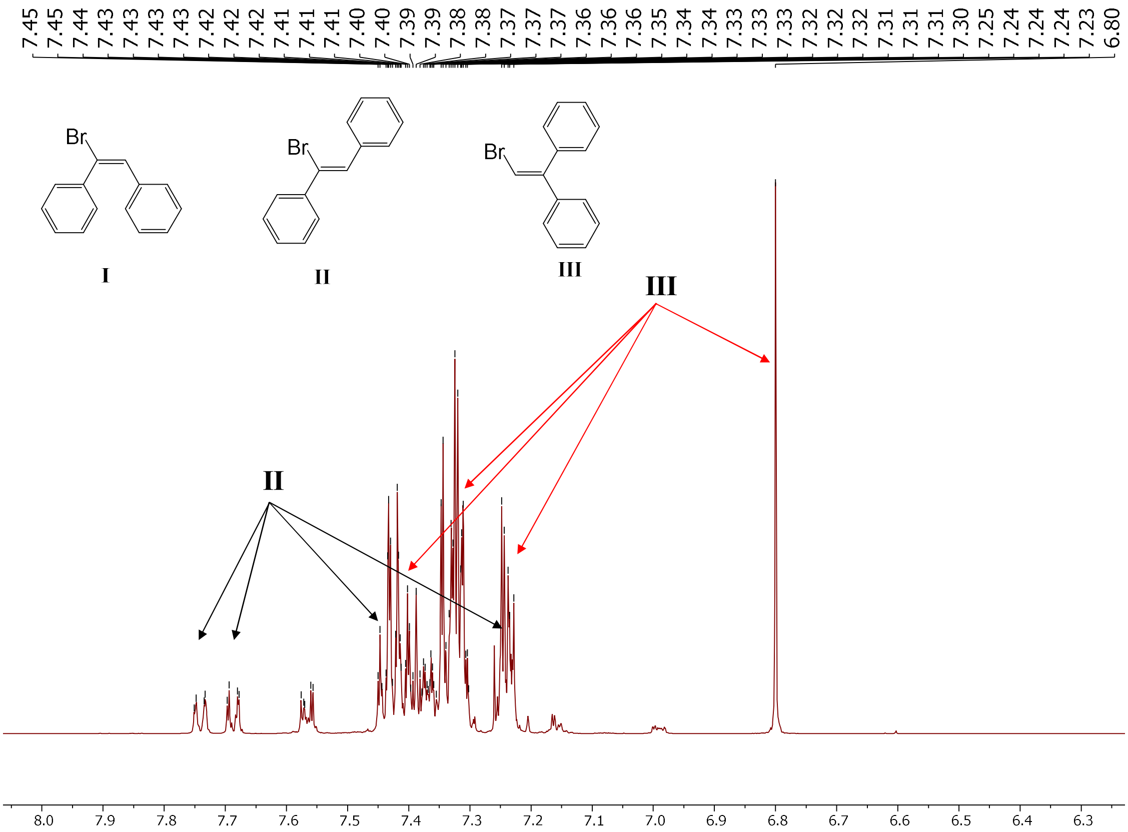

Figure S11. ^{1}H NMR spectrum showing the formation of 1-bromo-2,2-diphenylethylene **III** as the major product along with *Z*-1-bromo-1,2-diphenylethene **II** and a small amount of *E*-1-bromo-1,2-diphenylethene **I** (Conditions E).

S4.2 Bromoboration of di-*p*-tolylacetylene

Due to the lower solubility of di-*p*-tolylacetylene and particularly the corresponding bromoboration products in hexane-heptane mixed solvents, in-situ NMR tracking experiments in hexane-heptane were not conducted.

S4.2.1 Bromoboration of d di-*p*-tolylacetylene in dichloromethane-heptane mixed solvents

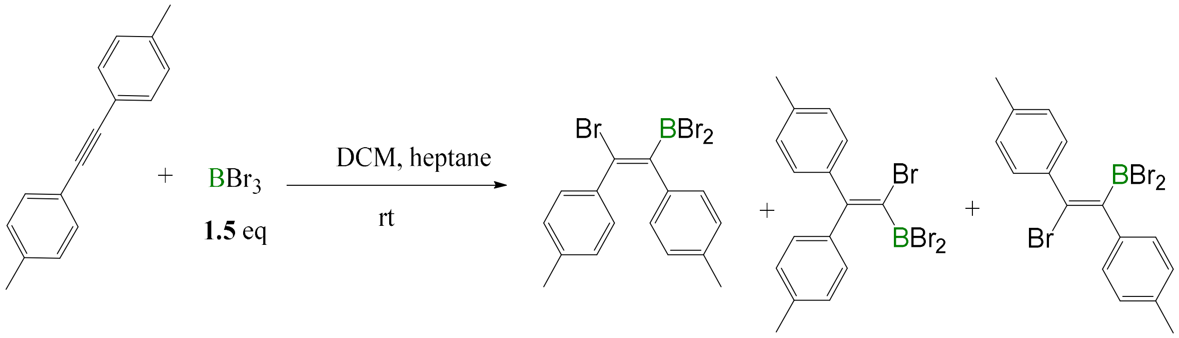

To a J-Young NMR tube charged with di-*p*-tolyl-acetylene (41 mg, 0.2 mmol, 1 eq) was added 0.6 mL dichloromethane to form a clear solution. Then 0.3 mL of boron tribromide solution (1 M in heptane, 0.3 mmol, 1.5 eq.) was added to the mixture. NMR studies right after BBr_3 addition showed a full consumption of di-*p*-tolyl-acetylene and the formation of two species in a 0.3:1 ratio based on the integration of methyl group resonances. Post-protodeboronation at this stage indicated the formation of both the 1,2-*cis*-bromoboration product and the 1,1-bromoboration product (See **S4.2.3**). Upon standing at room temperature for 60 hours, a new compound was formed with a boron resonance being similar to the 1,2-*cis*-bromoboration product (See Figure S13). Thus, the new compound was tentatively assigned as 1,2-*trans*-bromoboration product. We cannot confirm the formation of 1,2-*trans*-bromoboration product via protodeboronation reaction as *E*/*Z*-1-bromo-1,2-bis(*p*-tolyl)ethene were formed all the time under the reaction conditions (See **S4.2.3**), possibly due to the reaction of HBr with free alkyne (formed by retro-haloboration).

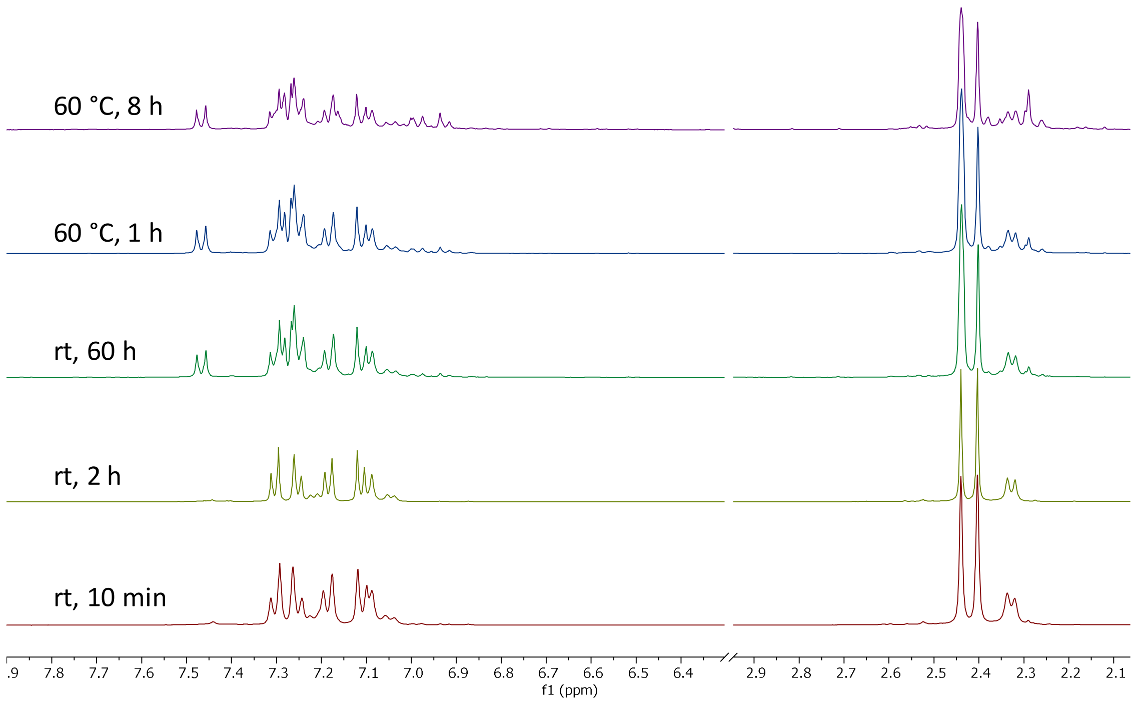

Figure S12. Stacked ^{1}H NMR spectra of the bromoboration of di-*p*-tolylacetylene with BBr_3 in dichloromethane-heptane mixed solvents.

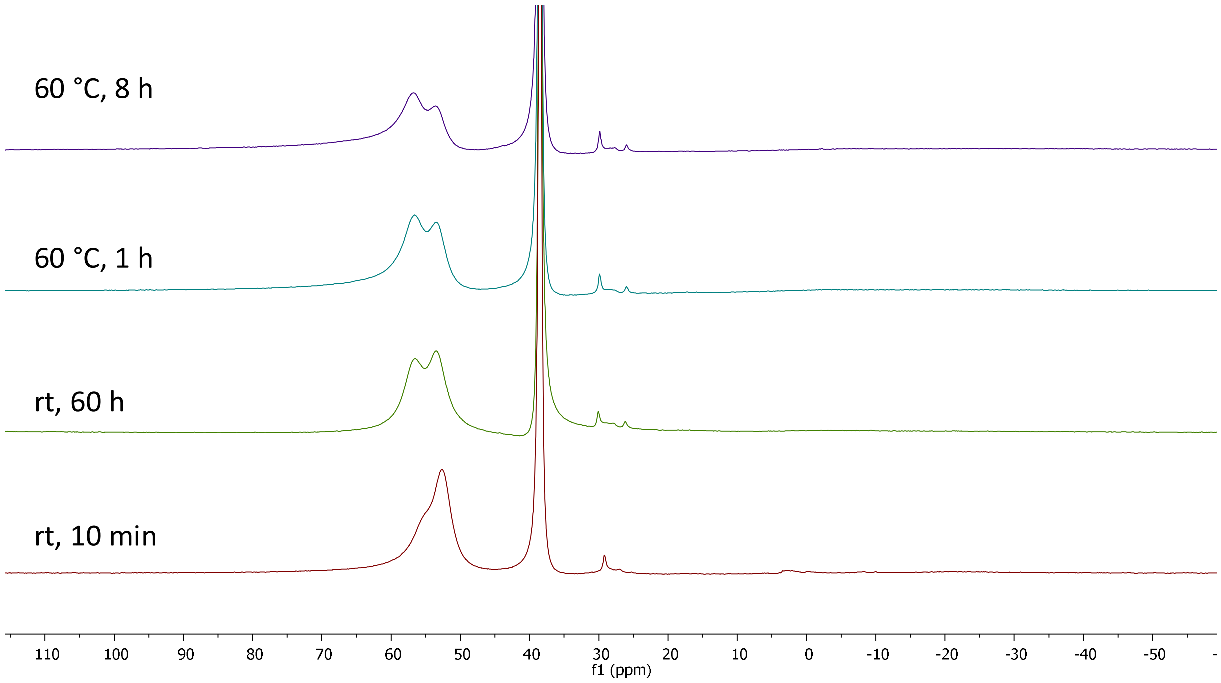

Figure S13. Stacked ^{11}B NMR spectra of bromoboration of di-*p*-tolylacetylene with BBr_3 in heptane / hexane mixed solvents.

S4.2.2 Variable Temperaure NMR studies of the equilibrium between 1,2- and 1,1- bromoboration

To determine if varying the temperature can lead to a significant difference in product distribution VT NMR studies were performed. A 1:0.3 mixture of 1,1- and 1,2-bromoboration products were prepared in a procedure similar to that described in S4.4.1 except that BBr_3 was dissolved in dichloromethane. The ratio of the two products was estimated from the integrations of the methyl group resonances. At 28 °C, a 1:0.3 ratio was obtained. The NMR sample was then gradually cooled to -40 °C over 30 minutes, integration of the methyl group resonances gave a ratio of 1 : 0.25. Upon warming up to room temperature, a 1:0.29 mixture was formed again.

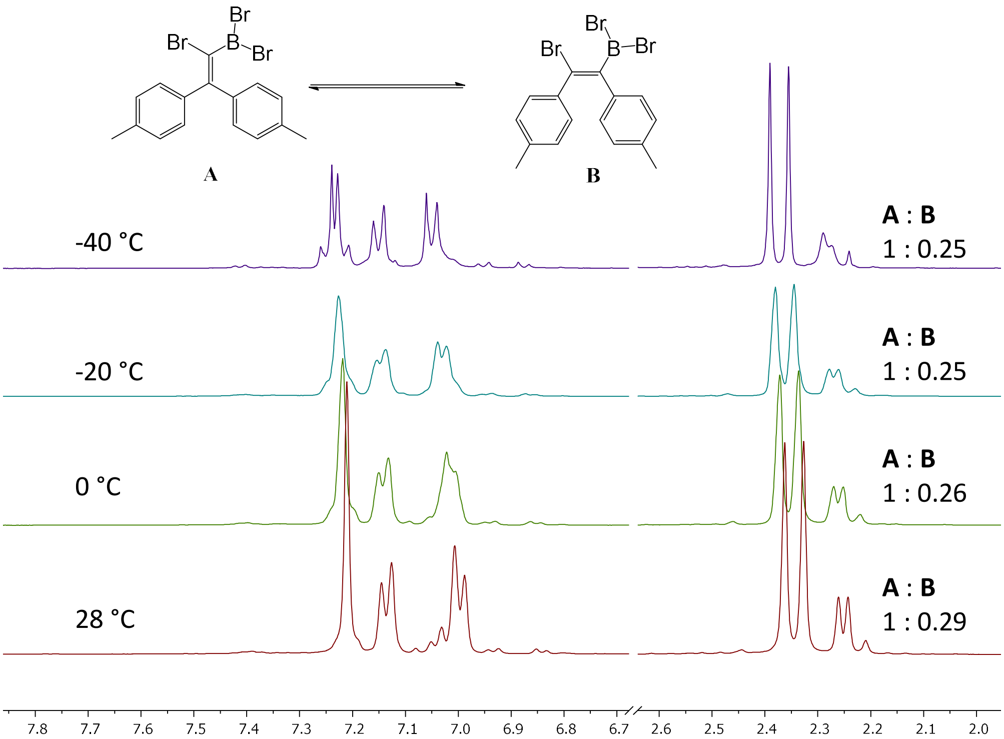

Figure S14. Stacked ^{1}H NMR spectra showing the relative ratio of 1,2- and 1,1- bromoboration products in DCM at different temperatures.

4.2.3 Bromoboration and Protodeboronation

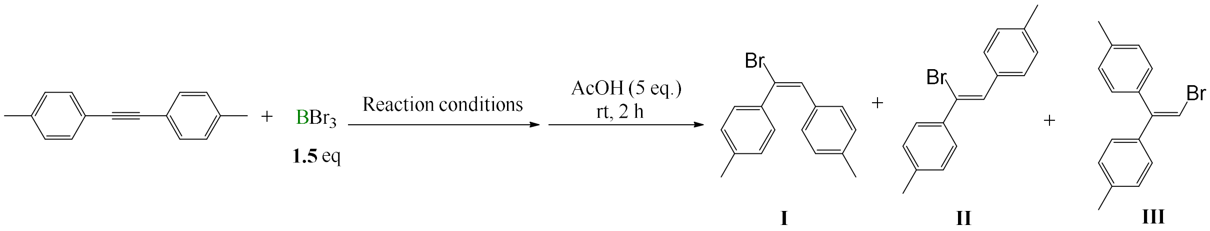

Table S2. Summary of the bromoboration/ protodeboronation of di-*p*-tolylacetylene outcomes under various conditions.

Entry	BBr_3	Reaction conditions	Products	
			**I** and **II**	**III**
**A**	1 M in heptane	Hexane, rt, 10 min	Major	Minor
**B**	1 M in heptane	Hexane, rt, 20 h	Minor	Major
**C**	1 M in dichloromethane	Dichloromethane, rt, 10 min	Minor	Major
**D**	1 M in dichloromethane	Dichloromethane, rt, 20 h	Minor	Major

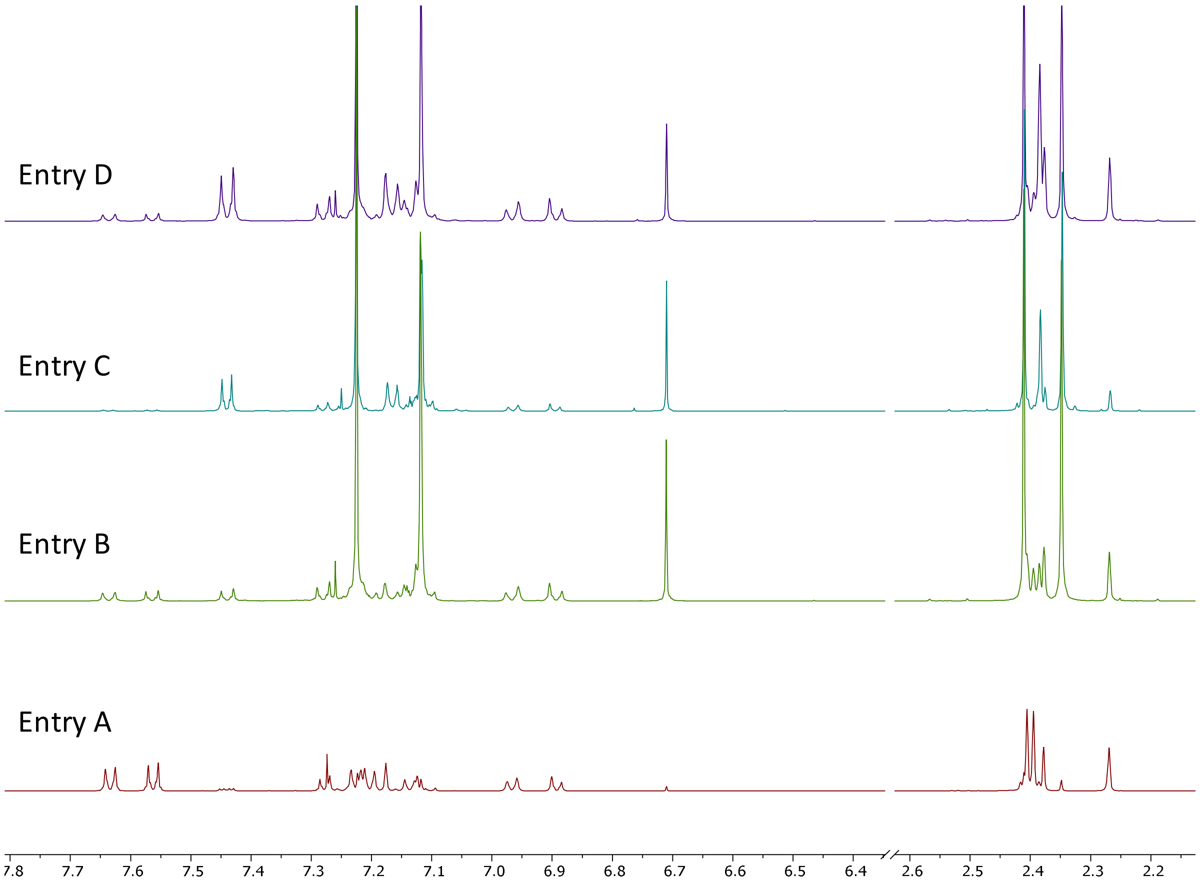

Figure S15. Stacked ^1H NMR spectra showing the outcomes of bromoboration with BBr_3 - protodeboronation of di-*p*-tolyl-acetylene under different conditions.

Conditions A: To an ampoule charged with di-*p*-tolyl-acetylene (41 mg, 0.2 mmol, 1 eq) was added 0.6 mL hexane to form a suspension. Then 0.3 mL of boron tribromide (1 M in heptane, 0.3 mmol, 1.5 eq.) was added and a dark brown suspension developed immediately. The solution was kept stirring for 10 minutes, then to the reaction mixture was added acetic acid (2.5 mL, 0.4 M in hexane, 1 mmol, 5 eq.), and a white precipitate was formed instantly. The mixture was stirred at room temperature for 2 hours. The protodeboronation products were separated by flash column chromatography using silica and a hexane eluent. Combined yield: 16 mg, 29%.

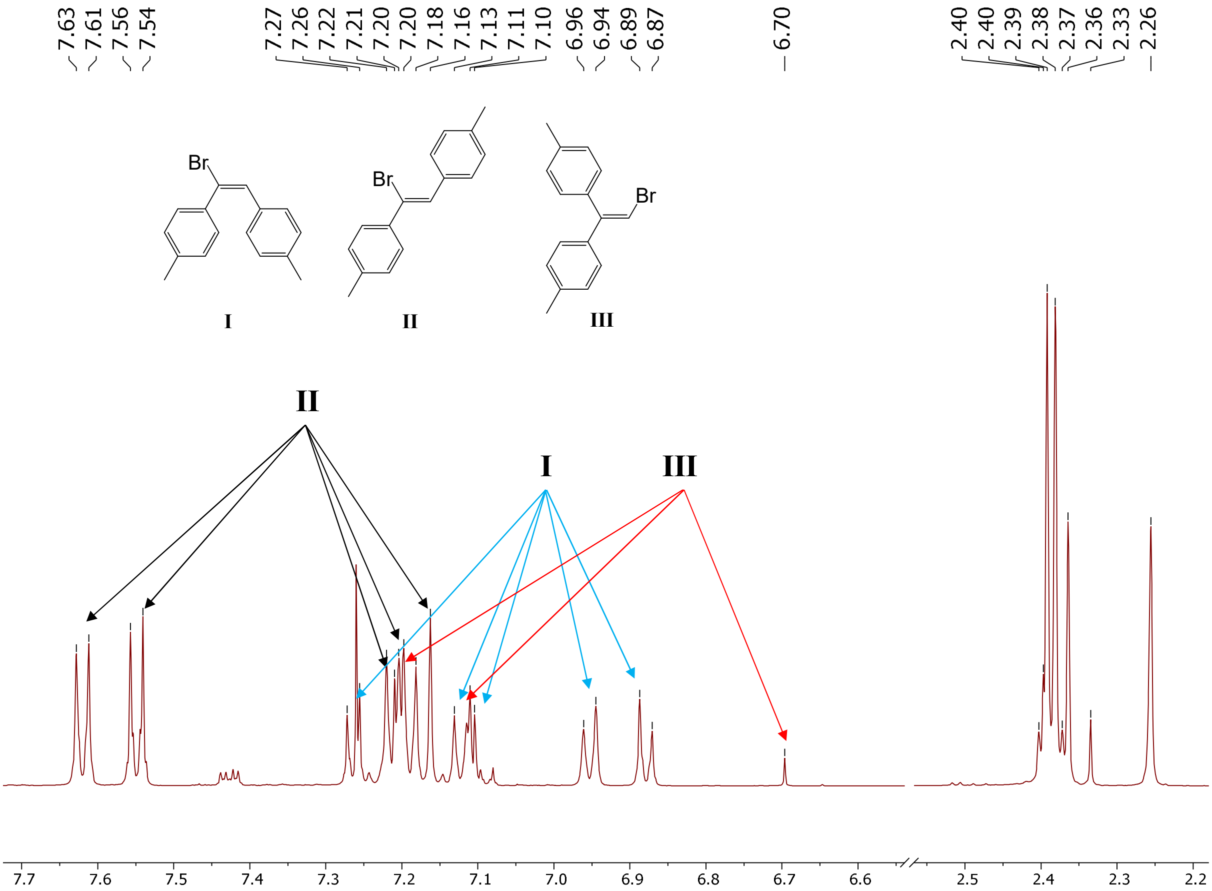

Figure S16. ^{1}H NMR spectrum showing the formation of *E*/*Z*-1-bromo-1,2-bis(*p*-tolyl)ethene (**I**, **II**) as the major product along with 1-bromo-2,2-bis(*p*-tolyl)ethylene (**III**) (Conditions A).

Conditions B: To an ampoule charged with di-*p*-tolyl-acetylene (41 mg, 0.2 mmol, 1 eq) was added 0.6 mL hexane to form a suspension. Then 0.3 mL of boron tribromide (1 M in heptane, 0.3 mmol, 1.5 eq.) was added and a dark brown suspension developed immediately. The solution was kept stirring for 20 hours, then to the reaction mixture was added acetic acid (2.5 mL, 0.4 M in hexane, 1 mmol, 5 eq.), and a white precipitate was formed instantly. The mixture was stirred at room temperature for 2 hours. The protodeboronation products were separated by flash column chromatography using silica and a hexane eluent. Combined yield: 42 mg, 73%.

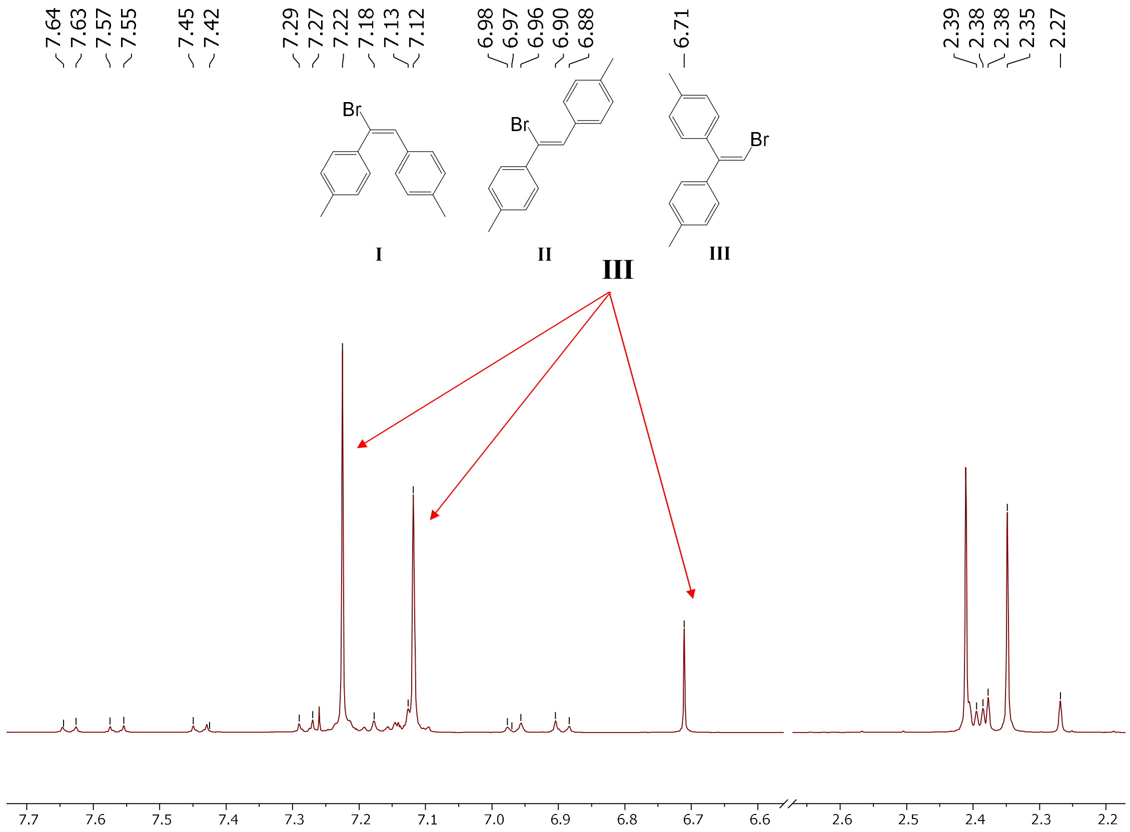

Figure S17. ^{1}H NMR spectrum showing the formation of 1-bromo-2,2-bis(*p*-tolyl)ethylene (**III**) as the major product (Condition B).

Conditions C: To an ampoule charged with di-*p*-tolyl-acetylene (41 mg, 0.2 mmol, 1 eq) was added 0.6 mL dichloromethane to form a homogenous solution. Then 0.3 mL of boron tribromide (1 M in dichloromethane, 0.3 mmol, 1.5 eq.) was added and a dark solution developed immediately. The solution was kept stirring for 10 min, then to the reaction mixture was added acetic acid (2.5 mL, 0.4 M in hexane, 1 mmol, 5 eq). The mixture was stirred at room temperature for 2 hours. The protodeboronation products were separated by flash column chromatography using silica and a hexane eluent. Combined yield: 42 mg, 73%.

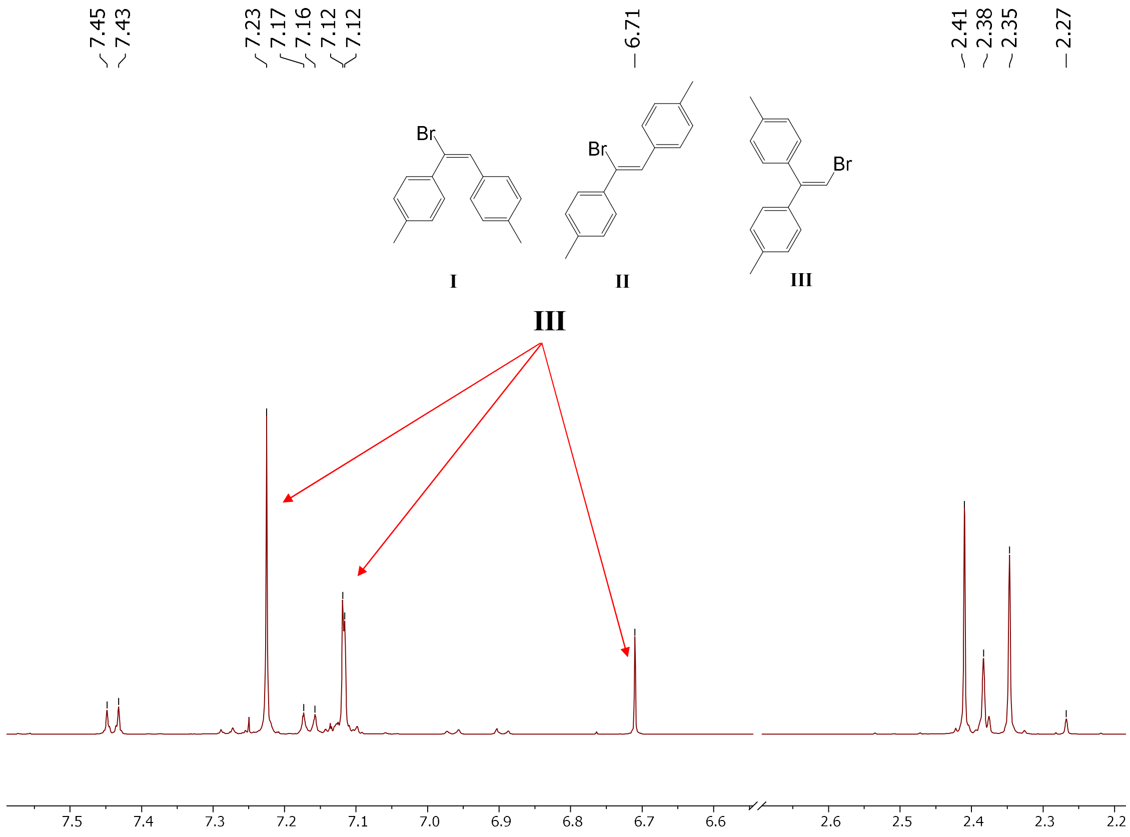

Figure S18. ^{1}H NMR spectrum showing the formation of 1-bromo-2,2-bis(*p*-tolyl)ethylene (**III**) as the major product along with a small amount of di-*p*-tolylacetylene (Conditions C).

D: To an ampoule charged with di-*p*-tolyl-acetylene (41 mg, 0.2 mmol, 1 eq) was added 0.6 mL dichloromethane to form a homogenous solution. Then 0.3 mL of boron tribromide (1 M in dichloromethane, 0.3 mmol, 1.5 eq.) was added and a dark solution developed immediately. The solution was kept stirring for 20 hours, then to the reaction mixture was added acetic acid (2.5 mL, 0.4 M in hexane, 1 mmol, 5 eq). The mixture was stirred at room temperature for 2 hours. The protodeboronation products were separated by flash column chromatography using silica and a hexane eluent. Combined yield: 33 mg, 57%.

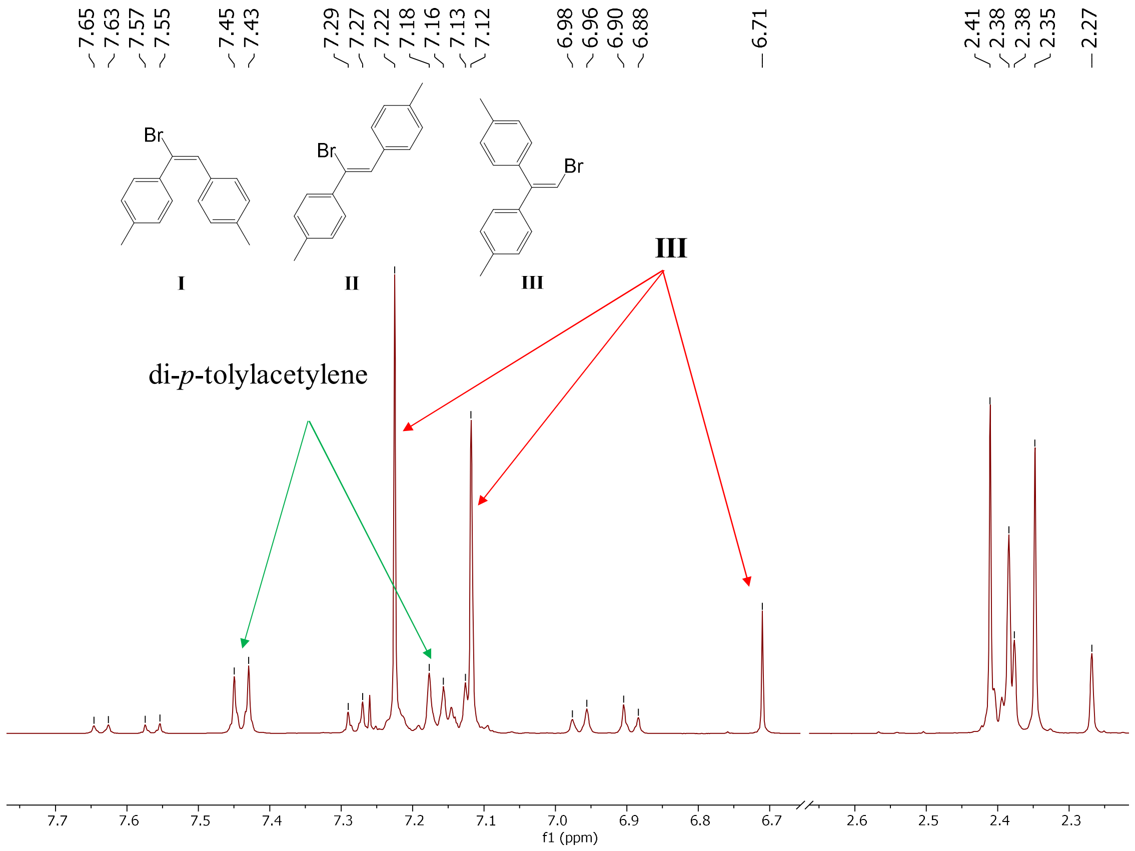

Figure S19. ^{1}H NMR spectrum showing the formation of 1-bromo-2,2-bis(*p*-tolyl)ethylene (**III**) as the major product along with some di-*p*-tolylacetylene (Conditions D).

S4.3 Bromoboration of 1,2-bis(4-fluorophenyl)ethyne

<u>**S4.3.1** NMR monitoring of bromoboration of 1,2-bis(4-fluorophenyl)ethyne</u>

(i) In hexane-heptane mixed solvents

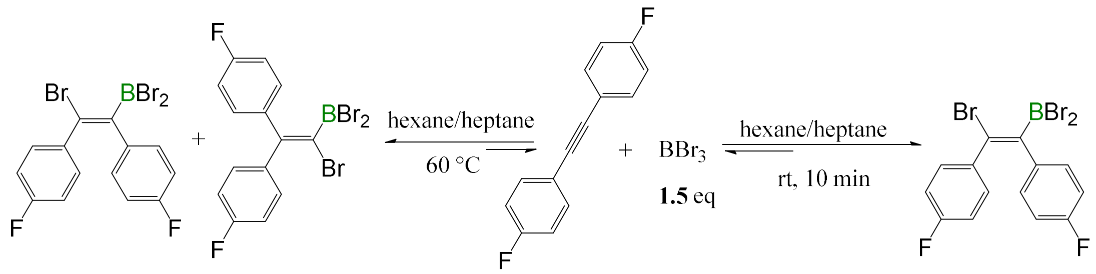

To a J-Young NMR tube charged with 1,2-bis(4-fluorophenyl)ethyne (43 mg, 0.2 mmol, 1 eq) was added 0.6 mL hexane to form a suspension. Then 0.3 mL of boron tribromide (1 M in heptane, 0.3 mmol, 1.5 eq.) was added to the solution and upon shaking a clear solution was obtained instantly. NMR spectroscopy after 10 minutes indicated the 1,2-bis(4-fluorophenyl)ethyne was fully consumed and one major product along with some minor products were observed in the ^{1}H and ^{11}B NMR spectra. Keeping the reaction mixture at room temperature for 20 hours, the NMR spectra showed almost no change. In order to accelerate the transformation, the reaction was heated at 60 °C.

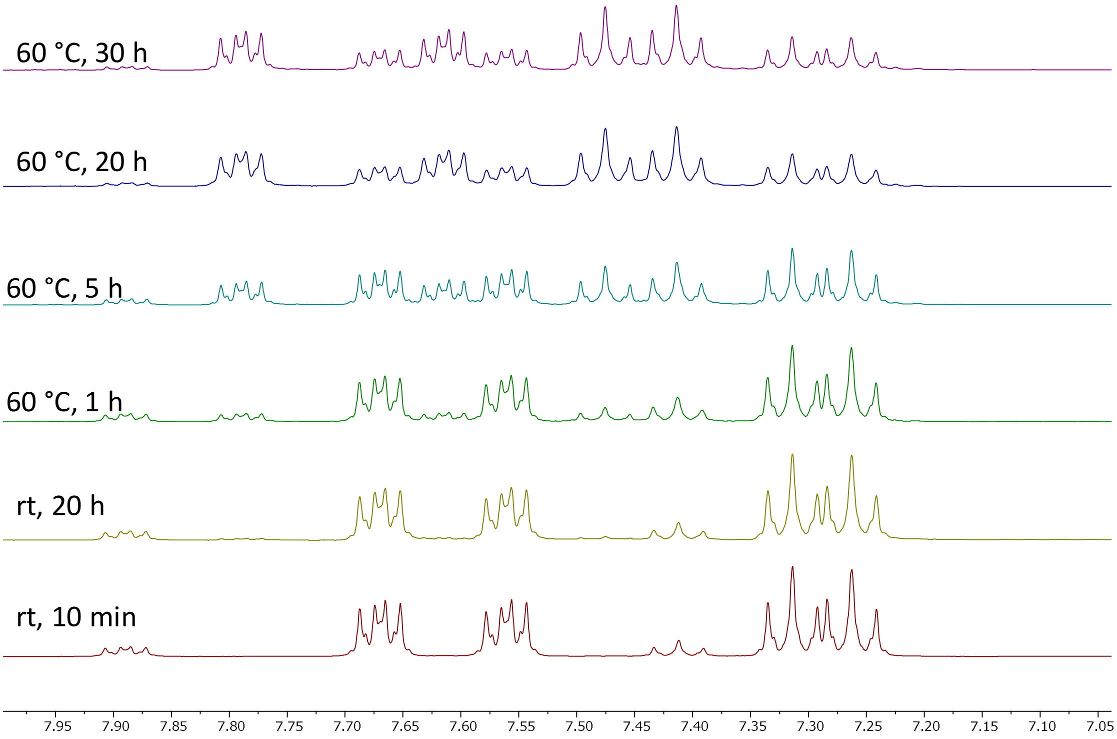

Figure S20. Stacked ^{1}H NMR spectra of the bromoboration of 1,2-bis(4-fluorophenyl)ethyne with BBr_3 in heptane and hexane mixed solvents.

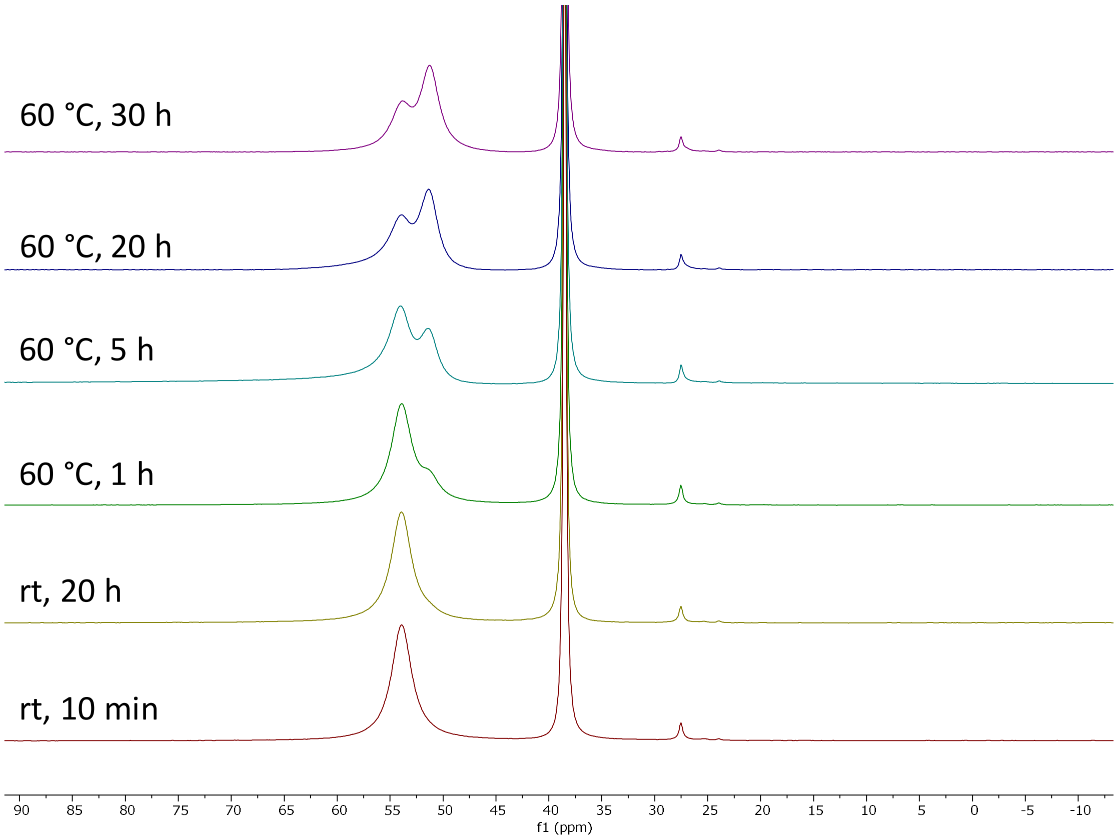

Figure S21. Stacked ^{11}B NMR spectra of the bromoboration of 1,2-bis(4-fluorophenyl)ethyne with BBr_3 in heptane and hexane mixed solvents.

(ii) In dichloromethane

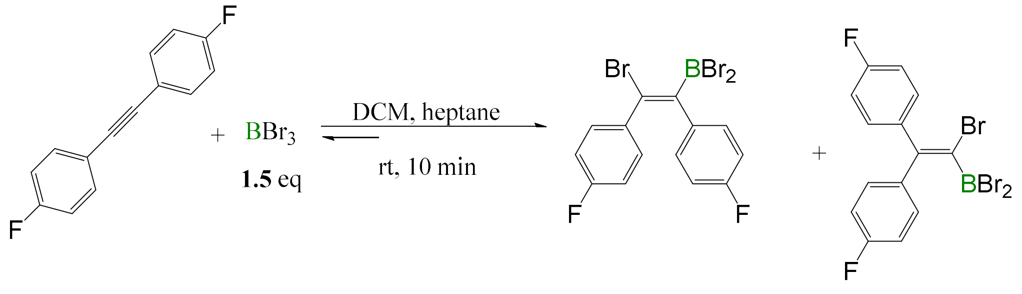

To a J-Young NMR tube charged with 1,2-bis(4-fluorophenyl)ethyne (43 mg, 0.2 mmol, 1 eq) was added 0.6 mL dichloromethane to form a homogenous solution. Then 0.3 mL of boron tribromide (1 M in dichloromethane, 0.3 mmol, 1.5 eq.) was added to the solution. NMR spectroscopy after 10 minutes indicated 1,2-bis(4-fluorophenyl)ethyne was fully consumed and two products were formed in a 1:1 ratio. Keeping the reaction mixture at room temperature for 2 hours, the ratio changed to 3:1. On keeping the reaction mixture at room temperature for 20 hours no further change was observed.

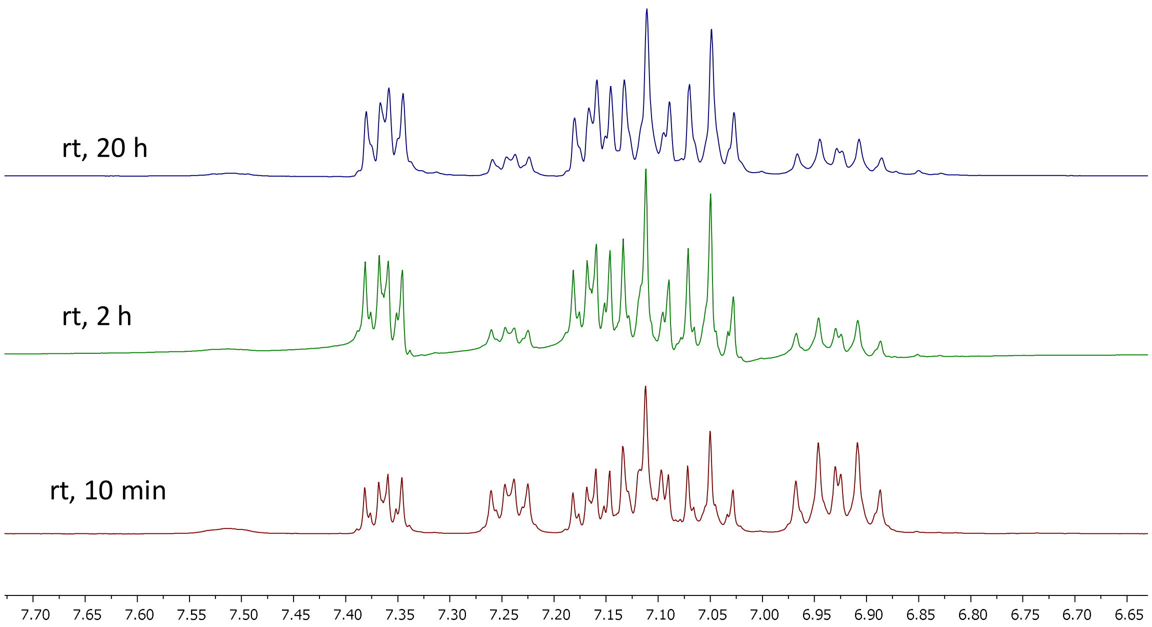

Figure S22. Stacked ^{1}H NMR spectra of the bromoboration of 1,2-bis(4-fluorophenyl)ethyne with BBr_3 in dichloromethane.

S4.3.2 Bromoboration and Protodeboronation

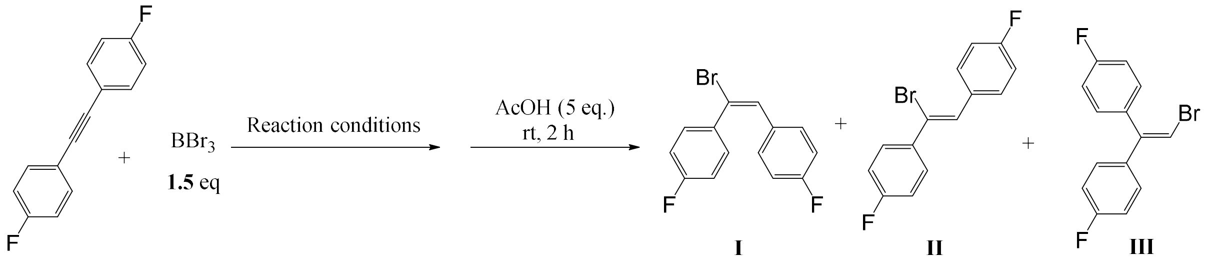

Table S3. Summary of the bromoboration/ protodeboronation of 1,2-bis(4-fluorophenyl)ethyne outcomes under various conditions.[a]

Entry	BBr_3	Reaction conditions	Products	
			**I and II**	**III**
**A**	1 M in heptane	Hexane, rt, 10 min	-	Not observed
**B**	1 M in heptane	Hexane, 60 °C, 30 h	-	Major
**C**	1 M in dichloromethane	Dichloromethane, rt, 10 min	-	Major

[a]Compounds **I** and **II** are not reported in literature.

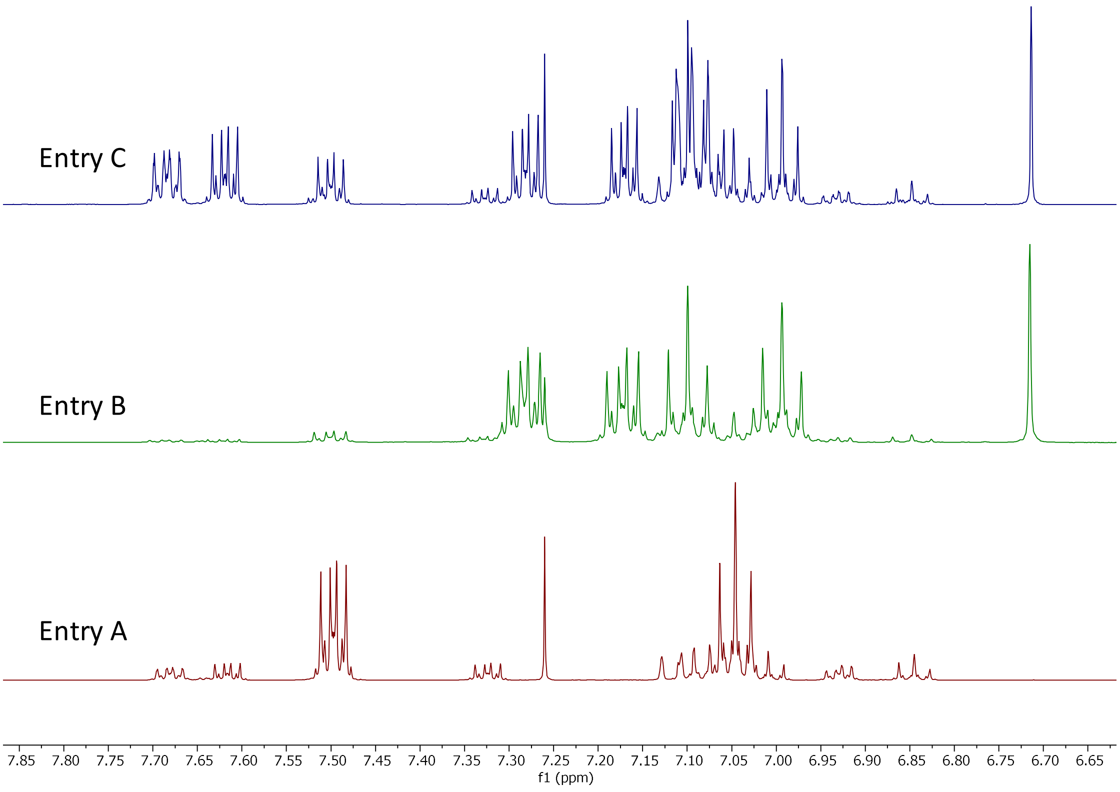

Figure S23. Stacked ^{1}H NMR spectra showing the outcomes of bromoboration-protodeboronation of 1,2-bis(4-fluorophenyl)ethyne with BBr_3 under different conditions.

Conditions A: To an ampoule charged with 1,2-bis(4-fluorophenyl)ethyne (43 mg, 0.2 mmol, 1 eq) was added 0.6 mL hexane to form a suspension. Then 0.3 mL of boron tribromide (1 M in heptane, 0.3 mmol, 1.5 eq.) was added and a clear solution was obtained instantly. The solution was kept stirring for 10 minutes, then to the reaction mixture was added acetic acid (2.5 mL, 0.4 M in hexane, 1 mmol, 5 eq.), and a white precipitate was formed instantly. The mixture was stirred at room temperature for 2 hours. The protodeboronation products were separated by flash column chromatography using silica and hexane: ethyl acetate (hexane then hexane: ethyl acetate 1:1). The ^{1}H NMR spectrum suggests the mixture mainly consists of 1,2-bis(4-fluorophenyl)ethyne while 1-bromo-2,2-bis(*p*-fluorophenyl)ethylene was not formed under these conditions.

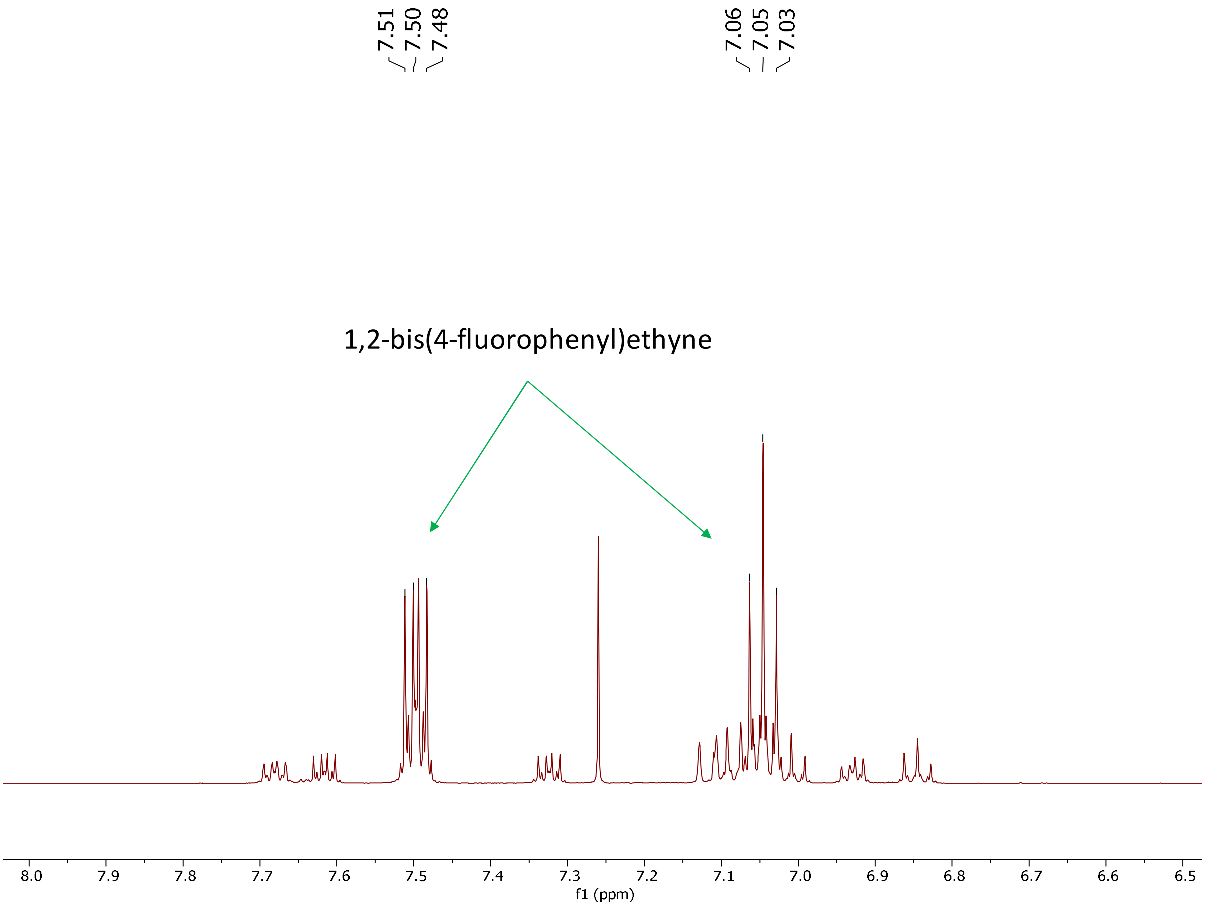

Figure S24. ^{1}H NMR spectrum showing the bromoboration-protodeboronation under conditions **A** afforded 1,2-bis(4-fluorophenyl)ethyne, no 1-bromo-2,2-bis(*p*-fluorophenyl)ethylene was observed.

Conditions B: To a J-Young NMR tube charged with 1,2-bis(4-fluorophenyl)ethyne (43 mg, 0.2 mmol, 1 eq) was added 0.6 mL hexane to form a suspension. Then 0.3 mL of boron tribromide (1 M in heptane, 0.3 mmol, 1.5 eq.) was added and upon shaking a clear solution was obtained instantly. The solution was heated at 60 °C for 30 hours until no further changes were observed in the ^{1}H and ^{11}B NMR spectra. The obtained solution was transferred to an ampoule to which was added acetic acid (2.5 mL, 0.4 M in hexane, 1 mmol, 5 eq.). The reaction was stirred at room temperature for 2 hours. The protodeboronation products were separated by flash column chromatography using silica and a hexane eluent. The ^{1}H NMR spectrum indicated the formation of 1-bromo-2,2-bis(*p*-fluorophenyl)ethylene as the major product. Yield: 27 mg, 46%.

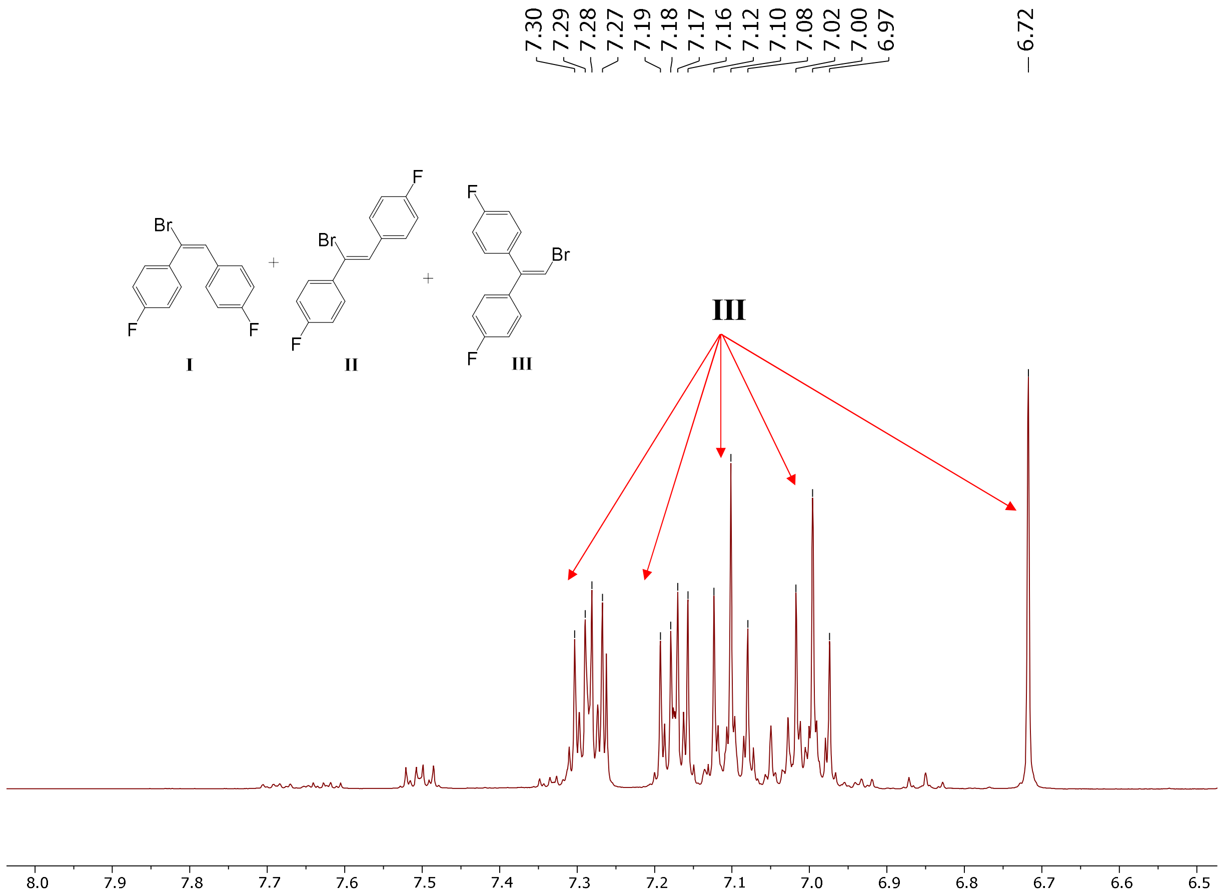

Figure S25. ^{1}H NMR spectrum showing the bromoboration-protodeboronation under conditions **B** afforded 1-bromo-2,2-bis(*p*-fluorophenyl)ethylene as the major product.

Conditions C: To an ampoule charged with 1,2-bis(4-fluorophenyl)ethyne (43 mg, 0.2 mmol, 1 eq) was added 0.6 mL hexane. Then 0.3 mL of boron tribromide (1 M in dichloromethane, 0.3 mmol, 1.5 eq.) was added and a clear solution was formed immediately. The solution was kept stirring for 10 min, then to the reaction mixture was added acetic acid (2.5 mL, 0.4 M in hexane, 1 mmol, 5 eq). The mixture was stirred at room temperature for 2 hours. The protodeboronation products were separated as a mixture by flash column chromatography using silica and a hexane eluent. The ^{1}H NMR spectrum indicated the formation of 1-bromo-2,2-bis(*p*-fluorophenyl)ethylene as the major product. Yield: 27 mg, 46%.

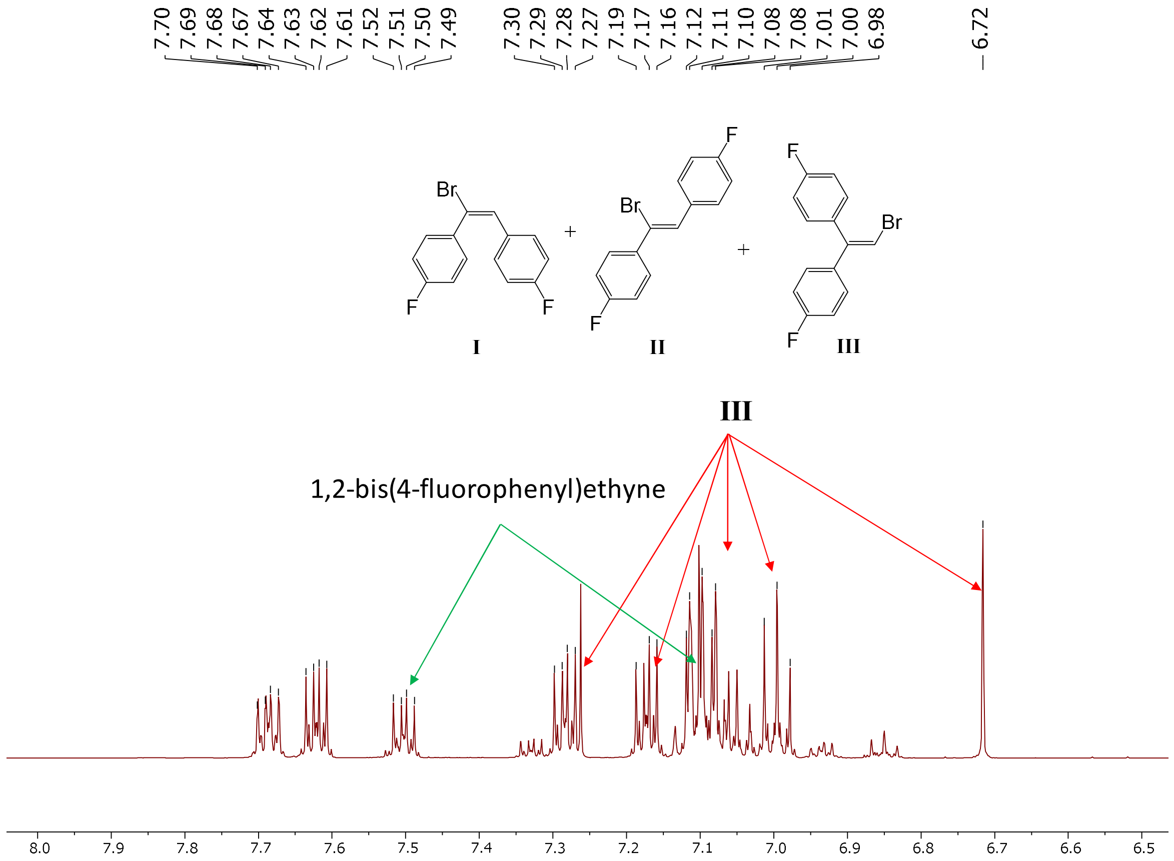

Figure S26. ^{1}H NMR spectrum showing the bromoboration-protodeboronation under conditions **C** afforded a complex mixture containing 1-bromo-2,2-bis(*p*-fluorophenyl)ethylene.

S4.4 Bromoboration of 1-phenyl-1-propyne

<u>**S4.4.1** NMR monitoring of the bromoboration of 1-phenyl-1-propyne</u>

To a J-Young NMR tube was added 1-phenyl-1-propyne (25 µL, 0.2 mmol, 1 eq) and 0.6 mL dichloromethane. Then 0.3 mL of boron tribromide solution (0.3 mL, 1 M in dichloromethane, 0.3 mmol, 1.5 eq.) was added to the mixture. After 10 minutes NMR spectroscopy indicated 1-phenyl-1-propyne was fully consumed and a single product was formed cleanly based on the NMR spectra. ON keeping the reaction mixture at room temperature for longer times, NMR studies suggests the formation of two new species.

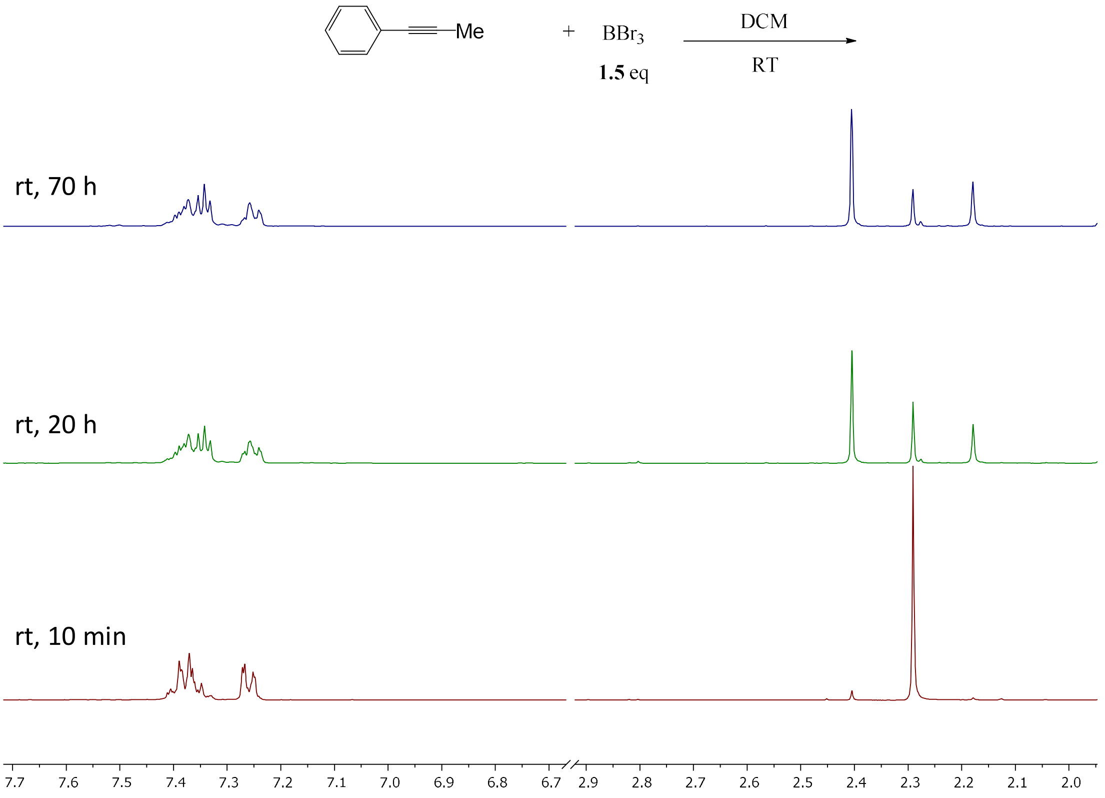

Figure S27. Stacked ^{1}H NMR spectra of the bromoboration of 1-phenyl-1-propyne in dichloromethane.

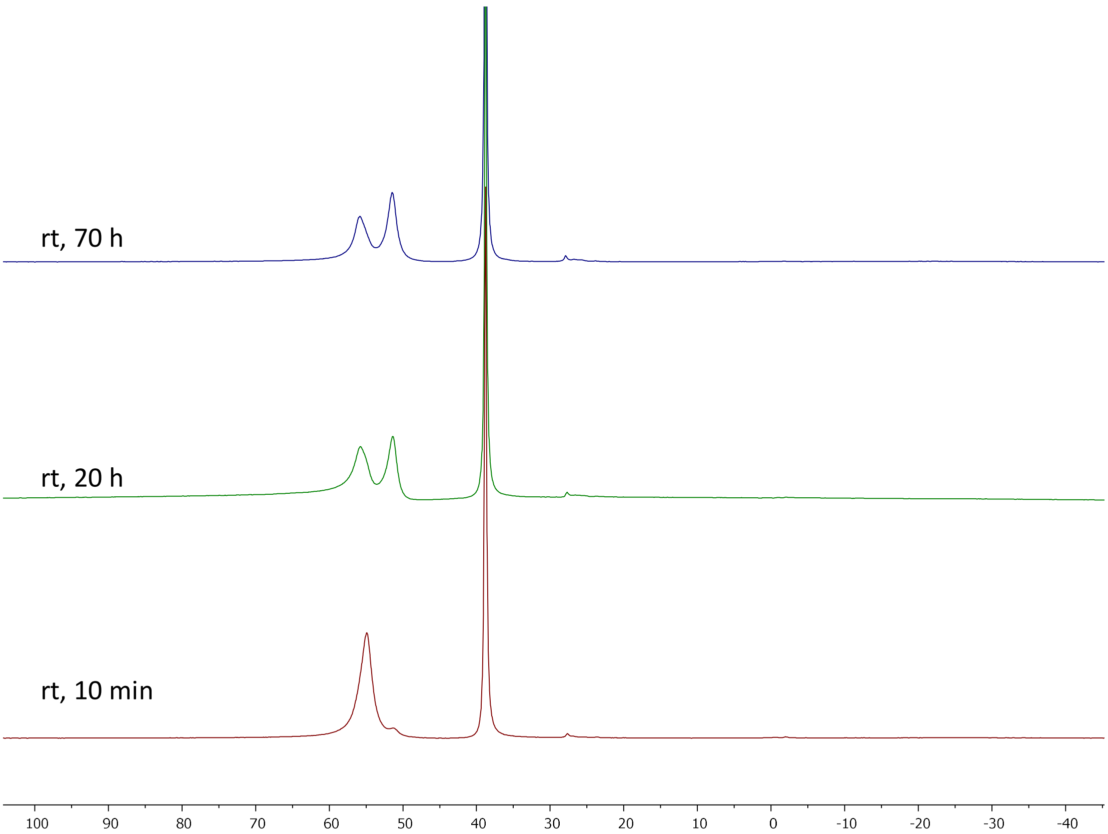

Figure S28. Stacked ^{11}B NMR spectra of the bromoboration of 1-phenyl-1-propyne in dichloromethane.

S4.4.2 Bromoboration and Protodeboronation

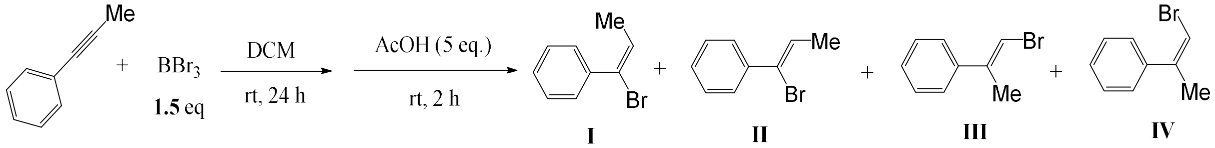

To an ampoule was added 1-phenyl-1-propyne (50 µL, 0.4 mmol, 1 eq) and 0.6 mL dichloromethane. Then 0.6 mL of boron tribromide solution (0.6 mL, 1 M in dichloromethane, 0.6 mmol, 1.5 eq.) was added to the mixture. The solution was kept stirring for 24 hours, then to the reaction mixture was added acetic acid (5 mL, 0.4 M in hexane, 2 mmol, 5 eq.). The generated mixture was stirred at room temperature for 2 hours. The protodeboronation products were isolated as a mixture by flash column chromatography using silica and a hexane eluent. The ^{1}H NMR spectrum indicated the formation of *E*-(1-bromoprop-1-en-1-yl)benzene **I** and *E*-(1-bromoprop-1-en-2-yl)benzene **III** as the major products along with some other mixtures. Combined yield: 36 mg, 46%.

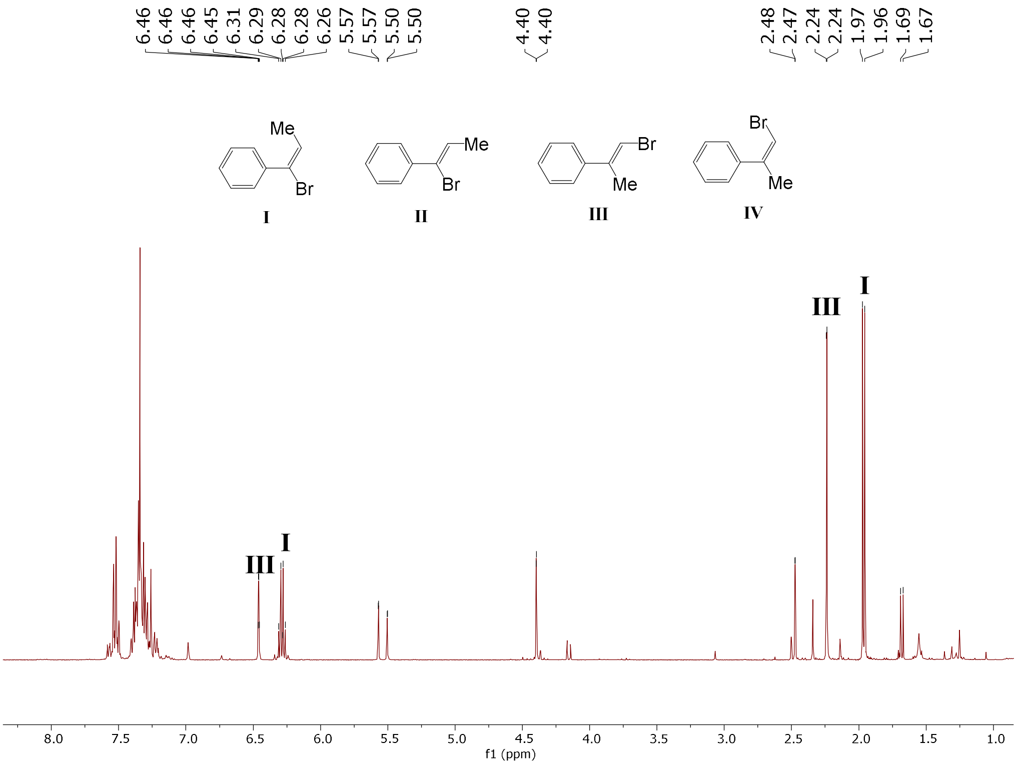

Figure S29. ^{1}H NMR spectrum showing the bromoboration-protodeboronation of 1-phenyl-1-propyne afforded a complex mixture containing *E*-(1-bromoprop-1-en-1-yl)benzene (**I**)and *E*-(1-bromoprop-1-en-2-yl)benzene (**III**)

S4.5 Reaction of the bromoboration products with pyridine and pinacol protection

To a J-Young NMR tube charged with di-*p*-tolylacetylene (41 mg, 0.2 mmol, 1 eq) was added 0.6 mL dichloromethane to form a clear solution. Then 0.3 mL of boron tribromide (1 M in heptane, 0.3 mmol, 1.5 eq.) was added to the solution. NMR studies right after BBr_3 addition show full consumption of di-*p*-tolyl-acetylene and the formation of two species in a 0.3:1 ratio based on the integration of methyl group resonances. To the NMR tube was added 24 µL pyridine (0.3 mmol, 1.5 eq). NMR spectrum showed the formation of di-*p*-tolylacetylene and a new compound in a 0.3:1 ratio, which indicates the di-*p*-tolyl-acetylene was produced from the minor species. Upon heating at 60 °C, the new compound was converted to di-*p*-tolylacetylene along with the formation of small amounts of protodeboronation product.

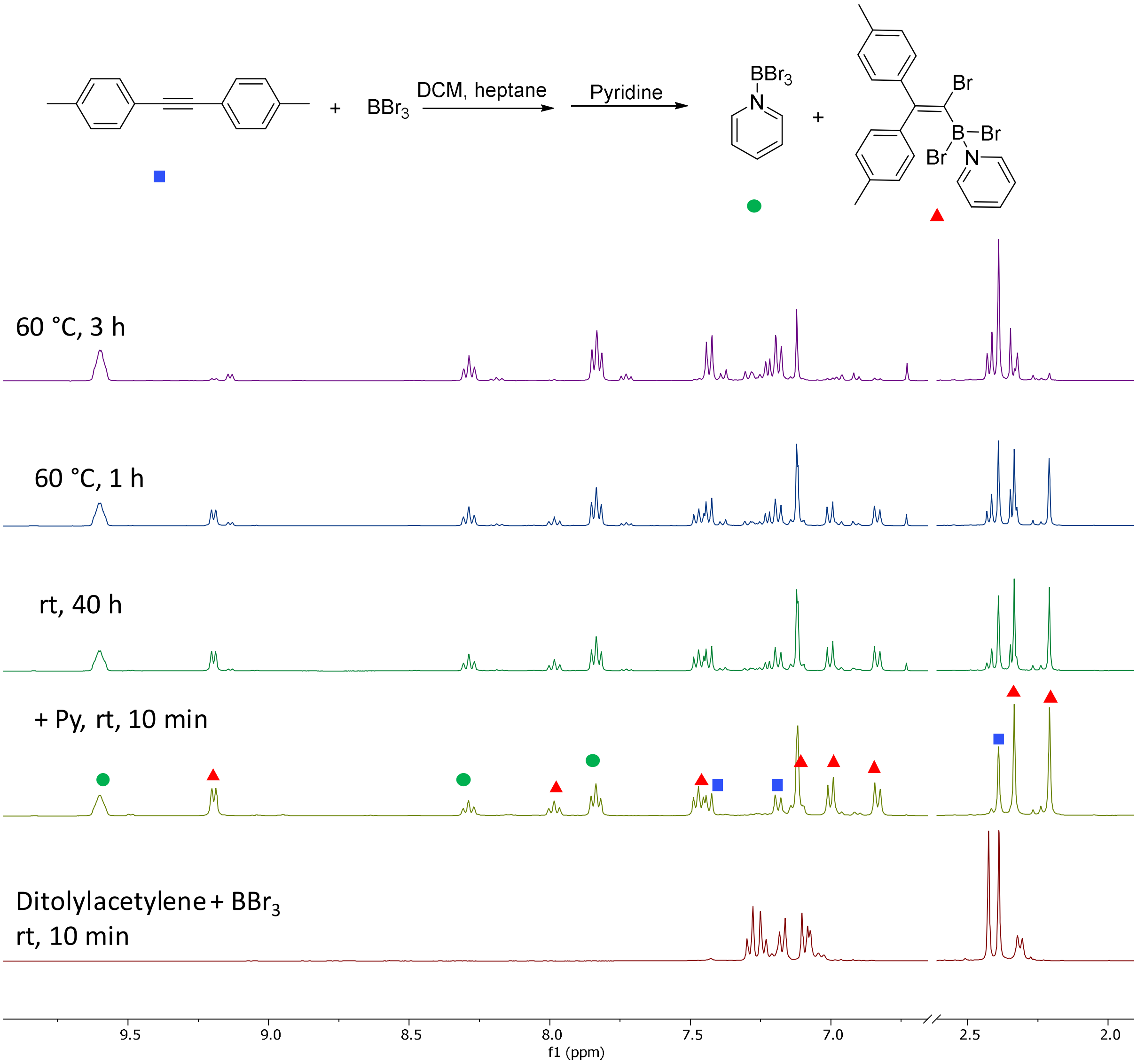

Figure S30. Stacked ^{1}H NMR spectra showing the bromoboration of di-*p*-tolylacetylene and transformation upon addition of 1.5 equiv. pyridine with the 1,2-bromoboration product rapidly converted to di-p-tolylacetylene and py-BBr_3, whereas the 1,1-bromoboration product converts more slowly back to the alkyne..

To a J-Young NMR tube charged with di-*p*-tolyl-acetylene (41 mg, 0.2 mmol, 1 eq) was added 0.6 mL dichloromethane to form a clear solution. Then 0.3 mL of boron tribromide (1 M in heptane, 0.3 mmol, 1.5 eq.) was added to the solution. NMR studies right after the BBr_3 addition show a full consumption of di-*p*-tolyl-acetylene and the formation of two species in a 3:10 ratio based on the integration of methyl group resonances. To the NMR tube was added 24 µL pyridine (0.3 mmol, 1.5 eq). The ^{1}H NMR spectrum shows the formation of di-*p*-tolyl-acetylene and a new compound in a 3:10 ratio. To the reaction mixture was added another 0.3 mL of boron tribromide (1 M in heptane, 0.3 mmol, 1.5 eq.) to sequester all pyridine as pyridine-BBr_3, two bromoboration species in a 3:10 ratio were formed again.

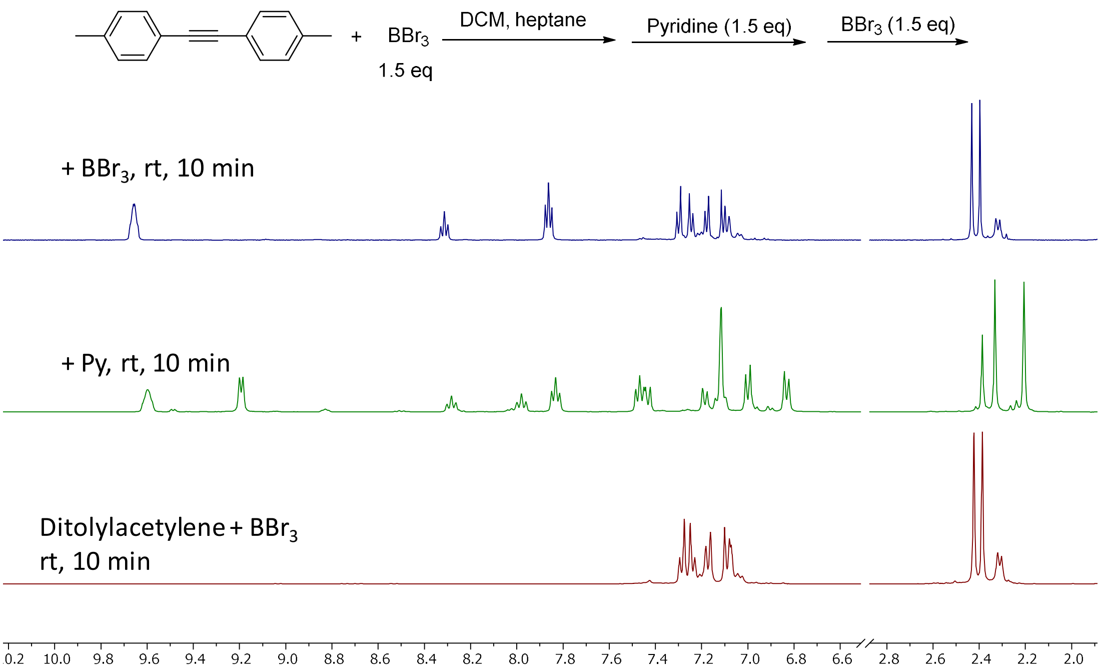

Figure S31. ^{1}H NMR spectrum showing the bromoboration of di-*p*-tolylacetylene and transformation upon addition of 1.5 equiv. pyridine and then 1.5 equiv. BBr_3.

S4.6 Pinacol protection

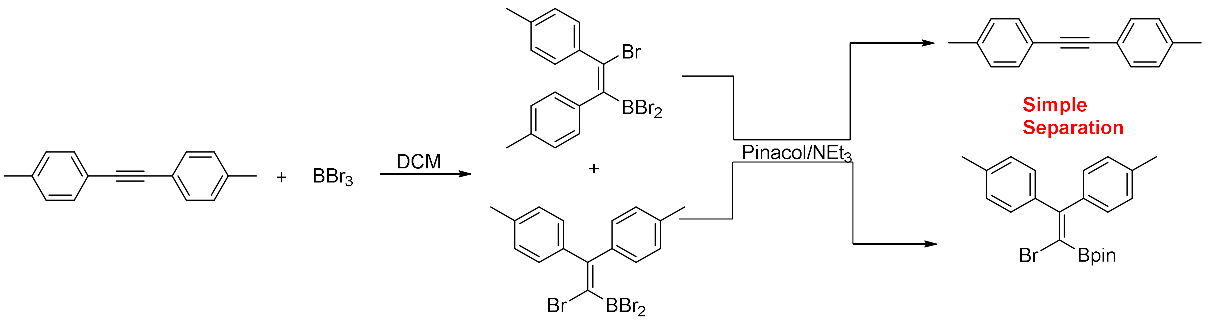

Since Lewis bases readily converted the 1,2-*cis*-bromoboration products into the alkyne starting materials (via retro-haloboration), while the 1,1-bromoboration products tended to form relatively stable Lewis acid-base adducts, the pinacol protection was carried out in the presence of an excess of NEt_3 in order to convert 1,2-*cis*-bromoboration product back into the starting alkyne for easy separation.

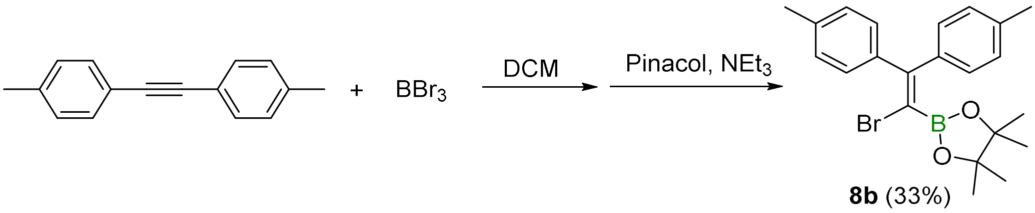

8b (33%)

Synthesis of **8b**

To an ampoule charged with di-*p*-tolyl-acetylene (41 mg, 0.2 mmol, 1 eq) was added 0.6 mL dichloromethane to form a clear solution. Then 0.3 mL of boron tribromide (1 M in dichloromethane, 0.3 mmol, 1.5 eq.) was added to the solution. The solution was kept stirring for 10 min, then to the reaction mixture was added pinacol (0.5 mL, 1 M in NEt_3, 0.5 mmol, 2.5 eq). The mixture was stirred at room temperature for 2 hours. The desired product was separated by flash column chromatography using silica and a hexane: ethyl acetate (20:1 to 10:1) eluent. Yield: 27 mg, 33%.

^{1}H NMR (601 MHz, $CDCl_3$) δ = 7.19 (d, J = 8.2 Hz, 2H), 7.15 (d, J = 7.9 Hz, 2H), 7.12 (d, J = 8.1 Hz, 2H), 7.07 (d, J = 7.9 Hz, 2H), 2.35 (s, 3H), 2.32 (s, 3H), 1.20 (s, 12H).

^{11}B NMR (128 MHz, $CDCl_3$) δ = 28.9.

^{13}C{^{1}H} NMR (151 MHz, $CDCl_3$) δ = 153.8, 139.3, 138.9, 138.2, 137.8, 129.5, 129.2, 128.9, 128.8, 84.5, 24.5, 21.5, 21.4.

HRMS (EI) Calculated for $C_{22}H_{26}BBrO_2$ ($[M]^+$) 412.1204. Found 412.1215.

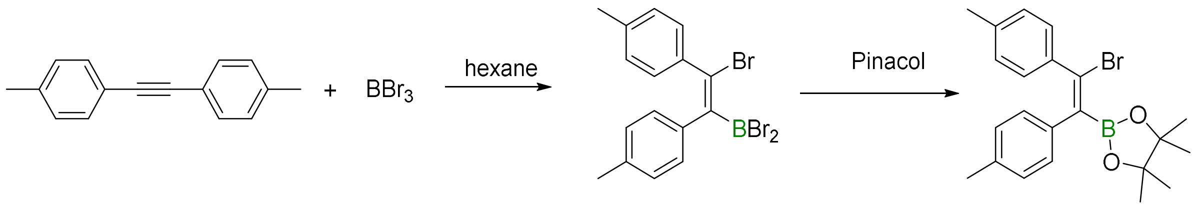

8b-1,2 (39%)

Synthesis of **8b-1,2**

To an ampoule charged with di-*p*-tolyl-acetylene (41 mg, 0.2 mmol, 1 eq) was added 0.6 mL hexane. Then 0.3 mL of boron tribromide (1 M in heptane, 0.3 mmol, 1.5 eq.) was added. The solution was kept stirring for 10 min, then to the reaction mixture was added pinacol (0.9 mL, 0.5 M in hexane, 0.45 mmol, 3 eq). The mixture was stirred at room temperature for 24 hours. The desired product was separated by flash column chromatography using silica and a hexane: ethyl acetate (20:1 to 10:1) eluent. Yield: 32 mg, 39%.

NMR ^{1}H NMR (400 MHz, $CDCl_3$) δ = 7.11 (d, J = 8.2 Hz, 2H), 6.96 (d, J = 7.9 Hz, 2H), 6.92 (m, 4H), 2.27 (s, 3H), 2.23 (s, 3H), 1.37 (s, 12H).

^{11}B NMR (128 MHz, $CDCl_3$) δ = 29.8.

^{13}C{^{1}H} NMR (101 MHz, $CDCl_3$) δ = 138.2, 137.4, 136.6, 136.3, 129.8, 129.1, 128.7, 128.6, 84.5, 24.8, 21.4, 21.3.

HRMS (EI) Calculated for $C_{22}H_{26}BBrO_2$ ($[M]^+$) 412.1204. Found 412.1212.

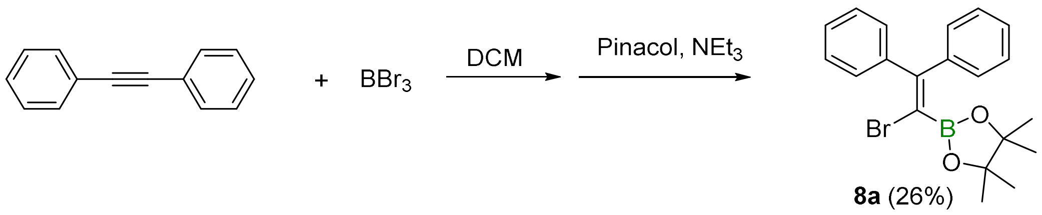

Synthesis of **8a**

To an ampoule charged with diphenylacetylene (53.4 mg, 0.3 mmol, 1 eq) was added 0.9 mL dichloromethane to form a clear solution. Then 0.45 mL of boron tribromide solution (1 M in dichloromethane, 0.45 mmol, 1.5 eq.) was added to the mixture and a light-yellow solution formed immediately. The solution was stirred for 2 hours, then to the reaction mixture was added pinacol (0.9 mL, 1 M in NEt_3, 0.9 mmol, 3 eq). The mixture was stirred at room temperature for 20 hours. The desired product was separated by flash column chromatography using silica and a hexane: ethyl acetate (20:1 to 10:1) eluent. Yield: 30 mg, 26%.

NMR ^1H NMR (400 MHz, $CDCl_3$) δ = 7.37 – 7.23 (m, 10H), 1.19 (s, 12H).

^{11}B NMR (128 MHz, $CDCl_3$) δ = 28.7.

$^{13}C\{^1H\}$ NMR (101 MHz, $CDCl_3$) δ = 153.9, 141.9, 141.6, 129.4, 129.2, 128.3, 128.2, 128.1, 128.0, 84.6, 24.5.

HRMS (EI) Calculated for $C_{20}H_{22}BBrO_2$ ($[M]^+$) 384.0891. Found 384.0908.

Confirming retro-haloboration of the 1,2-product in the presence of amine base

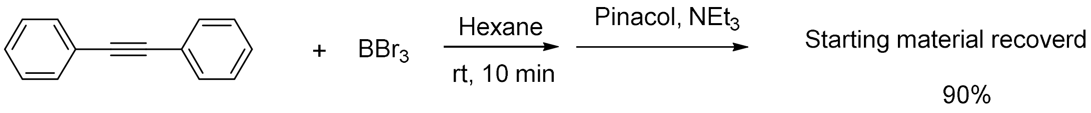

To an ampoule charged with diphenylacetylene (89 mg, 0.5 mmol, 1 eq) was added a solution of boron tribromide (0.75 mL, 1 M in heptane, 0.75 mmol, 1.5 eq.). A clear light-yellow solution was formed immediately. The solution was kept stirring at room temperature for 10 minutes, then to the reaction mixture was added pinacol (2 mL, 1 M in NEt_3, 2 mmol, 4 eq). A brown suspension was formed instantly. The mixture was stirred at room temperature for 20 hours. Flash column chromatography of the mixture using silica and a hexane eluent afforded diphenylacetylene (90% isolated yield).

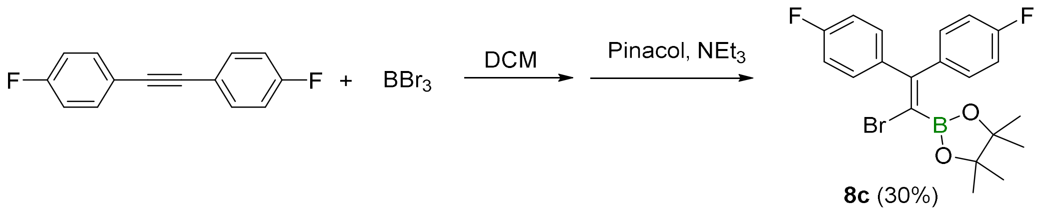

Synthesis of **8c**

To an ampoule charged with 1,2-bis(4-fluorophenyl)ethyne (64.2 mg, 0.3 mmol, 1 eq) was added 0.9 mL dichloromethane to form a clear solution. Then 0.45 mL of boron tribromide solution (1 M in dichloromethane, 0.45 mmol, 1.5 eq.) was added to the mixture. The solution was kept stirring for 2 hours, then to the reaction mixture was added pinacol (0.9 mL, 1 M in NEt_3, 0.9 mmol, 3 eq). The mixture was stirred at room temperature for 20 hours. The desired product was separated by flash column chromatography using silica and a hexane: ethyl acetate (20:1 to 10:1) eluent. Yield: 38 mg, 30%.

^{1}H NMR (400 MHz, $CDCl_3$) δ = 7.31 – 7.28 (m, 2H), 7.25 – 7.20 (m, 2H), 7.08 – 7.04 (m, 2H), 7.02 – 6.98 (m, 2H), 1.22 (s, 12H).

^{11}B NMR (128 MHz, $CDCl_3$) δ = 28.8.

^{19}F{^{1}H} NMR (376 MHz, $CDCl_3$) δ = -113.07, -113.13.

^{13}C{^{1}H} NMR (101 MHz, $CDCl_3$) δ = 163.9 (d, J = 55.2 Hz), 161.5 (d, J = 54.9 Hz), 151.8, 137.9 (d, J = 3.3 Hz), 137.2 (d, J = 3.4 Hz), 131.4 (d, J = 8.3 Hz), 131.0 (d, J = 8.2 Hz), 115.3 (d, J = 3.6 Hz), 115.1 (d, J = 3.5 Hz), 84.7, 24.5.

HRMS (EI) Calculated for $C_{20}H_{20}BBrF_2O_2$ ($[M]^+$) 420.0717. Found 420.0716.

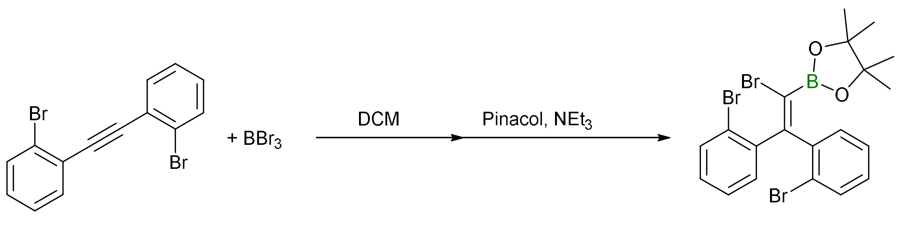

8d (39%)

Synthesis of **8d**

1,2-Bis(2-bromophenyl)acetylene (34.0 mg, 100 µmol, 1 eq.) was dissolved in dry dichloromethane (0.5 mL) and treated with BBr_3 (1 m in dichloromethane, 0.15 mL, 150 µmol, 1.5 eq.), giving a dark green solution. After three hours at room temperature, pinacol (1 m in NEt_3, 0.30 mL, 300 µmol, 3 eq.) was added and the resulting blue suspension was stirred for 18 h at room temperature. The reaction

mixture was filtered through a plug of silica using an EtOAc eluent. After evaporation of the solvent, the desired product was separated by column chromatography using silica and an n-hexane→EtOAc eluent and obtained (in ca. 95% purity) as an off-white solid. Yield: 15 mg, 39 µmol, 39%. Recrystallization from MeOH gave the pure compound as colourless crystals. Additionally, 20 mg (59 µmol, 59%) of the starting material were recovered.

^{1}H NMR (400 MHz, $CDCl_3$) δ = 7.43 (dd, J = 8.0, 1.2 Hz, 1H), 7.40 (dd, J = 7.9, 1.3 Hz, 1H), 7.15 (ddd, J = 9.7, 7.6, 1.8 Hz, 2H), 7.06 (td, J = 7.5, 1.3 Hz, 1H), 7.03 – 6.99 (m, 1H), 6.99 – 6.95 (m, 1H), 6.95 – 6.90 (m, 1H), 1.35 (s, 6H), 1.34 (s, 6H).

^{13}C{^{1}H} NMR (126 MHz, $CDCl_3$) δ = 141.7, 141.3 (2x), 132.9, 132.0, 131.3, 130.2, 129.7, 128.6, 127.4, 127.3, 122.6, 122.6, 84.7, 25.1, 24.8; n.o.: C−Bpin.

^{11}B{^{1}H} NMR (128 MHz, $CDCl_3$) δ = 29.3.

HRMS (ESI) Calculated for $C_{20}H_{20}BBr_3KO_2$ ([M]·K^+) 578.8743. Found 578.8745.

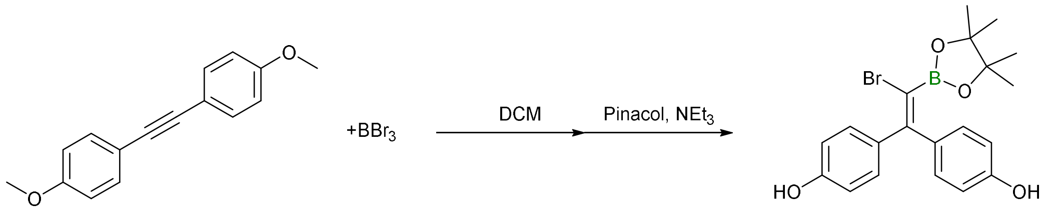

8e (28%)

<u>Synthesis of **8e**</u>

1,2-Bis(4-methoxyphenyl)acetylene (24.0 mg, 100 µmol, 1 eq.) was dissolved in dry dichloromethane (0.6 mL) and treated with BBr_3 (1 m in dichloromethane, 0.15 mL, 150 µmol, 1.5 eq.), turning the colourless clear solution into a purple suspension. After 10 min at room temperature, pinacol (1 m in NEt_3, 0.30 mL, 300 µmol, 3 eq.) was added and the resulting suspension was stirred for 18 h at room temperature. The reaction mixture was filtered through a plug of silica using an EtOAc eluent. After evaporation of the solvent, the desired product was separated by column chromatography using silica and an n-hexane→EtOAc eluent. Yield: 8 mg, 28 µmol, 28%.

^{1}H NMR (400 MHz, $CDCl_3$) δ = 7.22 – 7.14 (m, 2H), 7.13 – 7.05 (m, 2H), 6.80 – 6.77 (m, 2H), 6.74 – 6.68 (m, 2H), 1.20 (s, 12H).

^{13}C{^{1}H} NMR (101 MHz, $CDCl_3$) δ = 155.9, 155.4, 153.0, 135.1, 134.2, 131.4, 131.0, 115.6, 115.1, 114.9, 84.6, 24.5.

^{11}B{^{1}H} NMR (128 MHz, $CDCl_3$) δ = 30.0.

HRMS (EI) Calculated for $C_{20}H_{22}BBrO_4$ ([M]$^+$) 416.0789. Found 416.0785.

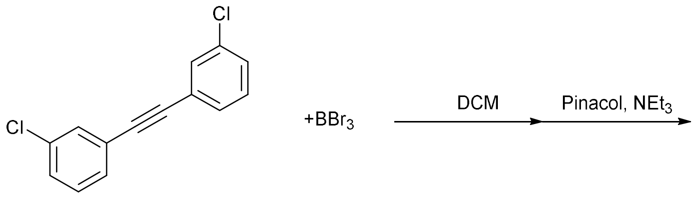

8f (32%)

Synthesis of **8f**

1,2-Bis(3-chlorophenyl)acetylene (75 mg, 0.35 mmol, 1 eq.) was dissolved in dry dichloromethane (3.5 mL) and treated with BBr_3 (1 m in dichloromethane, 0.52 mL, 0.52 mmol, 1.5 eq.). After 20 min at room temperature, pinacol (123 mg, 1.04 mmol, 3 eq.) was added as a solid and the resulting suspension was stirred for 3 h at room temperature. After evaporation of the solvent, the desired product was separated by column chromatography using silica and an n-hexane→dichloromethane eluent. Yield: 46 mg, 0.11 mmol, 32%. Additionally, 43 mg of the starting material was recovered.

^{1}H NMR (400 MHz, $CDCl_3$) δ = 7.23 (t, J = 1.8 Hz, 1H), 7.16 (ddd, J = 8.0, 2.1, 1.2 Hz, 1H), 7.12 – 7.09 (m, 1H), 7.09 – 7.05 (m, 2H), 7.04 – 7.03 (m, 1H), 7.00 (dt, J = 7.7, 1.4 Hz, 1H), 6.85 (dt, J = 7.5, 1.5 Hz, 1H), 1.36 (s, 12H).

^{13}C{^{1}H} (126 MHz, $CDCl_3$) δ = 141.4, 140.8, 134.3, 134.1, 129.8, 129.7, 129.4, 128.8, 128.6, 128.4, 127.9, 127.3, 126.9, 84.9, 24.8; n.o.: C−Bpin.

^{11}B{^{1}H} NMR (128 MHz, $CDCl_3$) δ = 29.6.

HRMS (EI) Calculated for $C_{20}H_{20}BBrCl_2O_2$ ($[M]^+$) 452.0111. Found 452.0113.

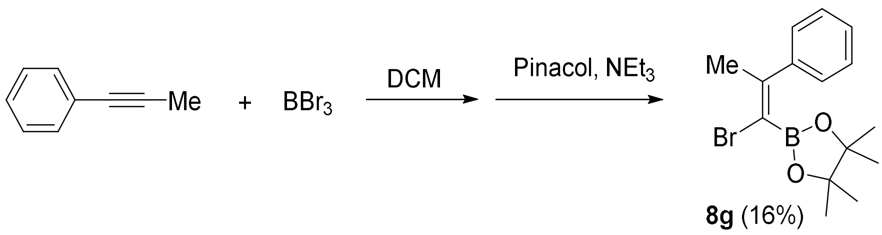

8g (16%)

Synthesis of **8g**

To an ampoule was added 1-phenyl-1-propyne (25 µL, 0.2 mmol, 1 eq) and 0.6 mL dichloromethane. Then 0.6 mL of boron tribromide solution (0.3 mL, 1 M in dichloromethane, 0.3 mmol, 1.5 eq.) was added to the mixture. The solution was kept stirring for 24 hours, then to the reaction mixture was added pinacol (0.6 mL, 1 M in NEt_3, 0.6 mmol, 3 eq). The mixture was stirred at room temperature for

20 hours. The desired product **8d** was separated by flash column chromatography using silica and a hexane: ethyl acetate (20:1 to 10:1) eluent. Yield: 10 mg, 16%.

^{1}H NMR (500 MHz, CD_2Cl_2) δ = 7.31 (m, 3H), 7.28 – 7.23 (m, 2H), 2.29 (s, 3H), 1.12 (s, 12H).

^{11}B NMR (160 MHz, CD_2Cl_2) δ = 28.4.

^{13}C{^{1}H} NMR (126 MHz, CD_2Cl_2) δ = 150.7, 142.3, 128.1, 127.9, 127.4, 84.1, 24.4, 24.1.

Isomer identity was determined by ^{1}H-^{1}H NOESY spectroscopy.

HRMS (EI) Calculated for $C_{15}H_{21}BBrO_2$ ($[M+H]^+$) 323.0813. Found 323.0783.

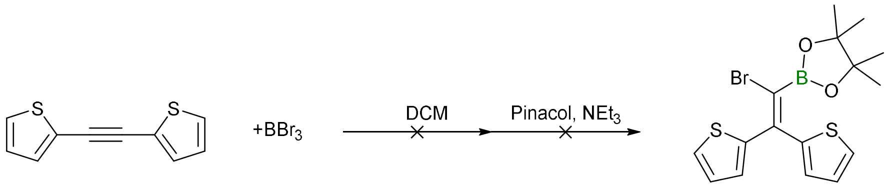

Attempted synthesis of 1-bromo-1-pinacolboryl-2,2-bis(2-thienyl)ethylene

1,2-Bis(2-thienyl)acetylene (19.0 mg, 100 µmol, 1 eq.) was dissolved in dry dichloromethane (0.6 mL) and treated with BBr_3 (1 m in dichloromethane, 0.15 mL, 150 µmol, 1.5 eq.), turning the yellow clear solution into a crimson suspension. After 10 min at room temperature, pinacol (1 m in NEt_3, 0.30 mL, 300 µmol, 3 eq.) was added and the resulting brown suspension was stirred for 18 h at room temperature. The reaction mixture was filtered through a plug of silica using an EtOAc eluent. After evaporation of the solvent, the mixture was separated by column chromatography using silica and an n-hexane→dichloromethane eluent. No defined product was obtained. Signals in between 6.50 to 7.00 ppm indicated the presence of vinylic H-atoms in the complex mixture (see spectra below). These might result from protodeborylation. In another run, 0.5 eq of the non-nucleophilic base 2,6-di-*tert*.-butyl-4-methylpyridine were added prior to the treatment with BBr_3. Still, no defined compound could be isolated.

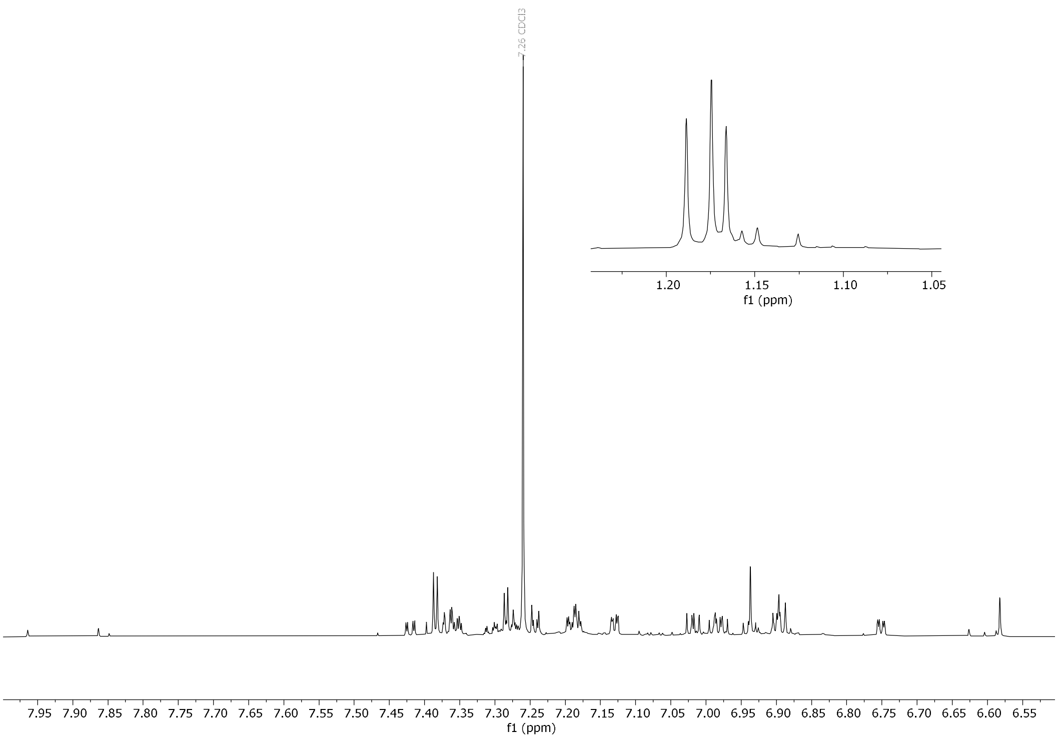

Figure S32: Aromatic region of the ^{1}H NMR spectrum ($CDCl_3$) of the isolated fraction after column chromatography. The reaction was carried out in the absence of 2,6-di-tert-butyl-4-methylpyridine.

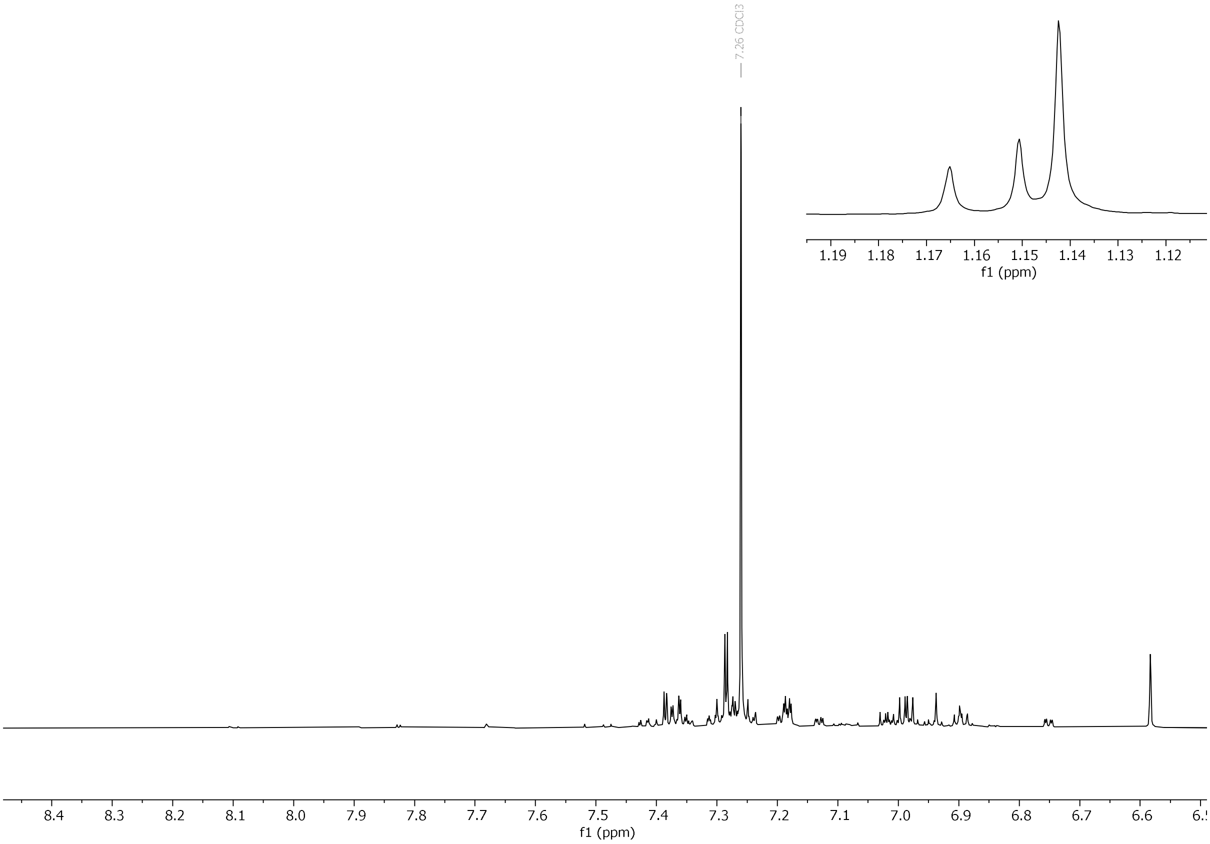

Figure S33: Aromatic region of the ^{1}H NMR spectrum ($CDCl_3$) of the isolated fraction after column chromatography. The reaction proceeded in the presence of 2,6-di-tert.-butyl-4-methylpyridine.

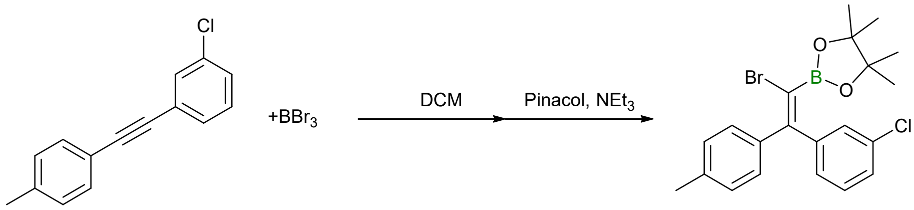

8h

<u>Attempted synthesis of 1-bromo-1-pinacolboryl-2-(3-chlorophenyl)-2-(4-methylphenyl)ethylene</u>

1-(3-chlorophenyl)-2-(4-methylphenyl)-acetylene (100 mg, 0.45 mmol, 1 eq.) was dissolved in dry dichloromethane (2.0 mL) and treated with BBr_3 (1 m in dichloromethane, 0.67 mL, 0.67 mmol, 1.5 eq.). After 10 min at room temperature, pinacol (1 m in NEt_3, 1.34 mL, 1.34 mmol, 3 eq.) was added and the resulting yellow suspension was stirred for 18 h at room temperature. The reaction mixture was filtered through a plug of silica using an EtOAc eluent. After evaporation of the solvent, the mixture was separated by column chromatography using silica and an n-hexane→dichloromethane eluent. A complex mixture was isolated.

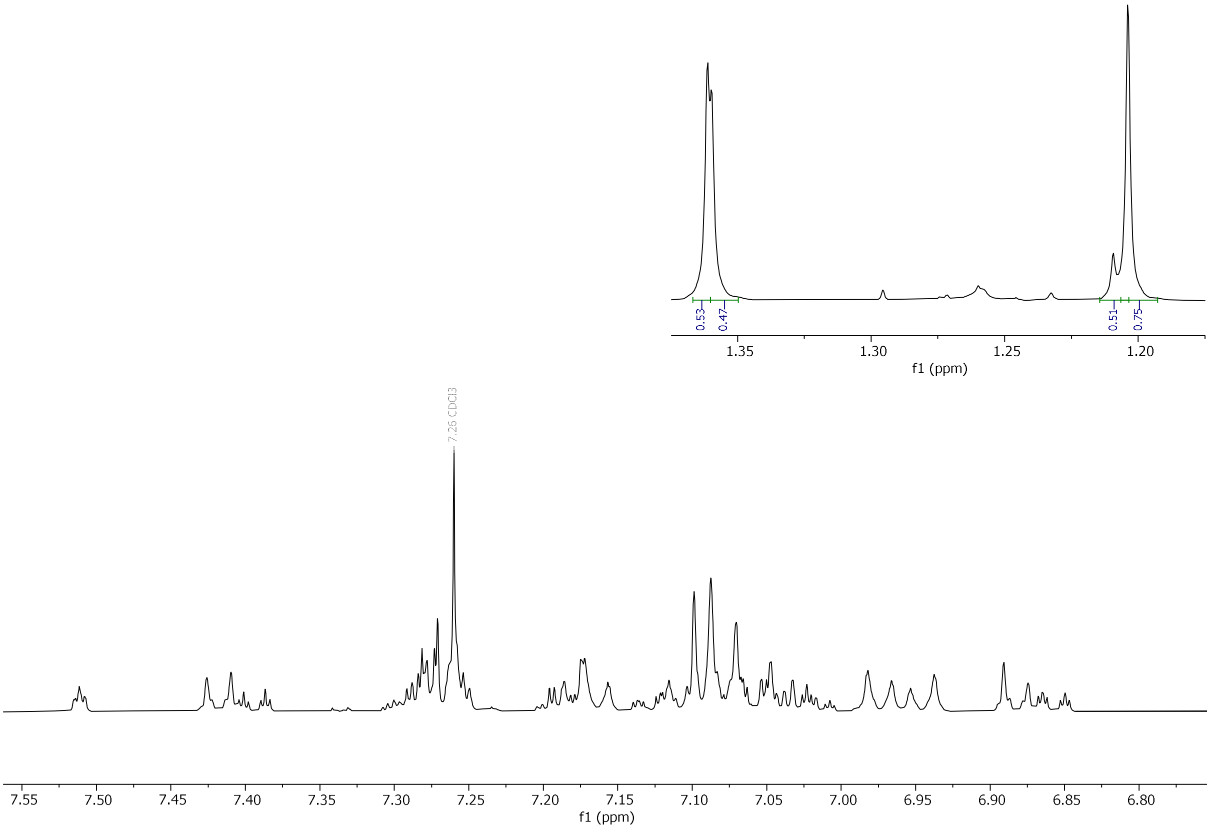

Figure S34: ^{1}H NMR spectrum of the isolated fraction after column chromatography (only aromatic region and region of the pinacol resonances) in $CDCl_3$.

S5. Photophysical Properties

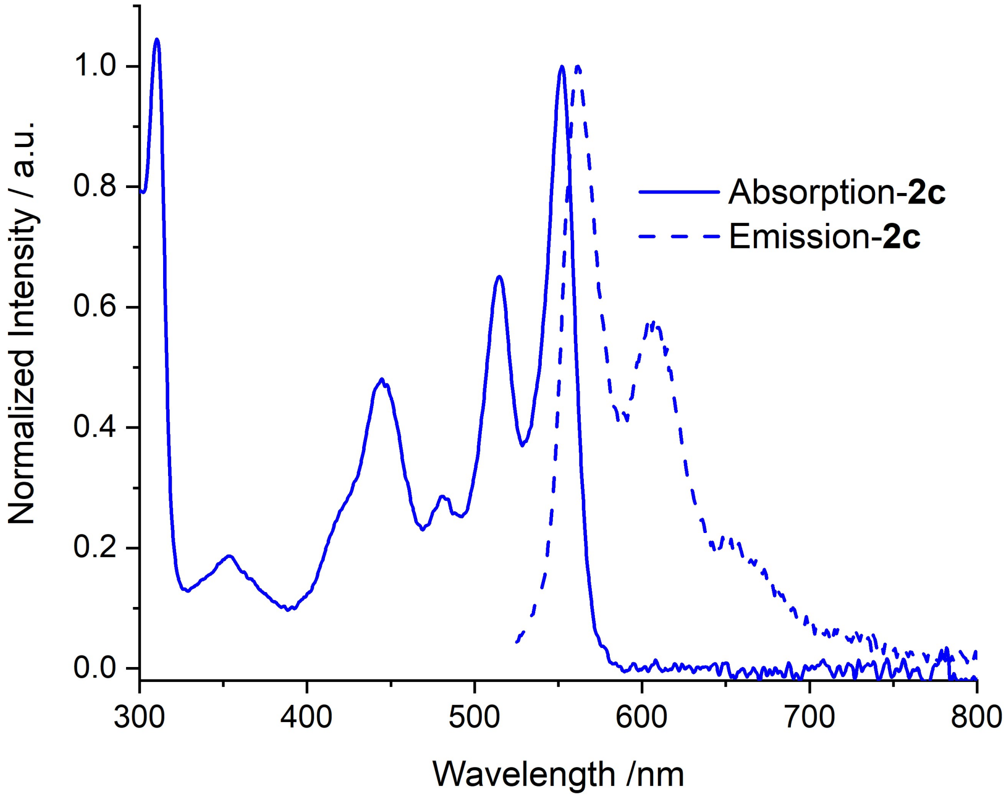

Figure S35. Absorption and emission spectra of compound **2c** in toluene (10^{-5} M).

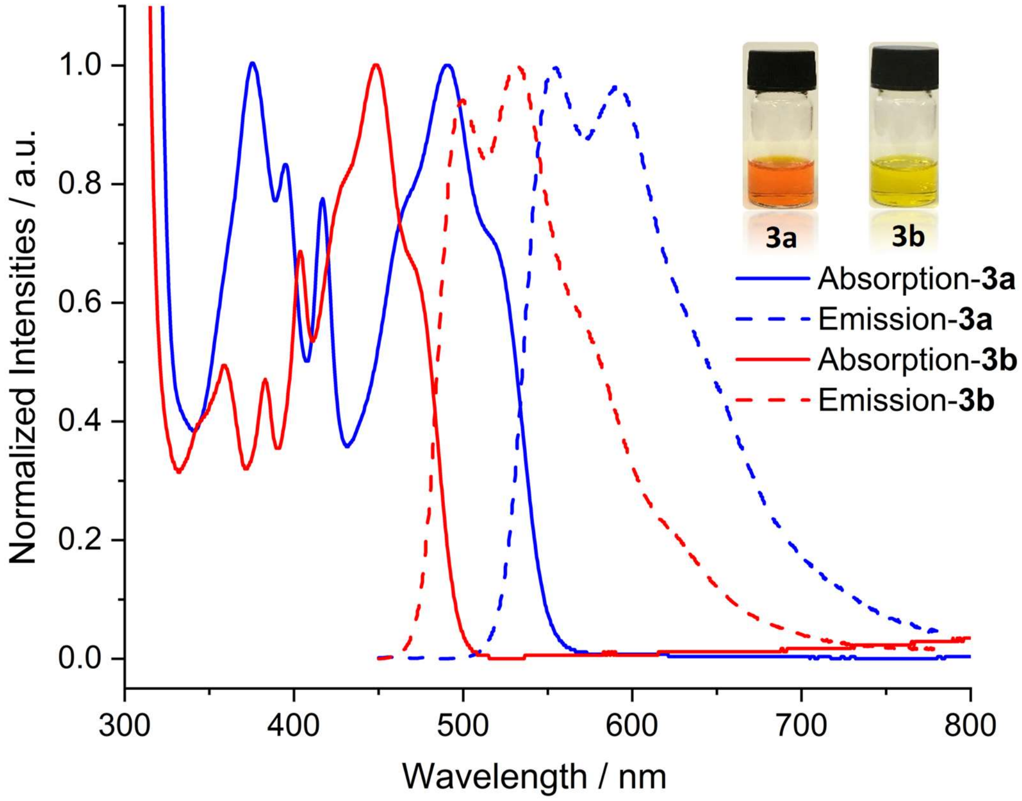

Figure S36. Absorption and emission spectra of compound **3a** and **3b** in toluene (10^{-5} M).

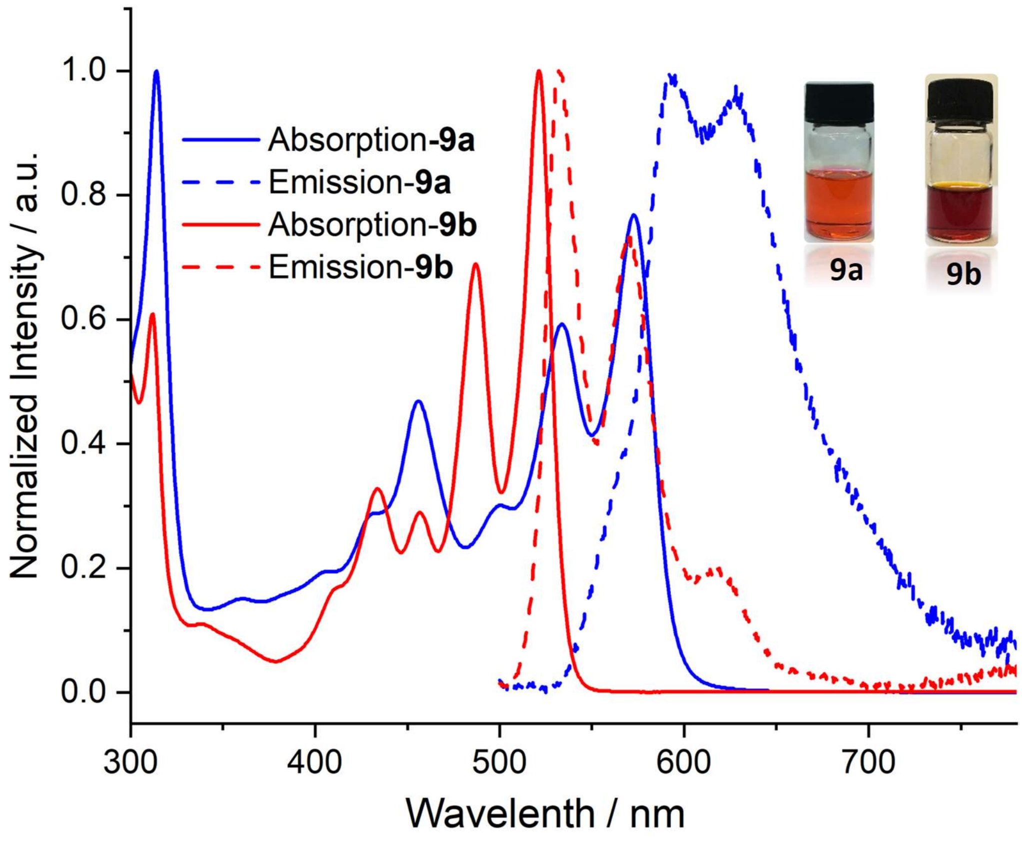

Figure S37. Absorption and emission spectra of compound **9a** and **9b** in toluene (10^{-5} M).

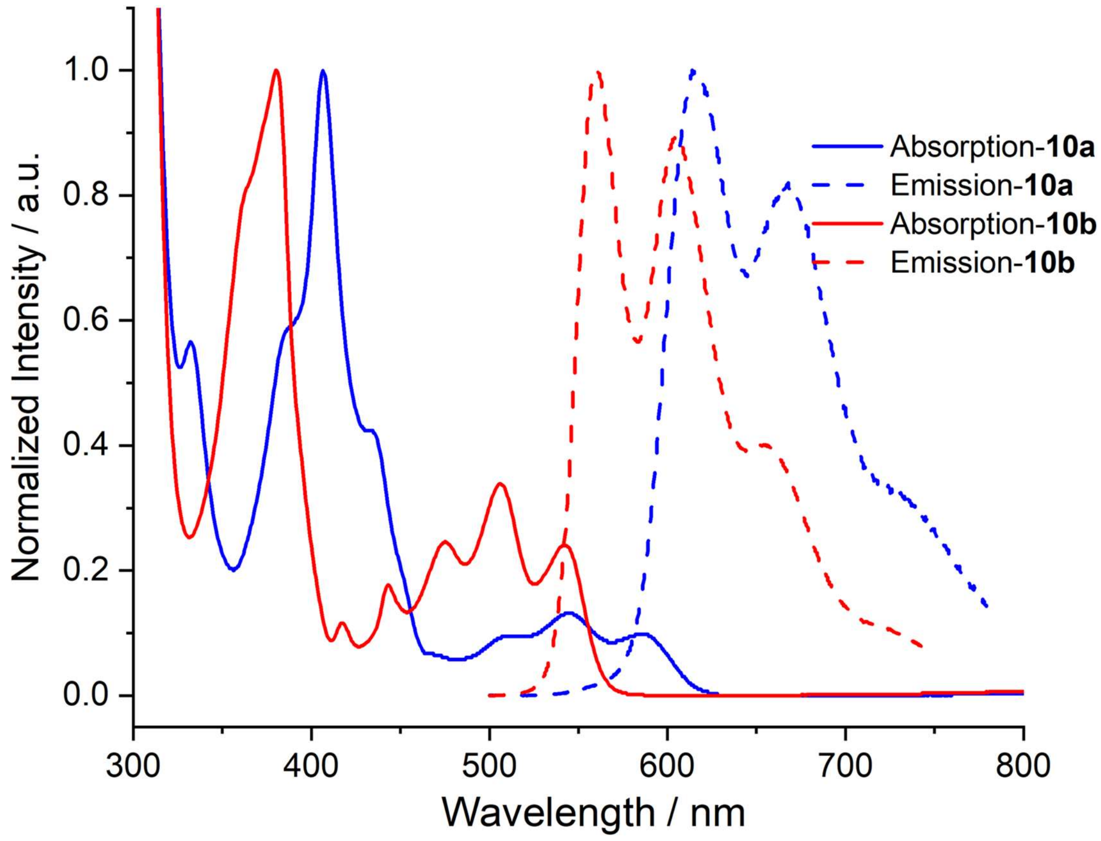

Figure S38. Absorption and emission spectra of compound **10a** and **10b** in toluene (10^{-5} M).

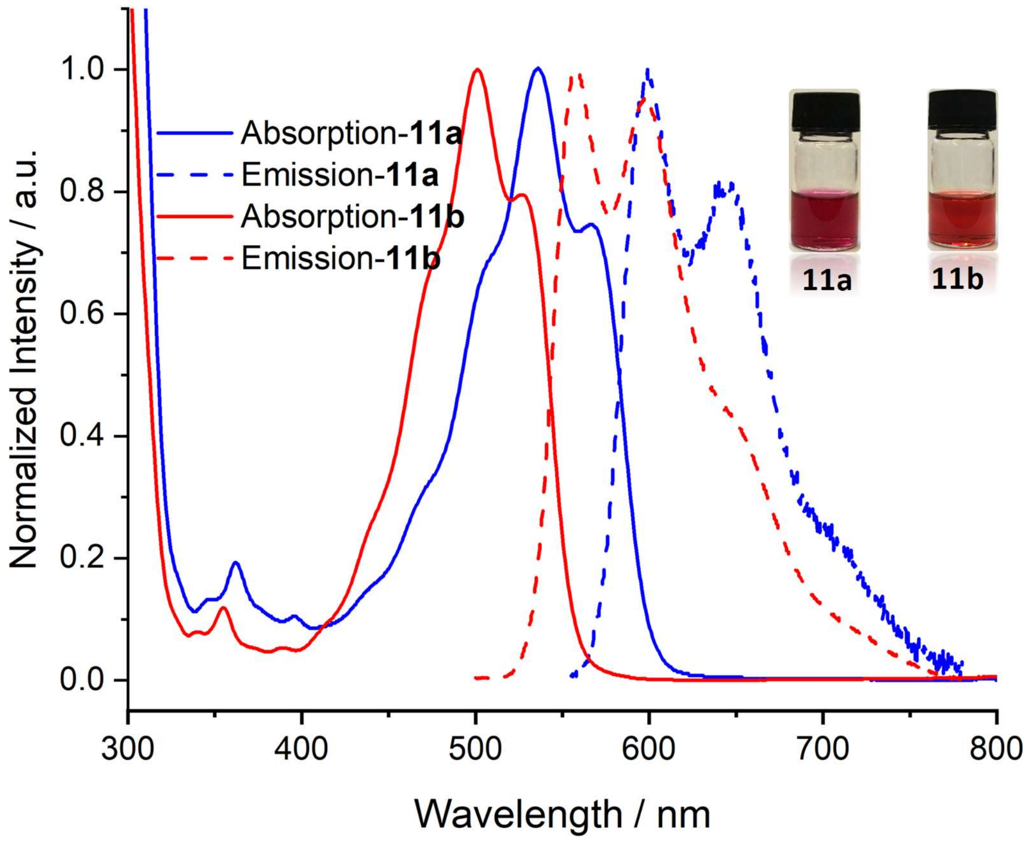

Figure S39. Absorption and emission spectra of compound **11a** and **11b** in toluene (10^{-5} M).

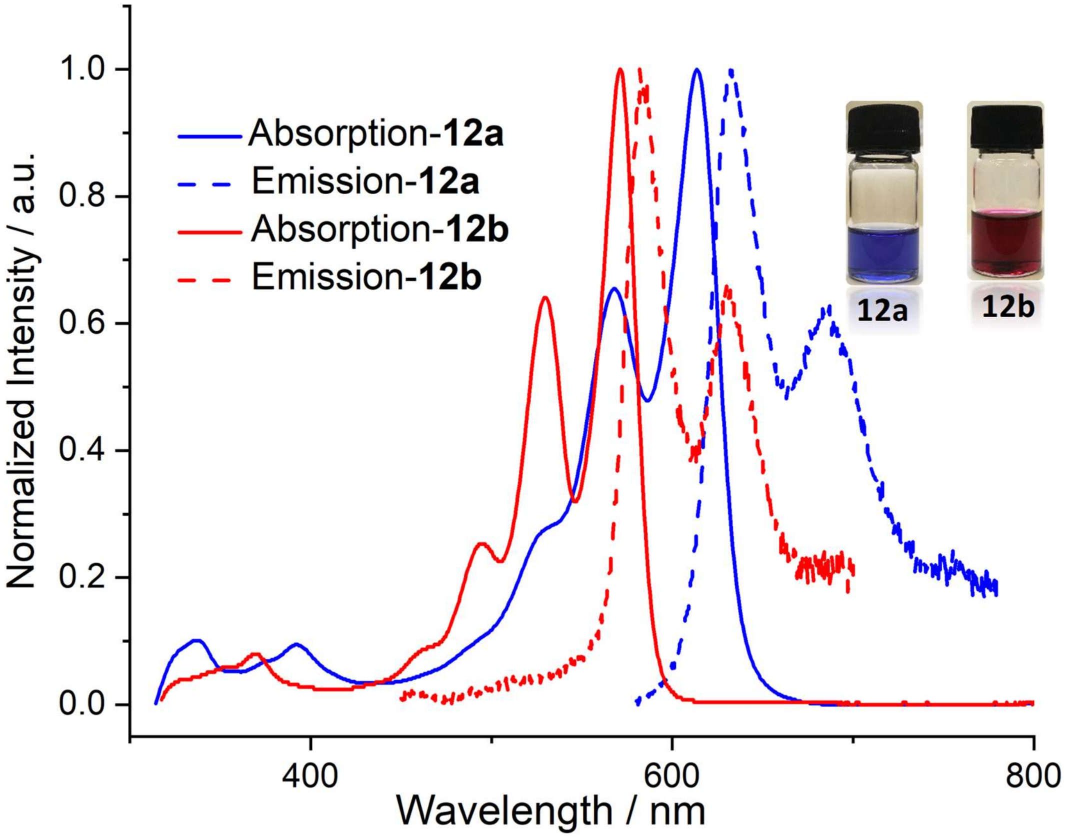

Figure S40. Absorption and emission spectra of compound **12a** and **12b** in toluene (10^{-5} M).

S6. Electrochemical Properties

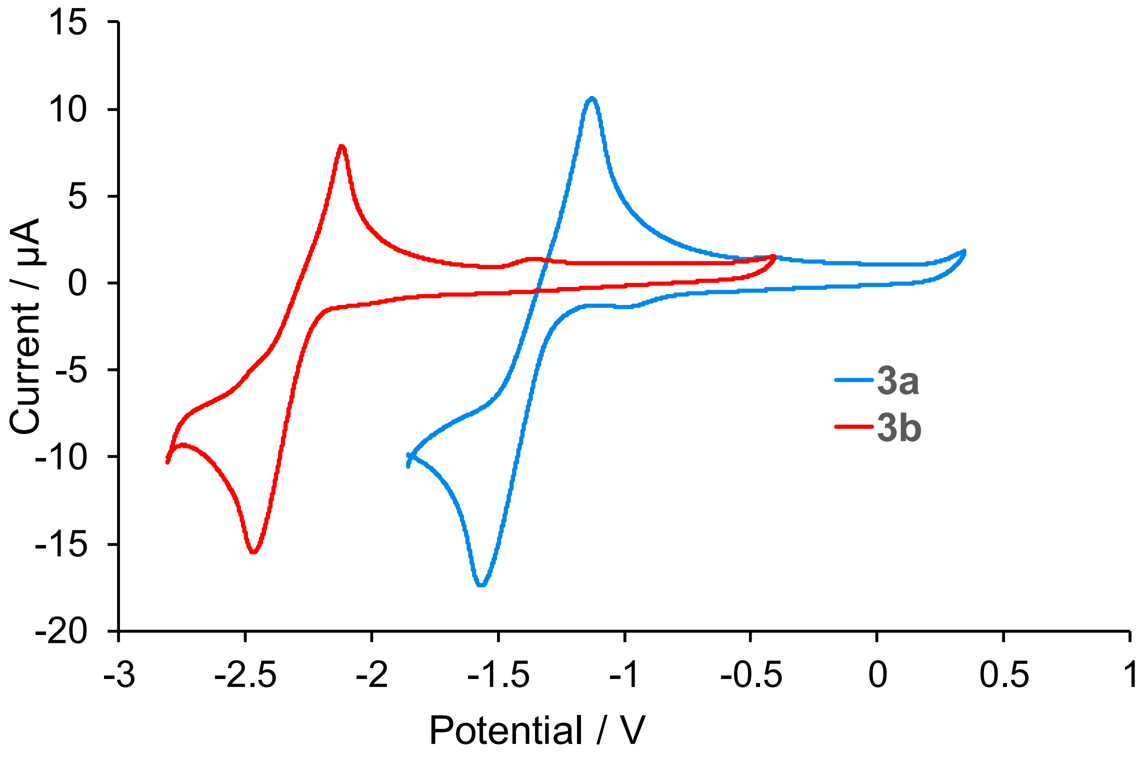

Figure S41. Cyclic Voltammetry diagrams of **3a** and **3b**. Measured in THF (1 mM), with [nBu$_4$N][PF$_6$] (0.1 M) as the supporting electrolyte at a scan rate of 100 mV/s.

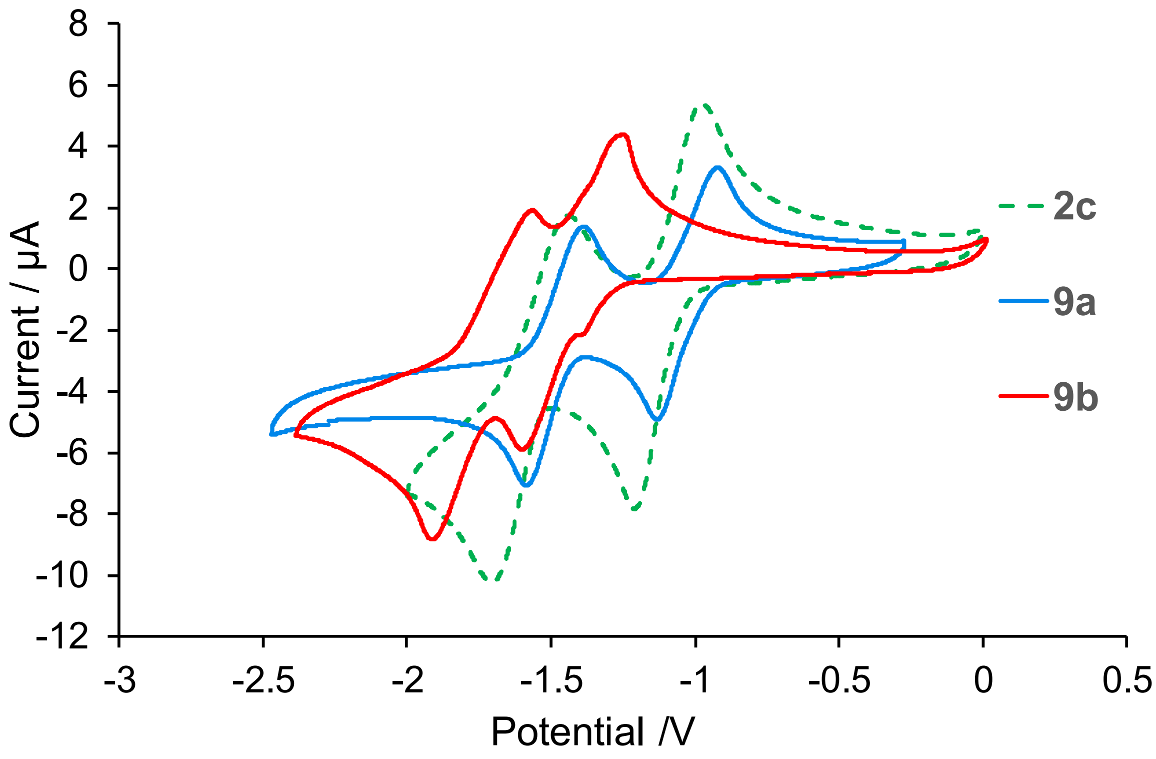

Figure S42. Cyclic Voltammetry diagrams of **2c, 9a** and **9b**. Measured in THF (1 mM), with [nBu$_4$N][PF$_6$] (0.1 M) as the supporting electrolyte at a scan rate of 100 mV/s.

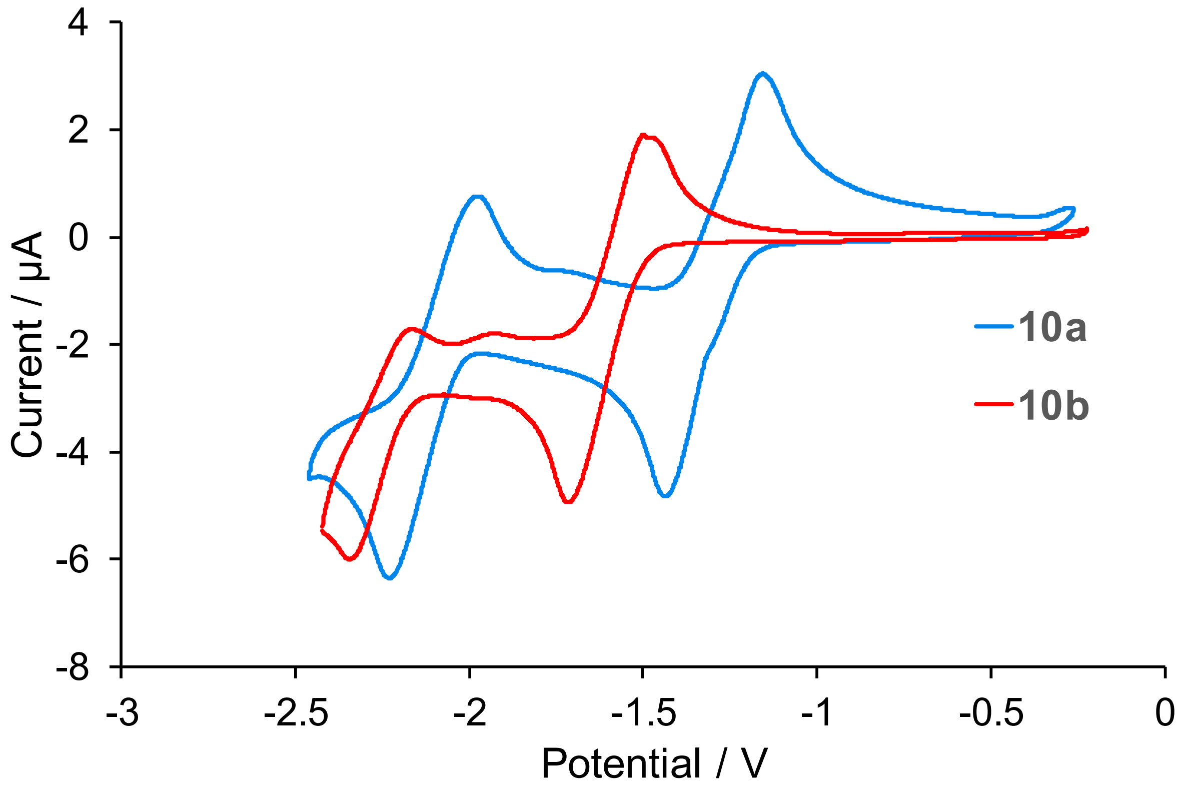

Figure S43. Cyclic Voltammetry diagrams of **10a** and **10b**. Measured in THF (1 mM), with [*n*Bu_4N][PF_6] (0.1 M) as the supporting electrolyte at a scan rate of 100 mV/s.

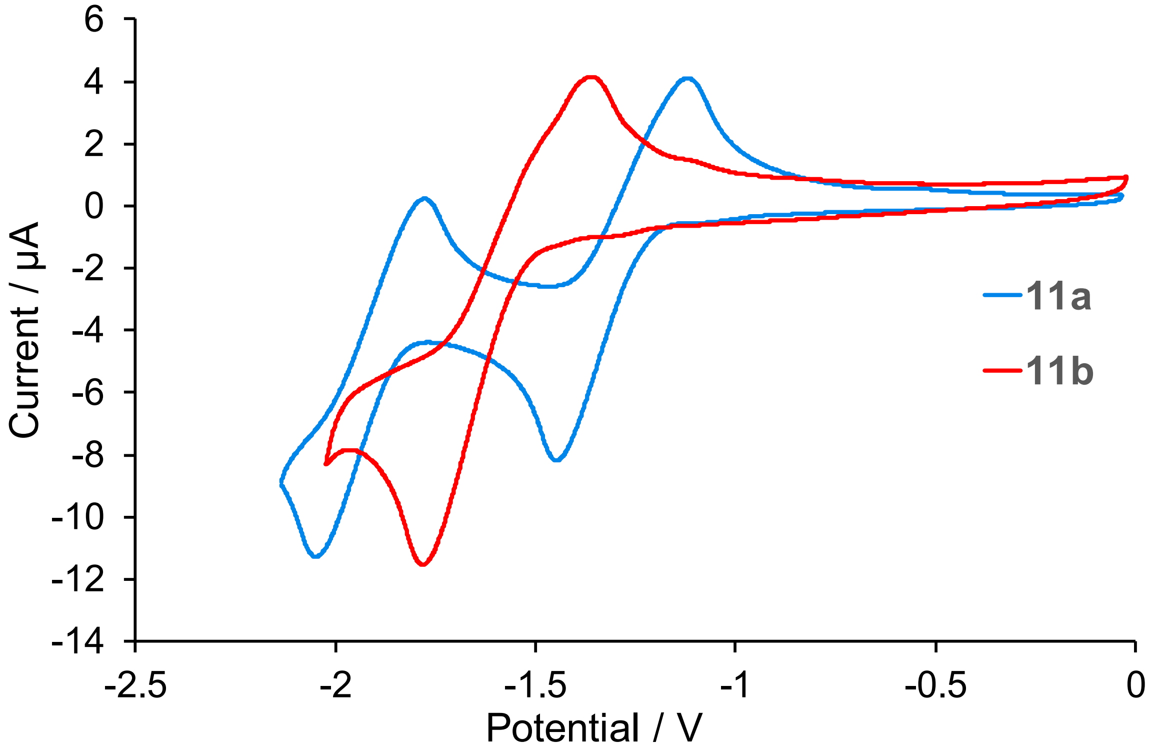

Figure S44. Cyclic Voltammetry diagrams of **11a** and **11b**. Measured in THF (1 mM), with [*n*Bu_4N][PF_6] (0.1 M) as the supporting electrolyte at a scan rate of 100 mV/s.

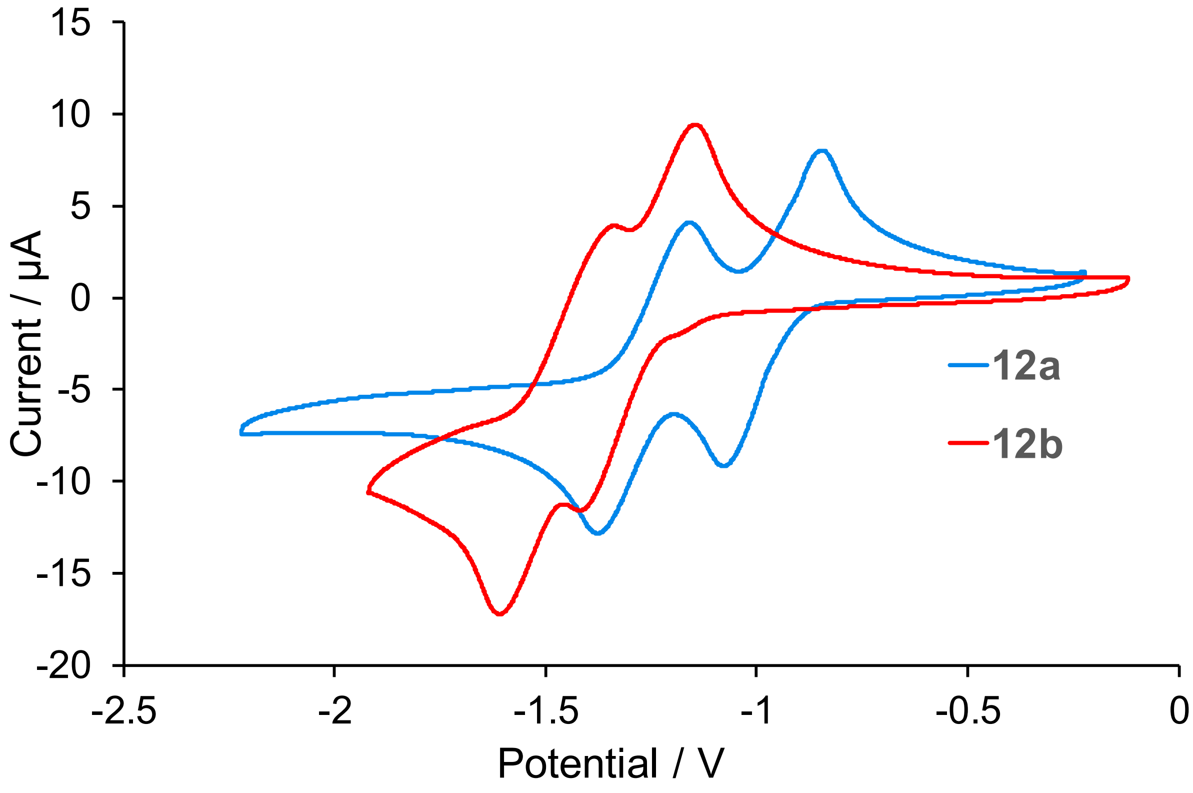

Figure S45. Cyclic Voltammetry diagrams of **12a** and **12b**. Measured in THF (1 mM), with [*n*Bu$_4$N][PF$_6$] (0.1 M) as the supporting electrolyte at a scan rate of 100 mV/s.

S7. NMR Spectra

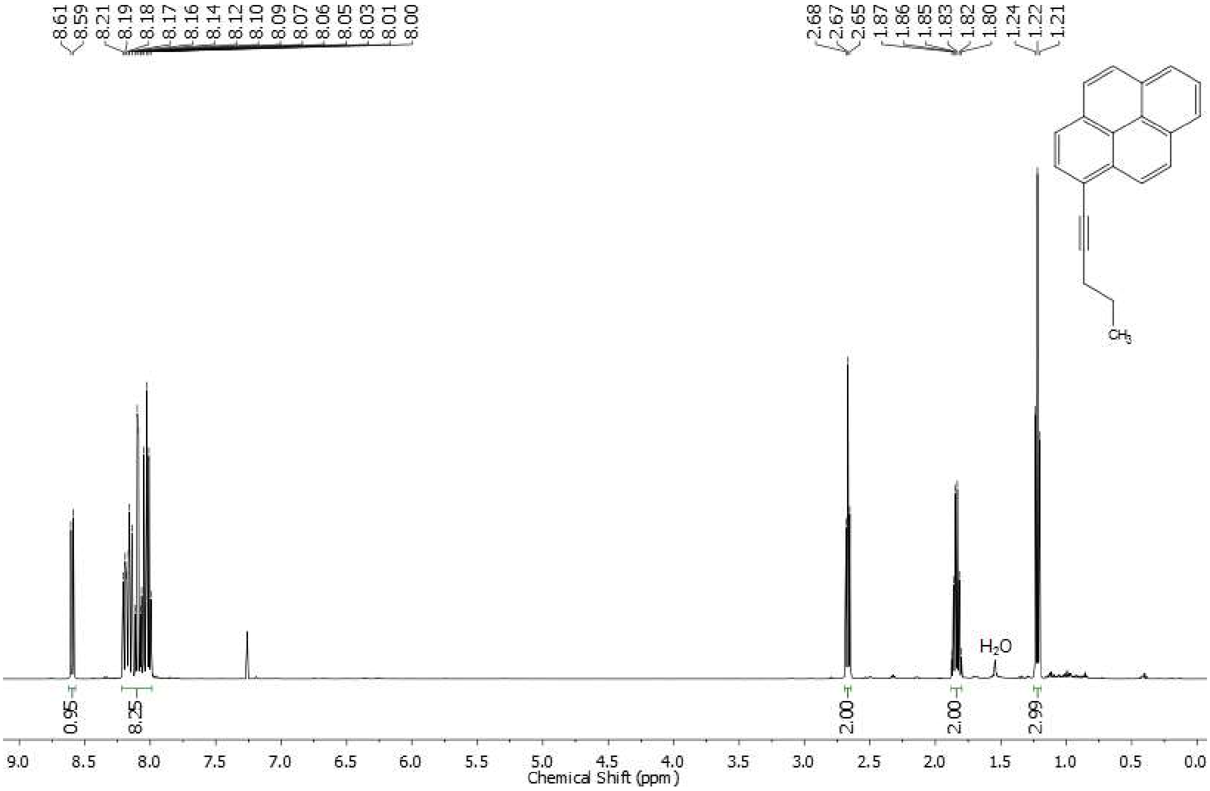

Figure S46. ^{1}H NMR spectrum of 1-(pent-1-yn-1-yl)pyrene in $CDCl_3$.

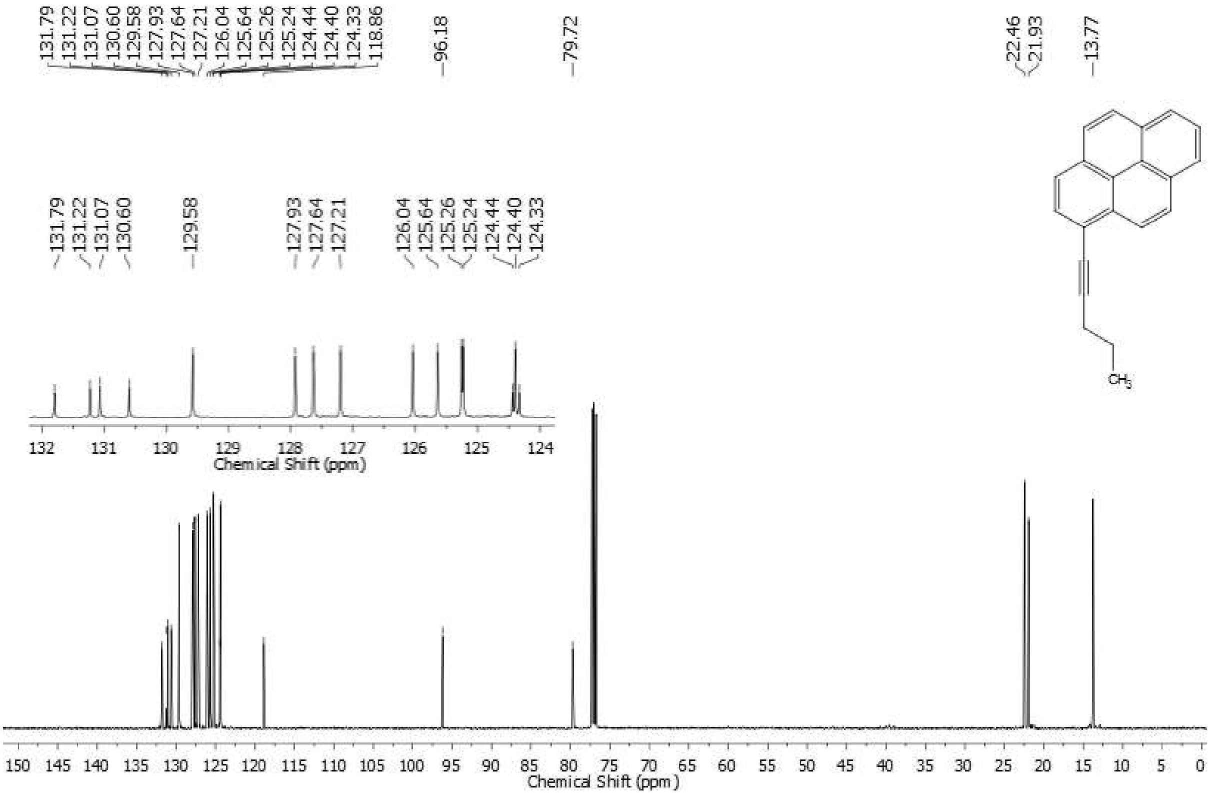

Figure S47. $^{13}C\{^{1}H\}$ NMR spectrum of 1-(pent-1-yn-1-yl)pyrene in $CDCl_3$.

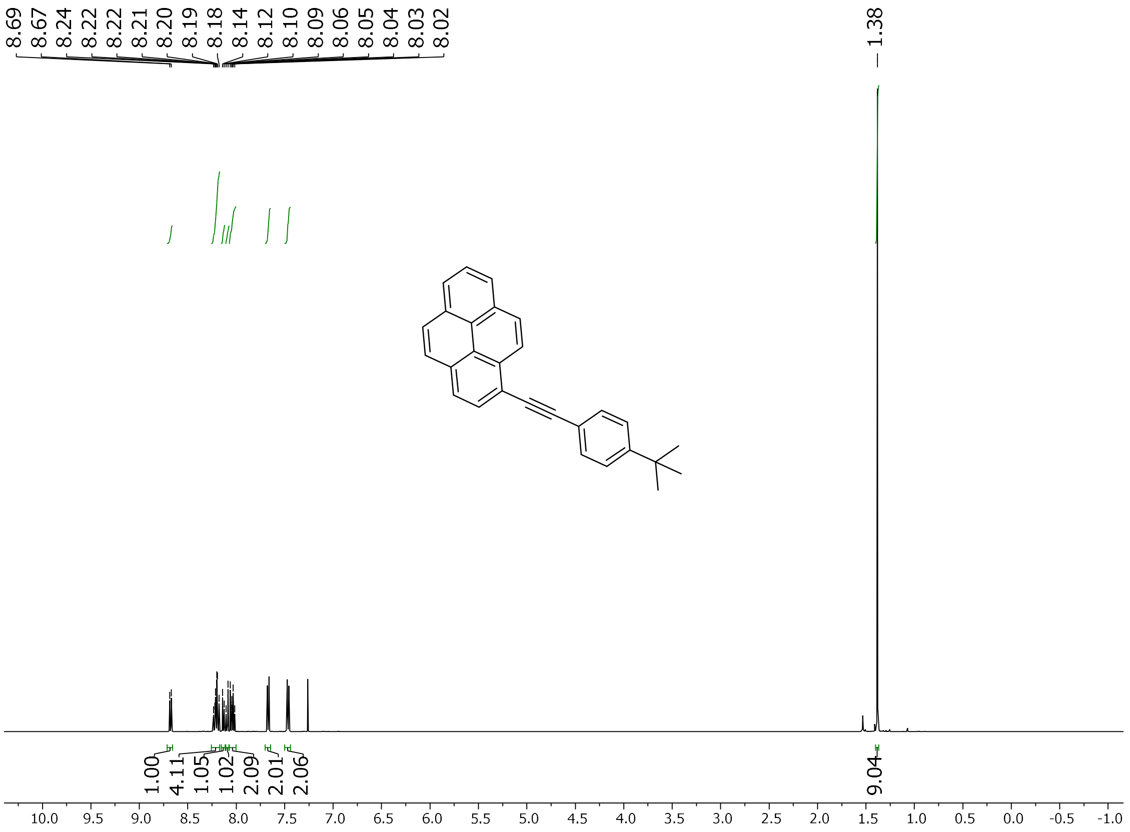

Figure S48. ^{1}H NMR spectrum of 1-((4-(*tert*-butyl)phenyl)ethynyl)pyrene **3** in $CDCl_3$.

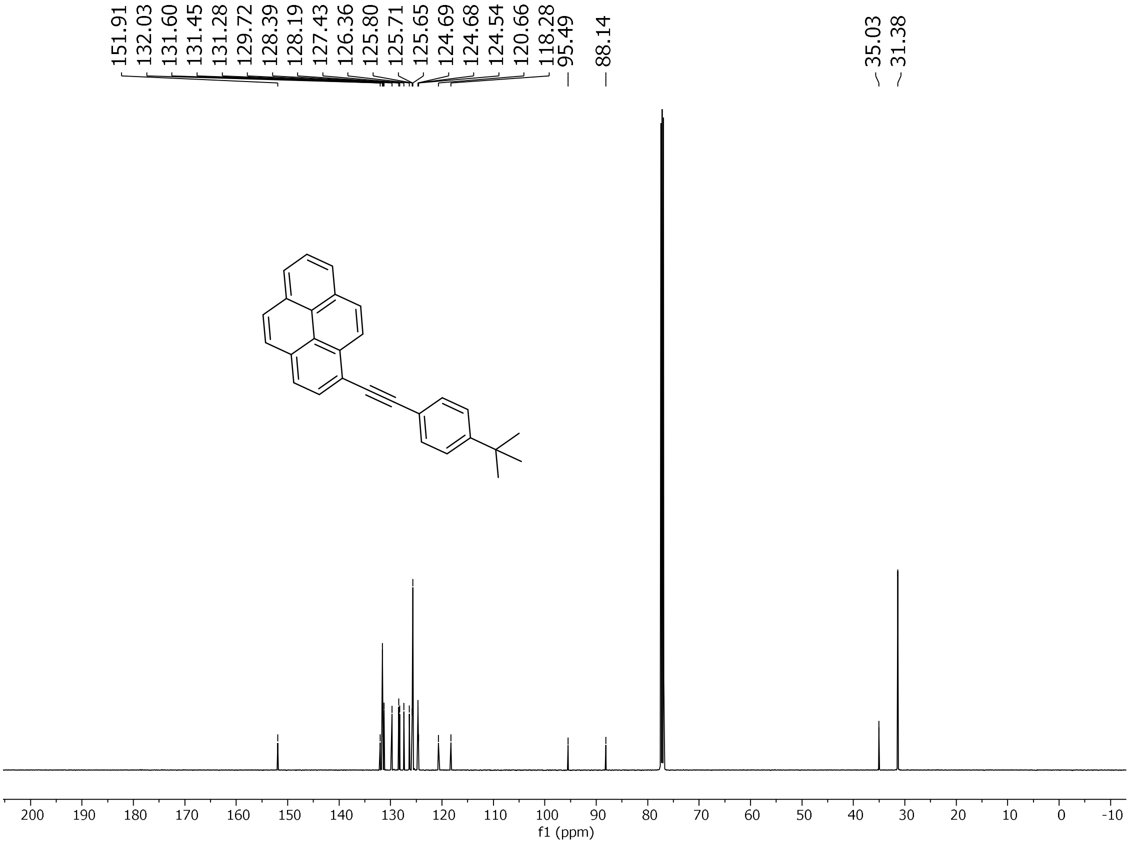

Figure S49. $^{13}C\{^{1}H\}$ NMR spectrum of 1-((4-(*tert*-butyl)phenyl)ethynyl)pyrene **3** in $CDCl_3$.

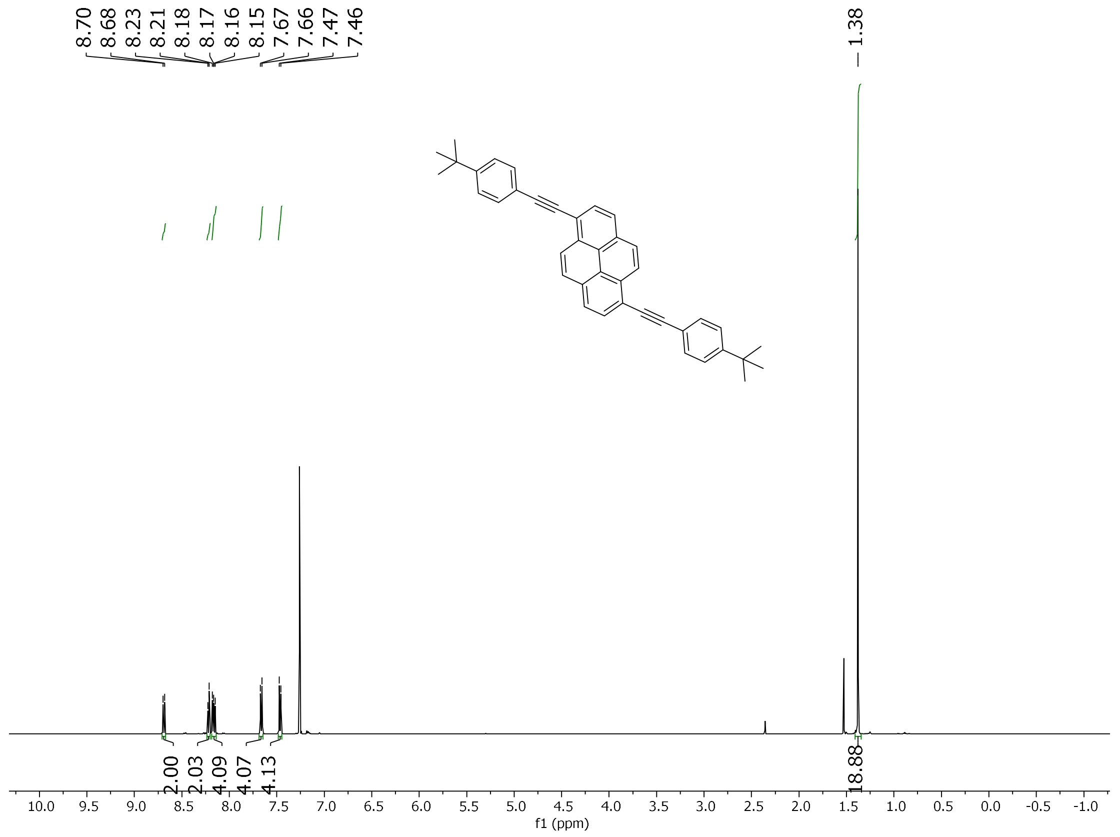

Figure S50. ^{1}H NMR spectrum of 1,6-bis((4-(*tert*-butyl)phenyl)ethynyl)pyrene in $CDCl_3$.

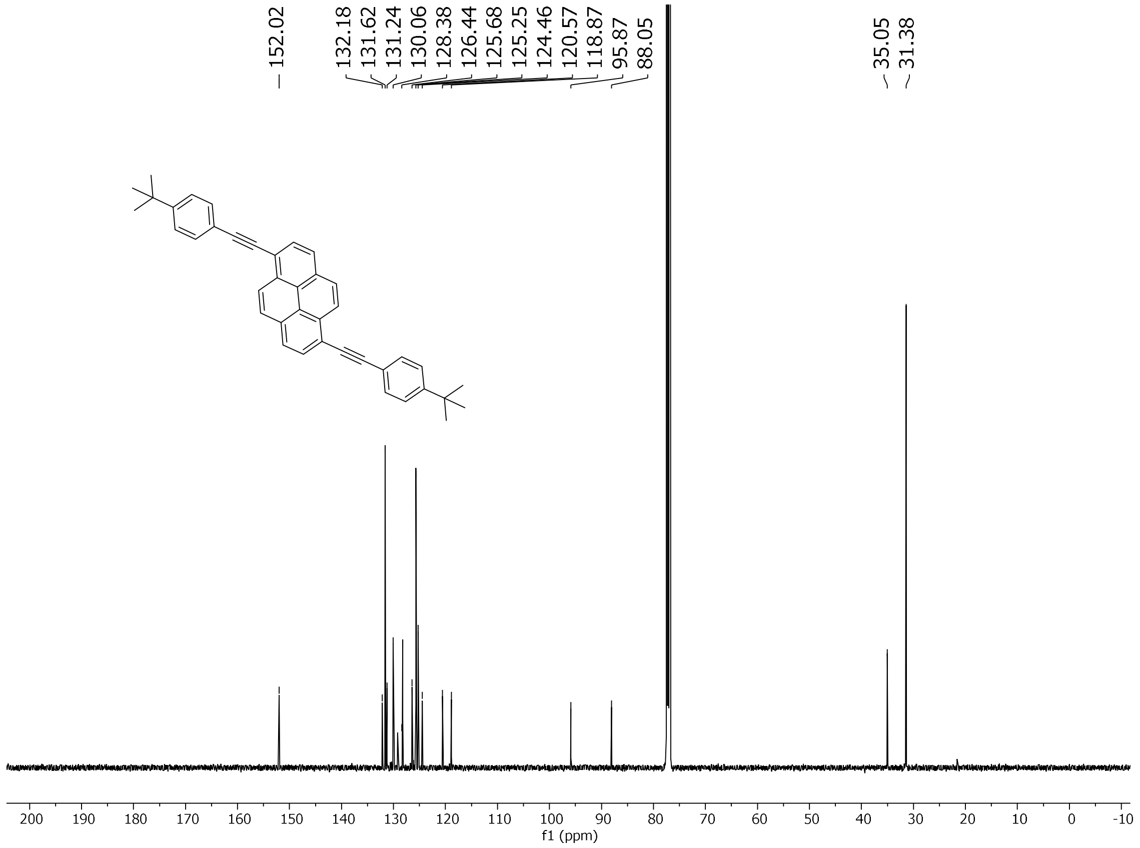

Figure S51. $^{13}C\{^{1}H\}$ NMR spectrum of 1,6-bis((4-(*tert*-butyl)phenyl)ethynyl)pyrene in $CDCl_3$.

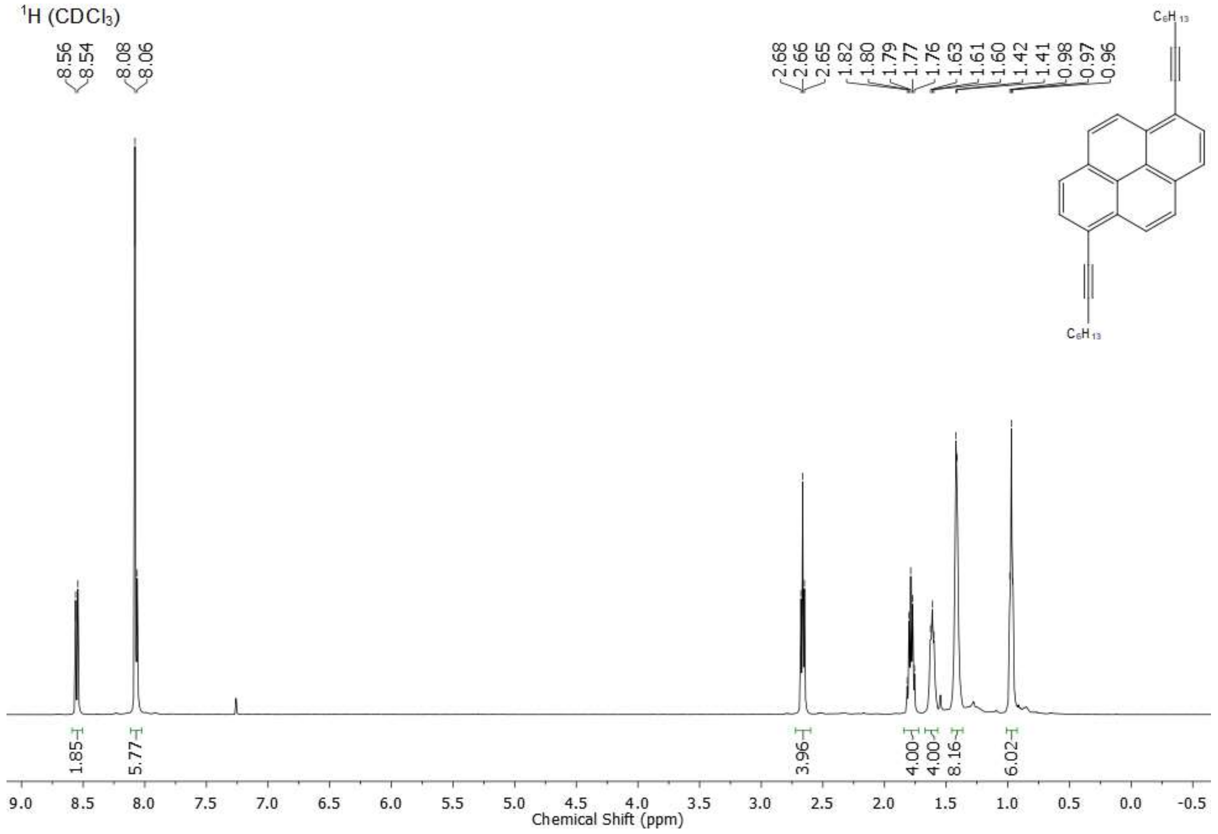

Figure S52. ^{1}H NMR spectrum of 1,6-di(oct-1-yn-1-yl)pyrene in $CDCl_3$.

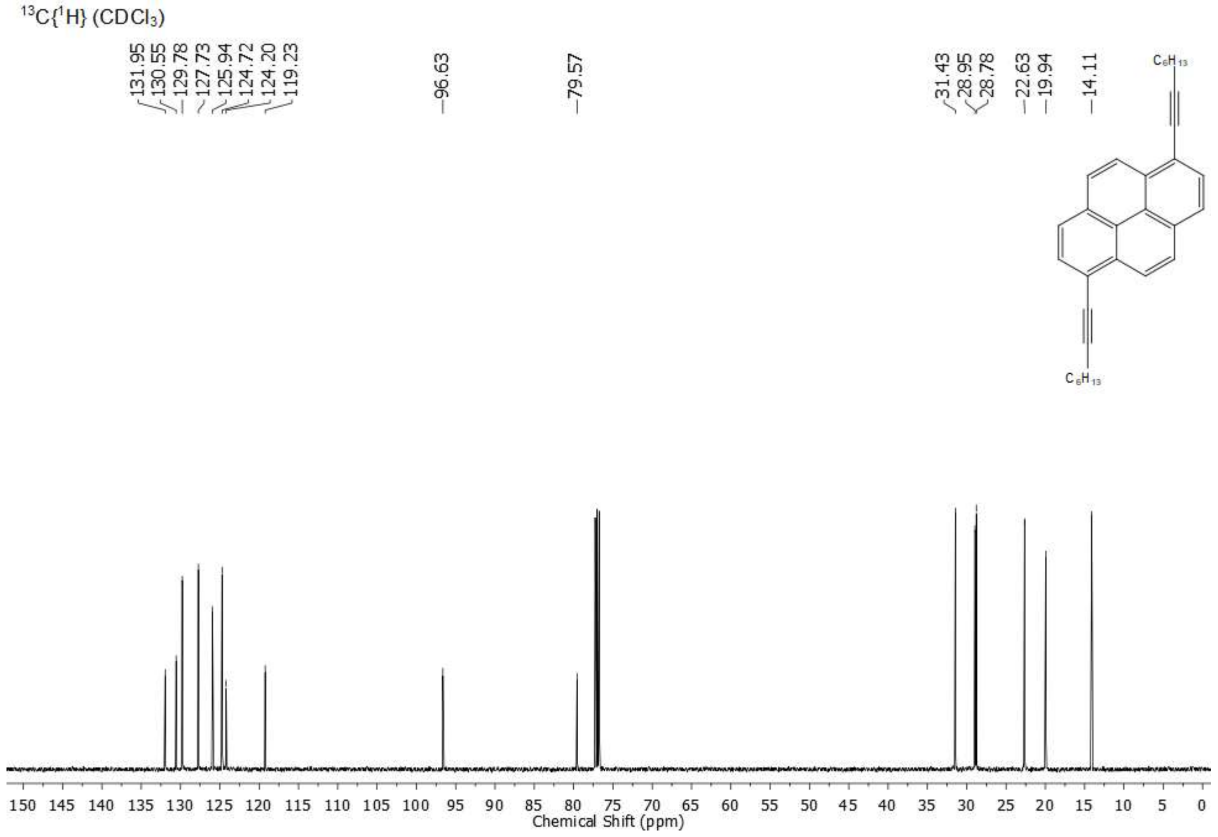

Figure S53. ^{13}C{^{1}H} NMR spectrum of 1,6-di(oct-1-yn-1-yl)pyrene in $CDCl_3$.

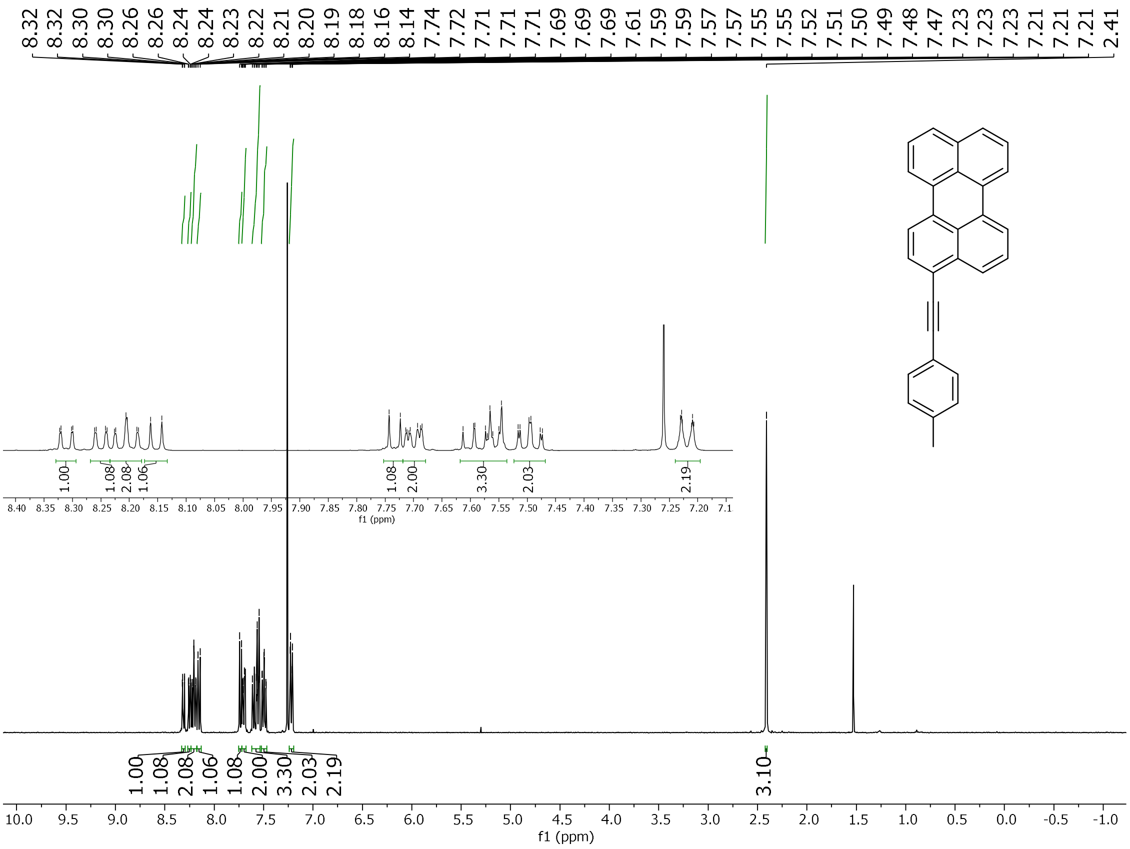

Figure S54. ^{1}H NMR spectrum of 3-(*p*-tolylethynyl)perylene in $CDCl_3$.

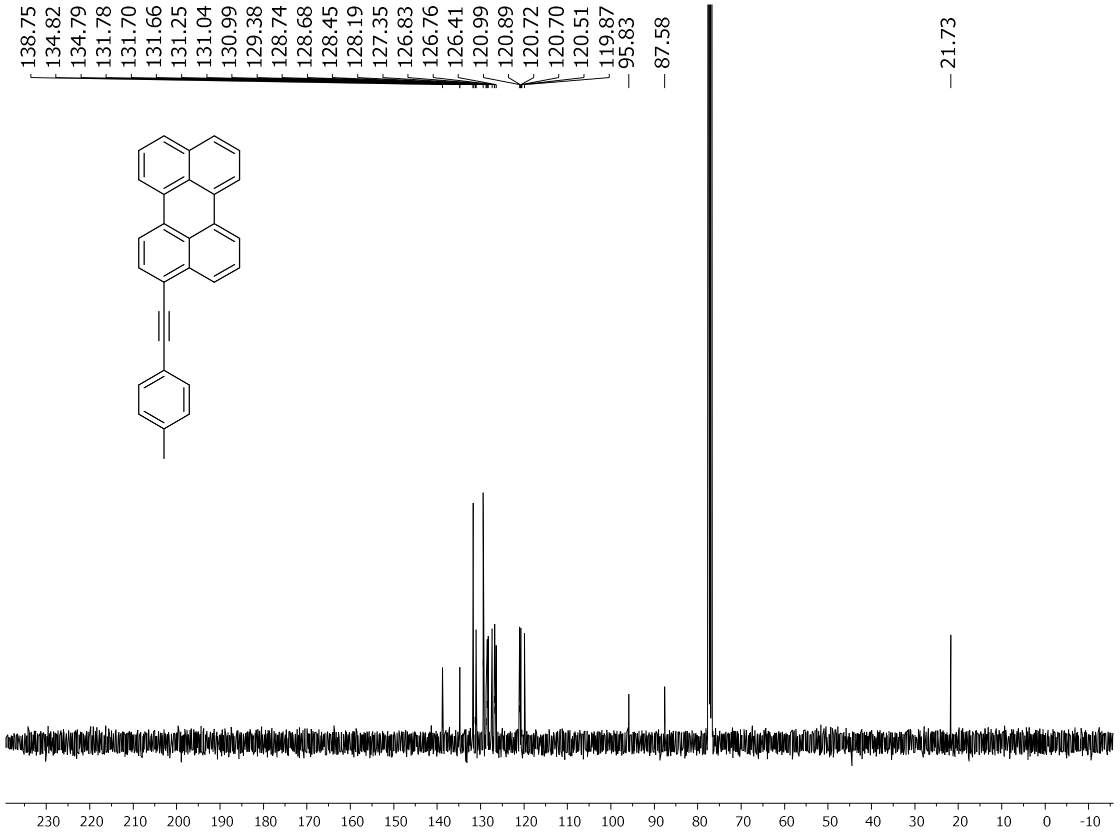

Figure S55. $^{13}C\{^{1}H\}$ NMR spectrum of 3-(*p*-tolylethynyl)perylene in $CDCl_3$.

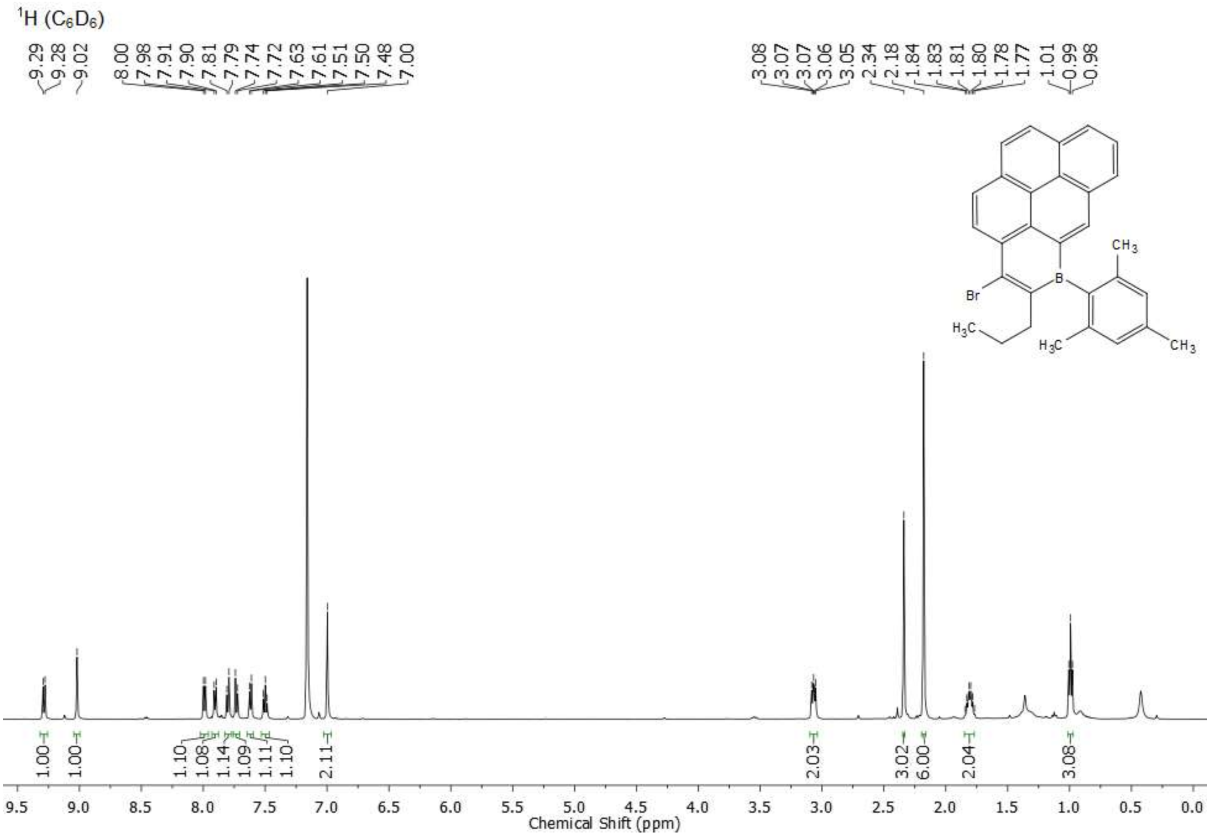

Figure S56. ^{1}H NMR spectrum of **1a**_in C_6D_6.

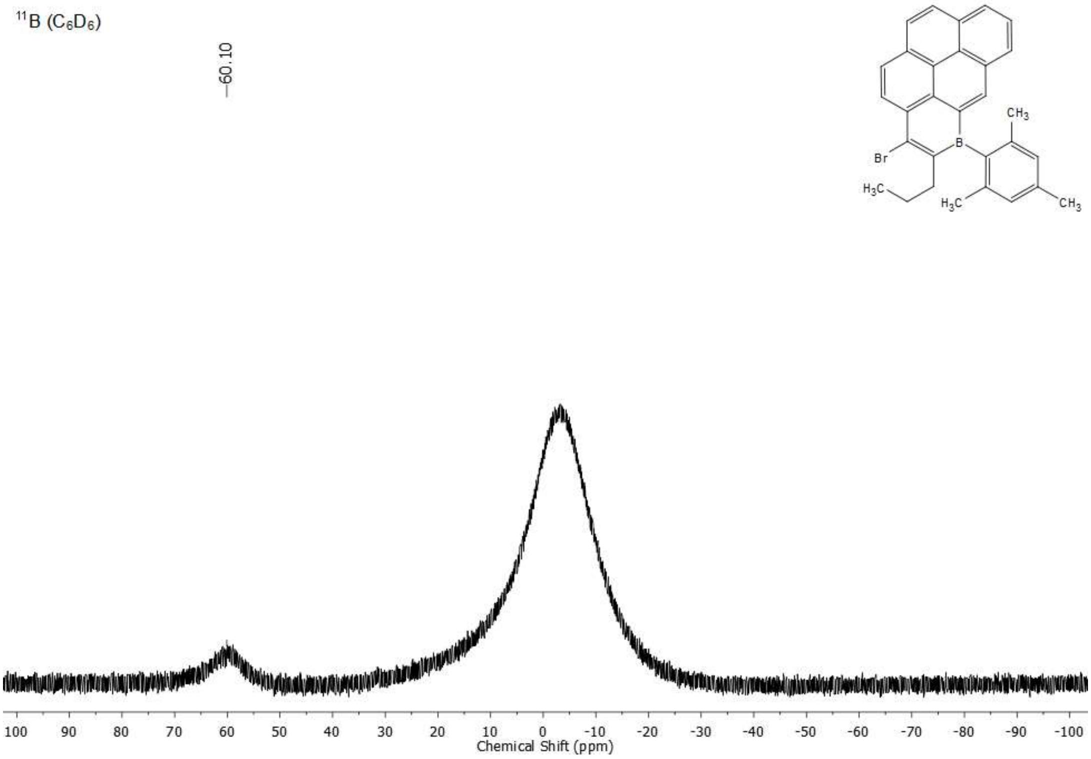

Figure S57. ^{11}B NMR spectrum of **1a**_in C_6D_6.

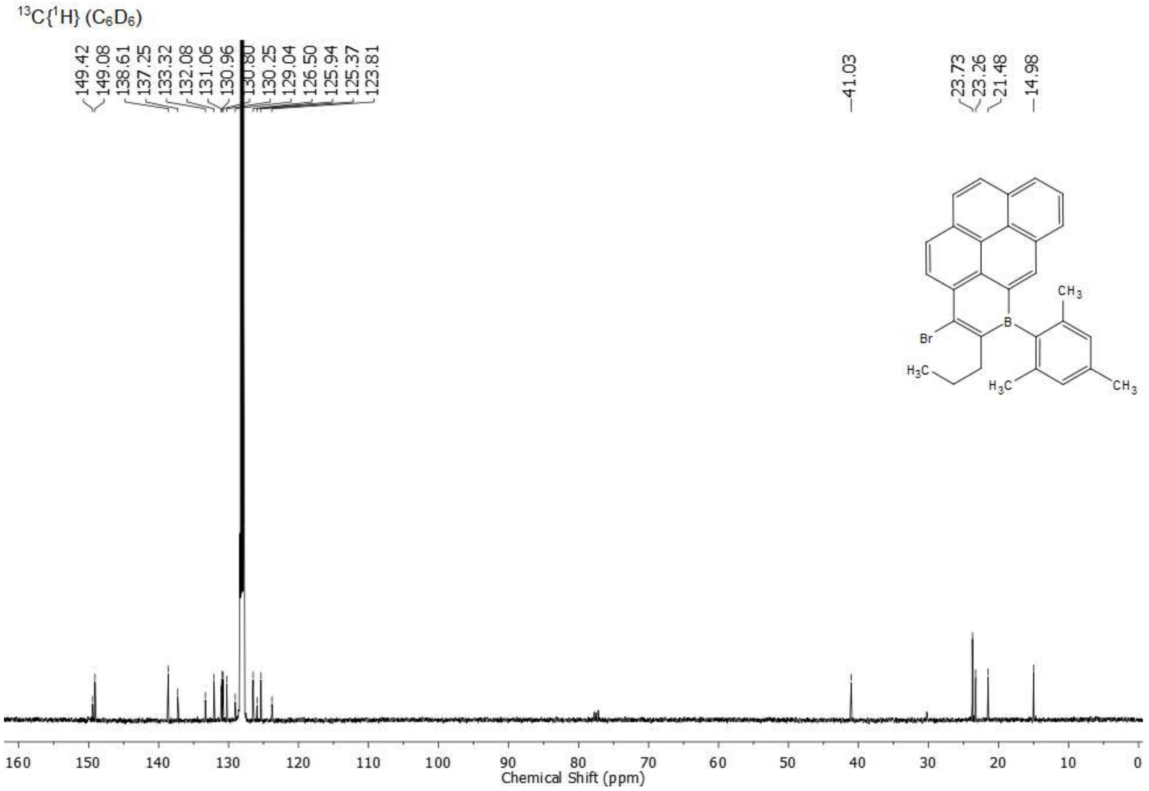

Figure S58. ^{13}C{^{1}H} NMR spectrum of **1a** in C_6D_6.

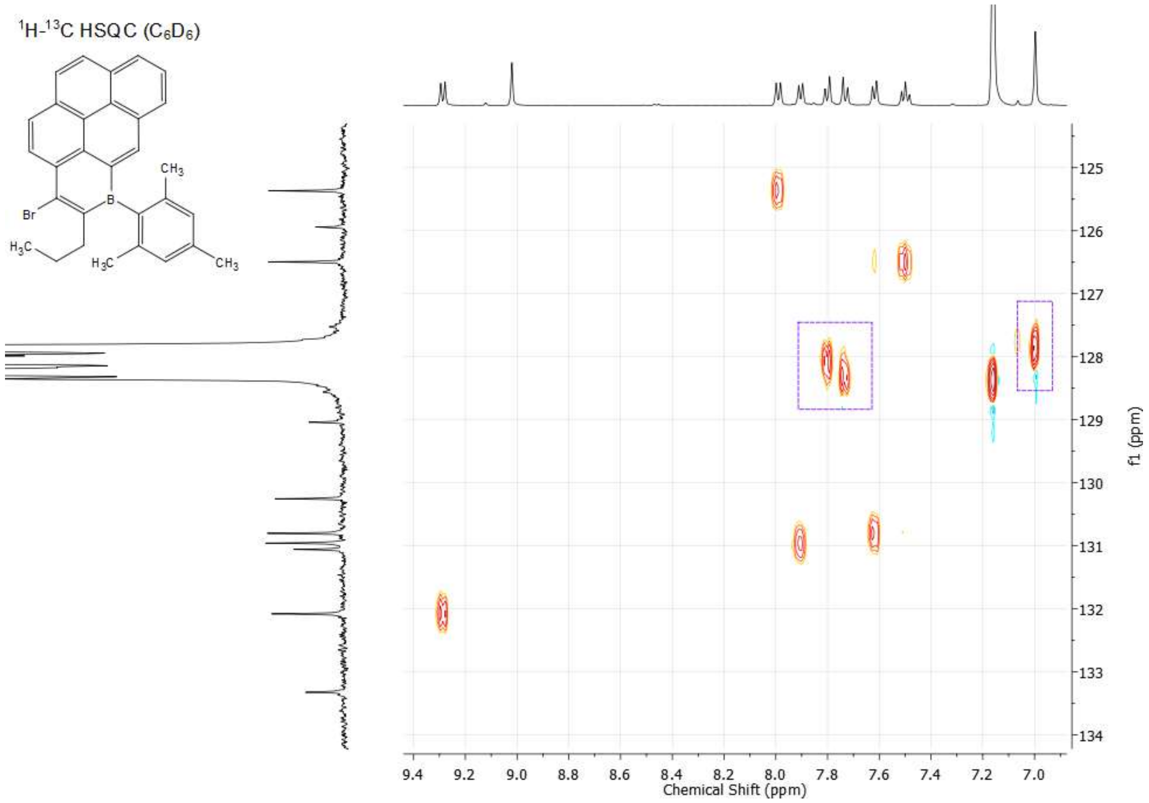

Figure S59. ^{1}H-^{13}C HSQC NMR spectrum of **1a** in C_6D_6. Three carbon resonances coincide with the benzene resonance.

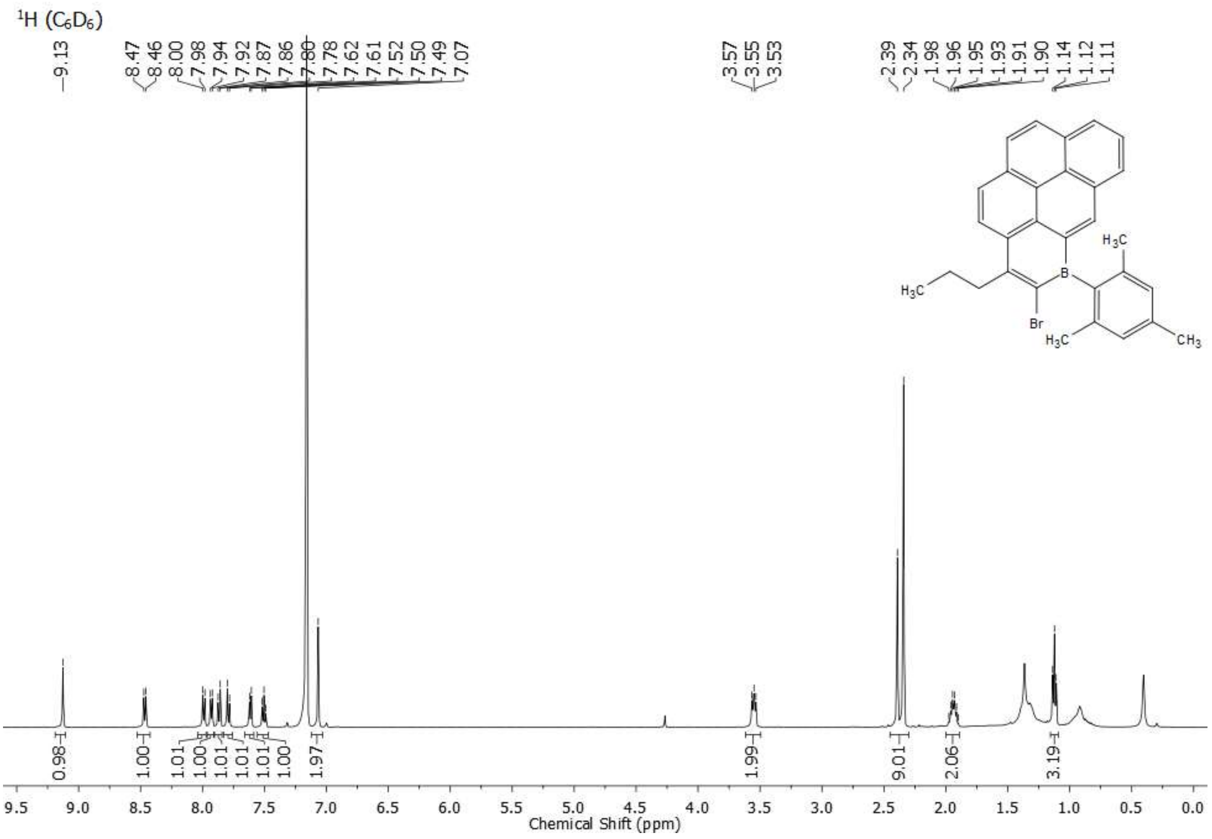

Figure S60. ^{1}H NMR spectrum of **1b**_in C_6D_6.

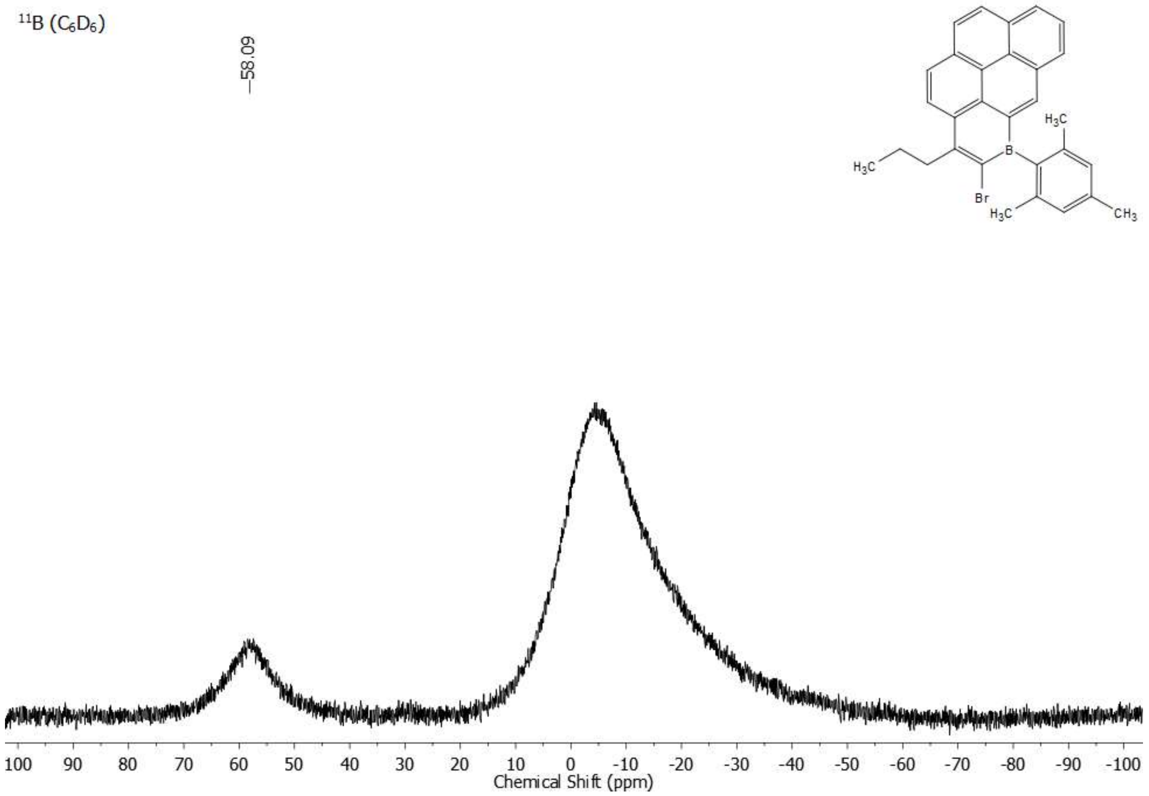

Figure S61. ^{11}B NMR spectrum of **1b** in C_6D_6.

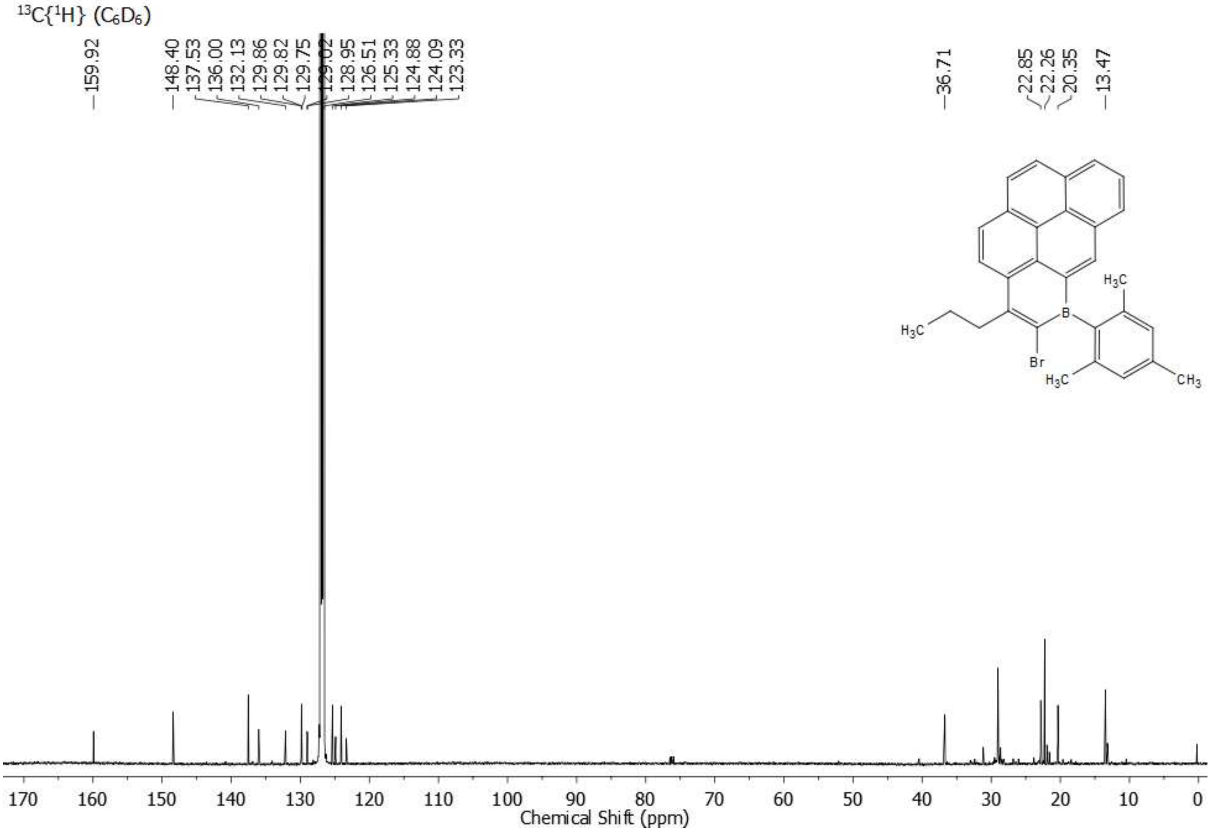

Figure S62. $^{13}C\{^{1}H\}$ NMR spectrum of **1b** in C_6D_6.

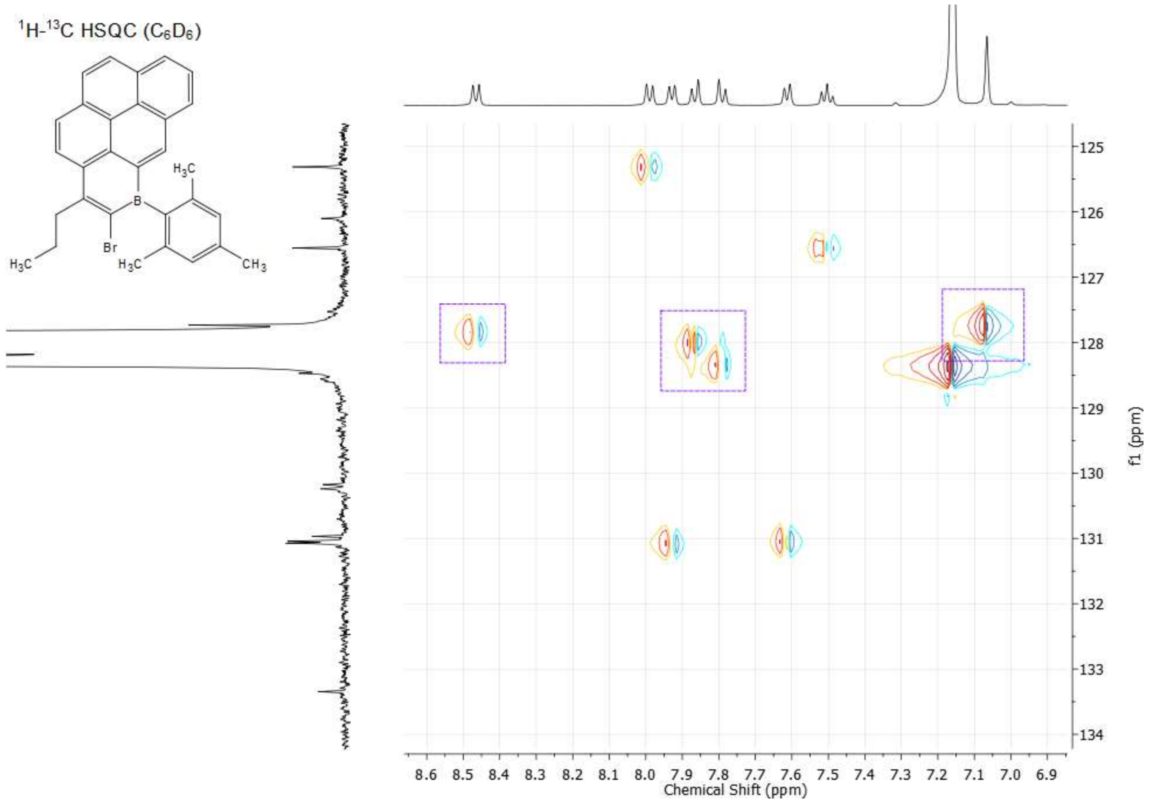

Figure S63. ^{1}H-^{13}C HSQC NMR spectrum of **1b** in C_6D_6. Four carbon resonances coincide with the benzene resonance.

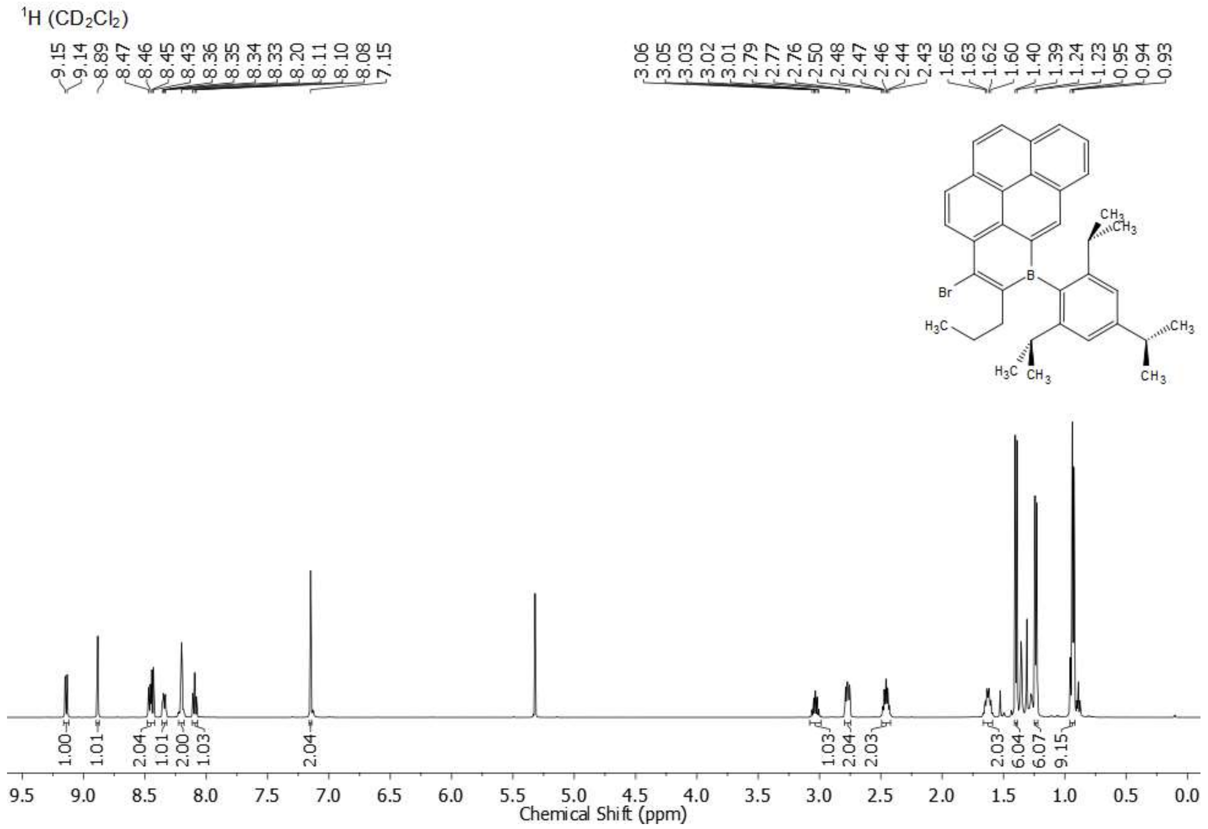

Figure S64. ^{1}H NMR spectrum of **1c** in CD_2Cl_2.

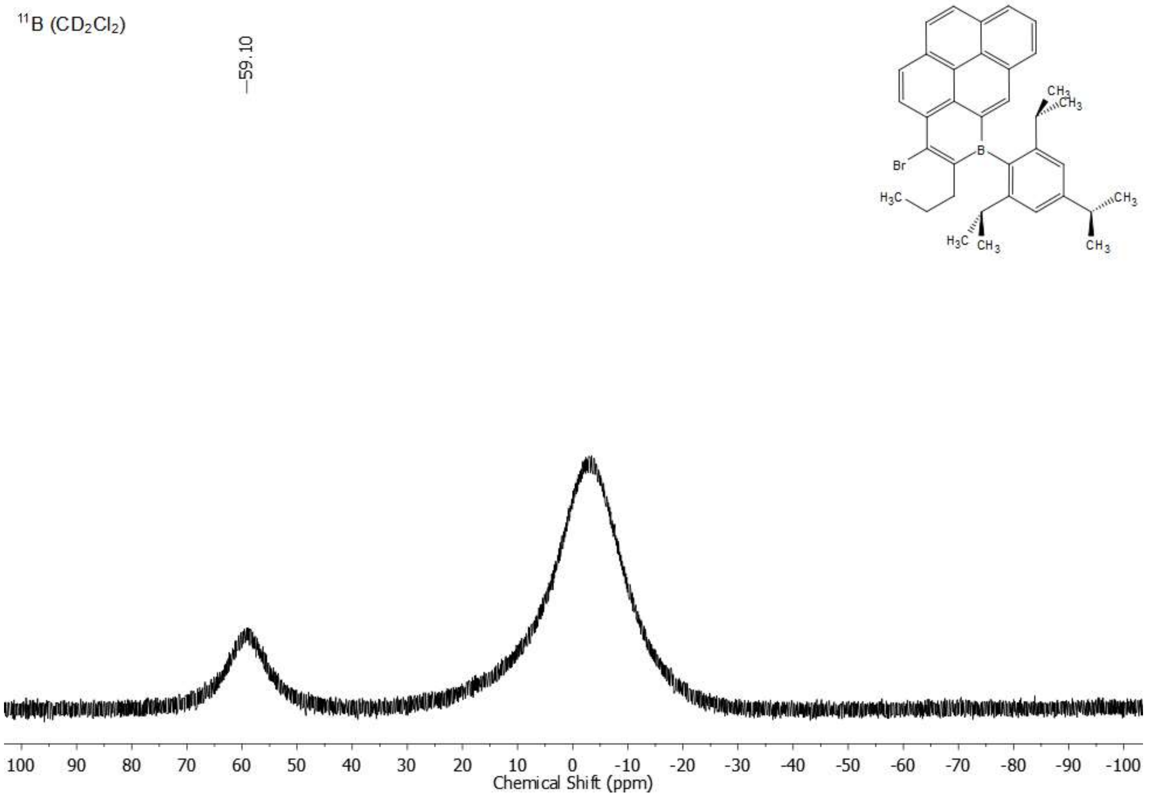

Figure S65. ^{11}B NMR spectrum of **1c** in CD_2Cl_2.

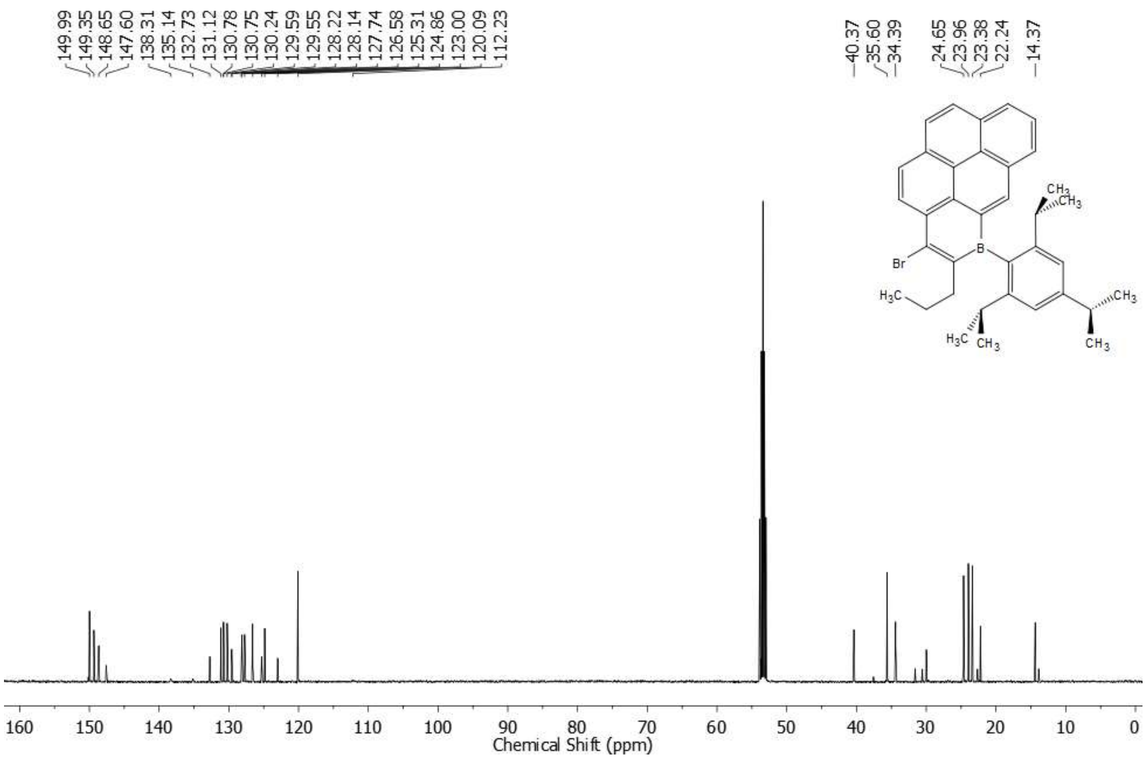

Figure S66. $^{13}C\{^{1}H\}$ NMR spectrum of **1c** in CD_2Cl_2.

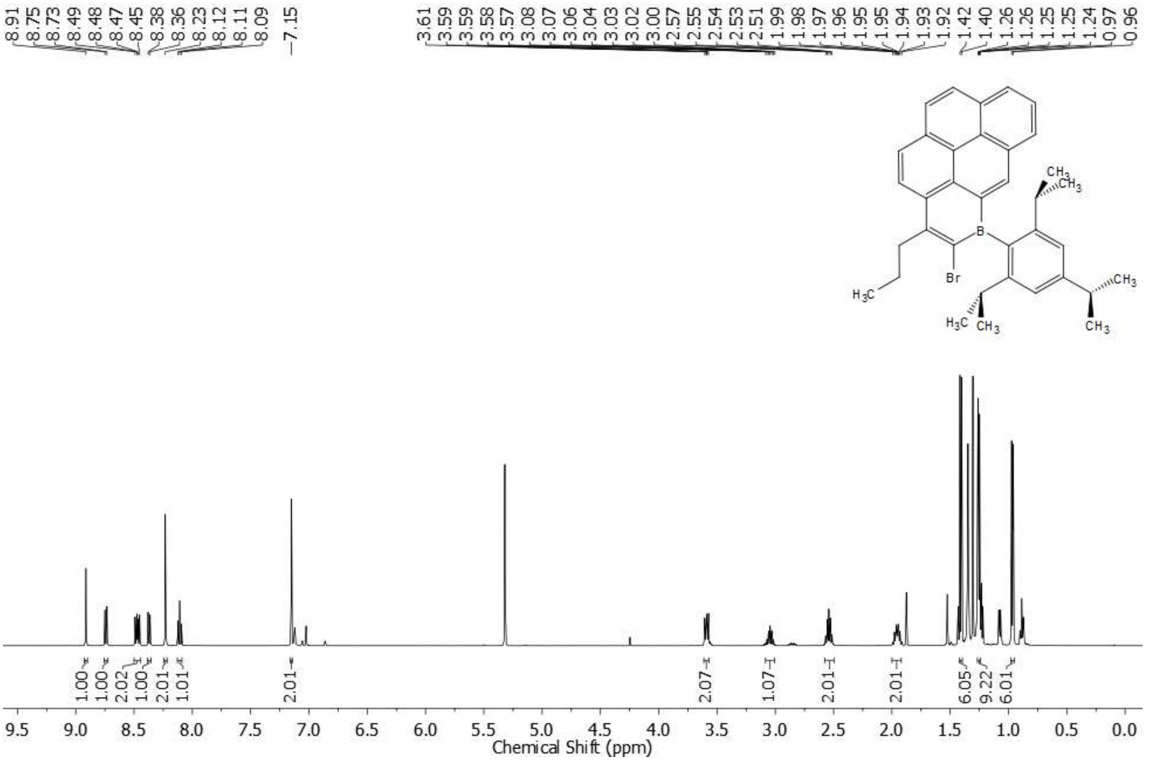

Figure S67. ^{1}H NMR spectrum of **1d** in CD_2Cl_2.

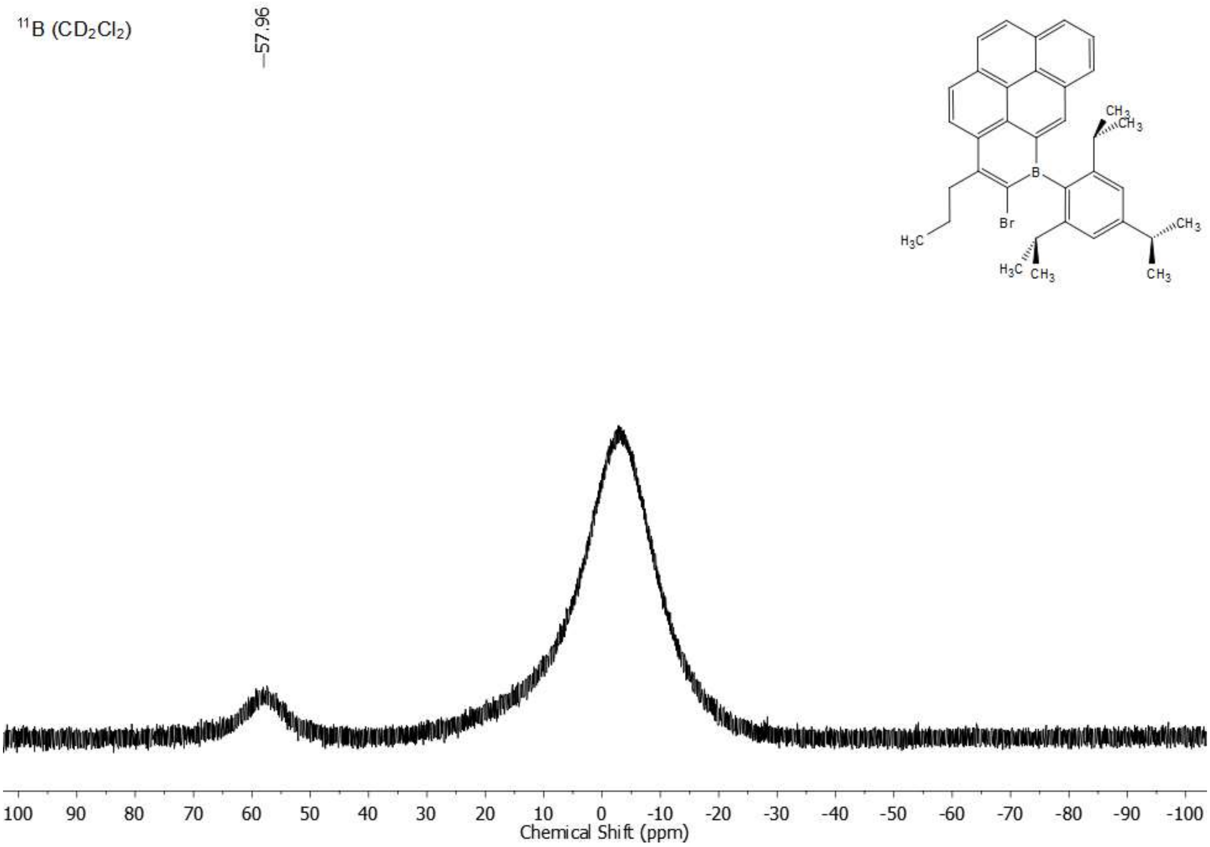

Figure S68. ^{11}B NMR spectrum of **1d** in CD_2Cl_2.

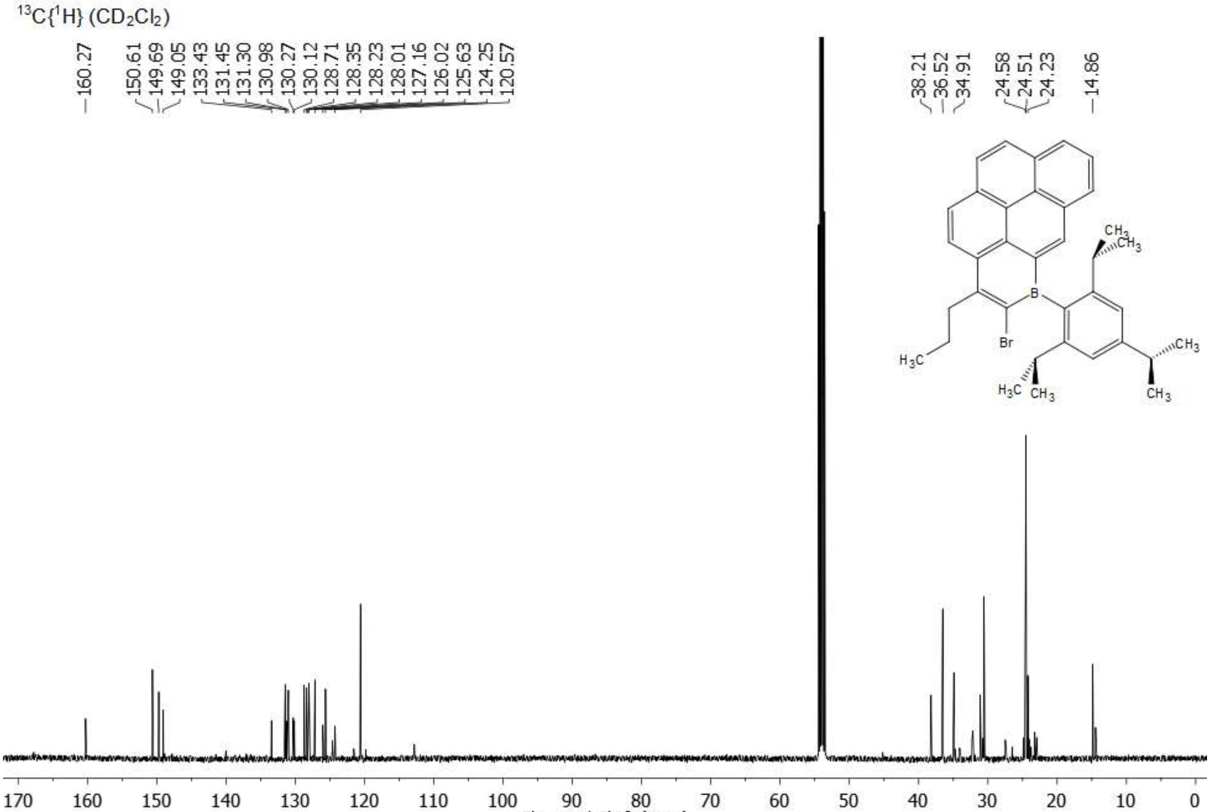

Figure S69. $^{13}C\{^1H\}$ NMR spectrum of **1d** in CD_2Cl_2.

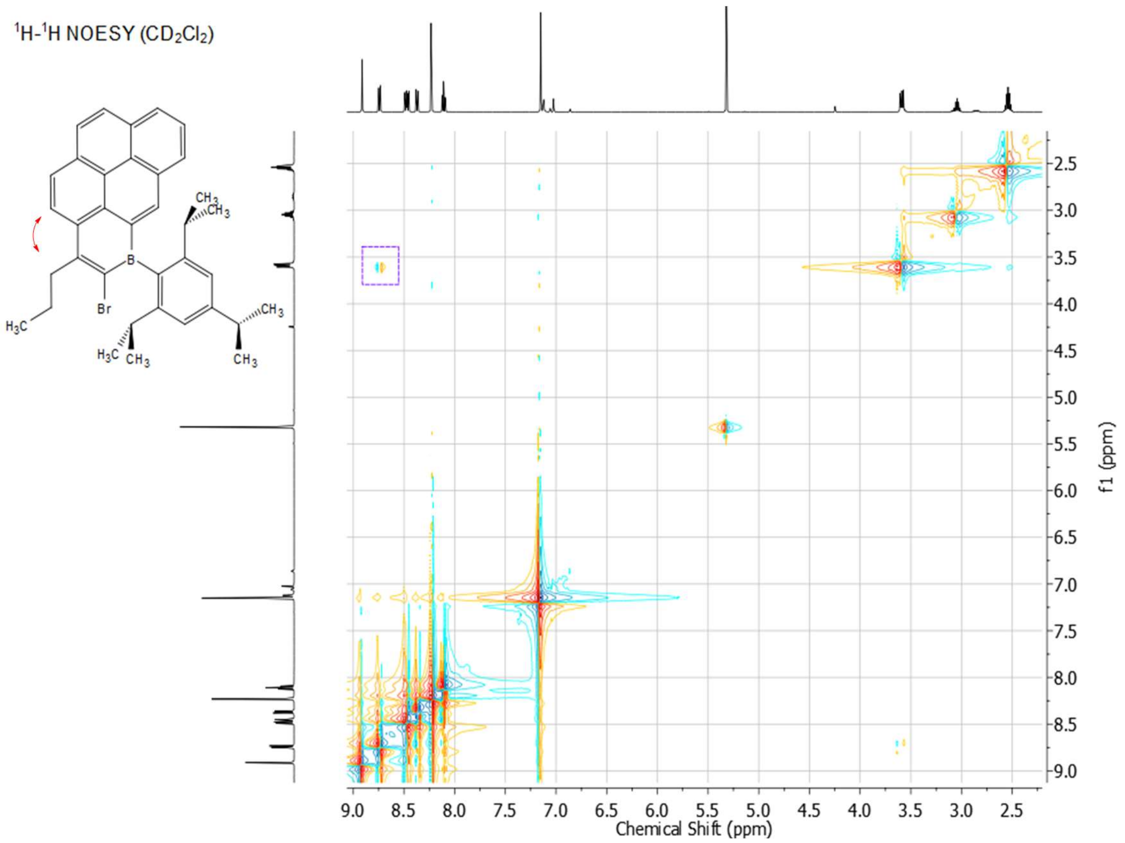

Figure S70. ^{1}H-^{1}H NOESY NMR spectrum of **1d** in CD_2Cl_2.

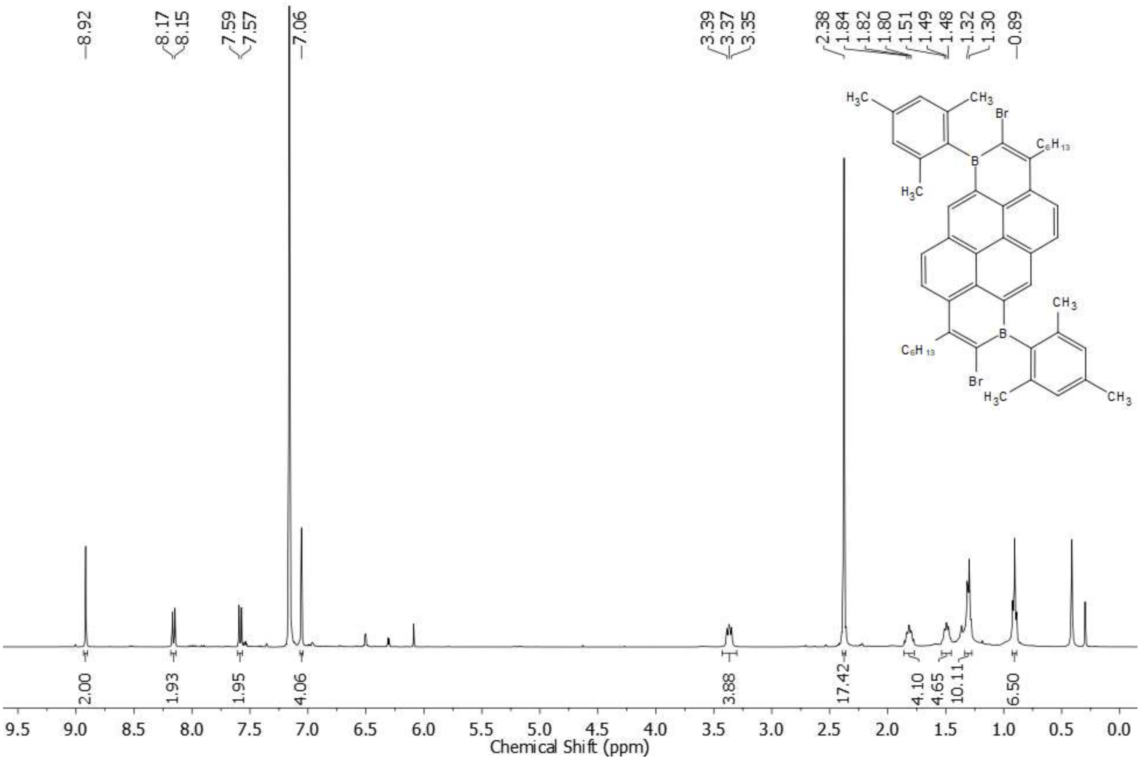

Figure S71. ^{1}H NMR spectrum of **2a** in C_6D_6.

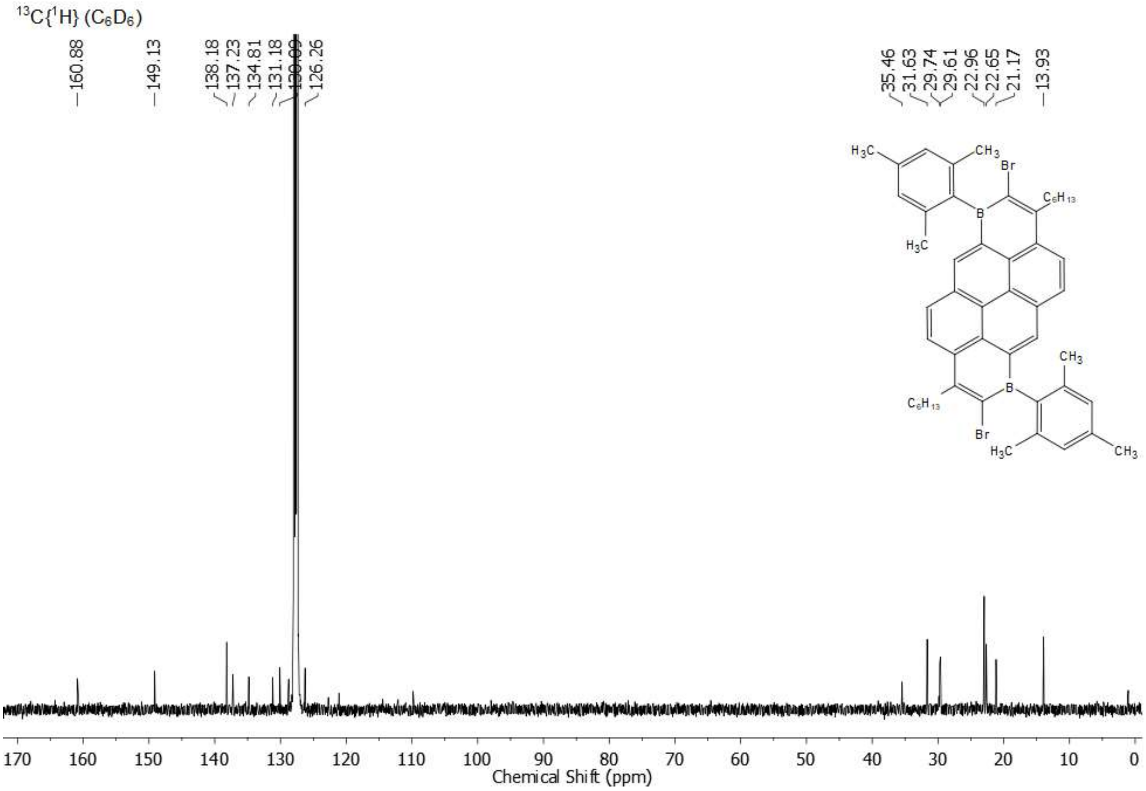

Figure S72. $^{13}C\{^1H\}$ NMR spectrum of **2a** in C_6D_6.

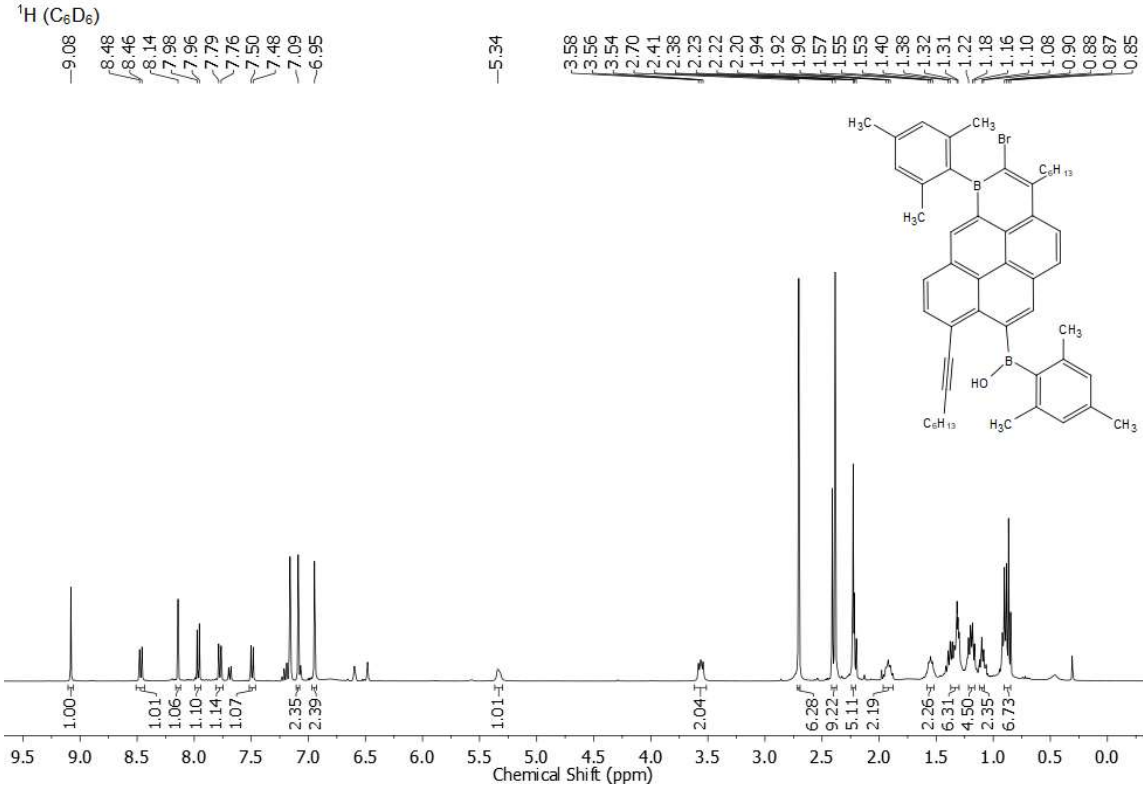

Figure S73. ^1H NMR spectrum of **2b** in C_6D_6.

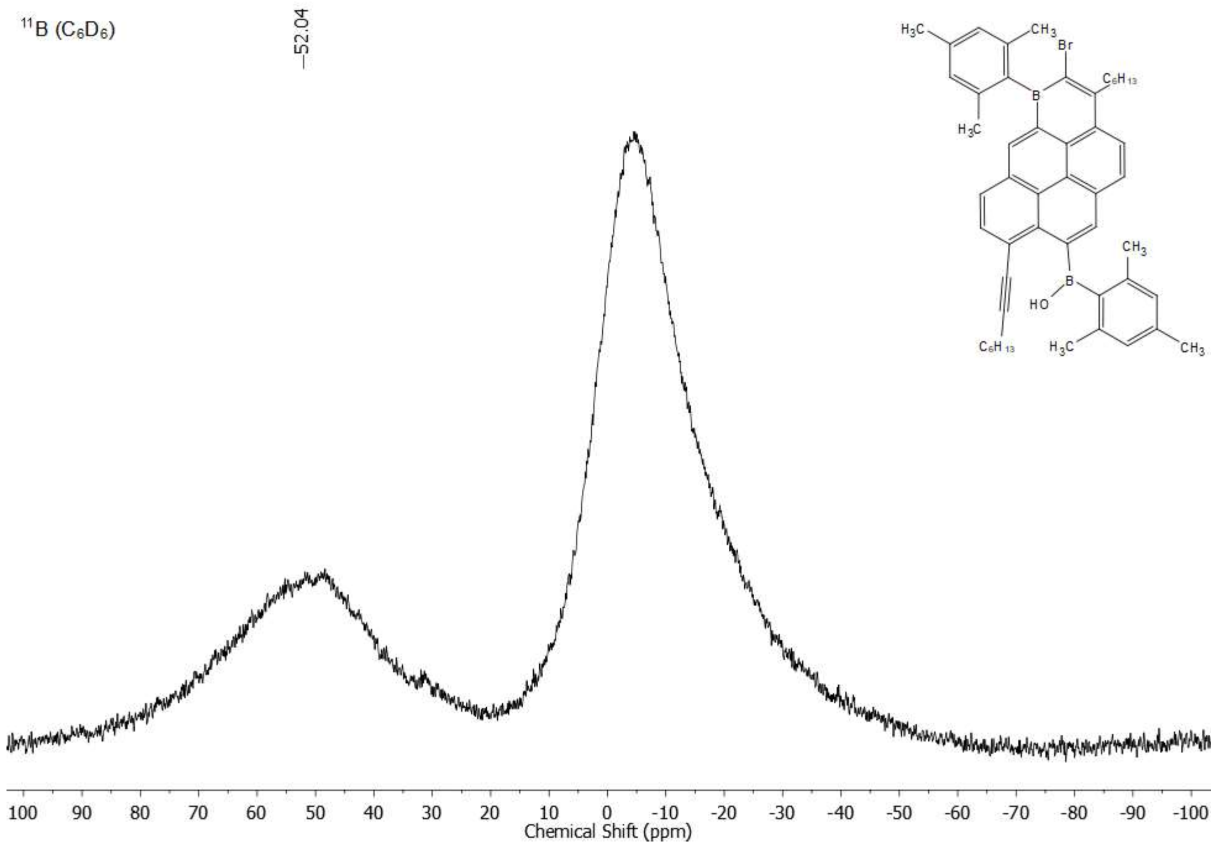

Figure S74. ^{11}B NMR spectrum of **2b** in C_6D_6.

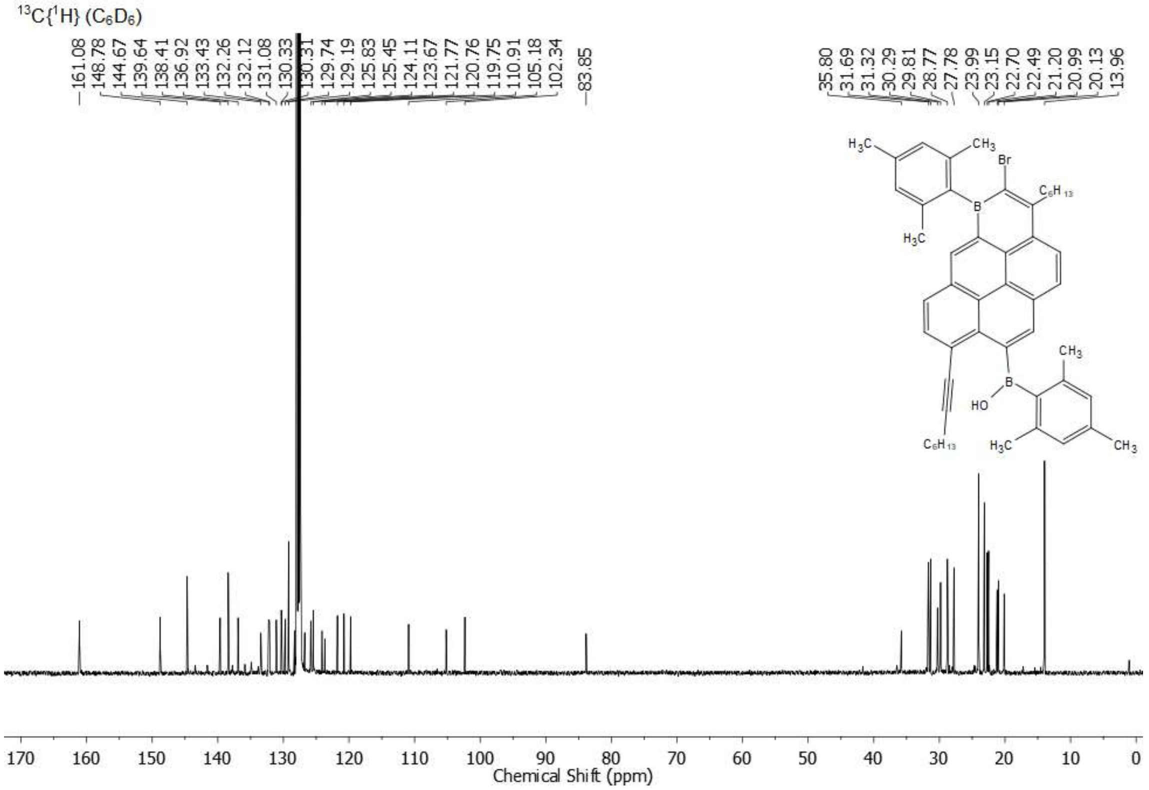

Figure S75. $^{13}C\{^{1}H\}$ NMR spectrum of **2b** in C_6D_6.

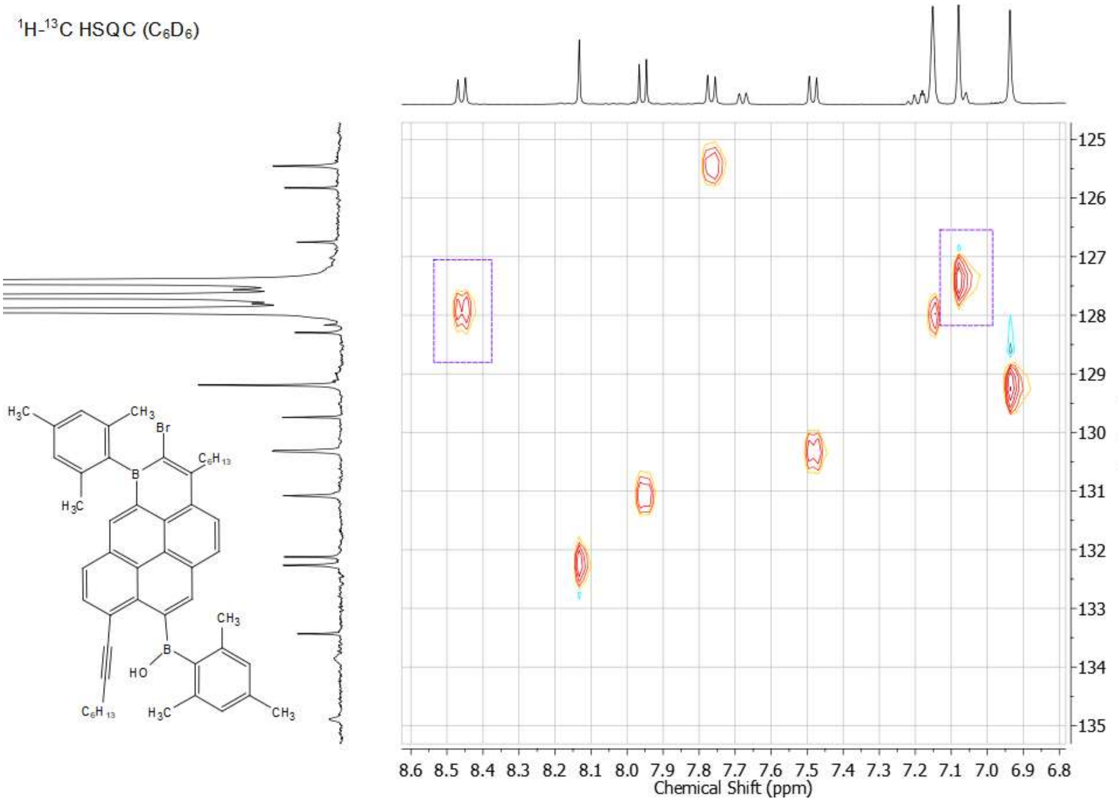

Figure S76. ^{1}H-^{13}C HSQC NMR spectrum of **2b** in C_6D_6. Two carbon resonances coincide with the benzene resonance.

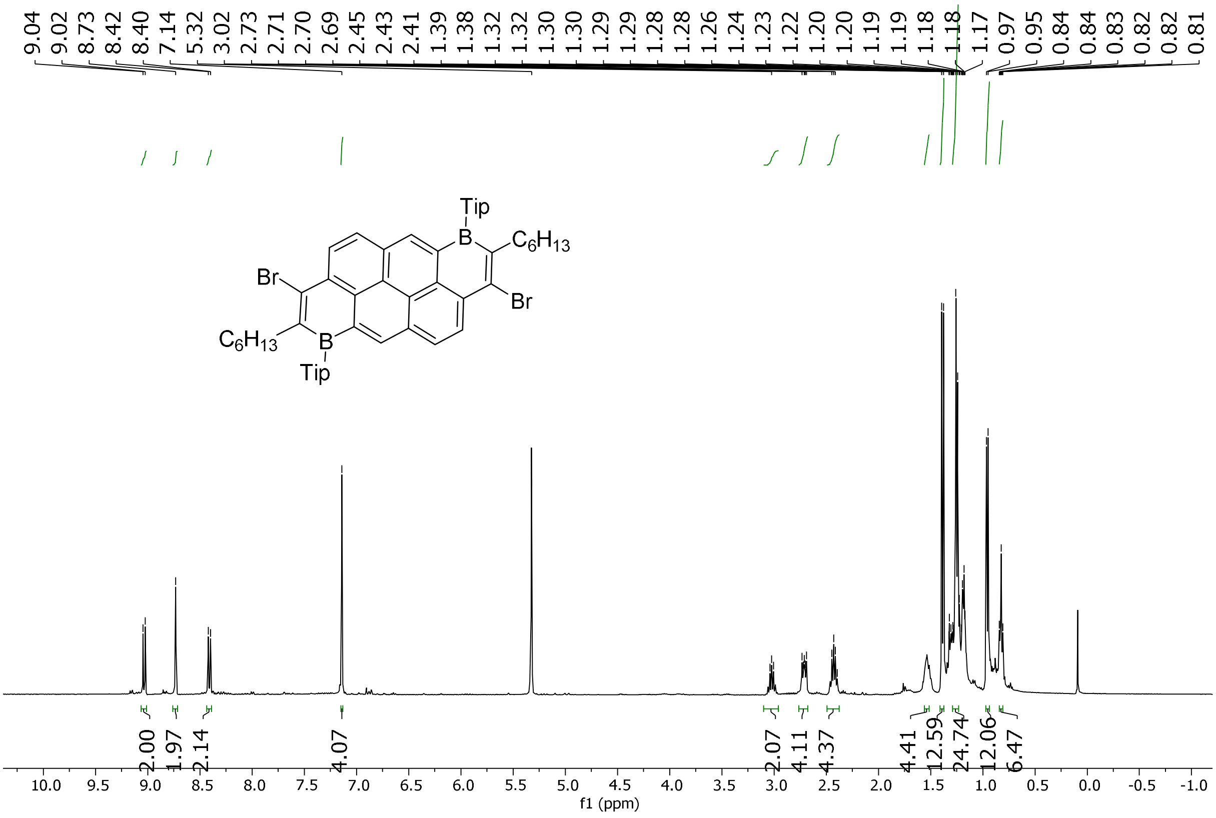

Figure S77. ^{1}H NMR spectrum of **2c** in CD_2Cl_2.

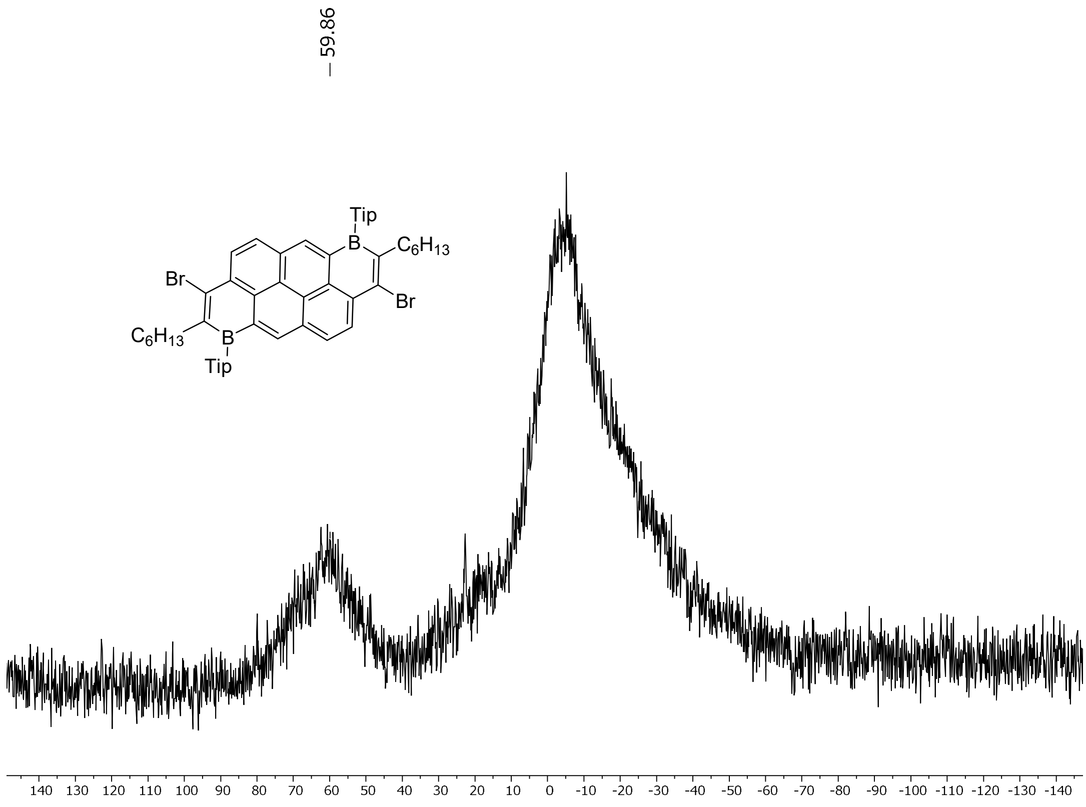

Figure S78. ^{11}B NMR spectrum of **2c** in CD_2Cl_2.

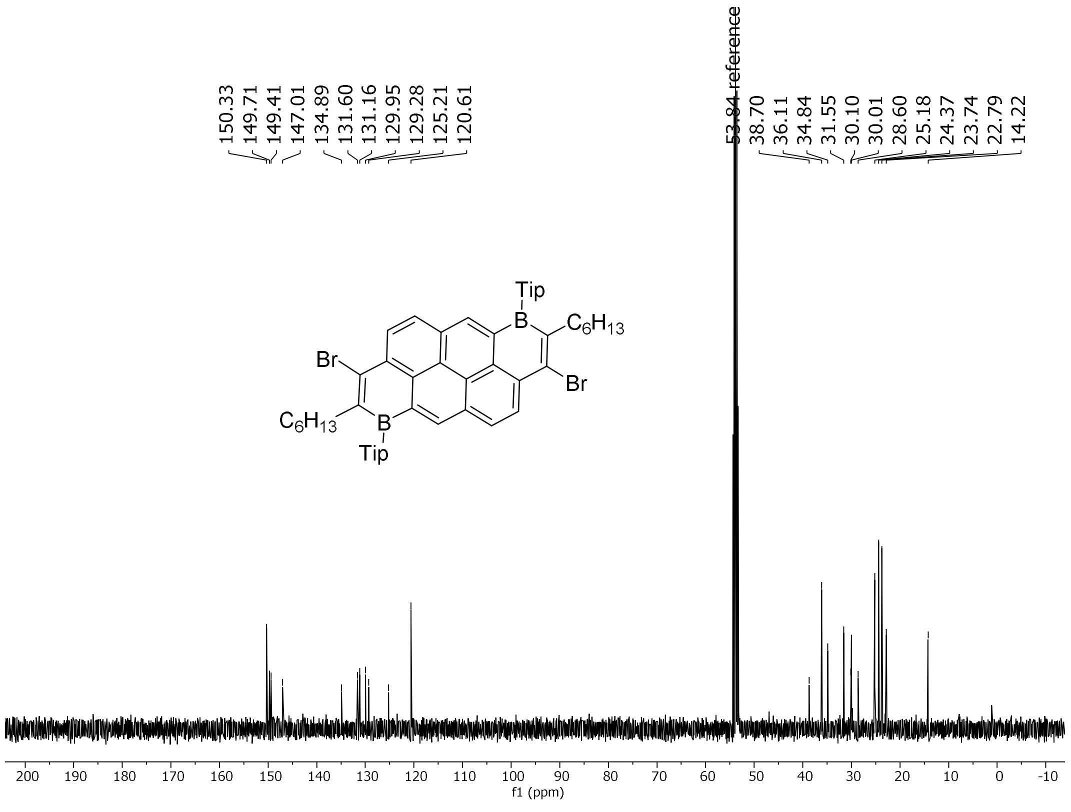

Figure S79. $^{13}C\{^1H\}$ NMR spectrum of **2c** in CD_2Cl_2.

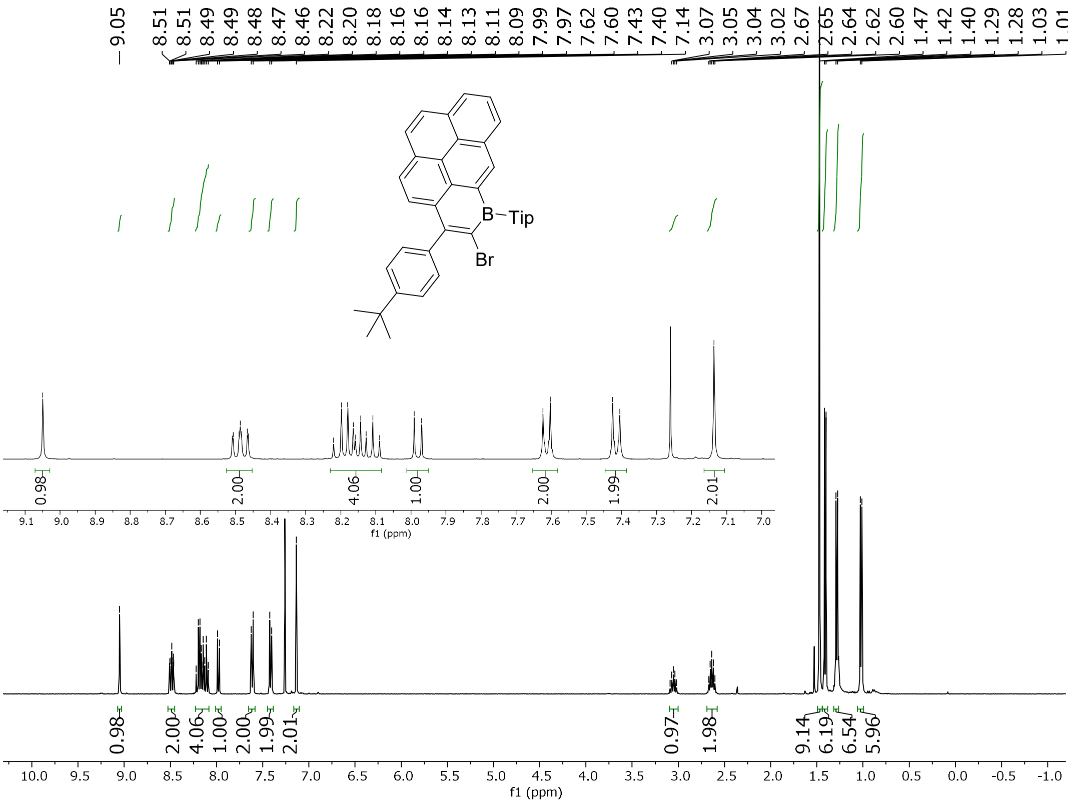

Figure S80. ^{1}H NMR spectrum of **3a** in $CDCl_3$.

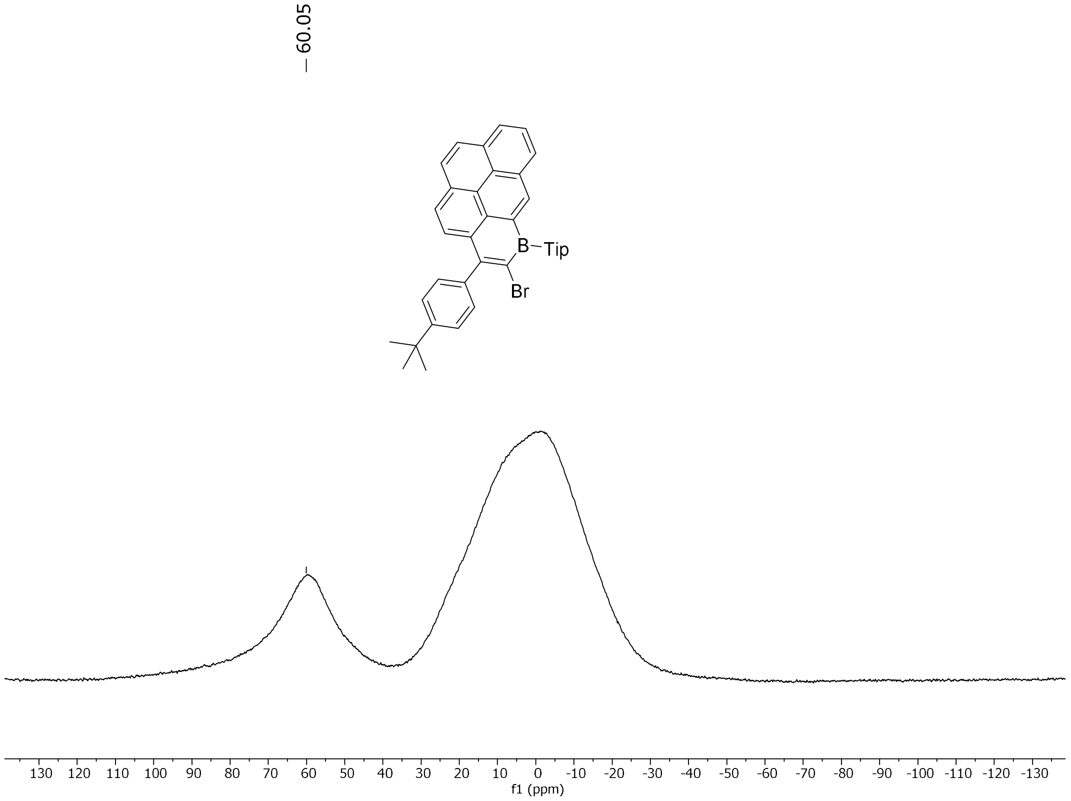

Figure S81. ^{11}B NMR spectrum of **3a** in $CDCl_3$.

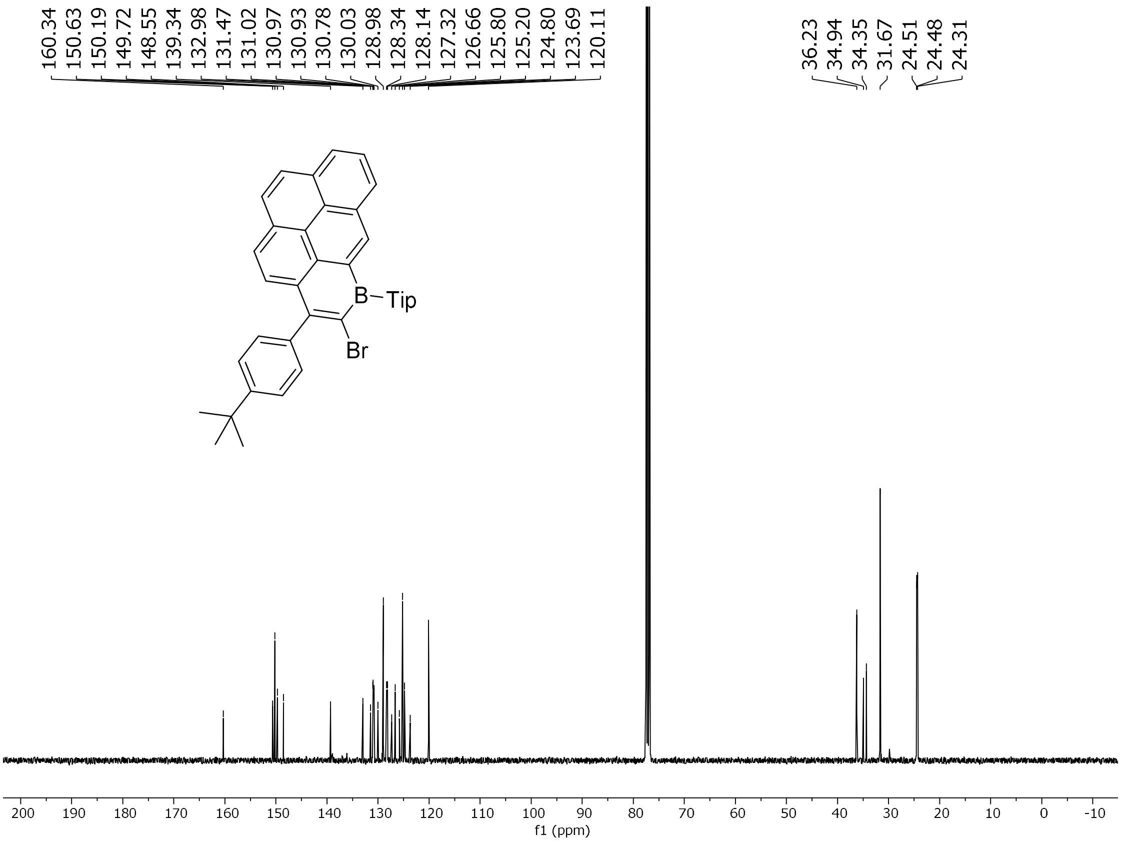

Figure S82. ^{13}C NMR spectrum of **3a** in $CDCl_3$.

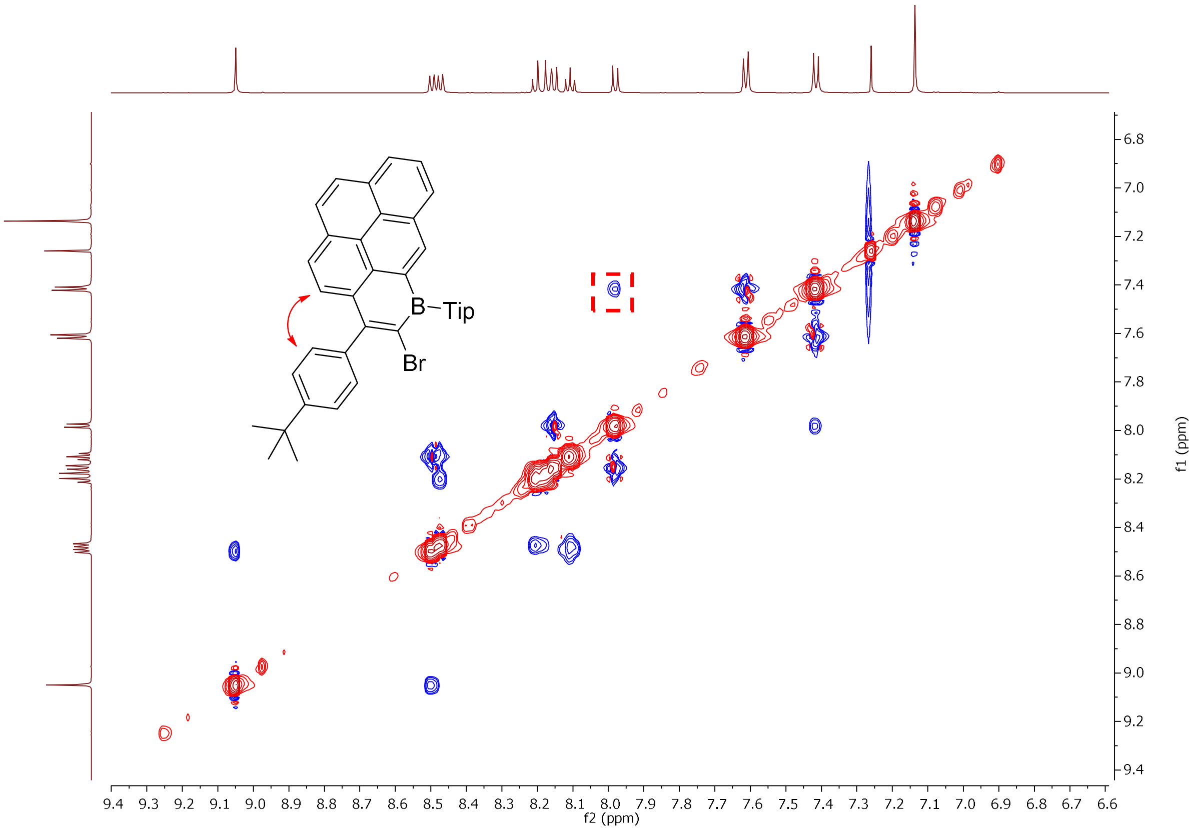

Figure S83. ^{1}H-^{1}H NOESY NMR spectrum of **3a** in $CDCl_3$.

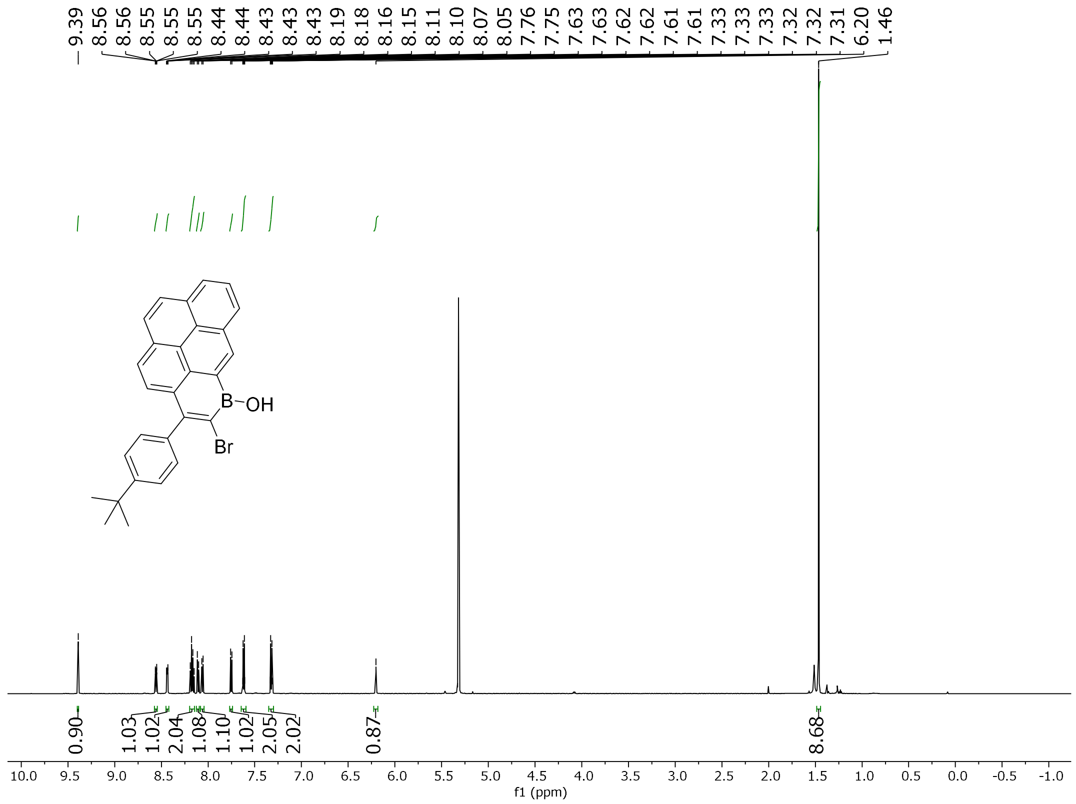

Figure S84. ^{1}H NMR spectrum of **3b** in CD_2Cl_2.

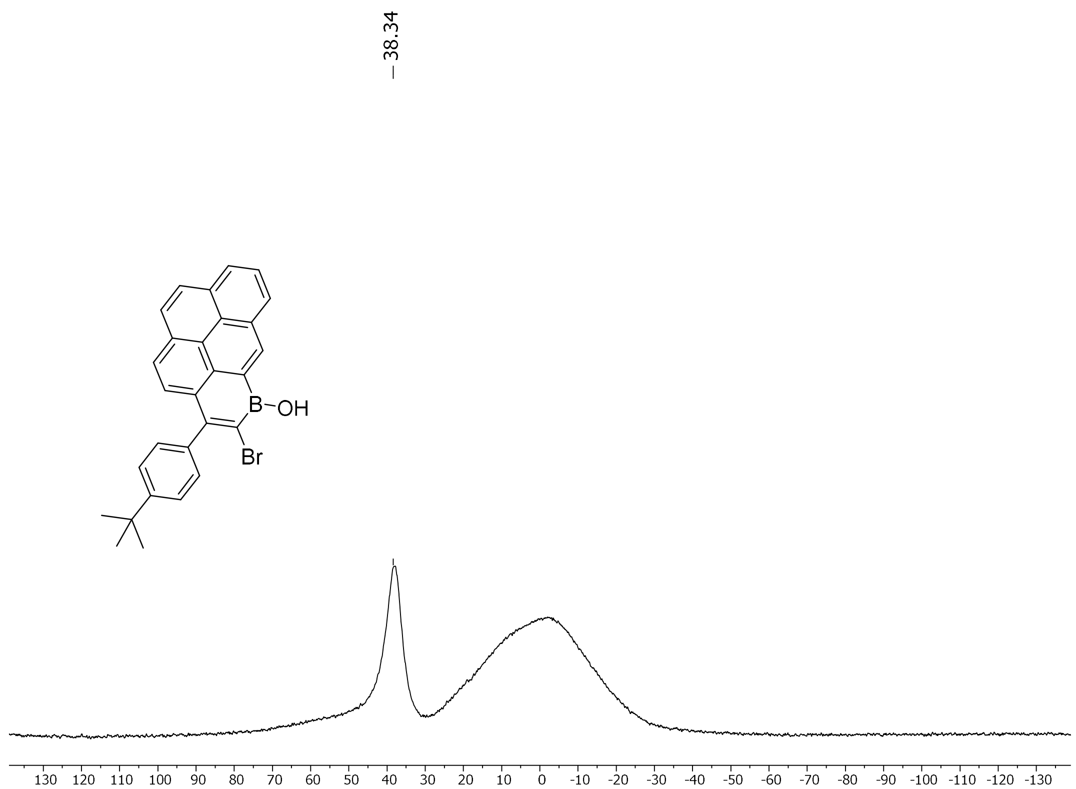

Figure S85. ^{11}B NMR spectrum of **3b** in $CDCl_3$.

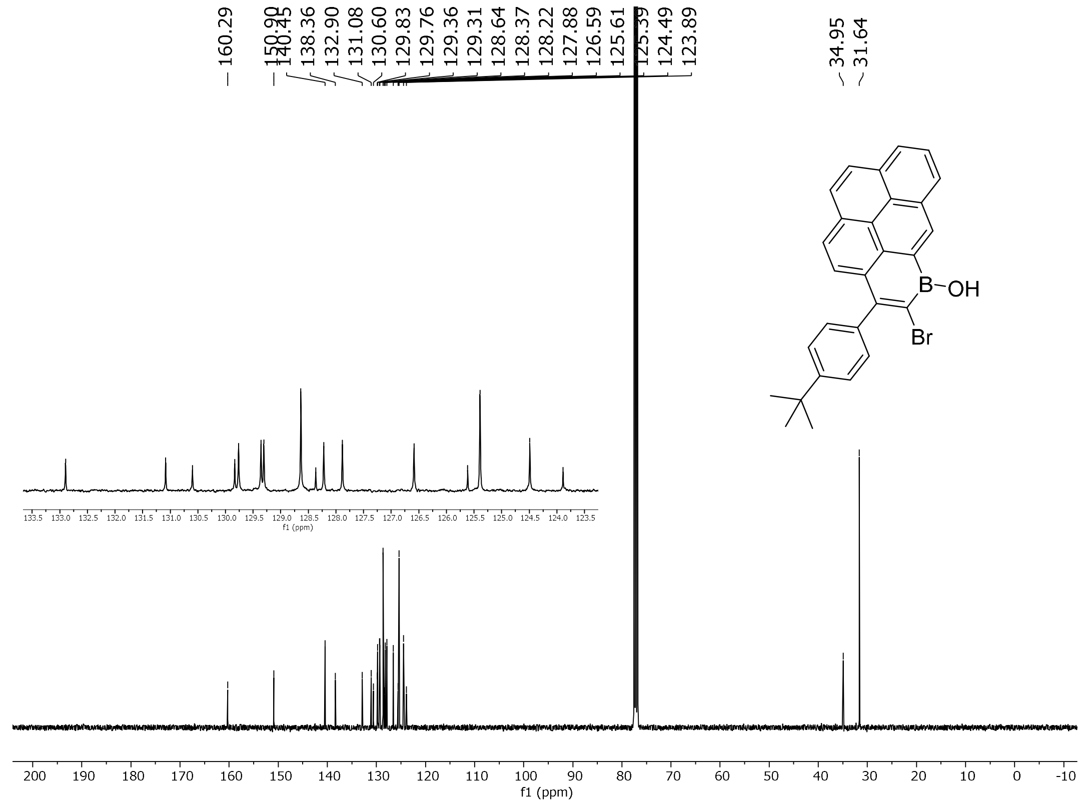

Figure S86. $^{13}C\{^{1}H\}$ NMR spectrum of **3b** in $CDCl_3$.

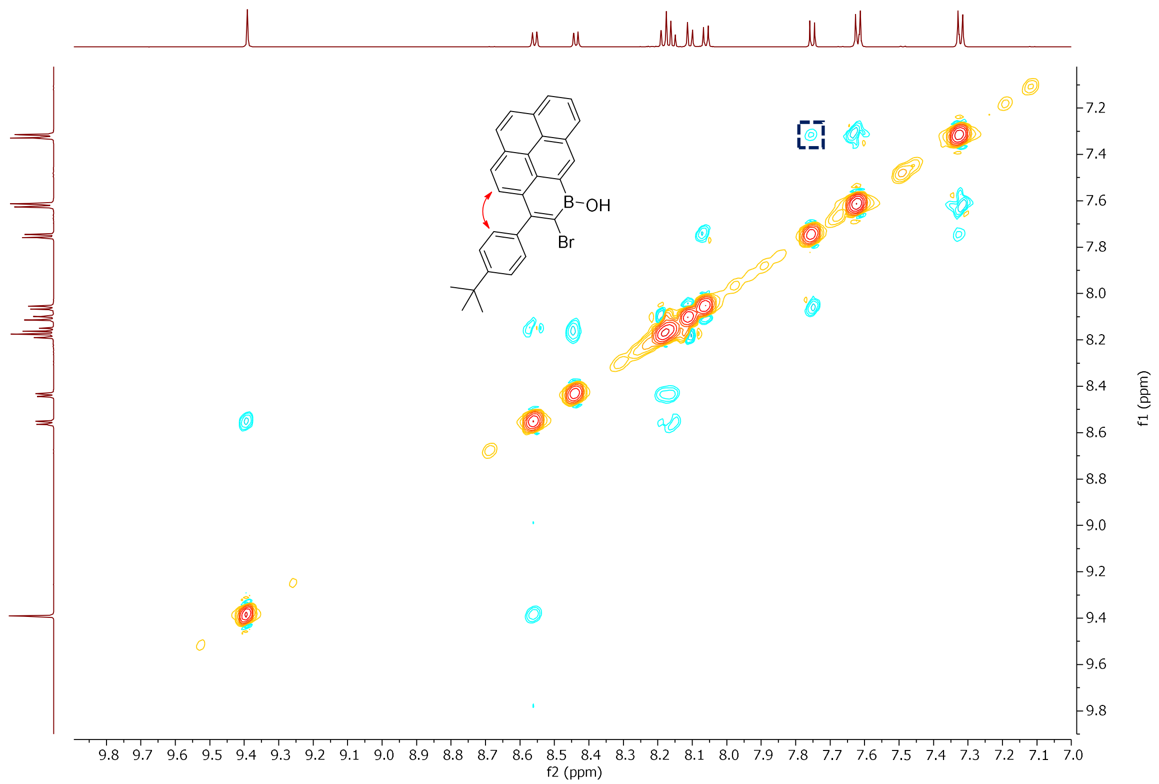

Figure S87. ^{1}H-^{1}H NOESY NMR spectrum of **3b** in CD_2Cl_2.

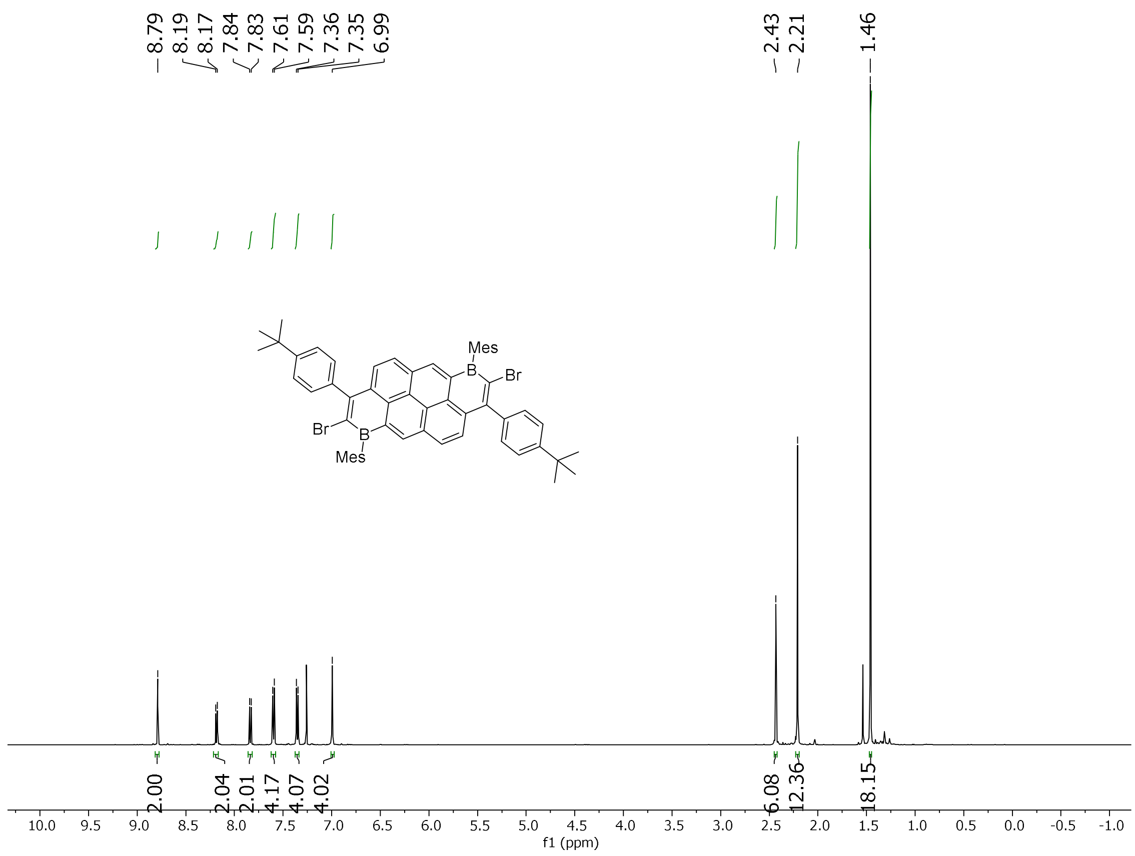

Figure S88. ^{1}H NMR spectrum of **9a** in $CDCl_3$.

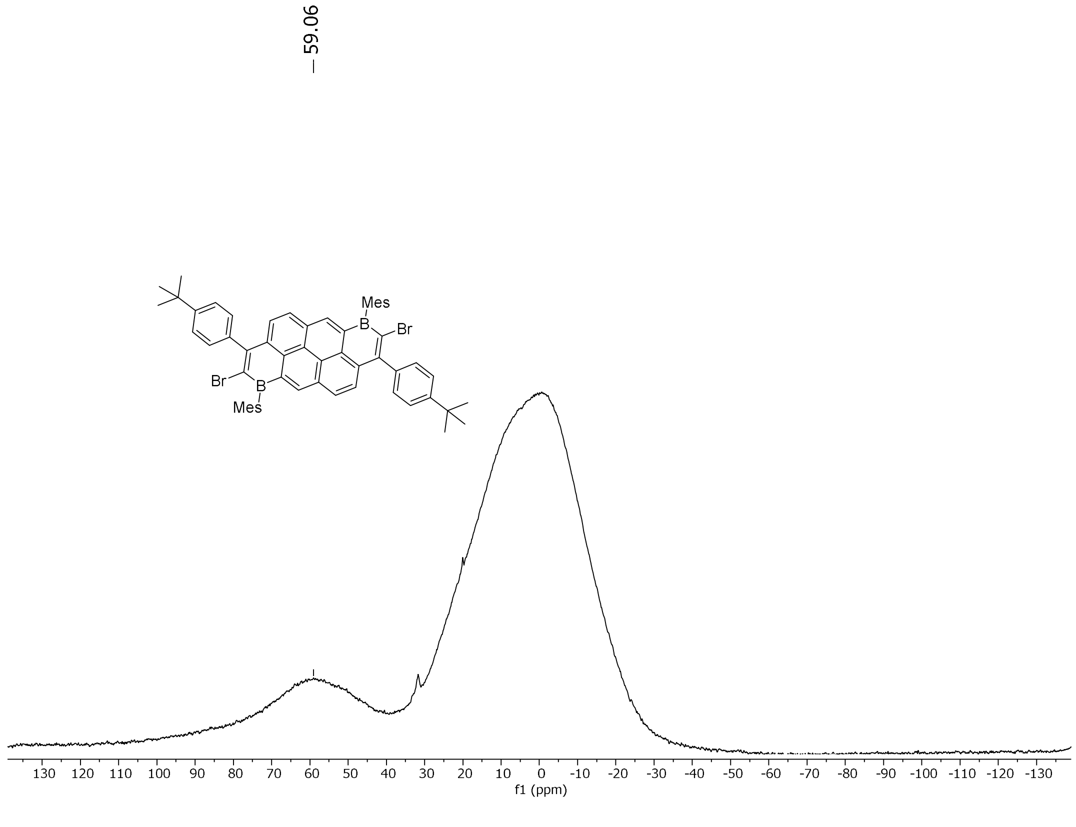

Figure S89. ^{11}B NMR spectrum of **9a** in $CDCl_3$.

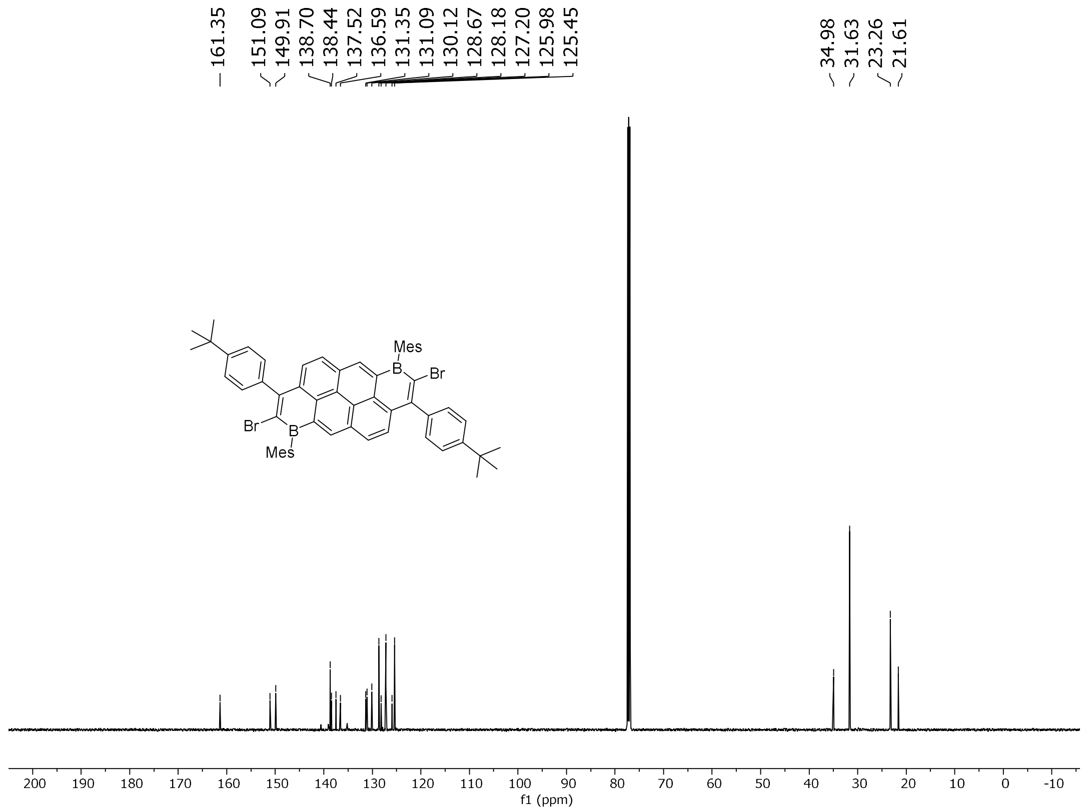

Figure S90. $^{13}C\{^{1}H\}$ NMR spectrum of **9a** in $CDCl_3$.

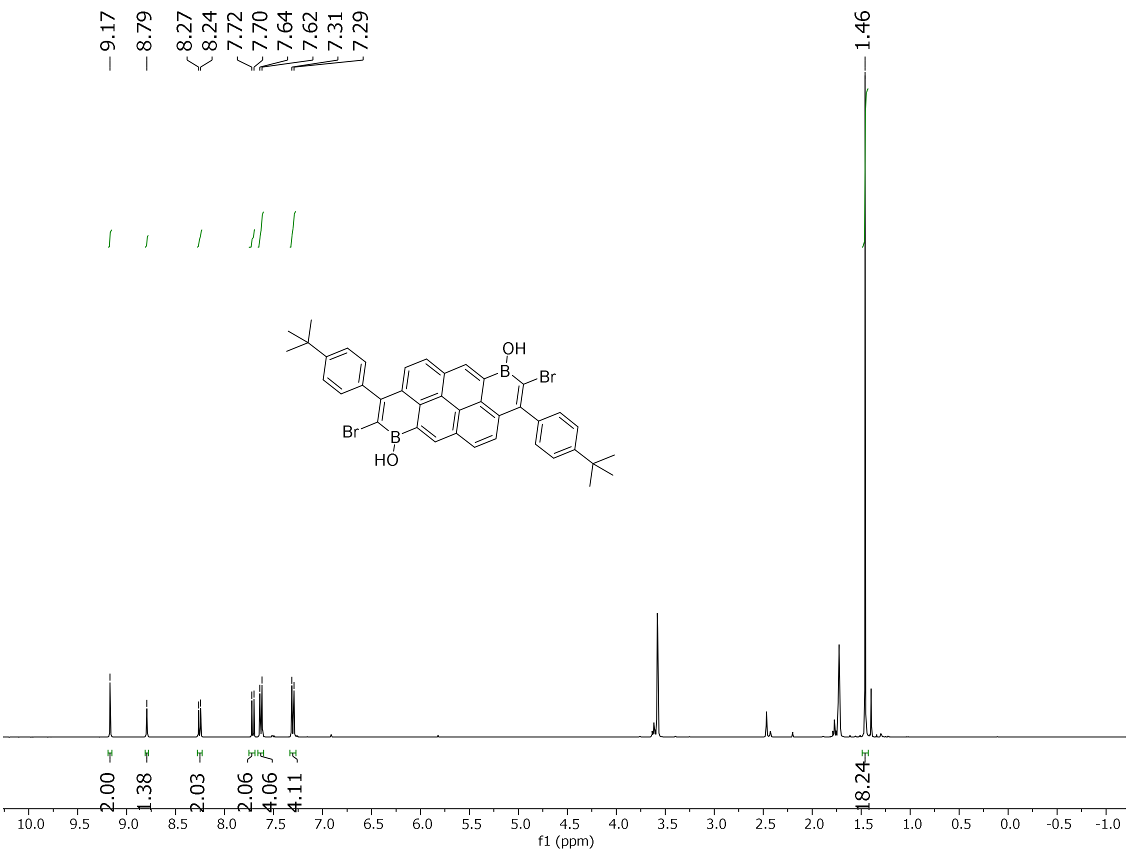

Figure S91. ^{1}H NMR spectrum of **9b** in THF-d_8.

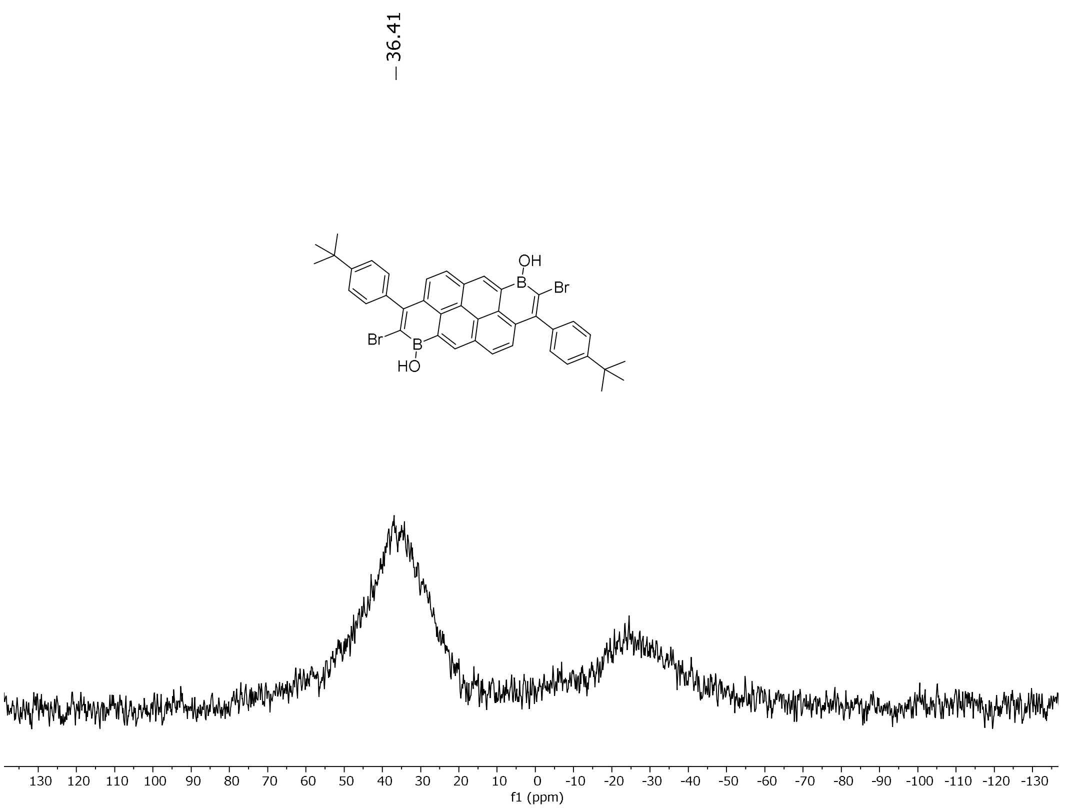

Figure S92. ^{11}B NMR spectrum of **9b** in THF-d_8. Note background subtracted NMR spectrum.

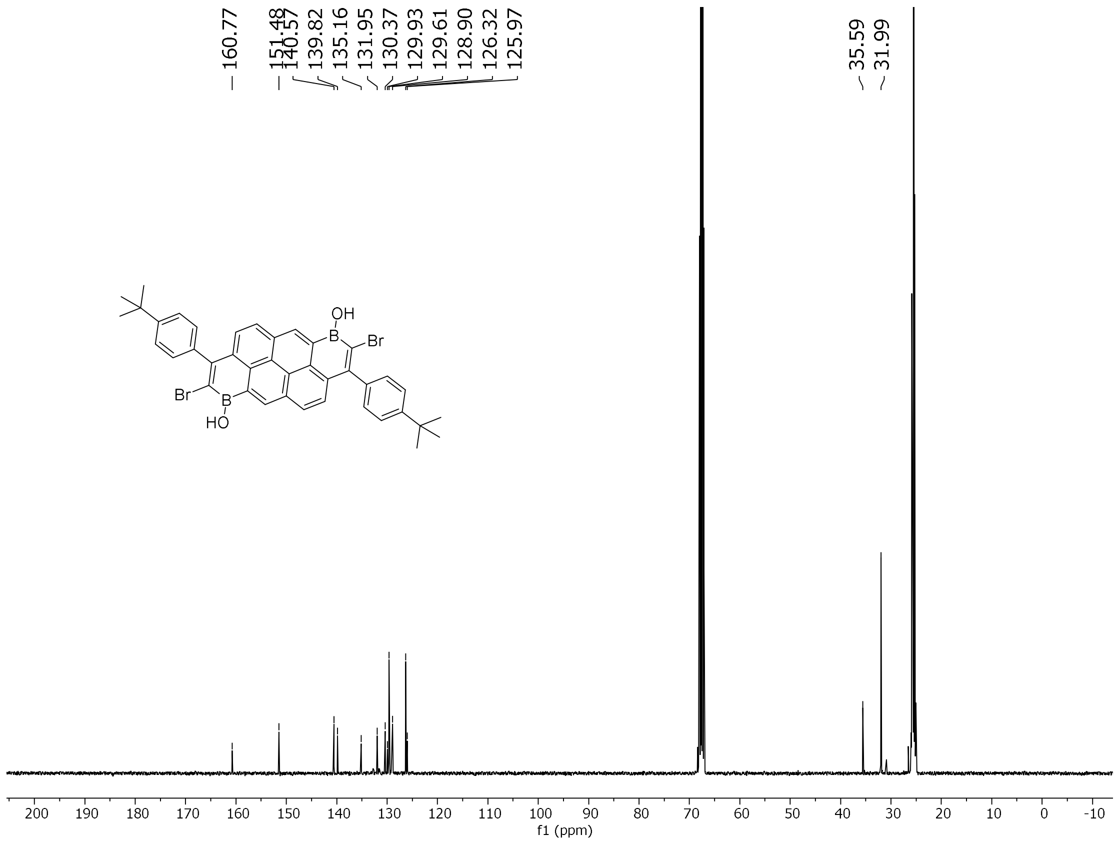

Figure S93. ^{13}C{^{1}H} NMR spectrum of **9b** in THF-d_8.

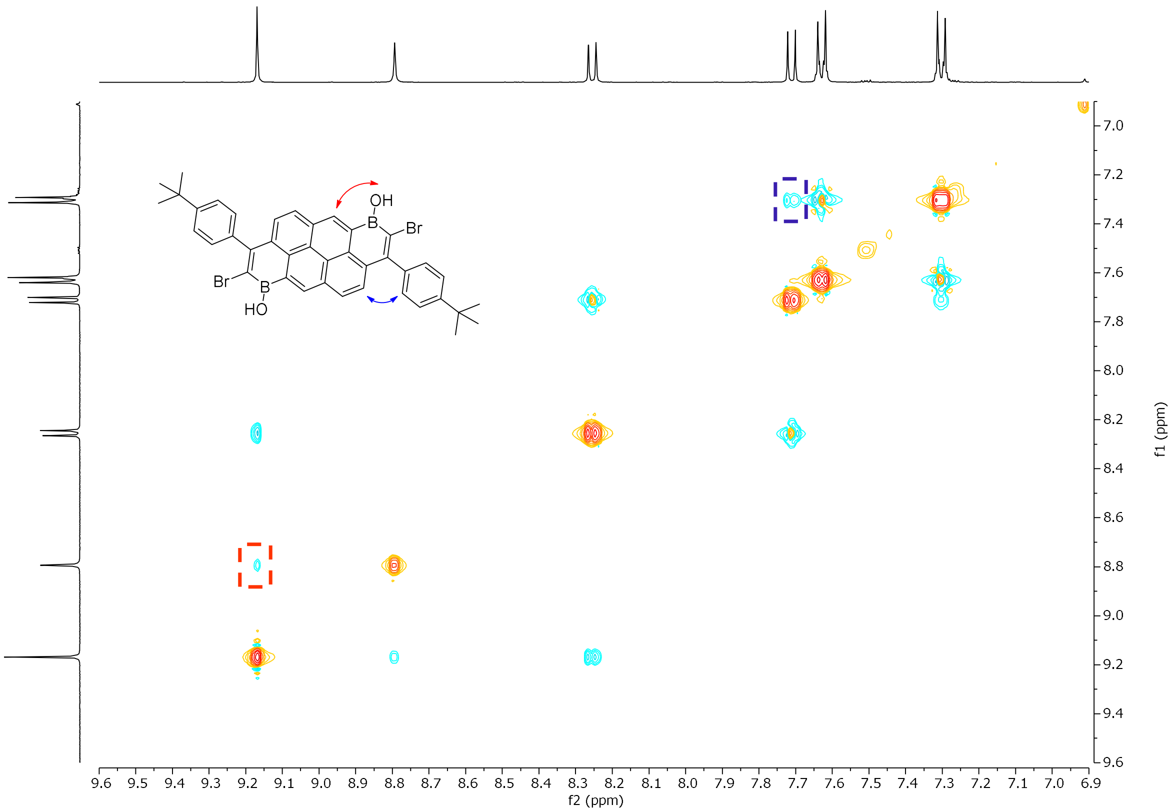

Figure S94. ^{1}H-^{1}H NOESY NMR spectrum of **9b** in THF-d_8.

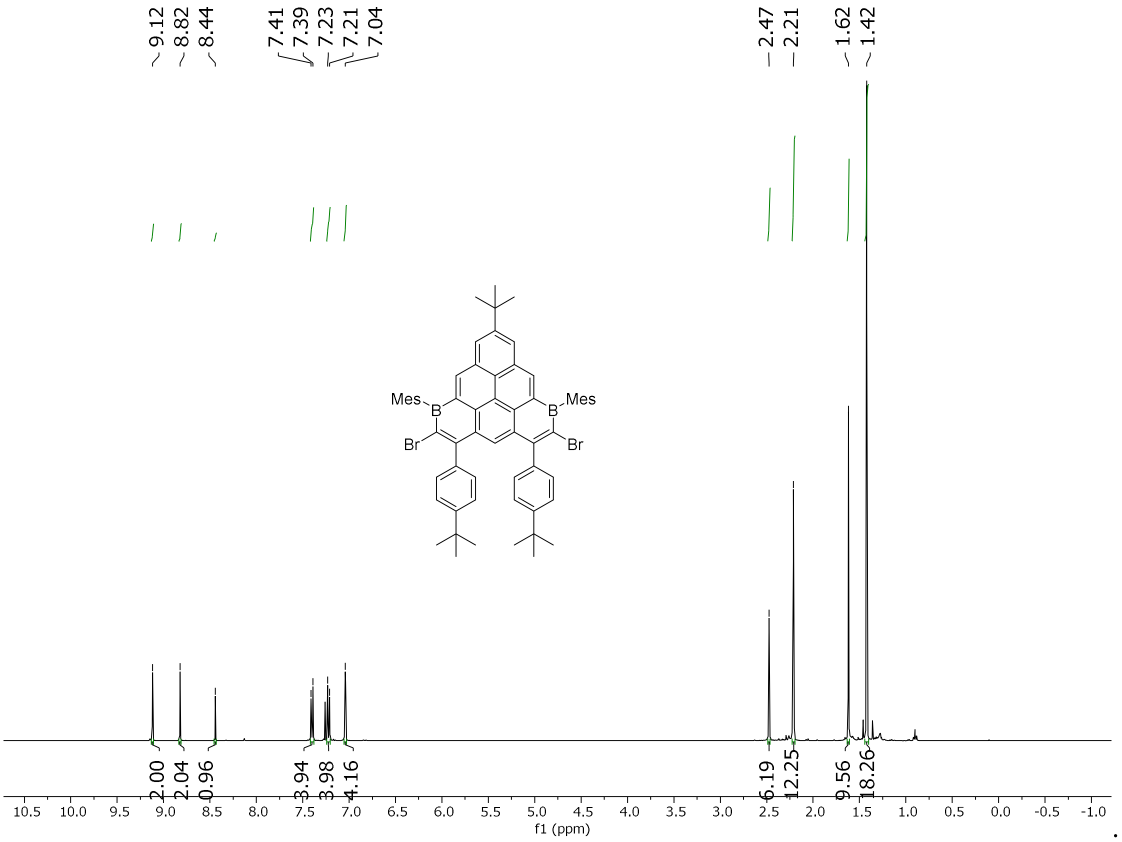

Figure S95. ^{1}H NMR spectrum of **10a** in CDCl$_3$.

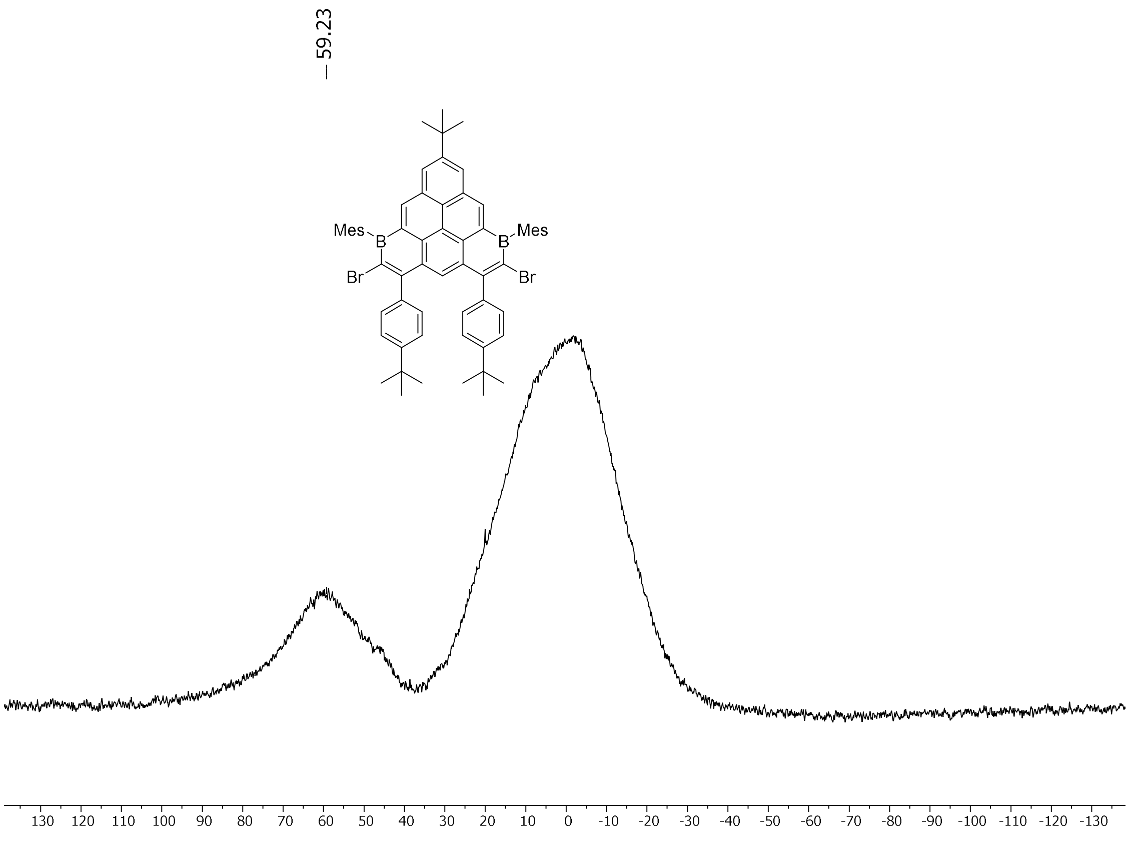

Figure S96. ^{11}B NMR spectrum of **10a** in $CDCl_3$.

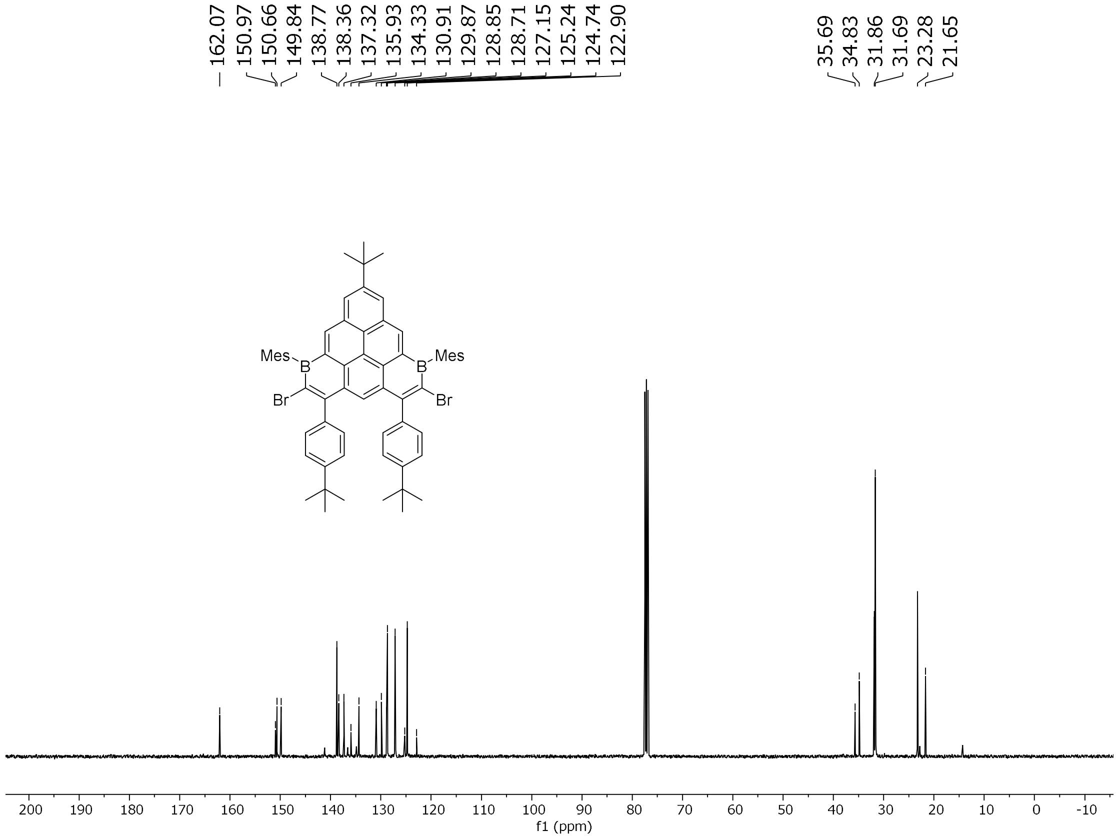

Figure S97. $^{13}C\{^{1}H\}$ NMR spectrum of **10a** in $CDCl_3$.

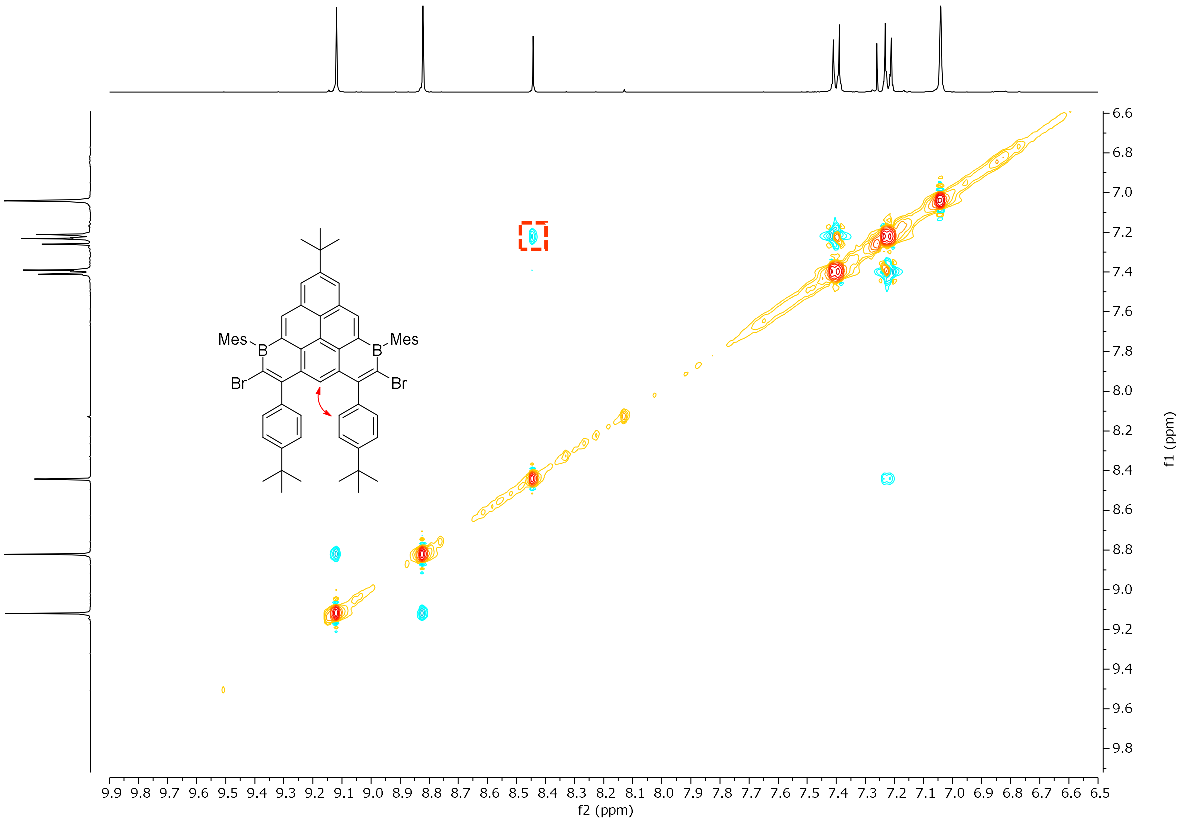

Figure S98. ^{1}H-^{1}H NOESY NMR spectrum of **10a** in $CDCl_3$.

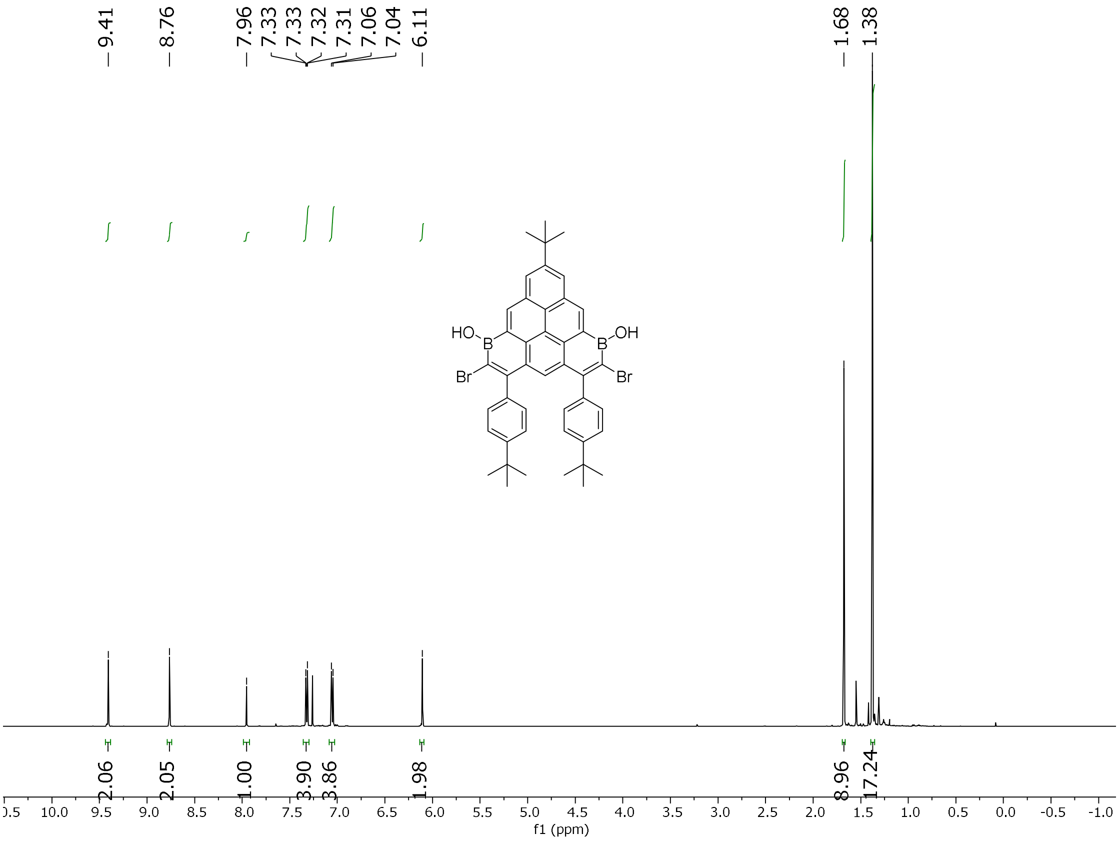

Figure S99. ^{1}H NMR spectrum of **10b** in $CDCl_3$.

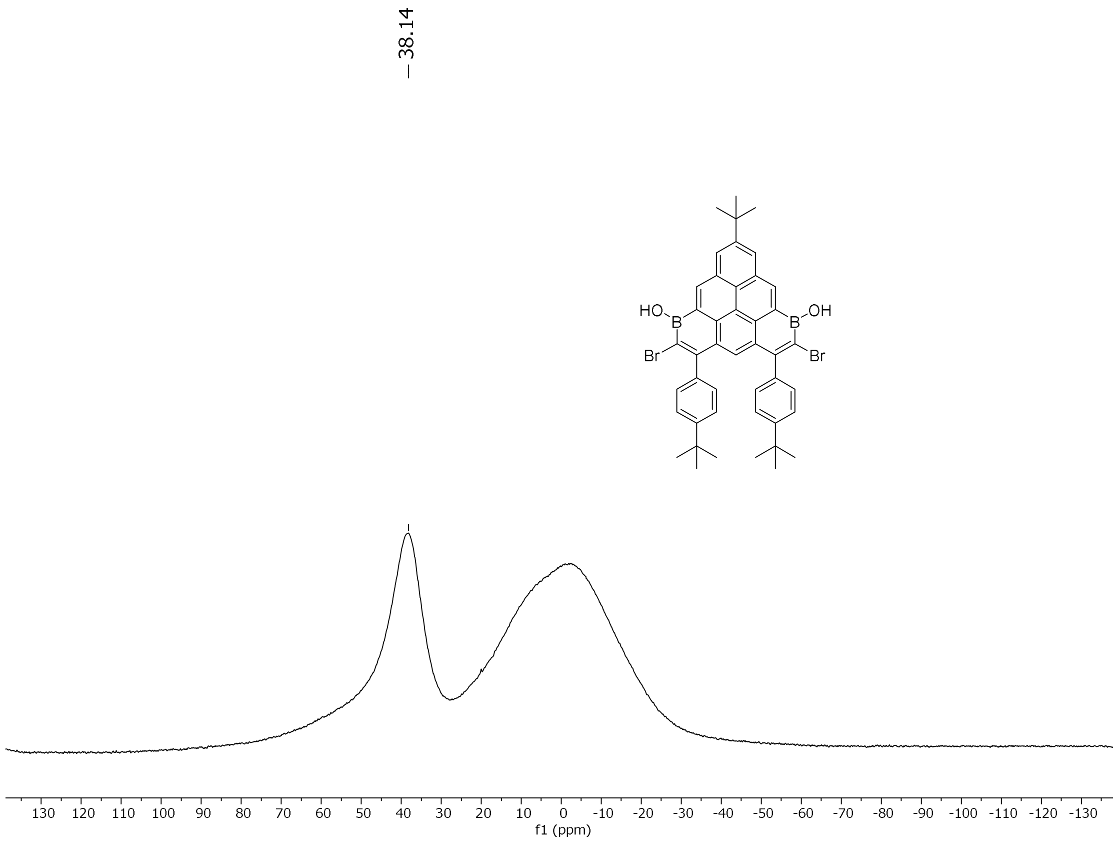

Figure S100. ^{11}B NMR spectrum of **10b** in $CDCl_3$.

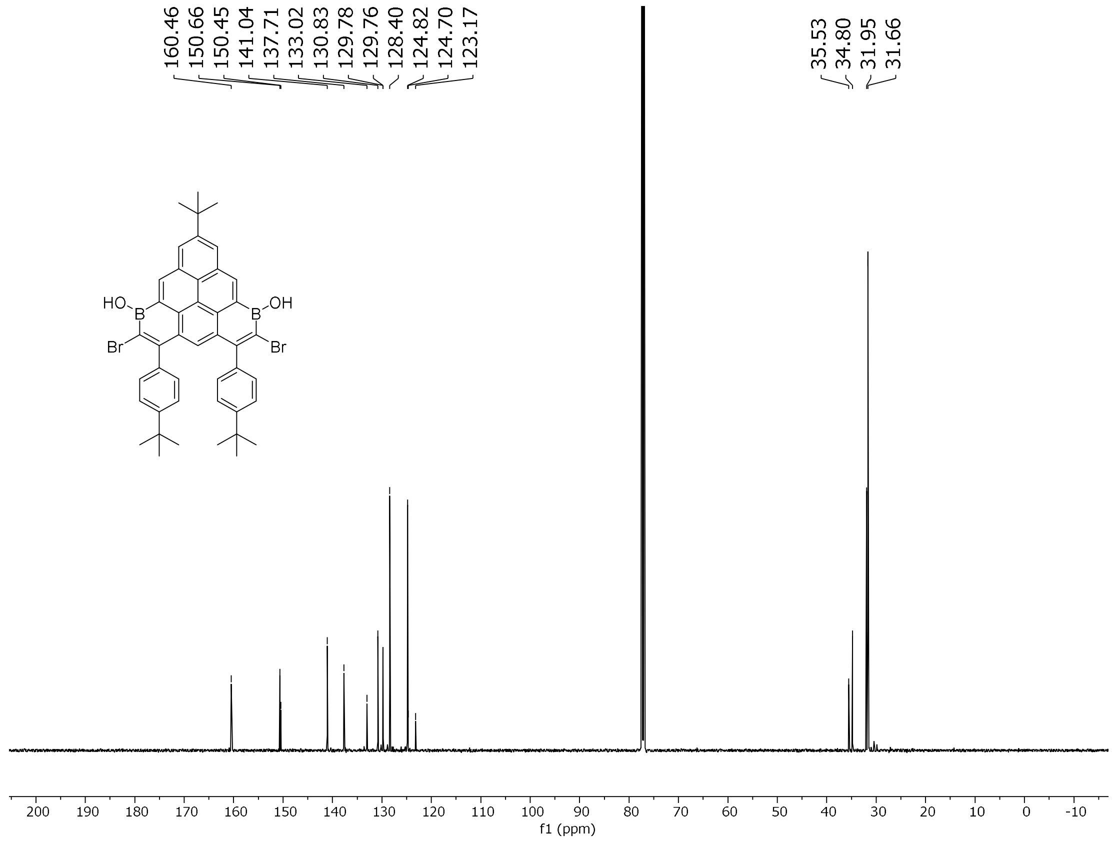

Figure S101. $^{13}C\{^{1}H\}$ NMR spectrum of **10b** in $CDCl_3$.

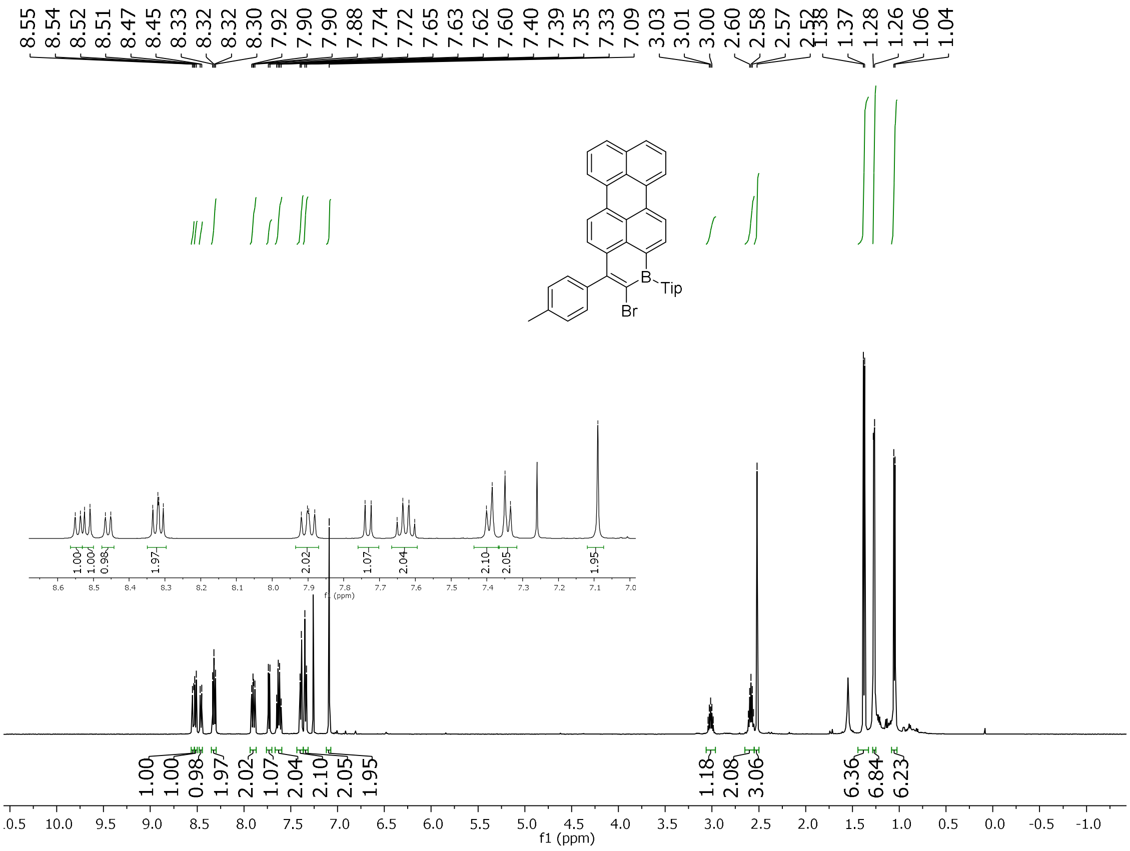

Figure S102. ^{1}H NMR spectrum of **11a** in $CDCl_3$.

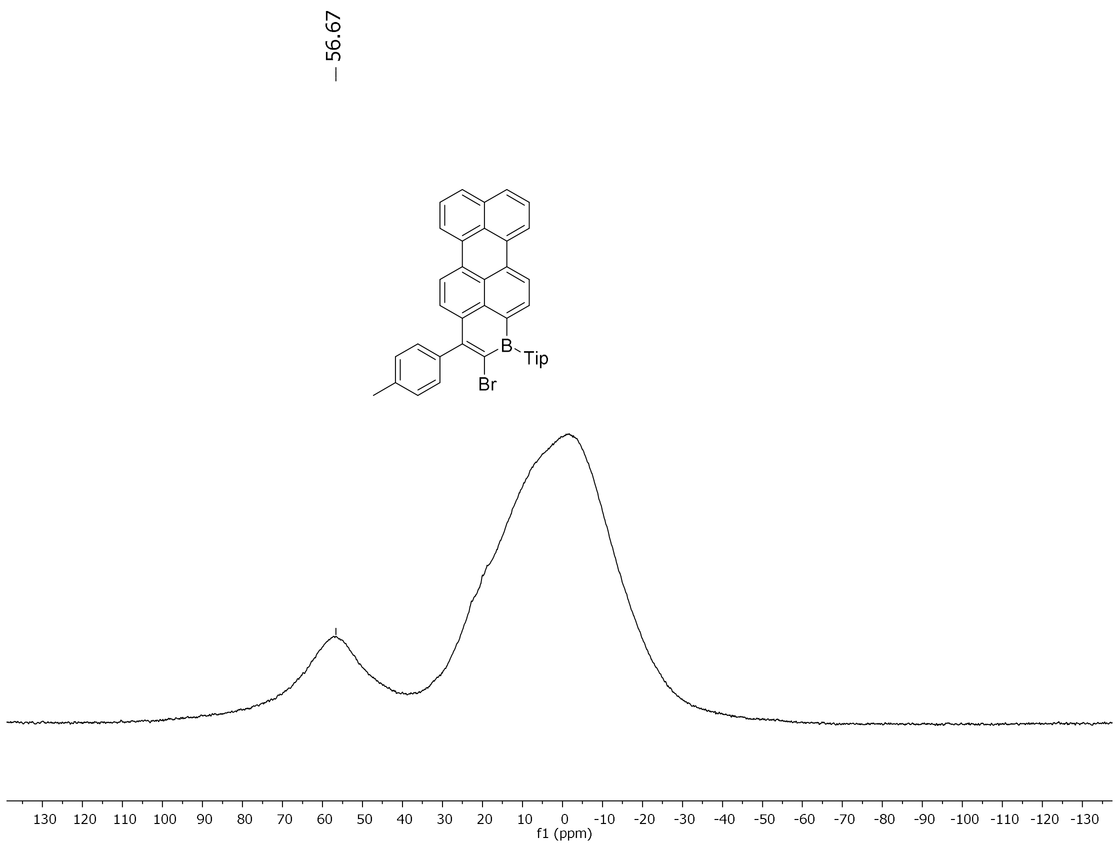

Figure S103. ^{11}B NMR spectrum of **11a** in $CDCl_3$.

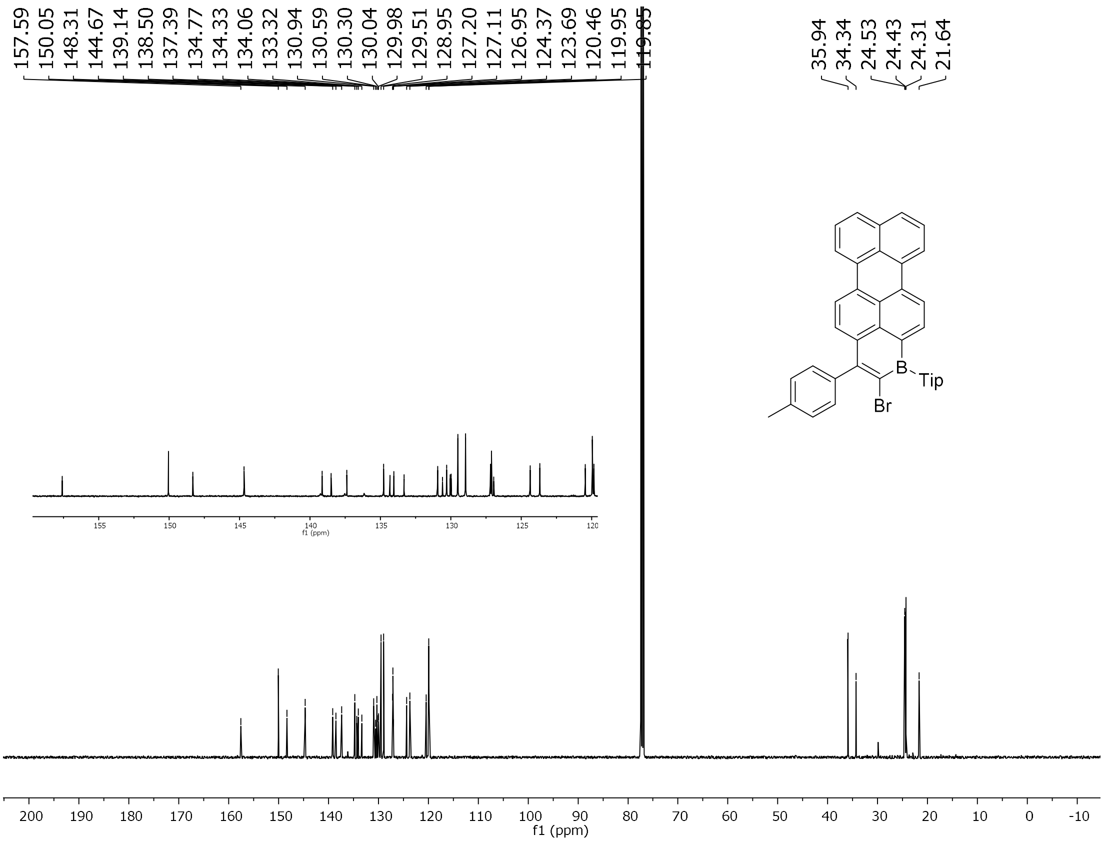

Figure S104. $^{13}C\{^{1}H\}$ NMR spectrum of **11a** in $CDCl_3$.

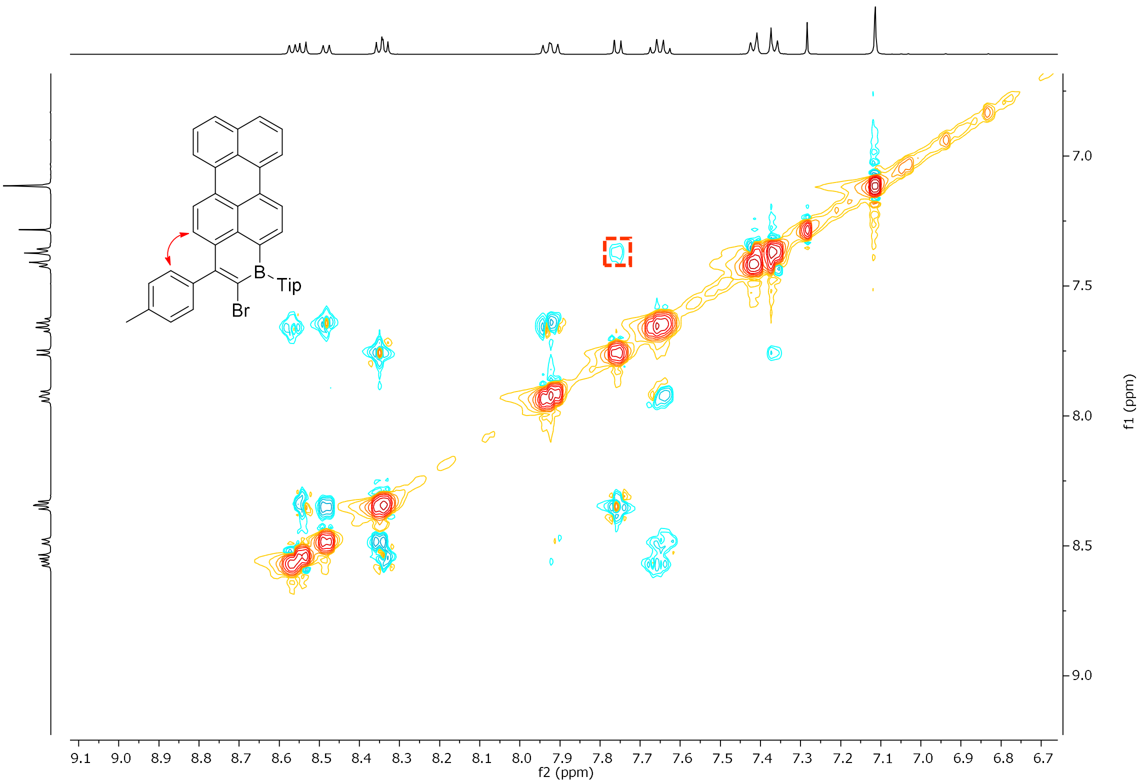

Figure S105. ^{1}H-^{1}H NOESY NMR spectrum of **11a** in $CDCl_3$.

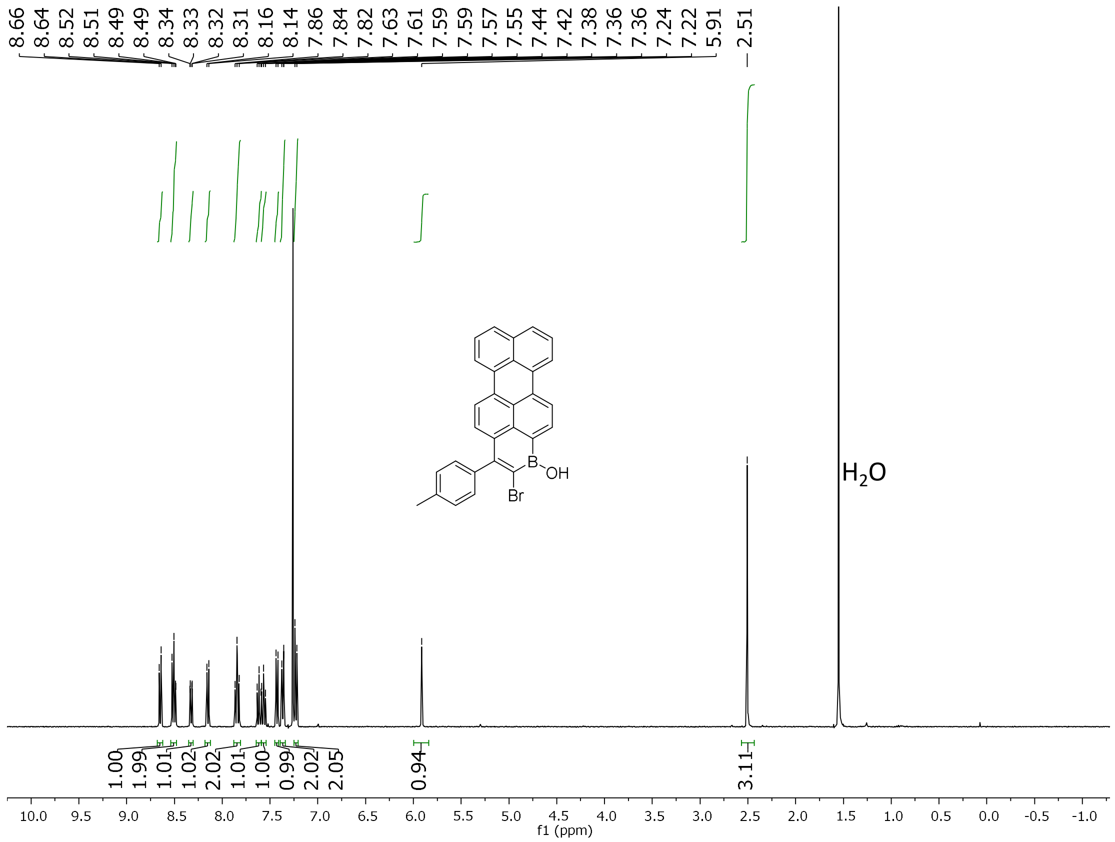

Figure S106. ^{1}H NMR spectrum of **11b** in $CDCl_3$.

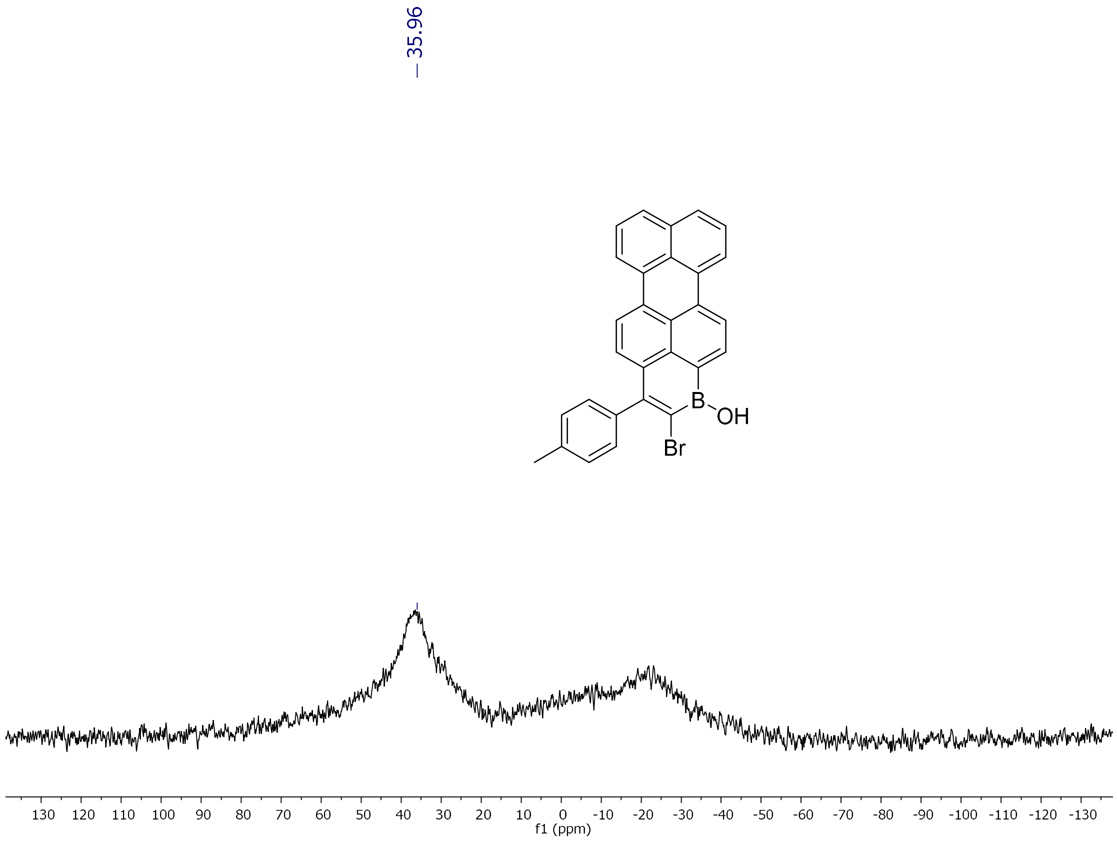

Figure S107. ^{11}B NMR spectrum of **11b** in $CDCl_3$. Note background subtracted spectrum.

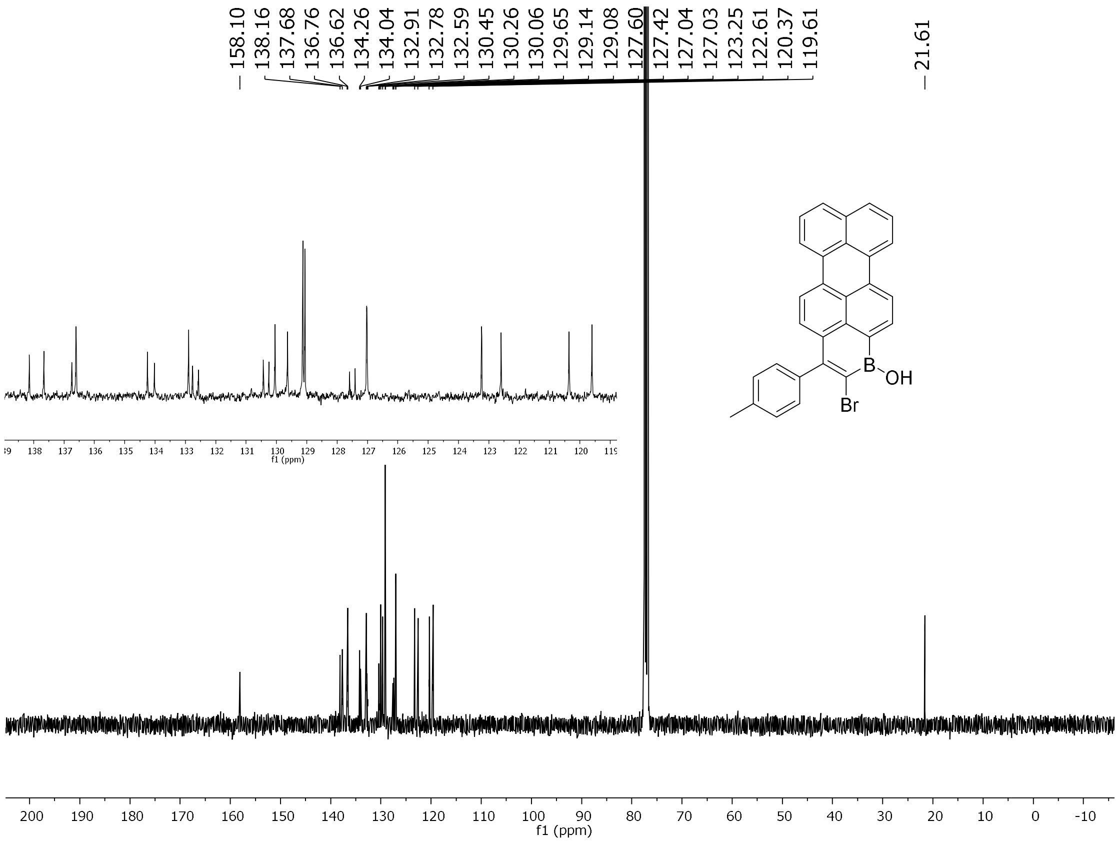

Figure S108. $^{13}C\{^{1}H\}$ NMR spectrum of **11b** in $CDCl_3$.

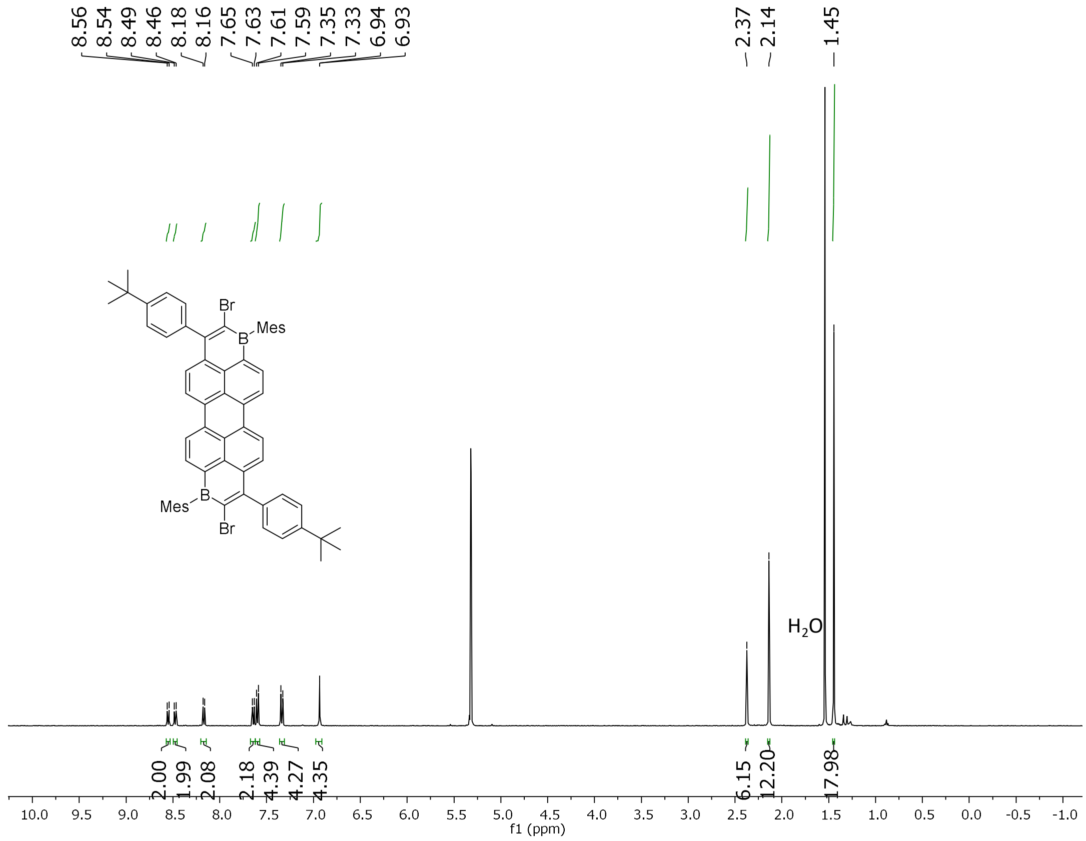

Figure S109. ^{1}H NMR spectrum of **12a** in CD_2Cl_2.

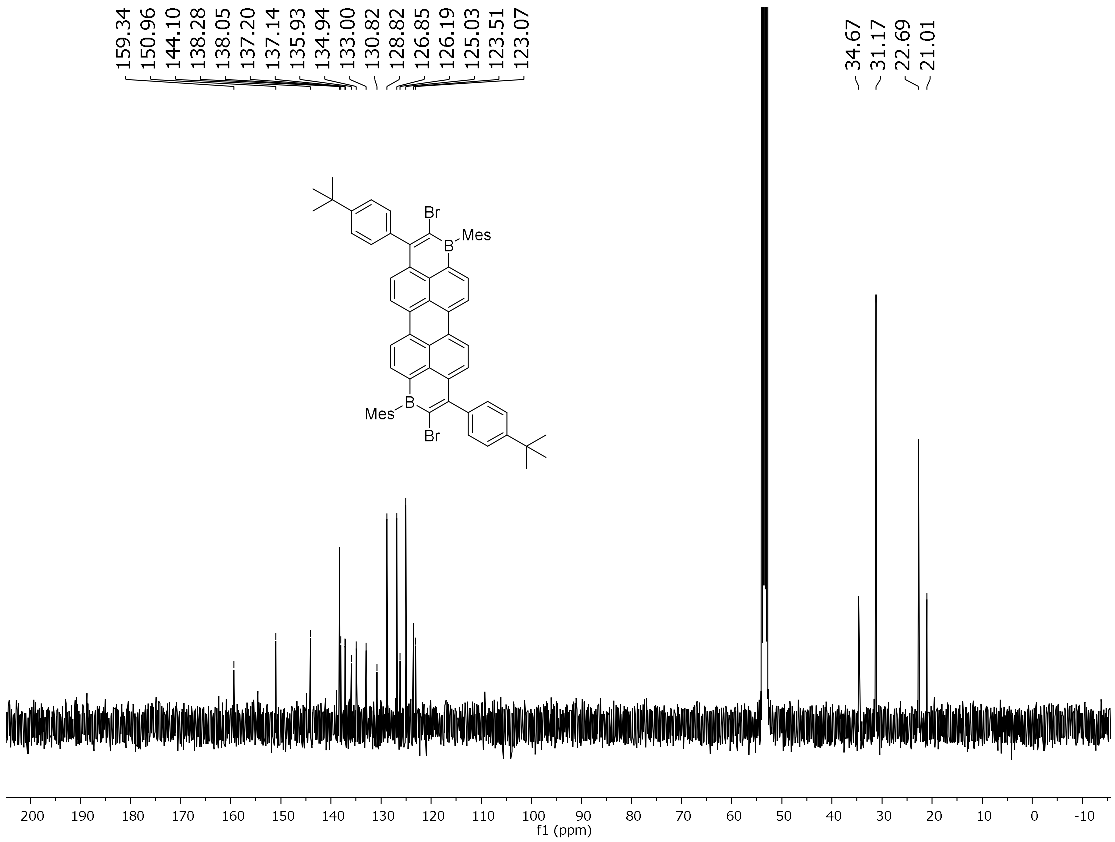

Figure S110. $^{13}C\{^{1}H\}$ NMR spectrum of **12a** in CD_2Cl_2.

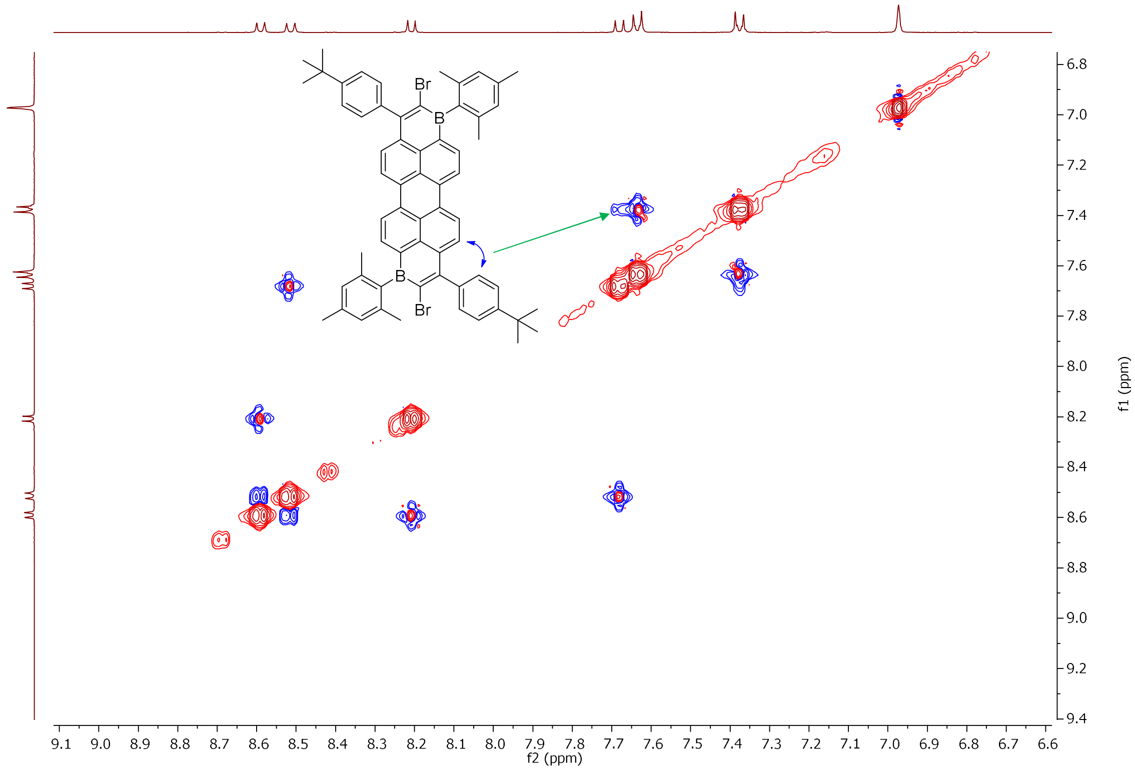

Figure S111. ^{1}H-^{1}H NOESY NMR spectrum of **12a** in CD_2Cl_2.

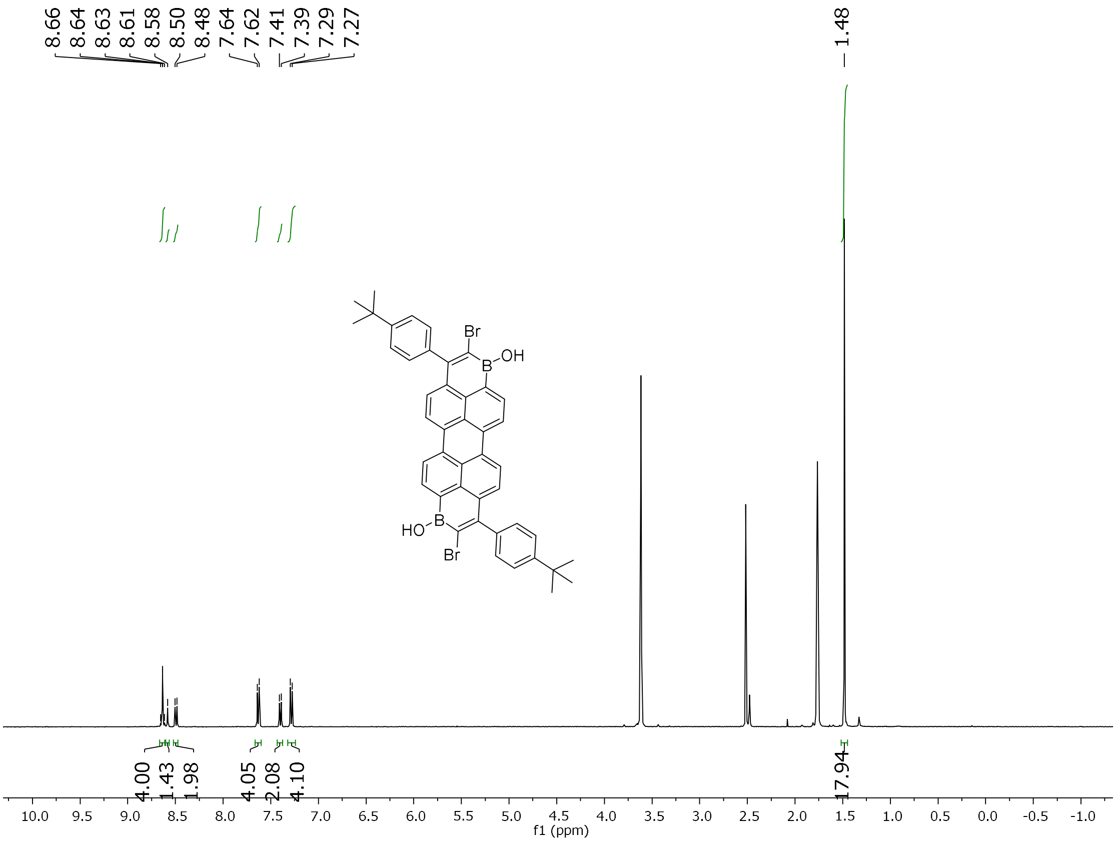

Figure S112. ^{1}H NMR spectrum of **12b** in THF-d_8.

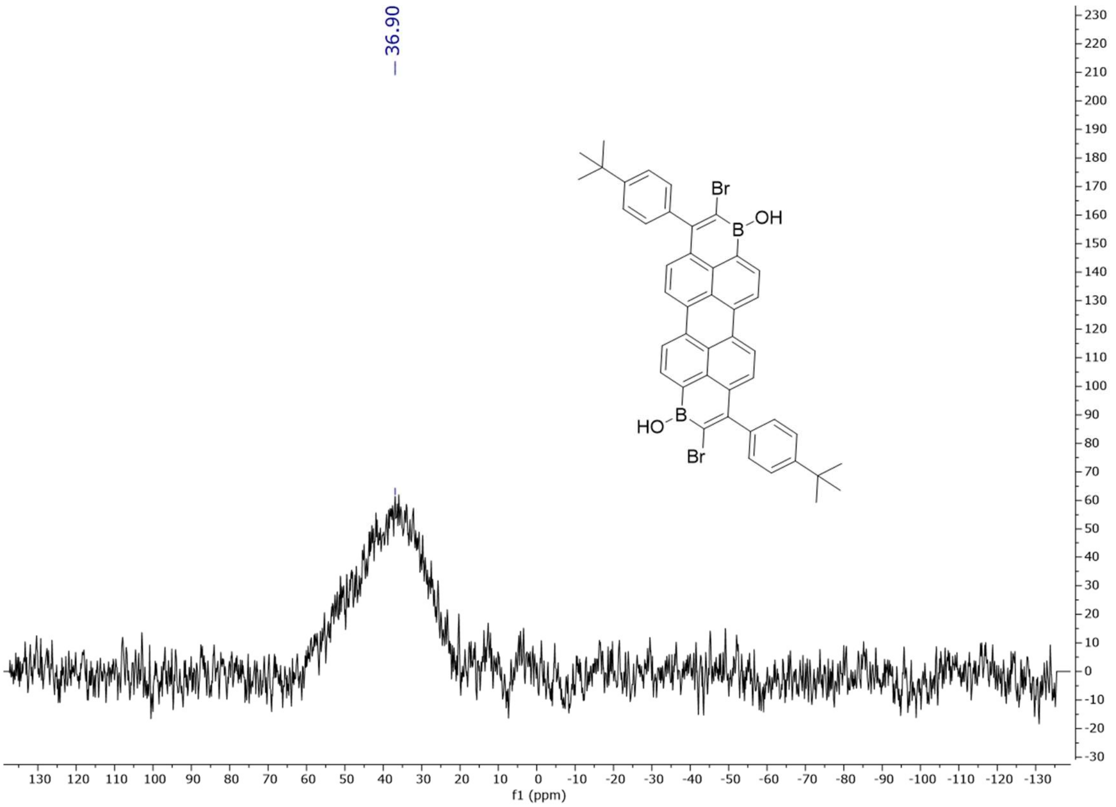

Figure S113. ^{11}B NMR spectrum of **12b** in THF-d_8. Note background subtracted spectrum.

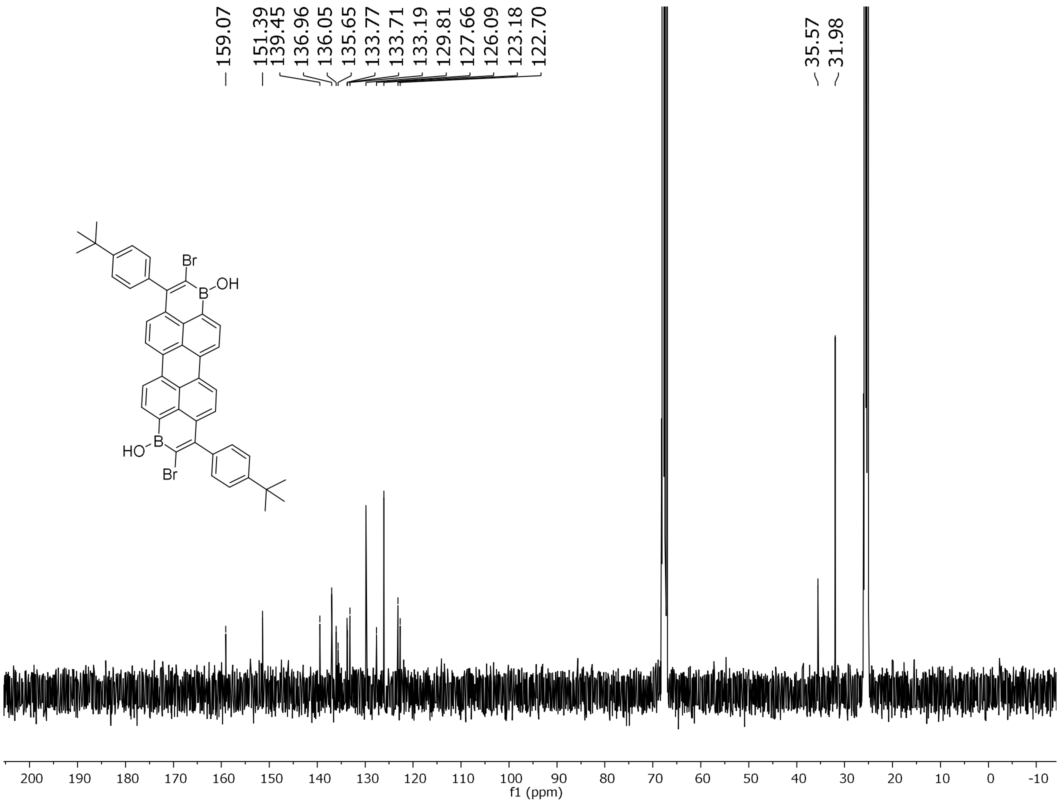

Figure S114. $^{13}C\{^{1}H\}$ NMR spectrum of **12b** in THF-d_8.

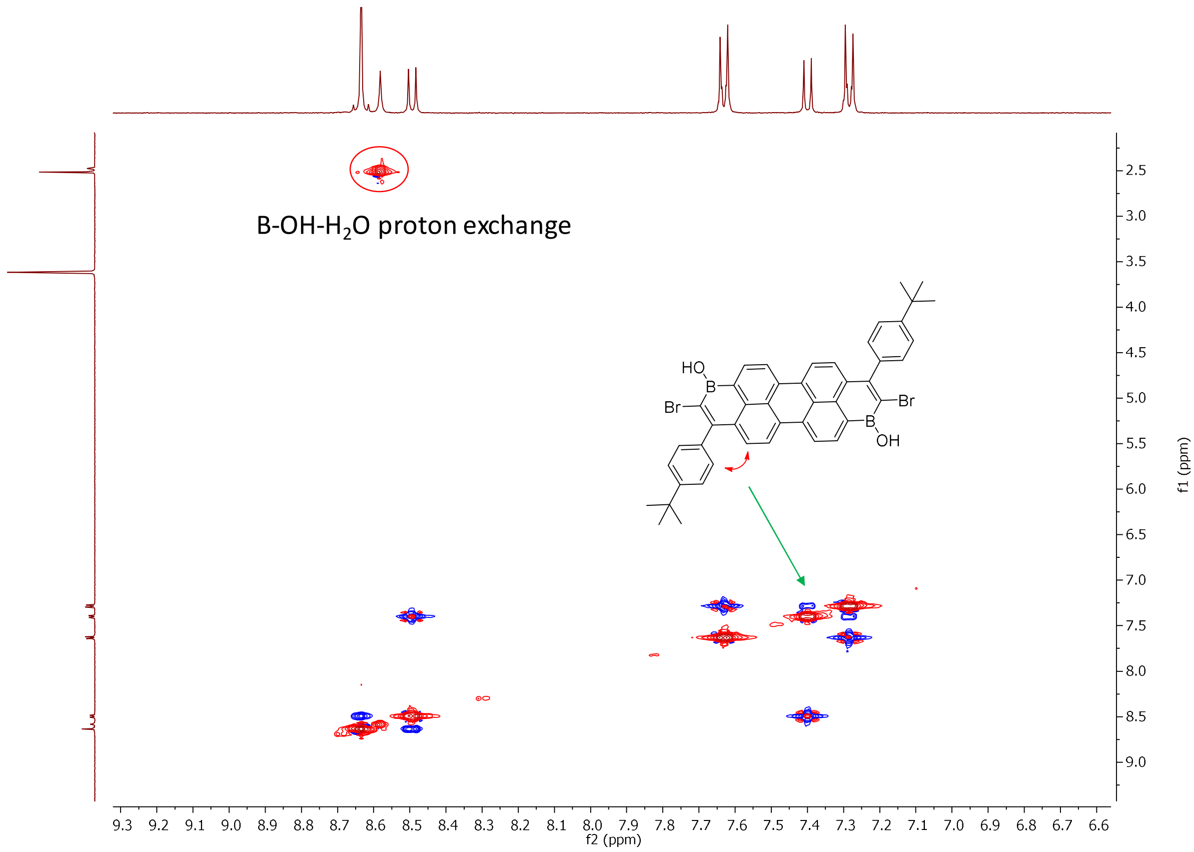

Figure S115. ^{1}H-^{1}H NOESY NMR spectrum of **12b** in THF-d_8.

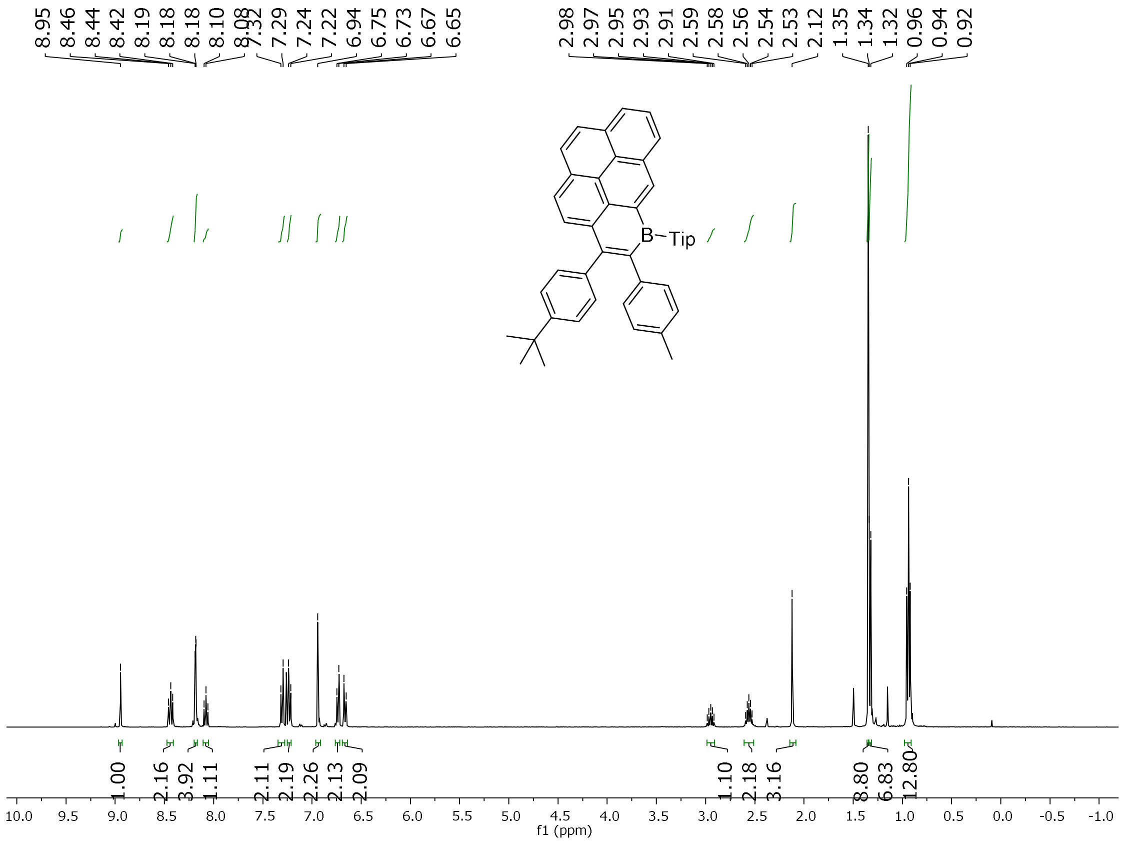

Figure S116. ^{1}H NMR spectrum of **13** in $CDCl_3$.

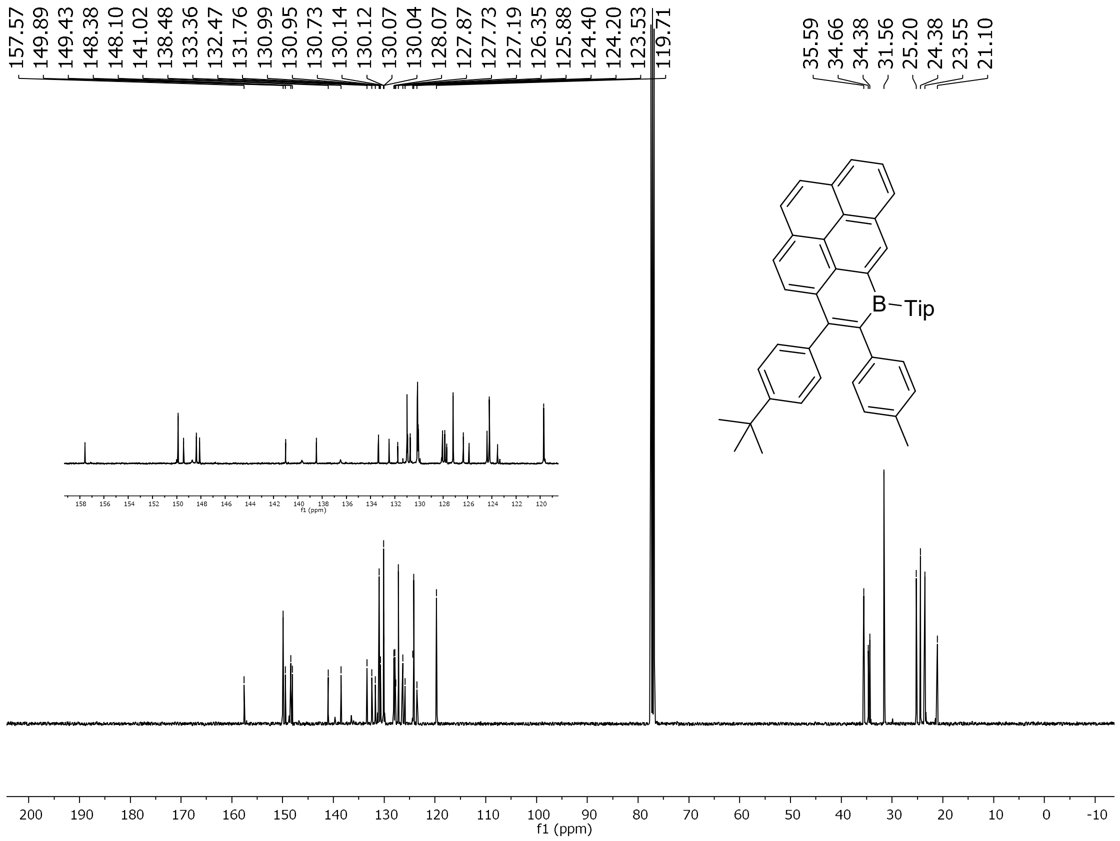

Figure S117. $^{13}C\{^{1}H\}$ NMR spectrum of **13** in $CDCl_3$.

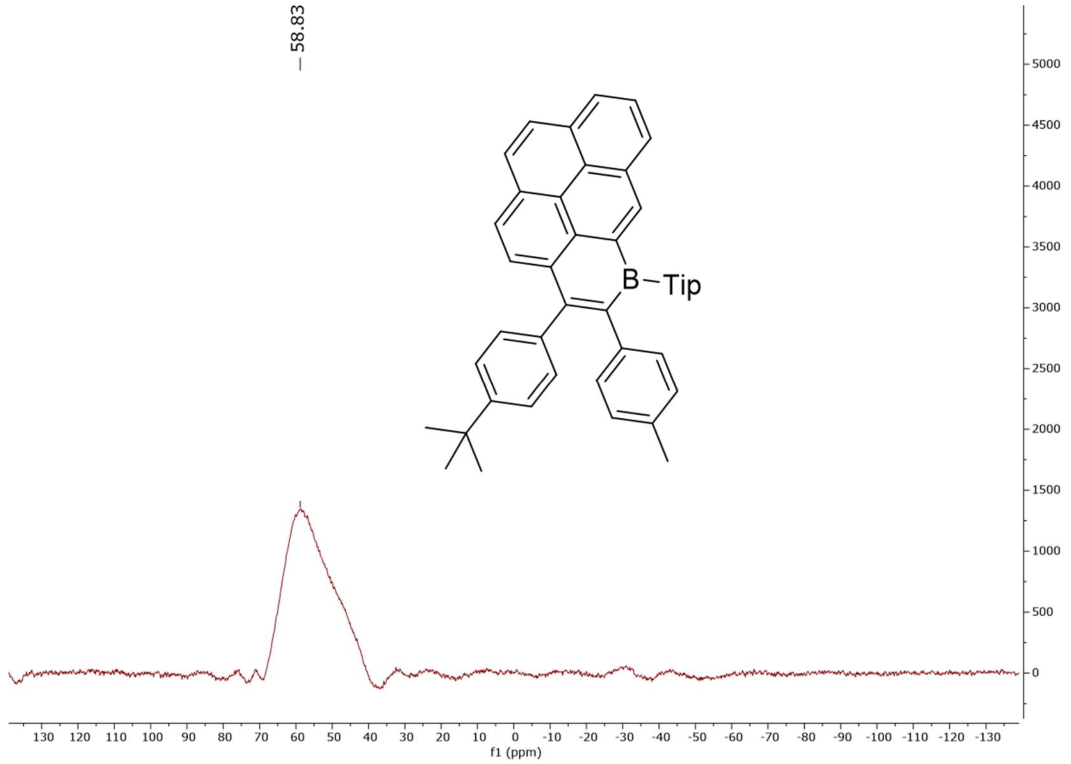

Figure S118. ^{11}B NMR spectrum of **13** in $CDCl_3$. Note background subtracted spectrum.

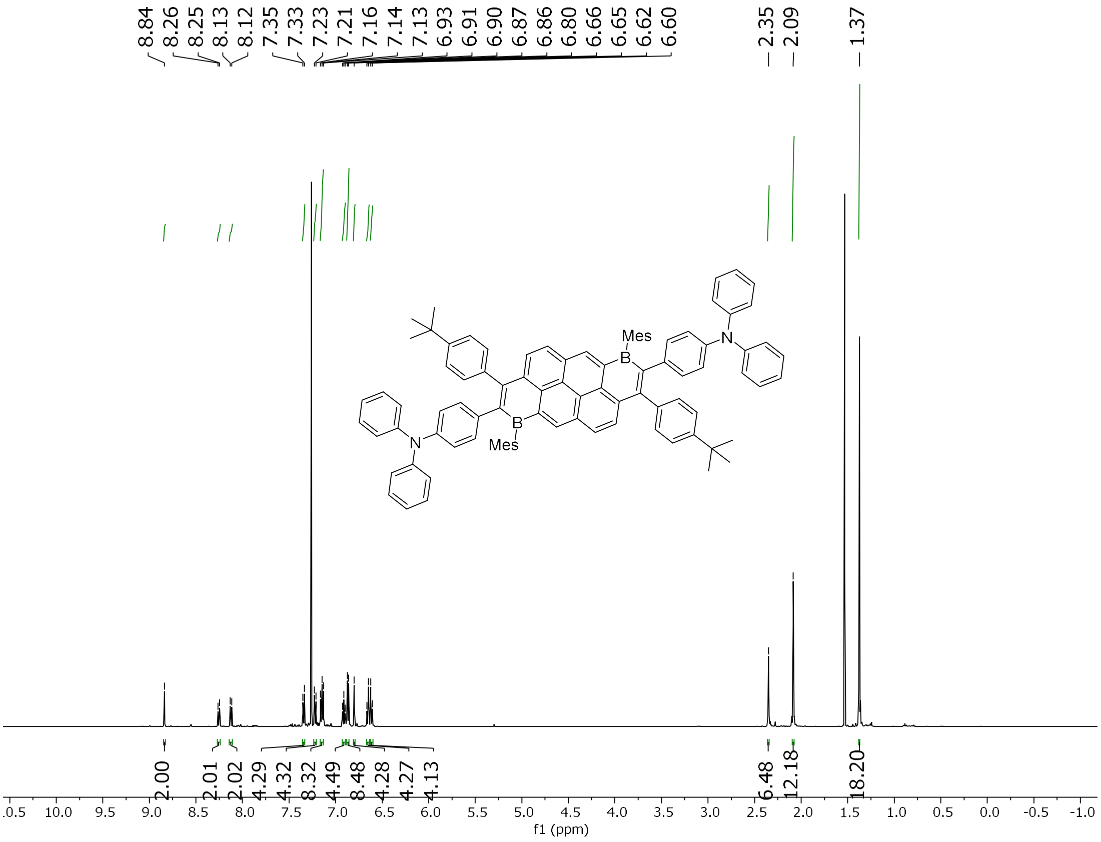

Figure S119. ^{1}H NMR spectrum of **14** in $CDCl_3$.

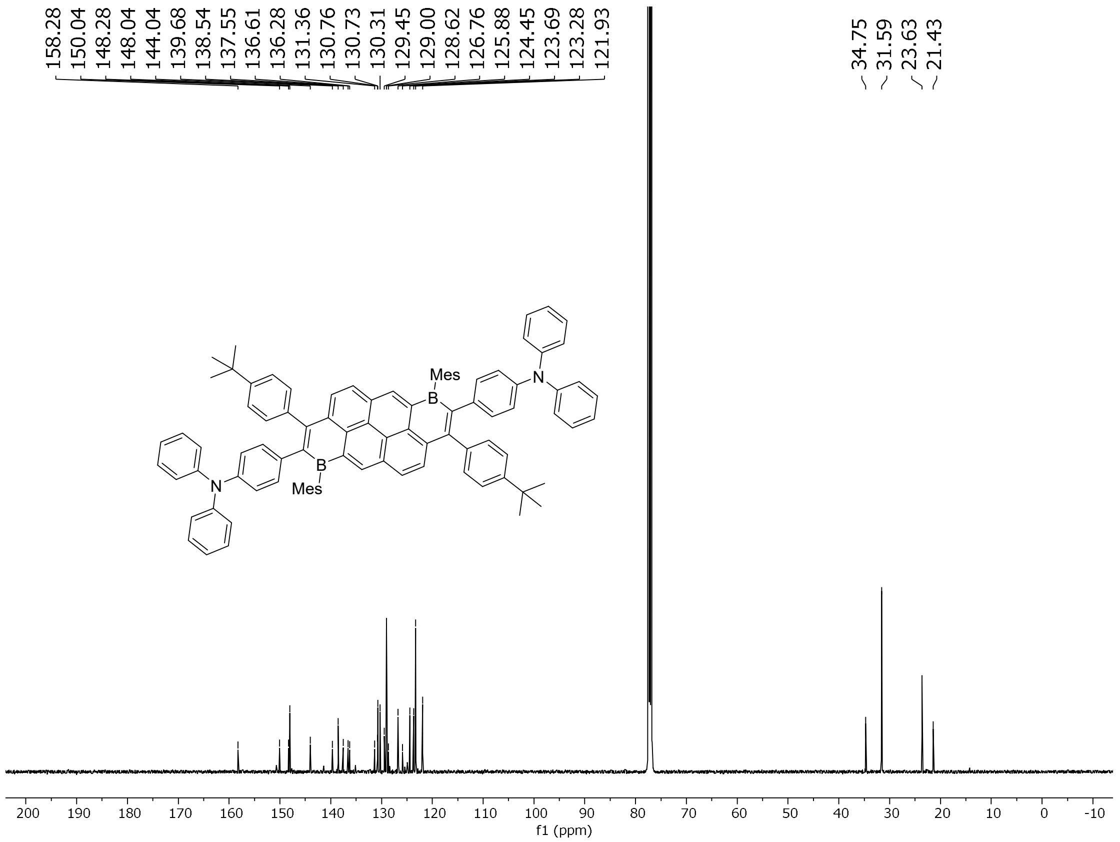

Figure S120. $^{13}C\{^{1}H\}$ NMR spectrum of **14** in $CDCl_3$.

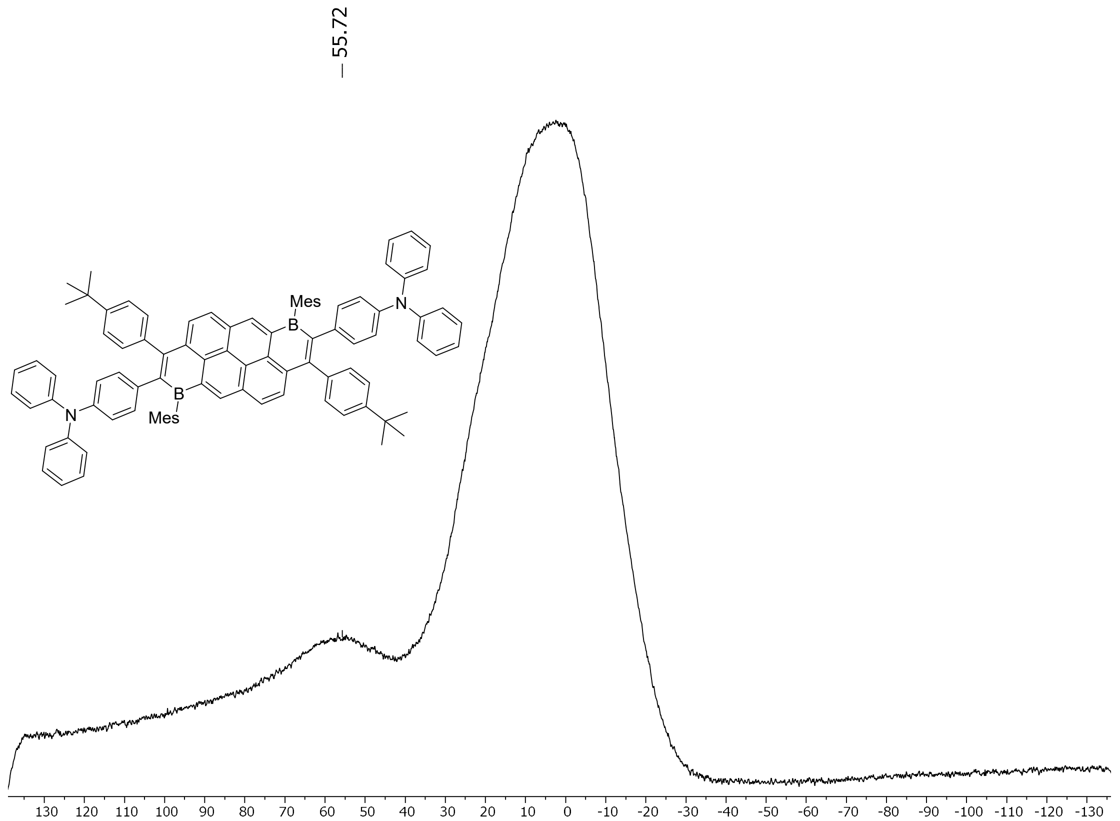

Figure S121. ^{11}B NMR spectrum of **14** in $CDCl_3$.

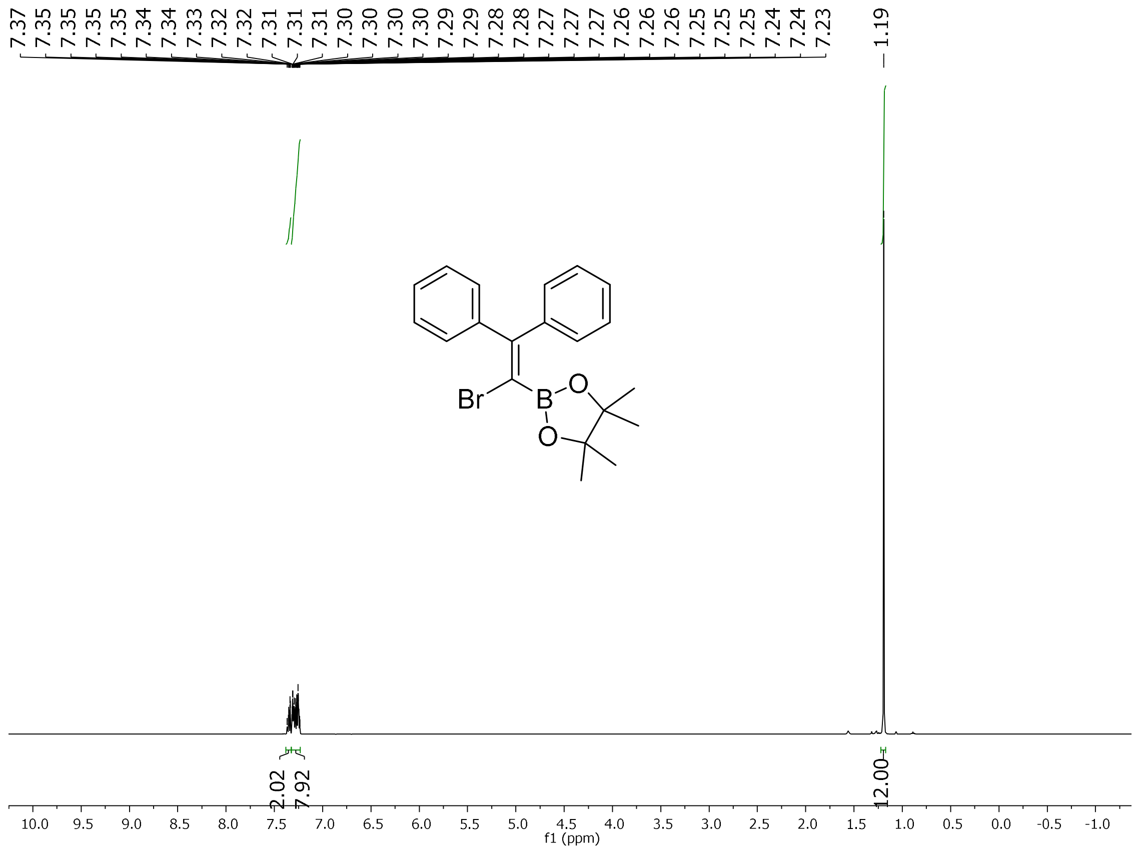

Figure S122. ^{1}H NMR spectrum of **8a** in $CDCl_3$

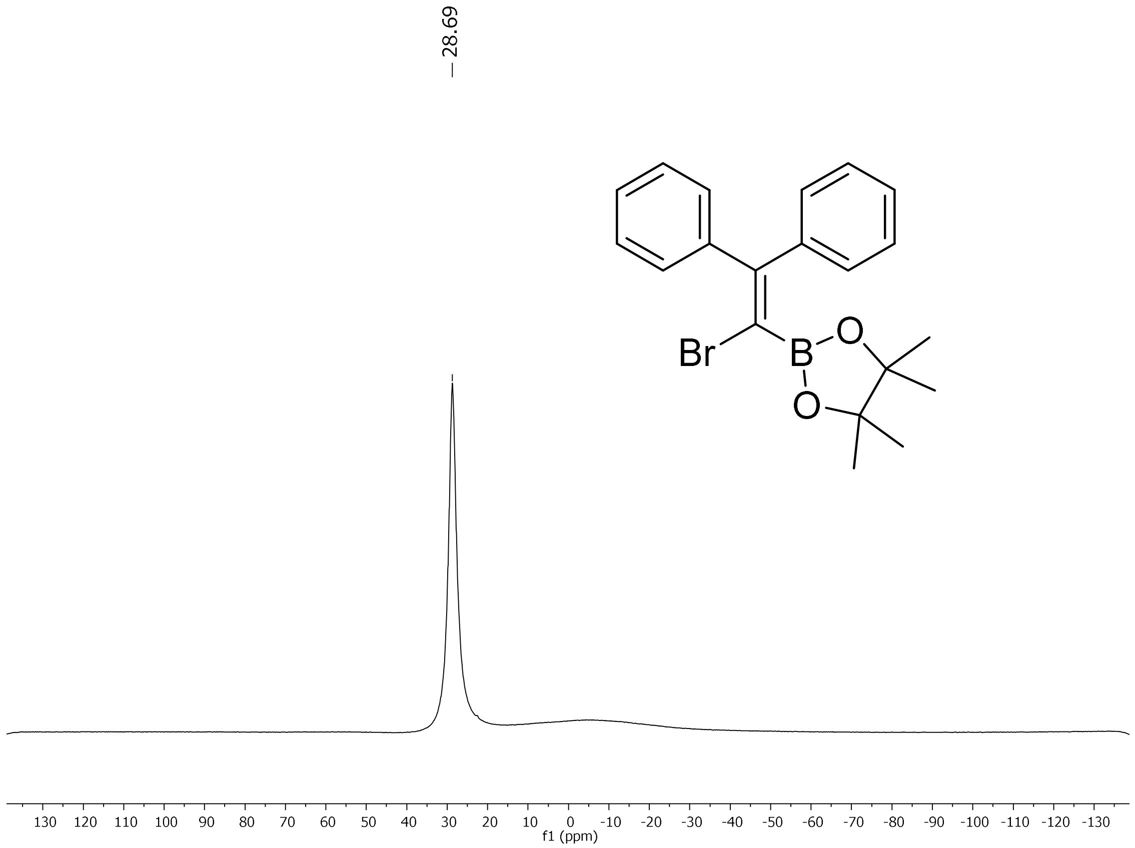

Figure S123. ^{11}B NMR spectrum of **8a** in $CDCl_3$.

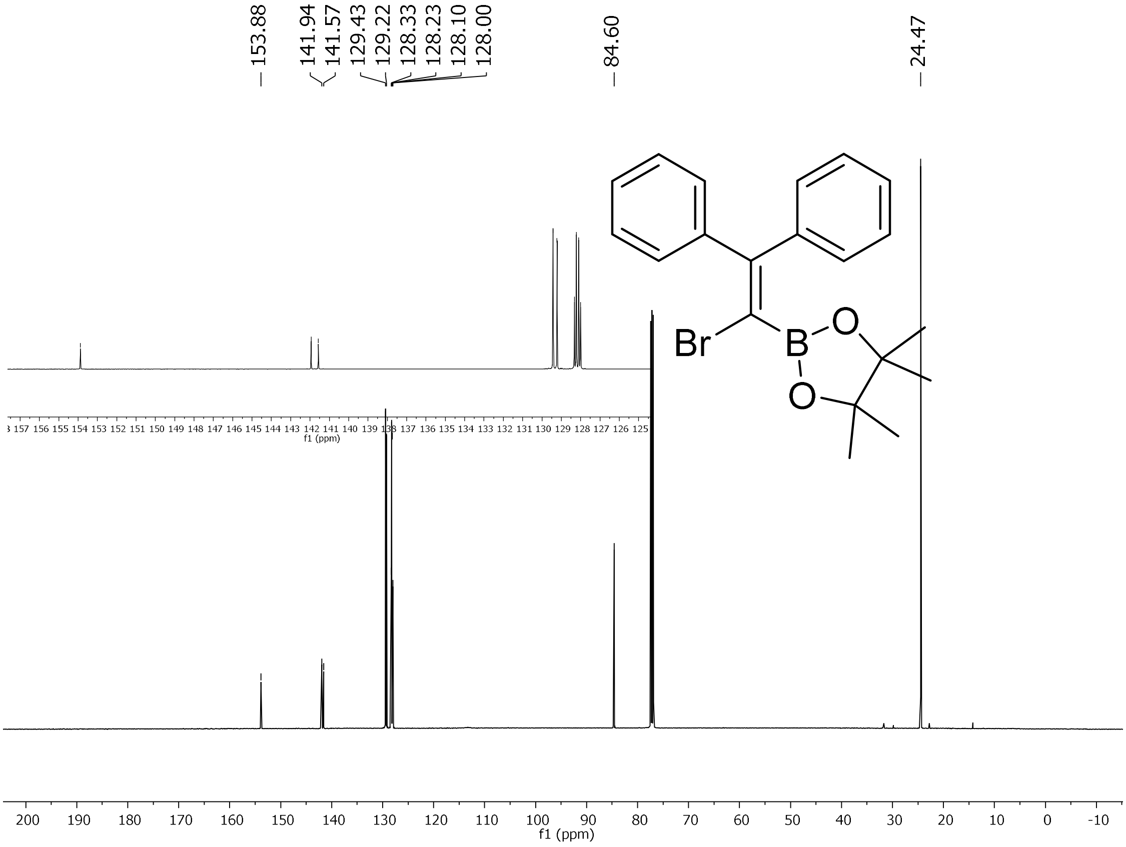

Figure S124. $^{13}C\{^1H\}$ NMR spectrum of **8a** in $CDCl_3$.

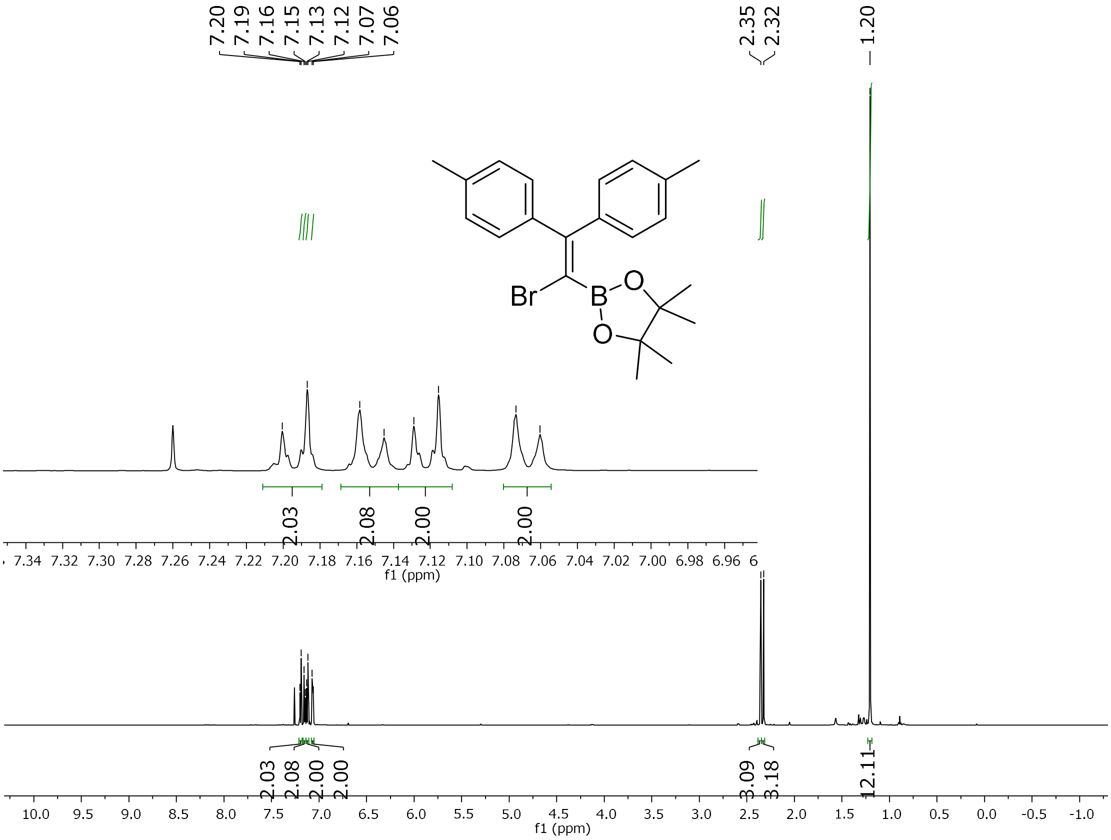

Figure S125. ^1H NMR spectrum of **8b** in $CDCl_3$.

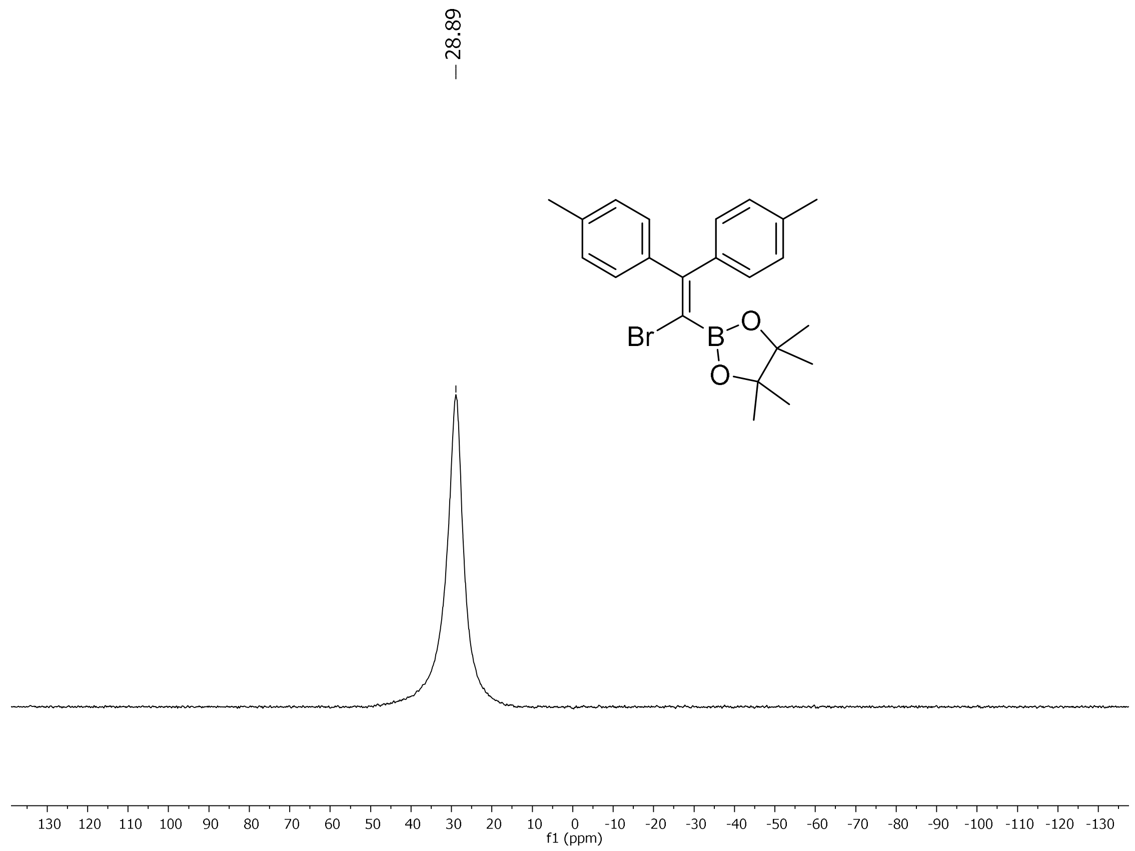

Figure S126. ^{11}B NMR spectrum of **8b** in $CDCl_3$.

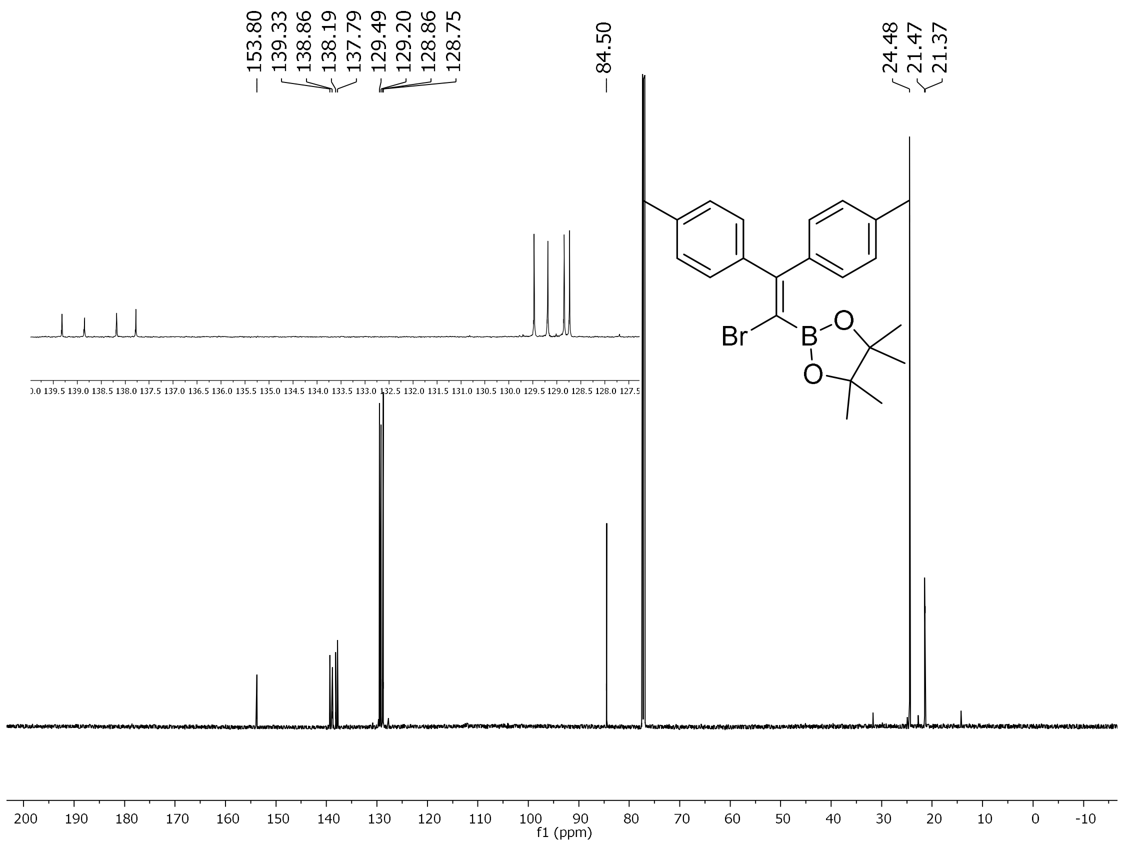

Figure S127. ^{13}C{^{1}H} NMR spectrum of **8b** in $CDCl_3$.

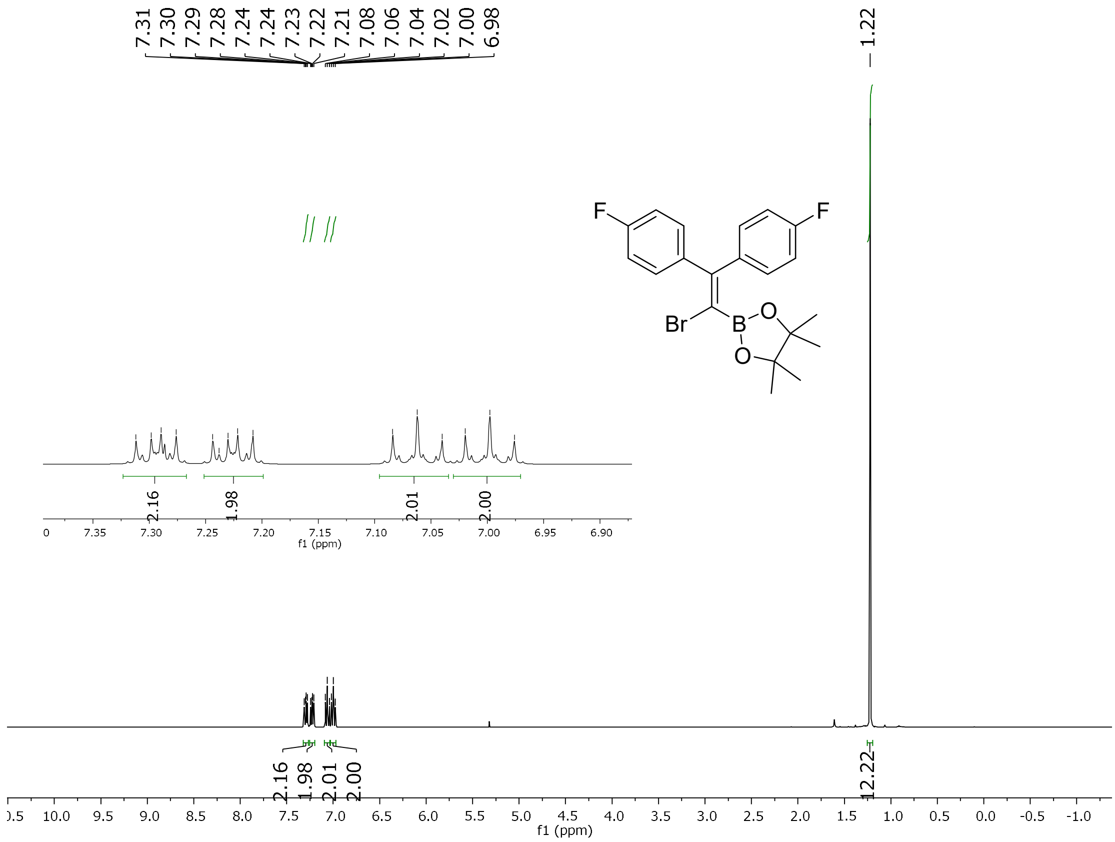

Figure S128. ^{1}H NMR spectrum of **8c** in $CDCl_3$.

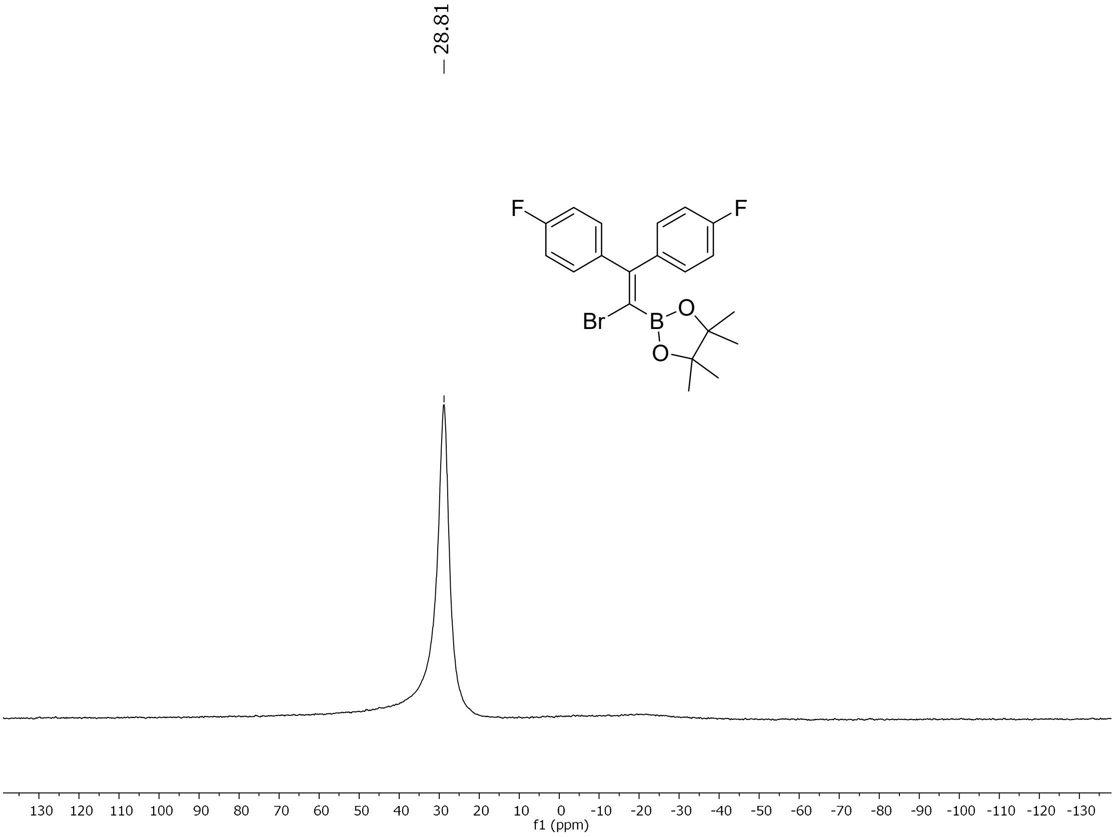

Figure S129. ^{11}B NMR spectrum of **8c** in $CDCl_3$.

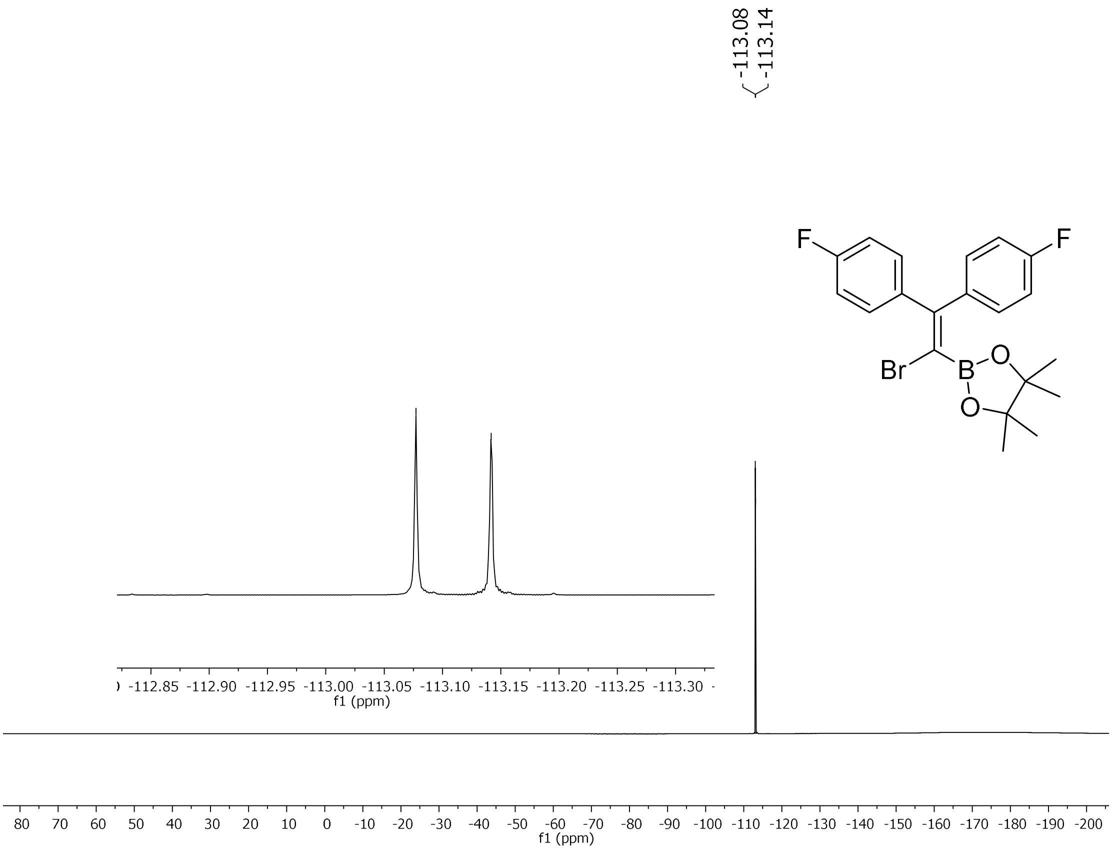

Figure S130. $^{19}F\{^1H\}$ NMR spectrum of **8c** in $CDCl_3$.

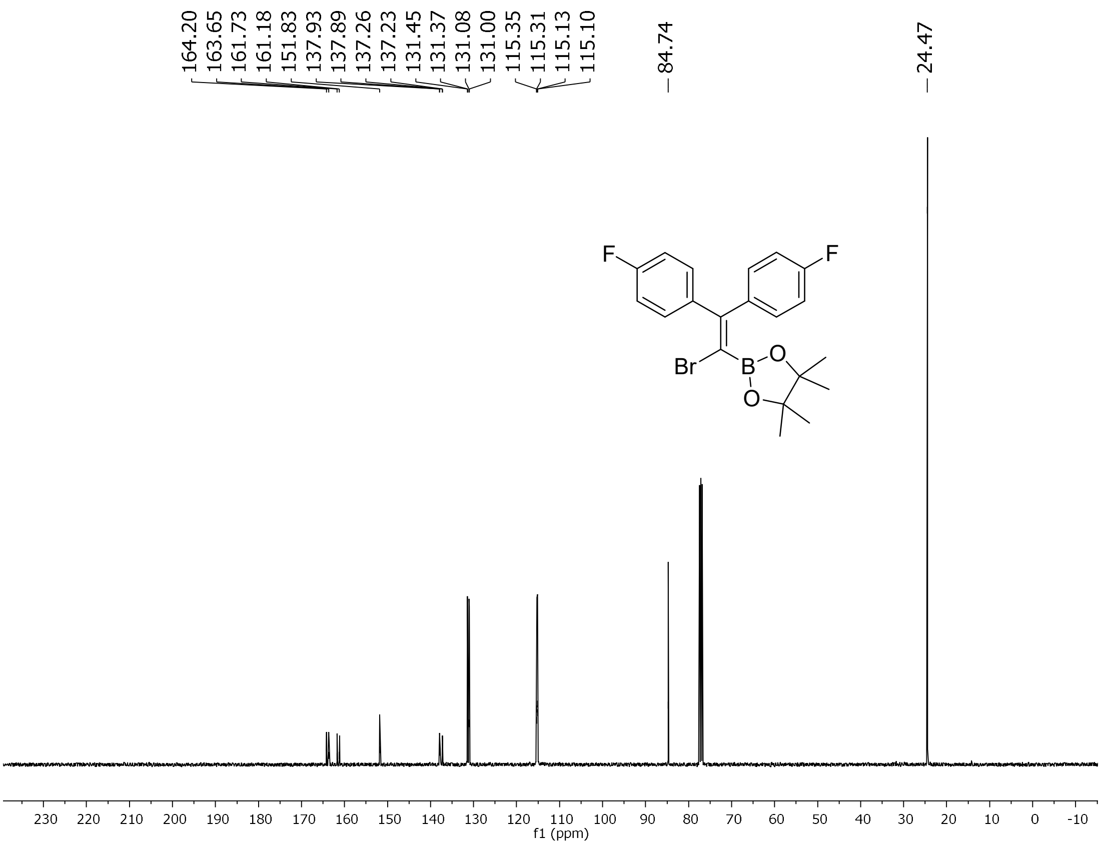

Figure S131. $^{13}C\{^1H\}$ NMR spectrum of **8c** in $CDCl_3$.

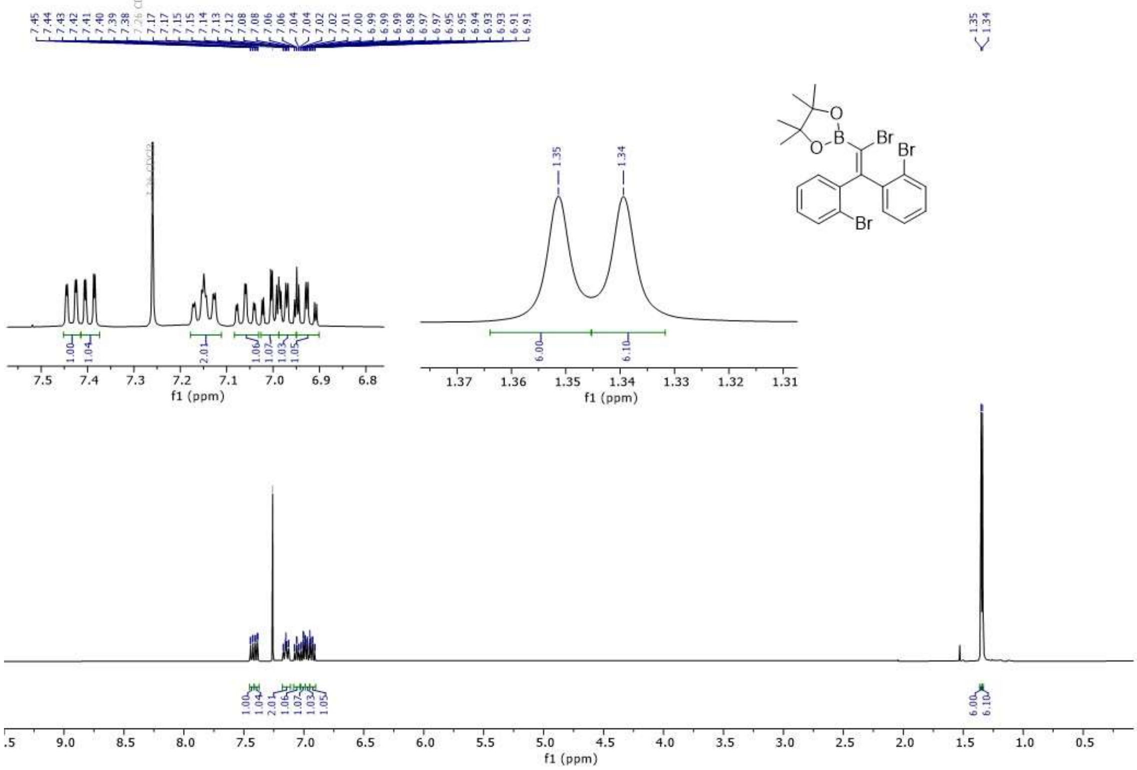

Figure S132: ^{1}H spectrum of **8d** in $CDCl_3$ after recrystallization from MeOH.

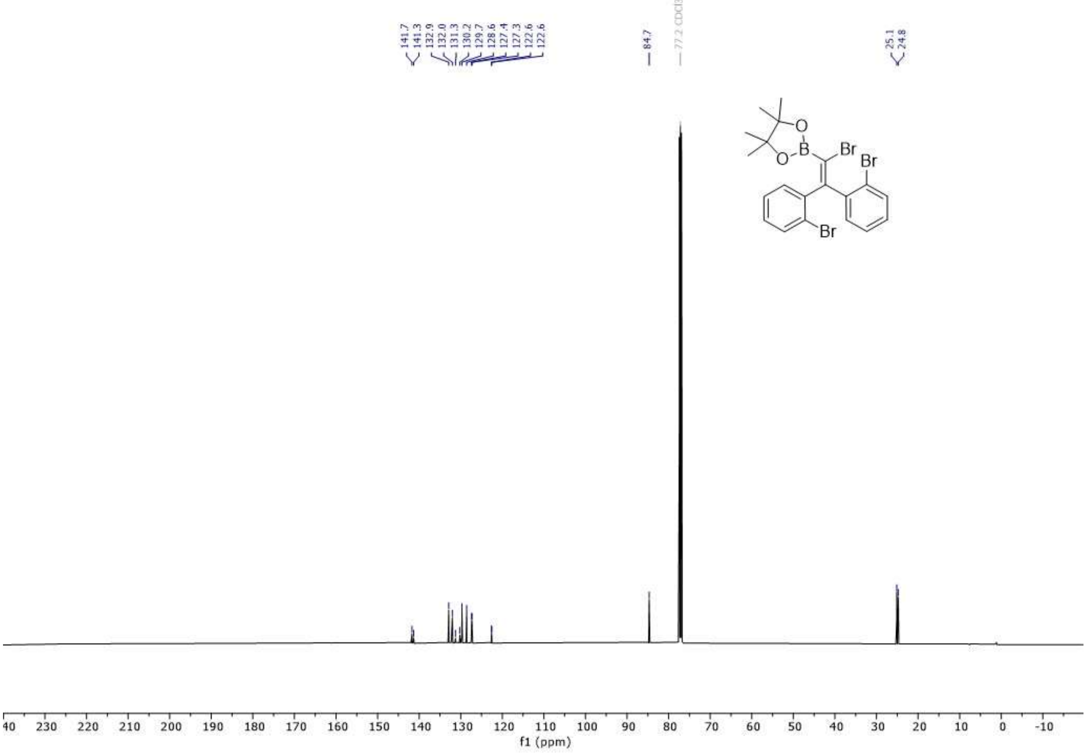

Figure S133: $^{13}C\{^{1}H\}$ NMR spectrum of **8d** in $CDCl_3$.

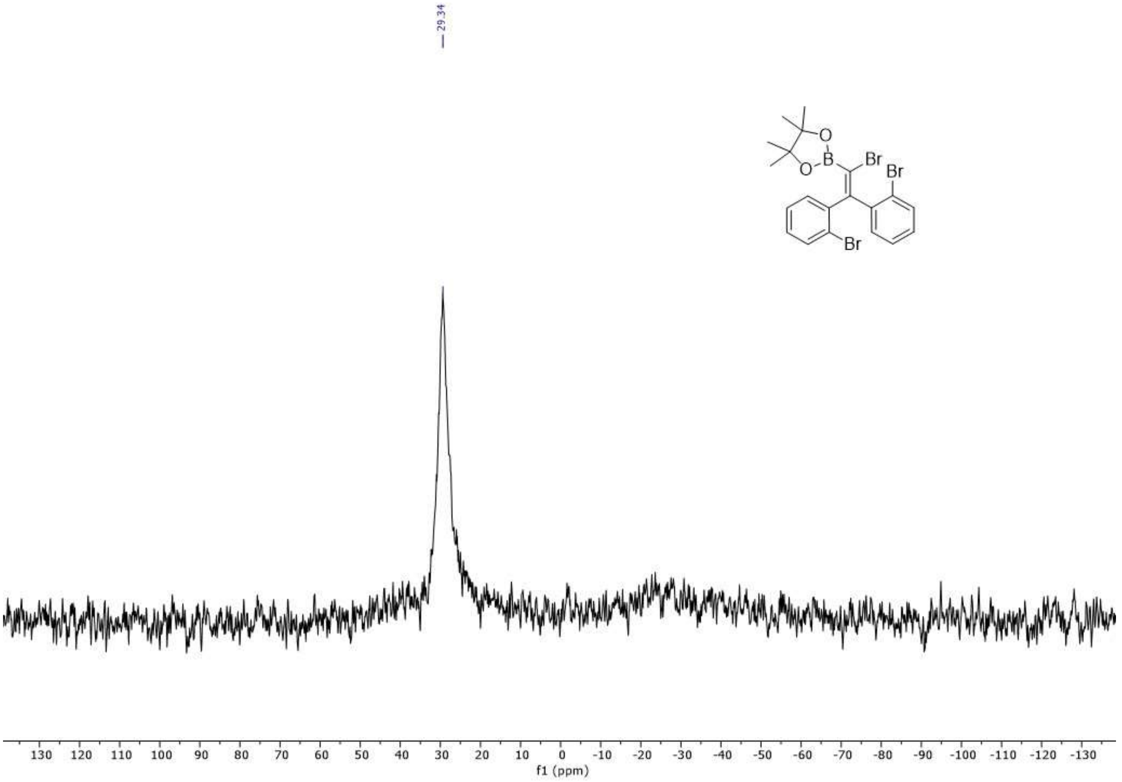

Figure S134: $^{11}B\{^{1}H\}$ NMR spectrum **8d** in $CDCl_3$.

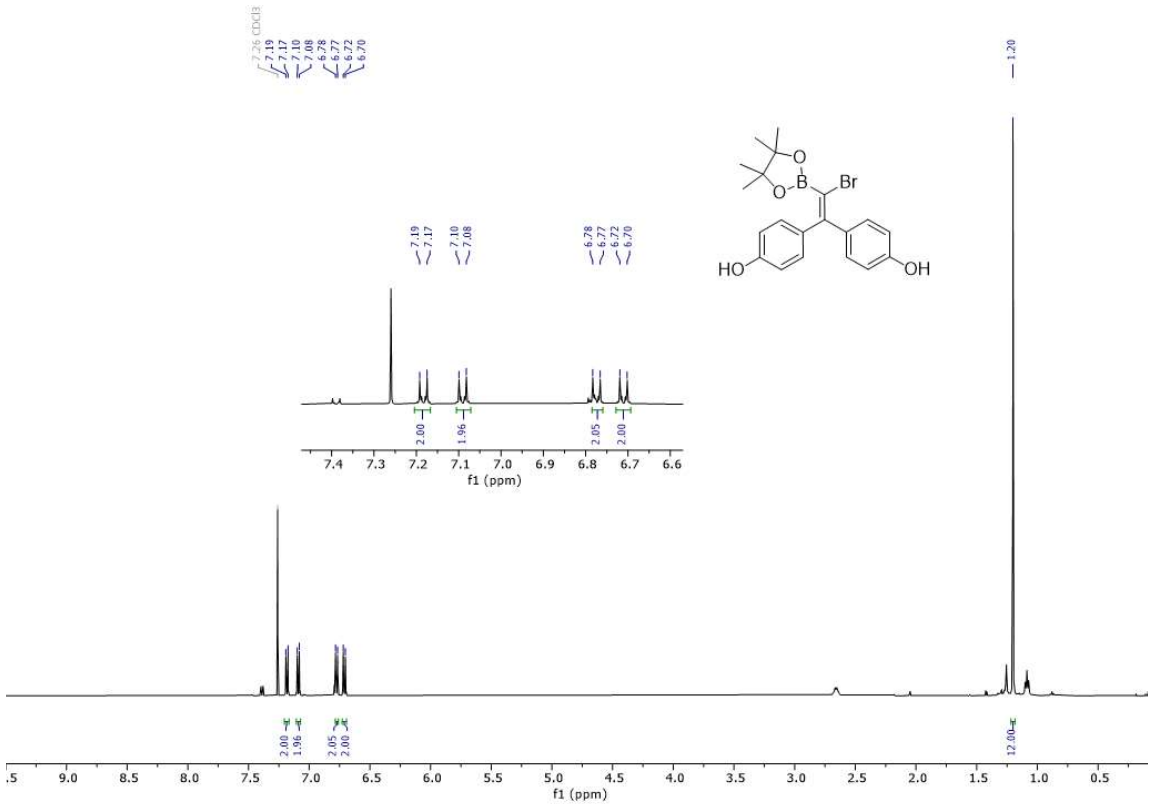

Figure S135: ^{1}H spectrum of **8e** in $CDCl_3$.

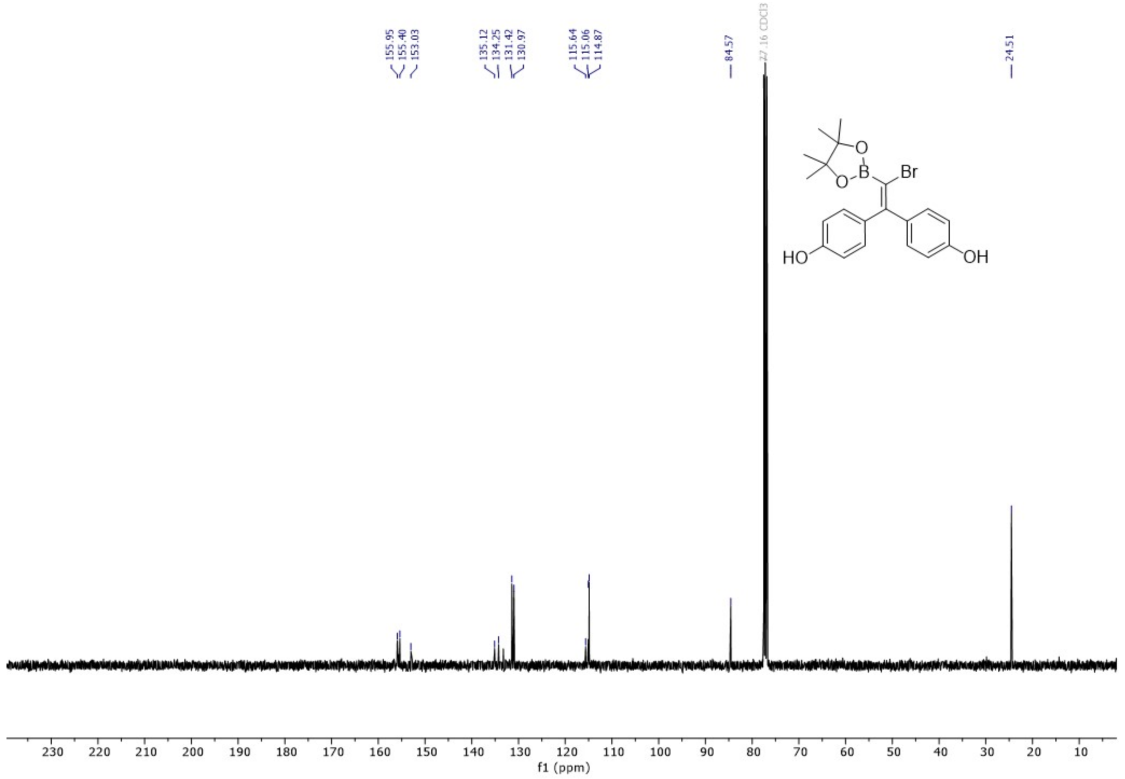

Figure S136: $^{13}C\{^1H\}$ NMR spectrum of **8e** in $CDCl_3$.

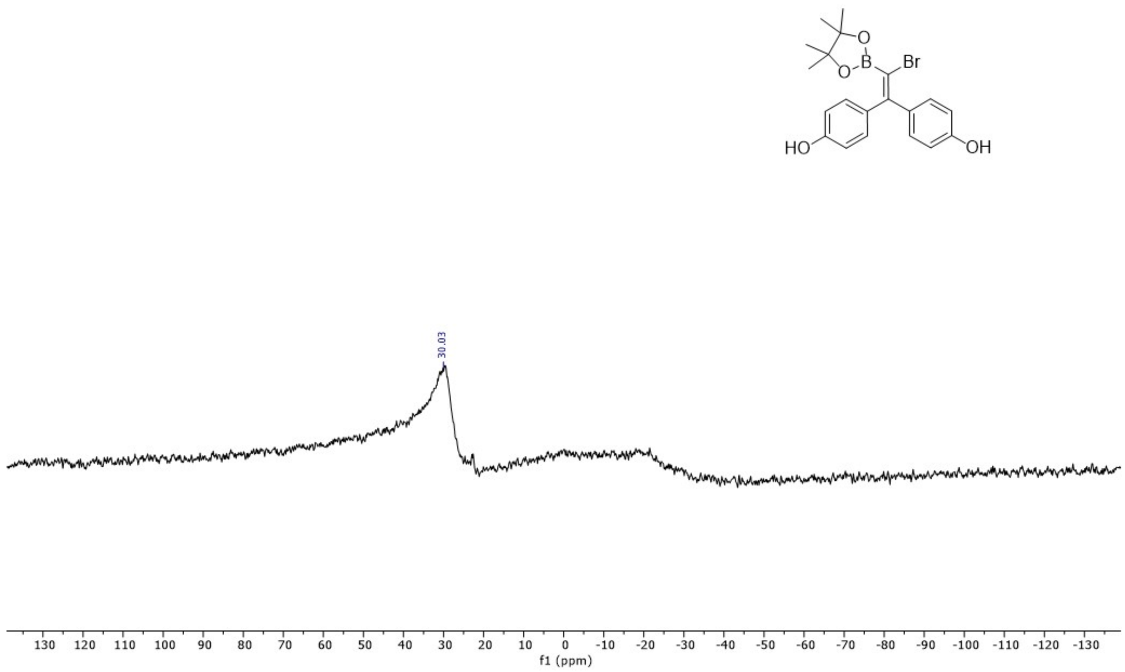

Figure S137: $^{11}B\{^1H\}$ NMR spectrum of **8e** in $CDCl_3$.

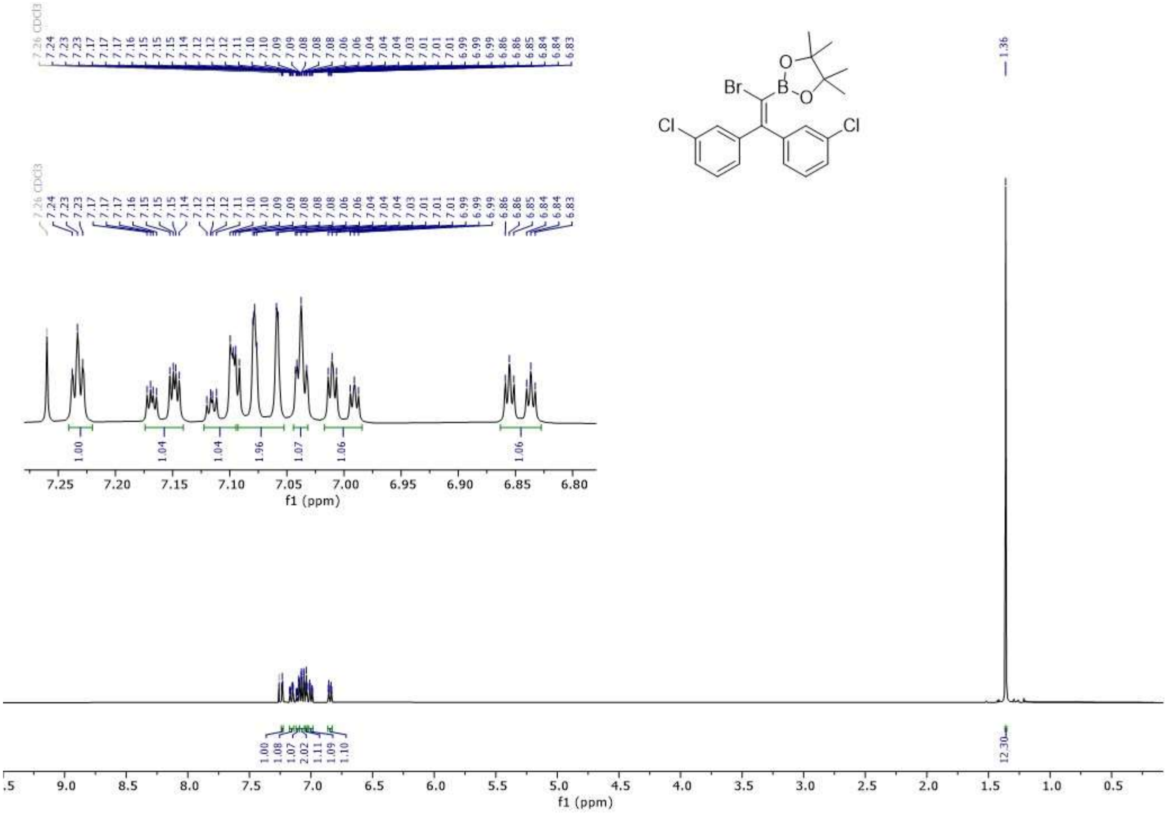

Figure S138: ^{1}H NMR spectrum of **8f** in $CDCl_3$.

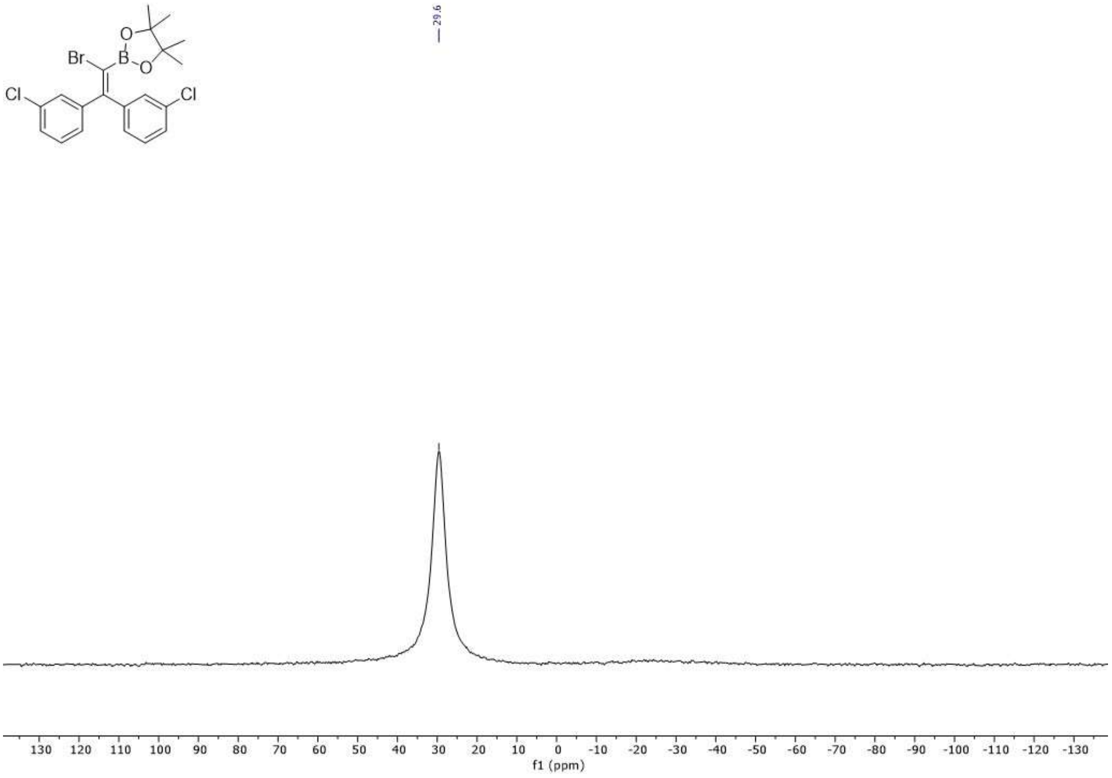

Figure S139: ^{11}B{^{1}H} NMR spectrum of **8f** in $CDCl_3$.

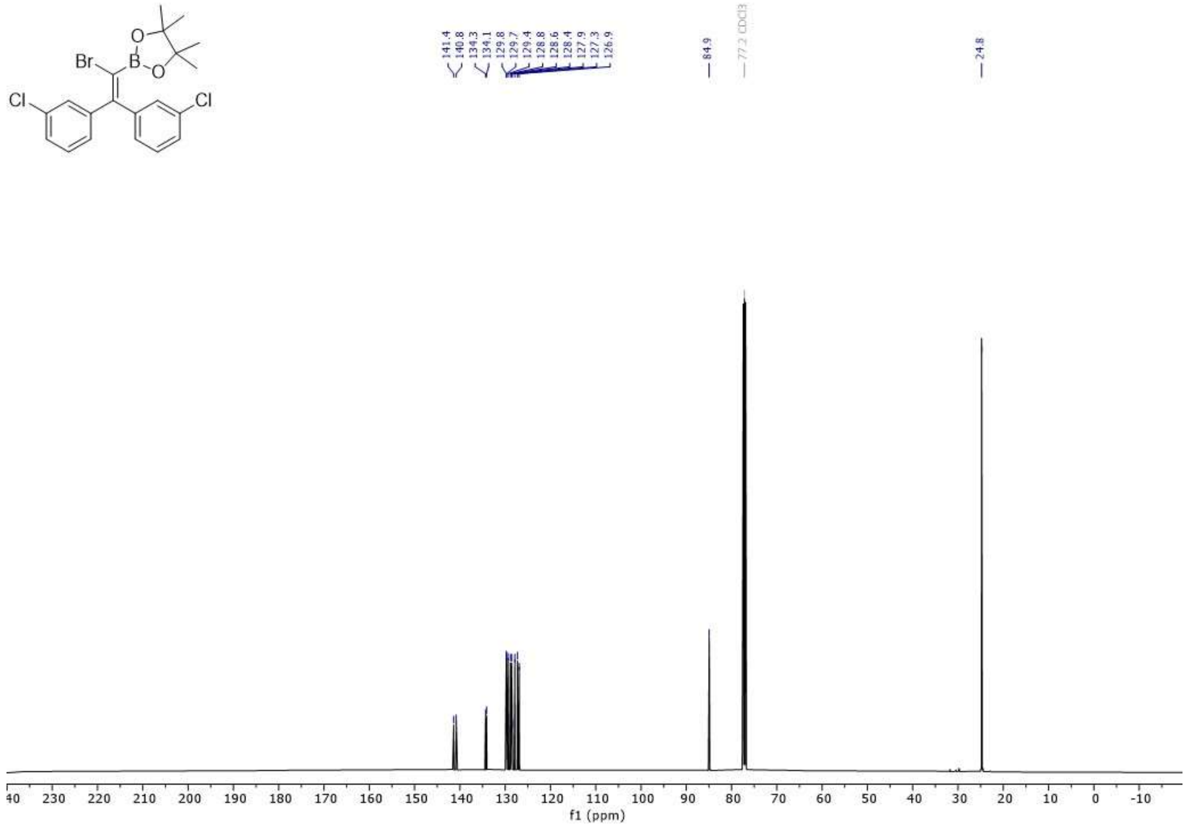

Figure S140: $^{13}C\{^{1}H\}$ NMR spectrum of **8f** in $CDCl_3$.

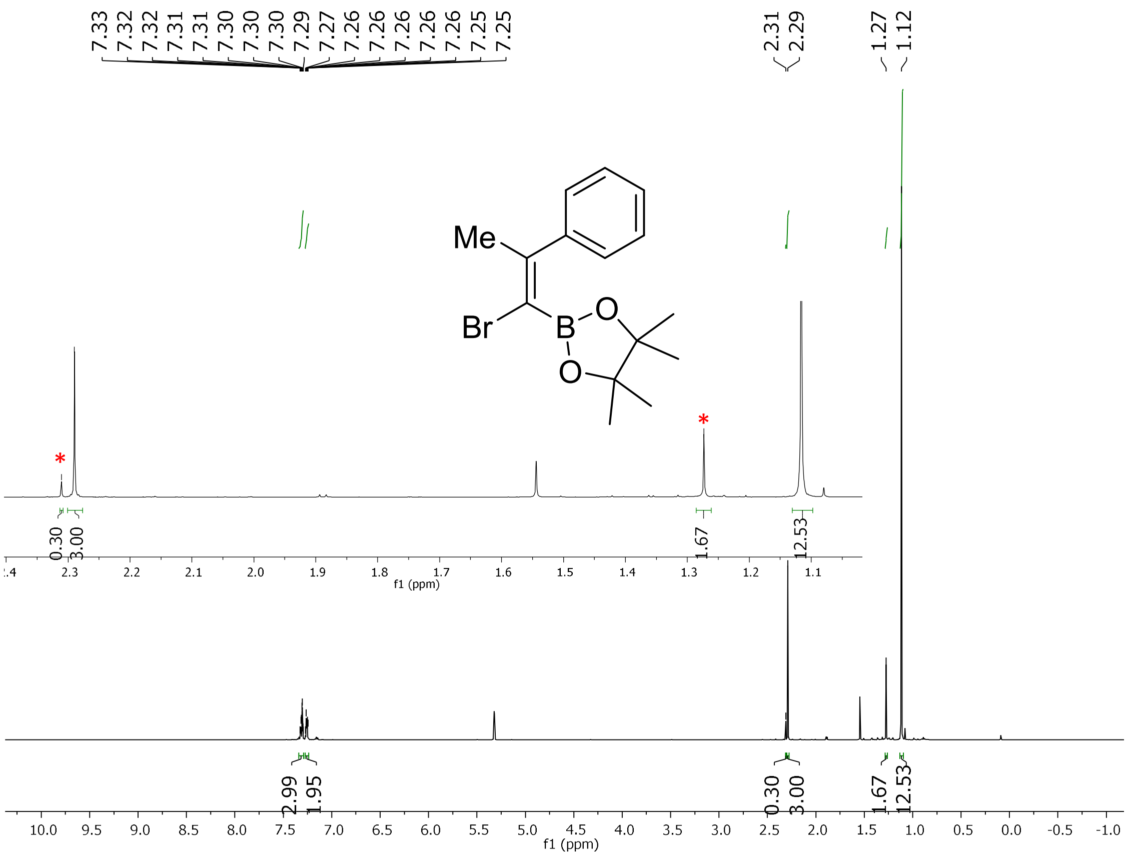

Figure S141. ^{1}H NMR spectrum of **8g** in $CDCl_3$. (* indicating a minor product presumably an isomer of **8g**)

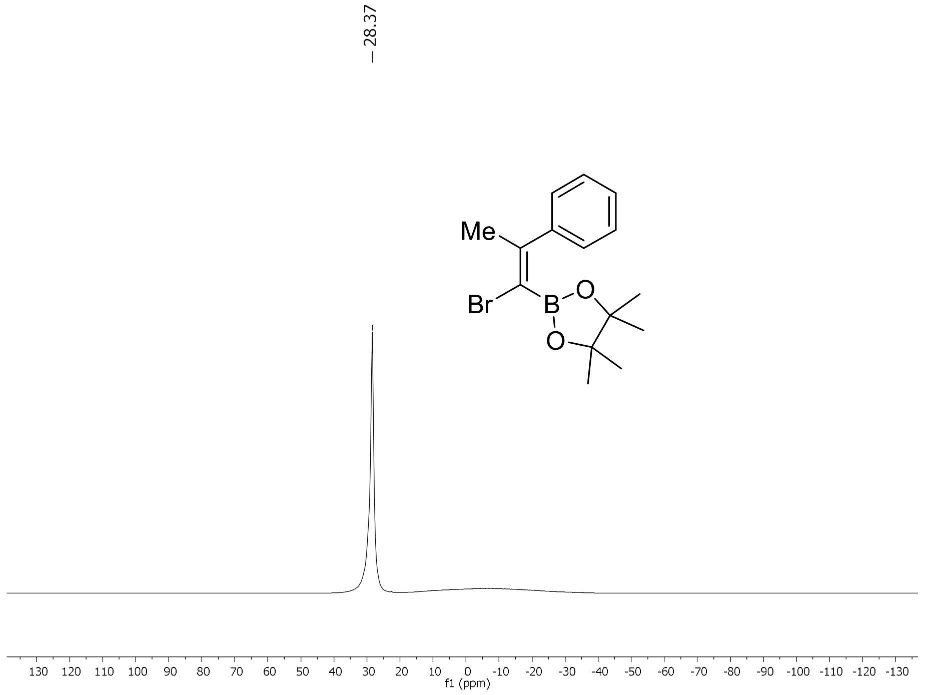

Figure S142. ^{11}B NMR spectrum of **8g** in $CDCl_3$.

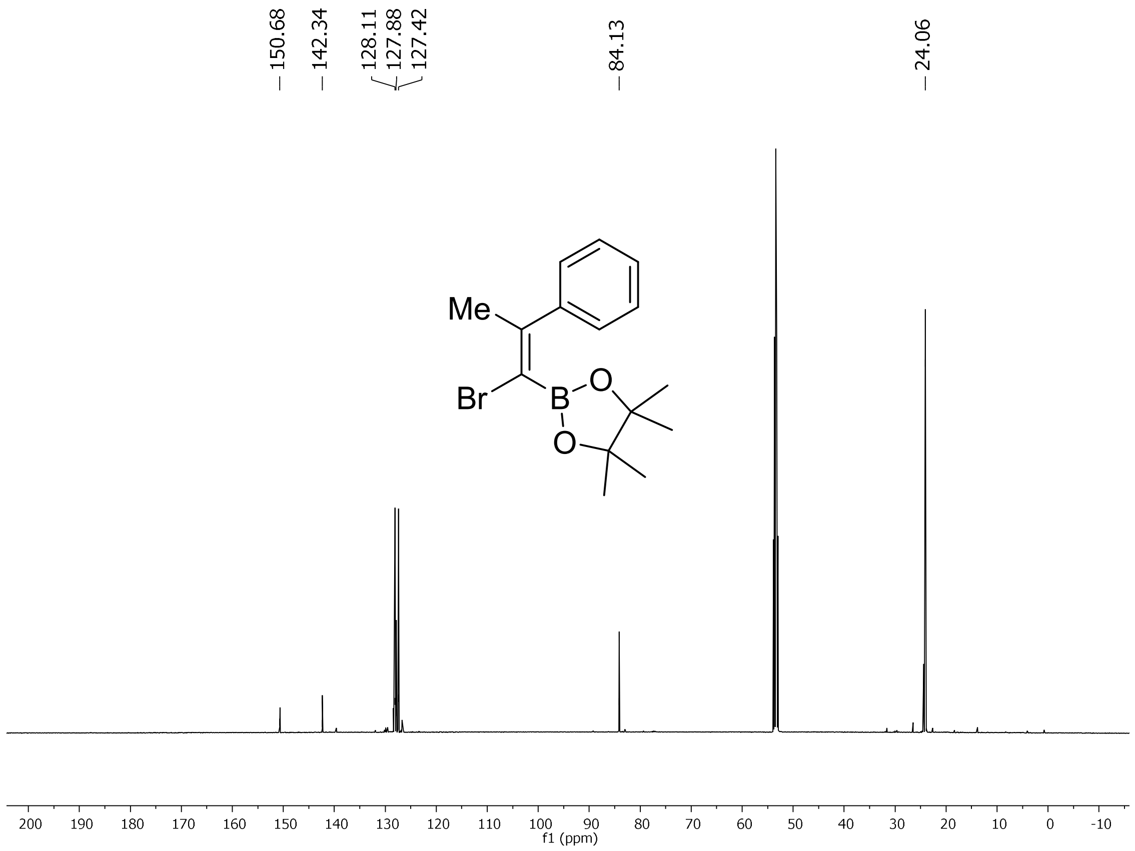

Figure S143. $^{13}C\{^{1}H\}$ NMR spectrum of **8g** in $CDCl_3$.

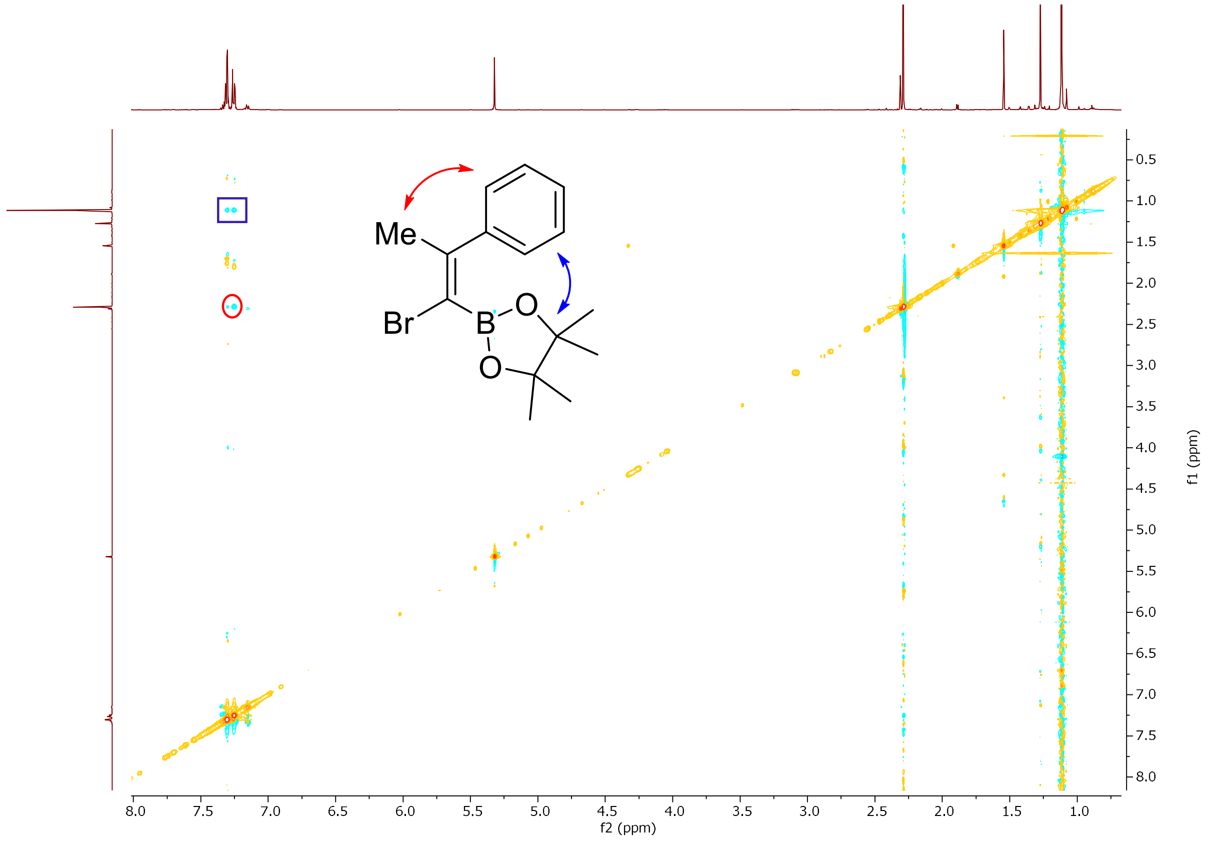

Figure S144. ^{1}H-^{1}H NOESY NMR spectrum of **8g** in $CDCl_3$.

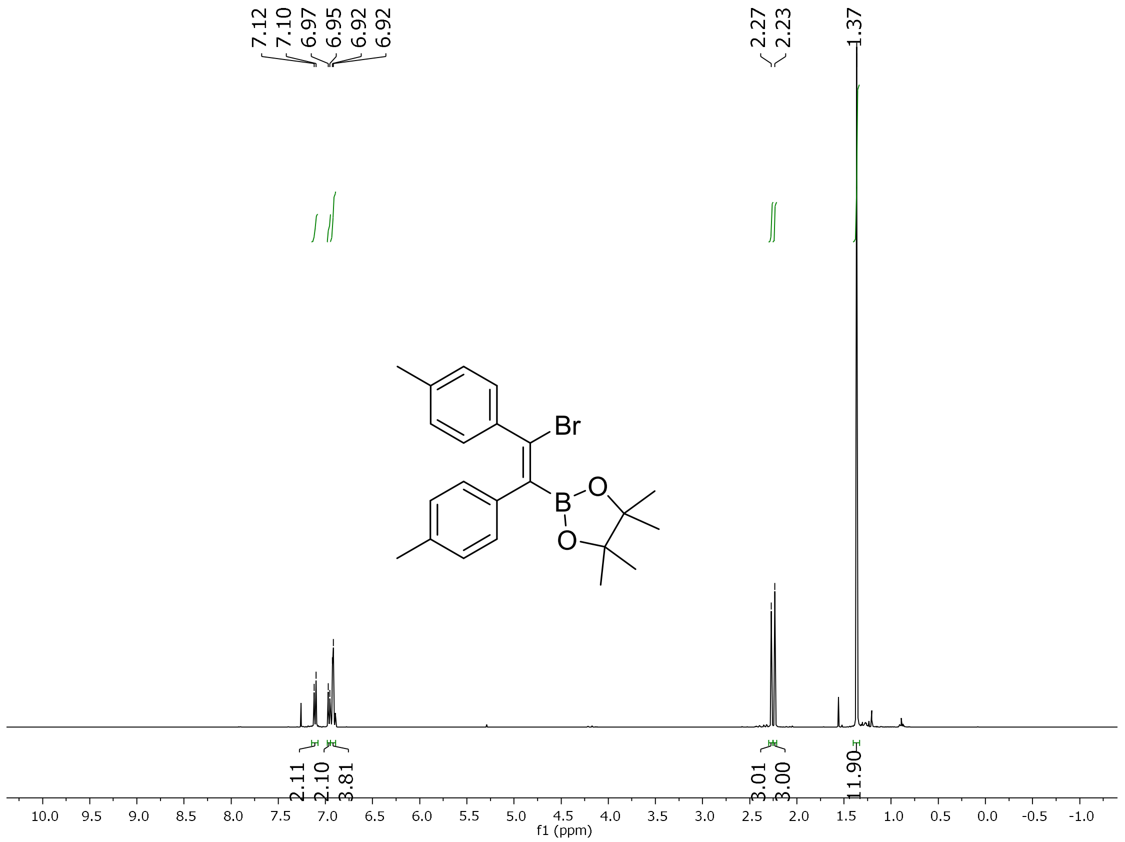

Figure S145. ^{1}H NMR spectrum of **8b-1,2** in $CDCl_3$.

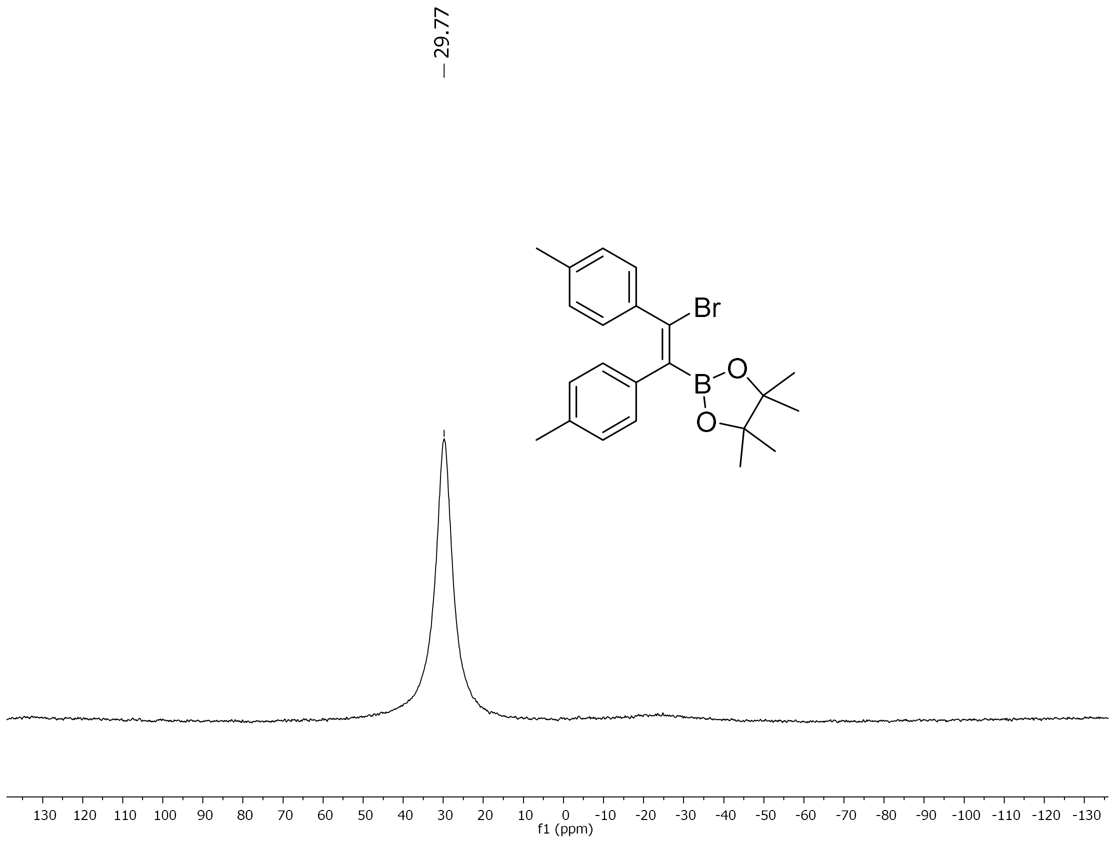

Figure S146. ^{11}B NMR spectrum of **8b-1,2** in $CDCl_3$.

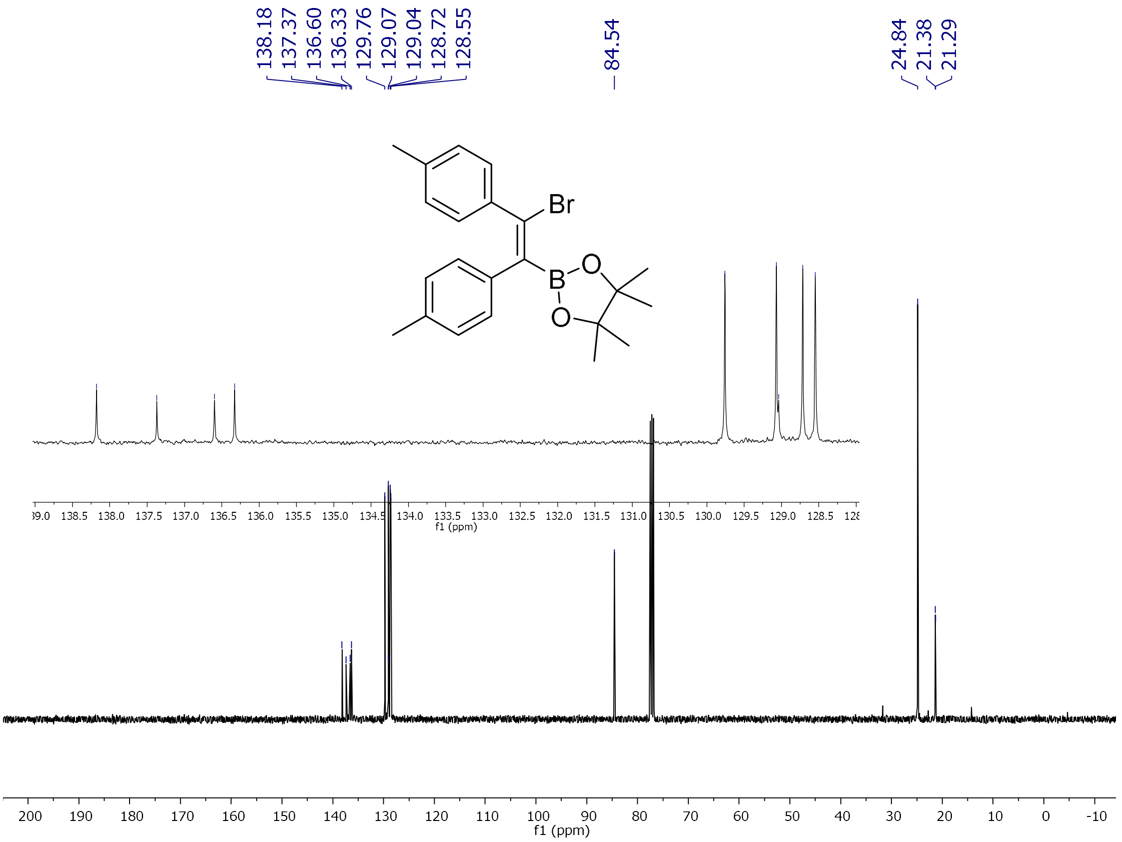

Figure S147. $^{13}C\{^{1}H\}$ NMR spectrum of **8b-1,2** in $CDCl_3$.

S8. Crystal Structures

Crystallographic data for compounds **1a, 3b, 8b,** and **12a** were recorded on an Agilent Supernova diffractometer with Mo Kα radiation (mirror monochromator, λ=0.7107) at 150 K (**1a**), 270K (**3b**) or at 120 K (**8b**, **12a**). Crystallographic data for compound **8e** were recorded on an Agilent Supernova diffractometer at 120 K with Cu Kα radiation (mirror monochromator, λ=1.54184). Crystallographic data for compounds **1c**, **2a** and **2c** were recorded on a dual source Rigaku FR-X rotating anode diffractometer equipped with a HyPix 6000 HE detector and an Oxford Cryostream 700 plus, at 150 K (**1c** and **2c**) or 100 K (**2a**) with Cu Kα radiation (mirror monochromator, λ = 1.54184). Crystallographic data for compound **4** were recorded on a Xcalibur, Eos diffractometer with Mo Kα radiation (mirror monochromator, λ=0.7107) at170 K. Crystallographic data for compound **9a** were recorded on a Bruker APEX-II CCD diffractometer, with Mo Kα radiation (mirror monochromator, λ=0.7107) at 120 K. The CrysAlisPro software package was used for data collection, cell refinement and data reduction. For all data sets the CrysAlisPro[6] software package was used for empirical absorption corrections, which were applied using spherical harmonics, implemented in SCALE3 ABSPACK scaling algorithm. For **1c**, the indexing of the collected data and frame inspections clearly showed twining signals. The data were processed with the twin/multicrystal routine of the CrysAlisPro software. All further data processing was undertaken within the Olex2 software package.[7] The molecular structures were solved with the

[6] CrysAlisPro, Agilent Technologies, Version 1.171.35.19 (release 27-10-2011 CrysAlis171 .NET) (compiled Oct 27 2011,15:02:11).

[7] Dolomanov, O. V.; Bourhis, L. J.; Gildea, R. J. ; Howard, J. A. K.; Puschmann, H. *J. Appl. Crystallogr.* **2009**, *42*, 339-341.

ShelXS[8] structure solution program using direct methods or ShelXT[9] structure solution program using Intrinsic Phasing and refined with the ShelXL[10] refinement package using Least Squares minimisation. Non-hydrogen atoms were refined anisotropically. Hydrogen atoms were all located in a difference map and repositioned geometrically. Structures **9a** and **12a** contain solvent voids with poorly defined features in the electron density map, which were modelled using a solvent mask calculated with Olex2. Crystals of sample **10a** were very weakly diffracting, therefore the data were truncated accordingly. Despite best efforts, better data could not be obtained and the data obtained were used only to confirm the connectivity. Selected crystallographic data are presented in Table S4 and full details in cif format can be obtained free of charge from the Cambridge Crystallographic Data Centre via www.ccdc.cam.uk/data_request/cif.

[8] Sheldrick, G. M. *Acta Cryst.* **2008**, *A64*, 112.
[9] Sheldrick, G. M. *Acta Cryst.* **2015**, *A71*, 3.
[10] Sheldrick, G. M. *Acta Cryst.* **2015**, *C71*, 3.

Table S4. Crystallographic data for compounds **1a, 1c** and **2a**.

	**1a**	**1c**	**2a**
CCDC No	1961365	1961366	1961367
Empirical formula	$C_{30}H_{26}BBr$	$C_{36}H_{36}BBr$	$C_{100}H_{108}B_4Br_4$
Formula weight	477.23	559.37	1672.74
Temperature (K)	150.0(3)	150.03(13)	99.8(5)
Radiation	0.71073	1.54184	1.54184
Crystal system	monoclinic	triclinic	triclinic
Space group	P 2_1/c	P -1	P -1
a (Å)	16.2062(11)	8.4803(5)	8.0182(3)
b (Å)	8.1904(4)	9.1672(5)	16.5443(7)
c (Å)	17.1370(7)	19.9099(11)	17.4491(8)
α (°)	90	80.254(4)	64.933(4)
β (°)	91.218(5)	85.195(5)	86.117(3)
γ (°)	90	68.593(5)	77.027(3)
Cell Volume ($Å^3$)	2274.2(2)	1419.82(14)	2042.19(16)
Z	4	2	1
ρ_{calc} (g/cm^3)	1.394	1.308	1.360
μ (mm^{-1})	1.824	2.127	2.763
F(000)	984.0	584.0	868.0
Crystal size (mm^3)	0.3 × 0.3 × 0.01	0.073 x 0.027 x 0.013	0.075 × 0.058 × 0.022
2Θ range for data collection (°)	5.512 to 58.262	4.504 to 152.552	5.594 to 136.49
Index ranges	$-16 \leq h \leq 21$, $-10 \leq k \leq 10$, $-22 \leq l \leq 23$	$-10 \leq h \leq 10$, $-11 \leq k \leq 11$, $-25 \leq l \leq 24$	$-9 \leq h \leq 8$, $-19 \leq k \leq 19$, $-20 \leq l \leq 20$
Reflections collected	12886	9463	22412
Independent reflections	5322 [R_{int} = 0.0512, R_{sigma} = 0.0780]	9463[R_{int} = 0.0590, R_{sigma} = 0.0428]	7310 [R_{int} = 0.0441, R_{sigma} = 0.0511]
Data/restraints/parameters	5322/0/293	9463/0/351	7310/225/541
Goodness-of-fit on F^2 (GoF)	1.018	1.068	1.044
Final R indices [I>=2σ (I)]	R_1 = 0.0516, wR_2 = 0.1045	R_1 = 0.0442, wR_2 = 0.1190	R_1 = 0.0457, wR_2 = 0.1204
Final R indices [all data]	R_1 = 0.0994, wR_2 = 0.1251	R_1 = 0.0551, wR_2 = 0.1259	R_1 = 0.0552, wR_2 = 0.1272
Largest diff. peak/hole (e $Å^{-3}$)	0.54/-0.54	0.65/-0.60	0.95/-1.45

Table S5. Crystallographic data for compounds **2c, 3b** and **4**.

	**2c**	**3b**	**4**
CCDC No	1961368	1961369	1961370
Empirical formula	$C_{64}H_7B_2Br_2$	$C_{28}H_{22}BBrO$	$C_{14}H_{10}BBr_3$
Formula weight	1028.70	465.17	428.76
Temperature (K)	150.03(10)	270.00(10)	170.00(20)
Radiation	1.54184	0.71073	0.71073
Crystal system	triclinic	monoclinic	monoclinic
Space group	P -1	P 2_1/c	P 2_1/n
a (Å)	9.4752(4)	16.0697(11)	13.2781(3)
b (Å)	9.8107(4)	19.1015(11)	5.93600(10)
c (Å)	15.1088(6)	7.1840(5)	18.4485(4)
α (°)	93.579(3)	90	90
β (°)	100.145(3)	99.697(6)	100.580(2)
γ (°)	90.470(3)	90	90
Cell Volume (Å^3)	1379.57(10)	2173.7(2)	1429.37(5)
Z	1	4	4
ρ_{calc} (g/cm^3)	1.238	1.421	1.992
μ (mm^{-1})	2.137	1.909	8.444
F(000)	542.0	952.0	816.0
Crystal size (mm^3)	0.08 x 0.05 x 0.02	0.39 × 0.106 × 0.061	0.538 × 0.157 × 0.122
2Θ range for data collection (°)	5.956 to 152.84	7.018 to 57.948	6.888 to 61.014
Index ranges	$-10 \leq h \leq 11$, $-12 \leq k \leq 12$, $-18 \leq l \leq 19$	$-21 \leq h \leq 19$, $-18 \leq k \leq 24$, $-8 \leq l \leq 9$	$-18 \leq h \leq 18$, $-8 \leq k \leq 8$, $-26 \leq l \leq 26$
Reflections collected	18796	18623	41168
Independent reflections	5521 [R_{int} = 0.0415, R_{sigma} = 0.0424]	4863 [R_{int} = 0.0472, R_{sigma} = 0.0538]	4355 [R_{int} = 0.0595, R_{sigma} = 0.0316]
Data/restraints/parameters	5521/0/305	4863/18/284	4355/0/163
Goodness-of-fit on F^2 (GoF)	1.118	1.065	1.124
Final R indices [I>=2σ (I)]	R_1 = 0.0376, wR_2 = 0.0921	R_1 = 0.0513, wR_2 = 0.0946	R_1 = 0.0309, wR_2 = 0.0669
Final R indices [all data]	R_1 = 0.0426, wR_2 = 0.0954	R_1 = 0.0908, wR_2 = 0.1080	R_1 = 0.0453, wR_2 = 0.0716
Largest diff. peak/hole (e Å^{-3})	0.34/-0.52	0.49/-0.40	0.64/-1.13

Table S6. Crystallographic data for compounds **8b, 8e** and **9a**.

	**8b**	**8b-1,2**	**9a**
CCDC No	1961371	1961372	1961373
Empirical formula	$C_{22}H_{26}BBrO_2$	$C_{22}H_{26}BBrO_2$	$C_{58}H_{54}B_2Br_2$
Formula weight	413.15	413.15	1018.62
Temperature (K)	120.01(10)	120.01(10)	120(2)
Radiation	0.71073	1.54184	0.71073
Crystal system	monoclinic	orthorhombic	Triclinic
Space group	$P\ 2_1/n$	$P\ ca2_1$	P -1
a (Å)	6.4265(4)	20.7096(3)	13.450(3)
b (Å)	15.8695(10)	6.43840(10)	14.624(3)
c (Å)	20.6170(11)	15.5735(2)	15.214(3)
α (°)	90	90	91.36(3)
β (°)	97.511(5)	90	92.15(3)
γ (°)	90	90	116.43(3)
Cell Volume ($Å^3$)	2084.6(2)	2076.52(5)	2675.1(11)
Z	4	4	2
ρ_{calc} (g/cm^3)	1.316	1.322	1.265
μ (mm^{-1})	1.984	2.782	1.554
F(000)	856.0	856.0	1064.0
Crystal size (mm^3)	0.367 × 0.110 × 0.061	0.367 × 0.205 × 0.046	0.211 x 0.13 x 0.124
2Θ range for data collection (°)	6.444 to 54.966	8.54 to 151.766	4.378 to 52.796
Index ranges	$-7 \le h \le 8$, $-20 \le k \le 20$, $-24 \le l \le 26$	$-26 \le h \le 25$, $-4 \le k \le 7$, $-18 \le l \le 19$	$-16 \le h \le 16$, $-18 \le k \le 18$, $-19 \le l \le 19$
Reflections collected	18476	12887	82876
Independent reflections	4679 [R_{int} = 0.0483, R_{sigma} = 0.0524]	3824 [R_{int} = 0.0516, R_{sigma} = 0.0341]	10942 [R_{int} = 0.0483, R_{sigma} = 0.0344]
Data/restraints/parameters	4679/172/300	3824/1/253	10942/156/599
Goodness-of-fit on F^2 (GoF)	1.069	1.046	1.032
Final R indices [I>=2σ (I)]	R_1 = 0.0481, wR_2 = 0.0809	R_1 = 0.0438, wR_2 = 0.1086	R_1 = 0.0668, wR_2 = 0.1815
Final R indices [all data]	R_1 = 0.0689, wR_2 = 0.0877	R_1 = 0.0440, wR_2 = 0.1089	R_1 = 0.0889, wR_2 = 0.1948
Largest diff. peak/hole (e $Å^{-3}$)	0.72/-0.77	0.61/-0.99	1.03/-1.16
Flack parameter	-	-0.03(2)	-

Table S7. Crystallographic data for compound **12a**.

	**10b**	**12a**
CCDC No	1961374	1961375
Empirical formula	$C_{44}H_{42}B_2Br_2O_2$	$C_{62}H_{56}B_2Br_2$
Formula weight	784.21	982.50
Temperature (K)	120.01(10)	120.00(10)
Radiation	1.54184	0.71073
Crystal system	Orthorhombic	Triclinic
Space group	P ccn	P -1
a (Å)	42.071(3)	9.836(2)
b (Å)	29.3977(10)	10.894(2)
c (Å)	6.6541(2)	14.514(3)
α (°)	90	82.02(3)
β (°)	90	73.02(3)
γ (°)	90	71.06(3)
Cell Volume (Å^3)	8229.7(6)	1405.1(6)
Z	8	1
ρ_{calc} (g/cm^3)	1.266	1.161
μ (mm^{-1})	2.748	1.478
F(000)	3216.0	508.0
Crystal size (mm^3)	0.293 x 0.065 x 0.027	0.257 x 0.045 x 0.038
2Θ range for data collection (°)	6.984 to 100.866	6.506 to 51.36
Index ranges	$-41 \le h \le 42$, $-29 \le k \le 26$, $-6 \le l \le 6$	$-11 \le h \le 11$, $-13 \le k \le 13$, $-17 \le l \le 17$
Reflections collected	41818	22003
Independent reflections	4320 [R_{int} = 0.1415, R_{sigma} = 0.0711]	5328 [R_{int} = 0.0816, R_{sigma} = 0.0869]
Data/restraints/parameters	4320/0/462	5328/18/325
Goodness-of-fit on F^2 (GoF)	1.129	1.020
Final R indices [I>=2σ (I)]	R_1 = 0.0907, wR_2 = 0.2404	R_1 = 0.0698, wR_2 = 0.1328
Final R indices [all data]	R_1 = 0.1173, wR_2 = 0.2580	R_1 = 0.1034, wR_2 = 0.1467
Largest diff. peak/hole (e Å^{-3})	1.32/-0.78	1.16/-0.70

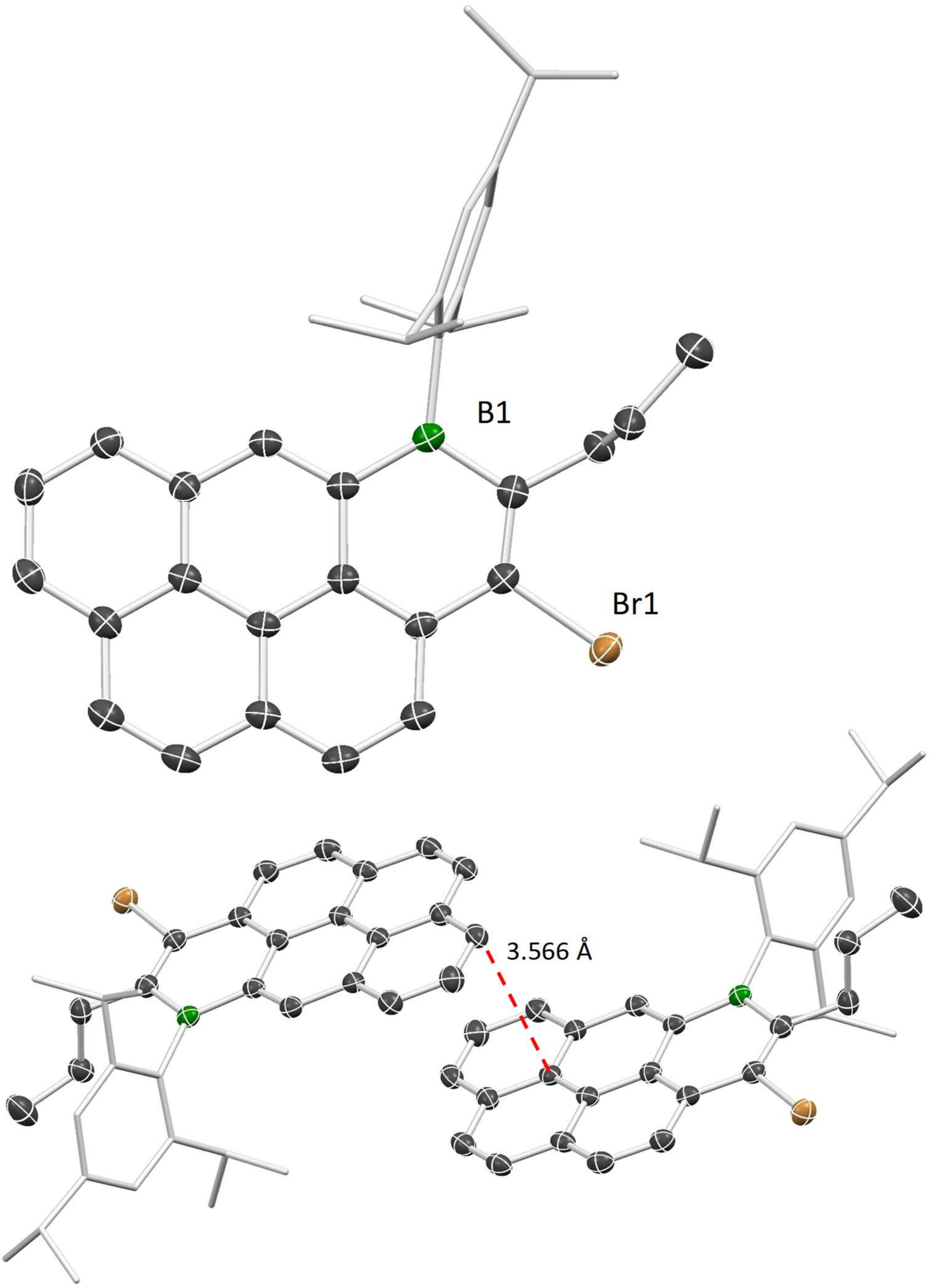

Figure S148. Top: solid-state structures of **1c**; bottom: extended packing structure of **1c**. Hydrogen atoms were omitted for clarity. Ellipsoids at the 50% probability level.

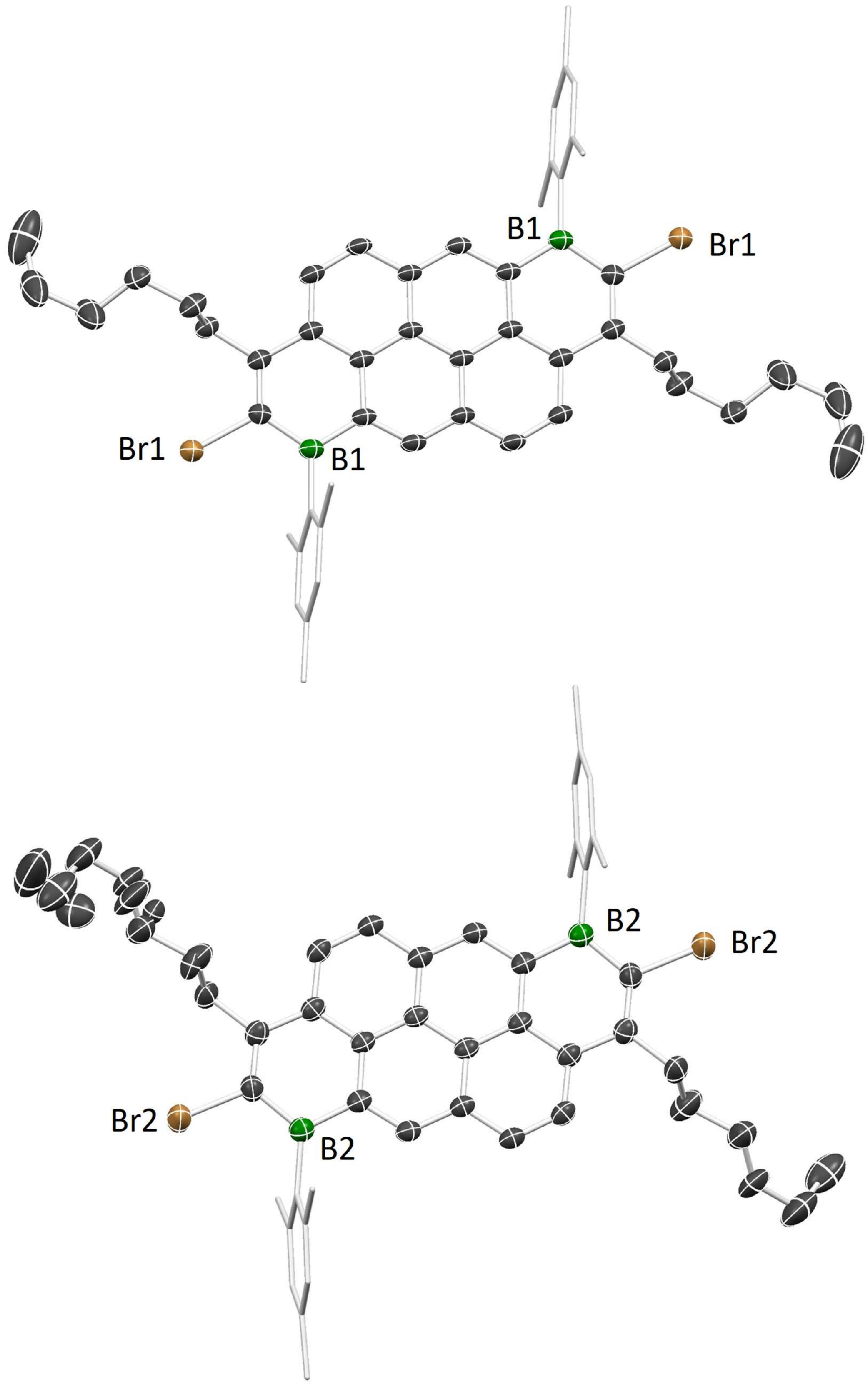

Figure S149. Crystal structure of **2a** showing the two independent molecules. Hydrogen atoms were omitted for clarity. Ellipsoids at the 50% probability level.

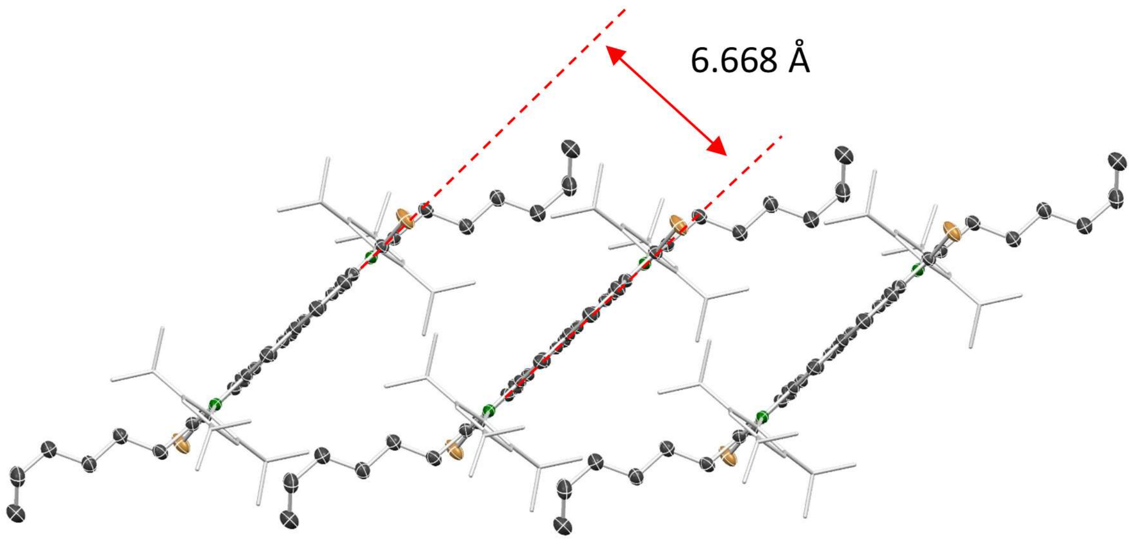

Figure S150. Extended packing structure for **2c**. Hydrogen atoms omitted for clarity. Ellipsoids at the 50% probability level.

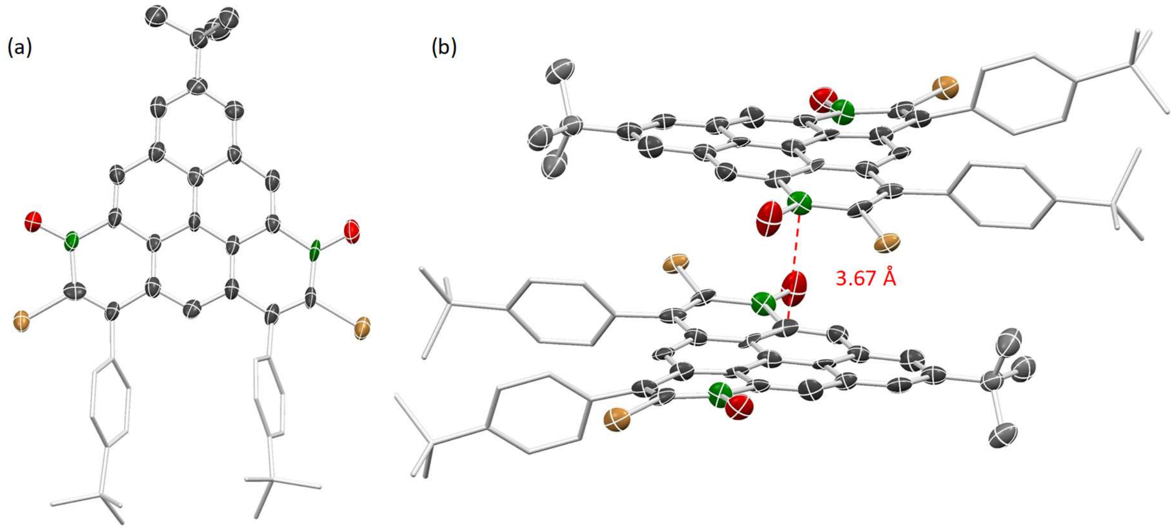

Figure S151. Solid-state structure (a) and packing structure (b) of **10b**. Hydrogen atoms were omitted for clarity. Ellipsoids at the 50% probability level. Due to the poor quality of the crystallographic data, no geometrical parameters other than atomic connectivity are appropriate to discuss for this compound.

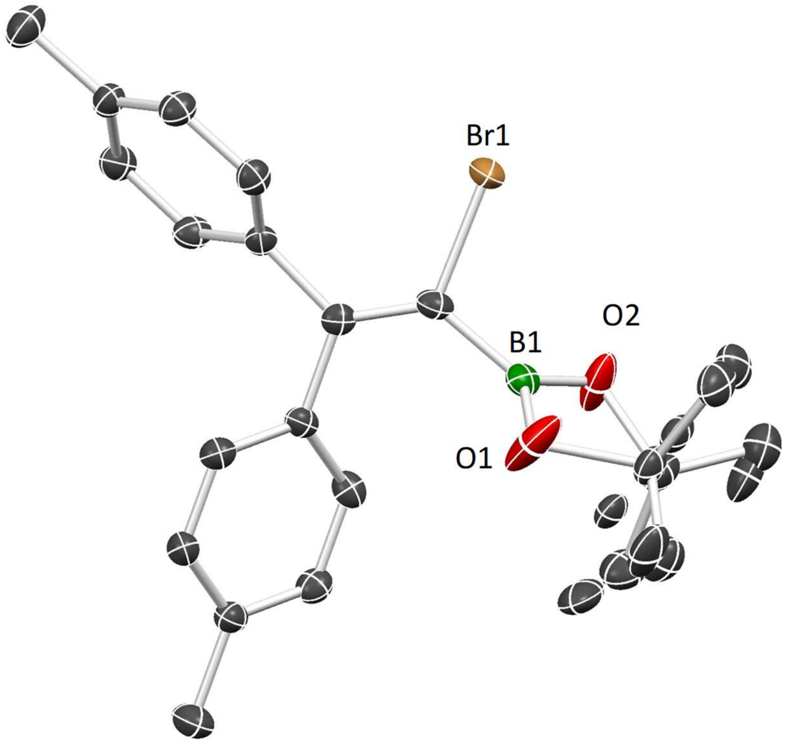

Figure S152. Solid-state structures of **8b**, hydrogen atoms were omitted for clarity. Ellipsoids at the 50% probability level.

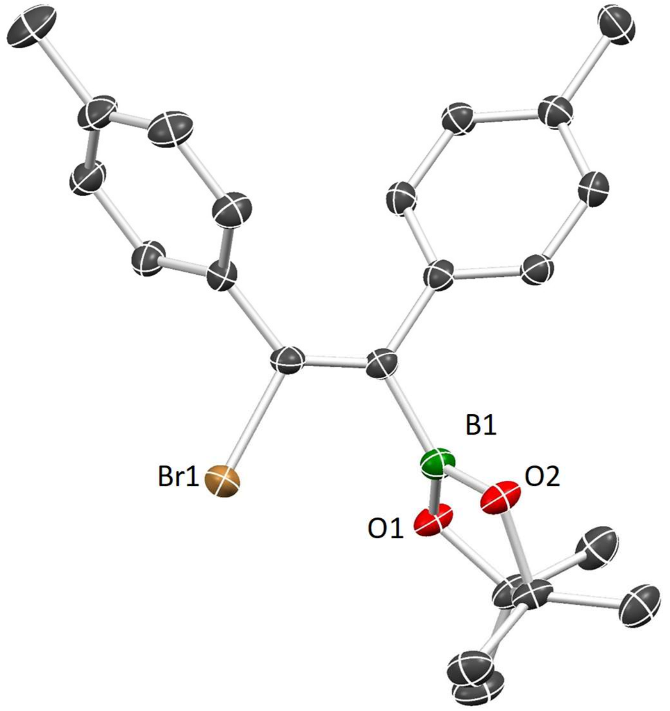

Figure S153. Solid-state structures of **8b-1,2**, hydrogen atoms were omitted for clarity. Ellipsoids at the 50% probability level.

S9. Computational Studies

DFT calculations were performed using the Gaussian09 suite of programs.[11] Geometries were optimised without constraints using the M06-2X functional[12] and 6-311G(d,p)[13] level of theory with a polarisable continuum model (PCM) for solvent (DCM) for all compounds except for compound **14**, which was optimised using the M06-2X functional and 6-31G(d) level of theory in the gas phase (compound **9a** was also calculated at both levels to allow for comparison with **14**). All stationary geometries were confirmed as minima through vibrational frequency analysis.

Figure S154. The relative thermal stability of the 1,2-cis-bromoboration product and the 1,1-bromoboration product (kcal/mol).

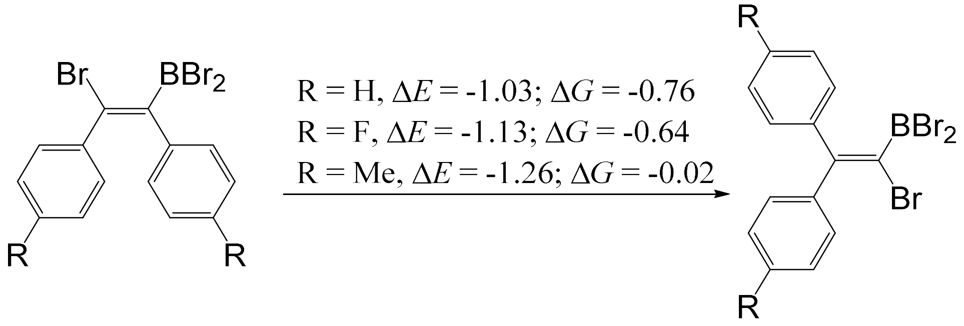

[11] Gaussian 09, Revision E.01, Frisch, M. J.; Trucks, G. W.; Schlegel, H. B.; Scuseria, G. E.; Robb, M. A.; Cheeseman, J. R.; Scalmani, G.; Barone, V.; Mennucci, B.; Petersson, G. A.; Nakatsuji, H.; Caricato, M.; Li, X.; Hratchian, H. P.; Izmaylov, A. F.; Bloino, J.; Zheng, G.; Sonnenberg, J. L.; Hada, M.; Ehara, M.; Toyota, K.; Fukuda, R.; Hasegawa, J.; Ishida, M.; Nakajima, T.; Honda, Y.; Kitao, O.; Nakai, H.; Vreven, T.; Montgomery, J. A., Jr.; Peralta, J. E.; Ogliaro, F.; Bearpark, M.; Heyd, J. J.; Brothers, E.; Kudin, K. N.; Staroverov, V. N.; Kobayashi, R.; Normand, J.; Raghavachari, K.; Rendell, A.; Burant, J. C.; Iyengar, S. S.; Tomasi, J.; Cossi, M.; Rega, N.; Millam, J. M.; Klene, M.; Knox, J. E.; Cross, J. B.; Bakken, V.; Adamo, C.; Jaramillo, J.; Gomperts, R.; Stratmann, R. E.; Yazyev, O.; Austin, A. J.; Cammi, R.; Pomelli, C.; Ochterski, J. W.; Martin, R. L.; Morokuma, K.; Zakrzewski, V. G.; Voth, G. A.; Salvador, P.; Dannenberg, J. J.; Dapprich, S.; Daniels, A. D.; Farkas, Ö.; Foresman, J. B.; Ortiz, J. V.; Cioslowski, J.; Fox, D. J. Gaussian, Inc., Wallingford CT, **2009**.
[12] Zhao, Y.; Truhlar, D. G. *Theor. Chem. Acc.* **2008**, *120*, 215.
[13] Mennucci, B.; Cancès, E.; Tomasi, J. *J. Phys. Chem. B* **1997**, *101*, 10506.

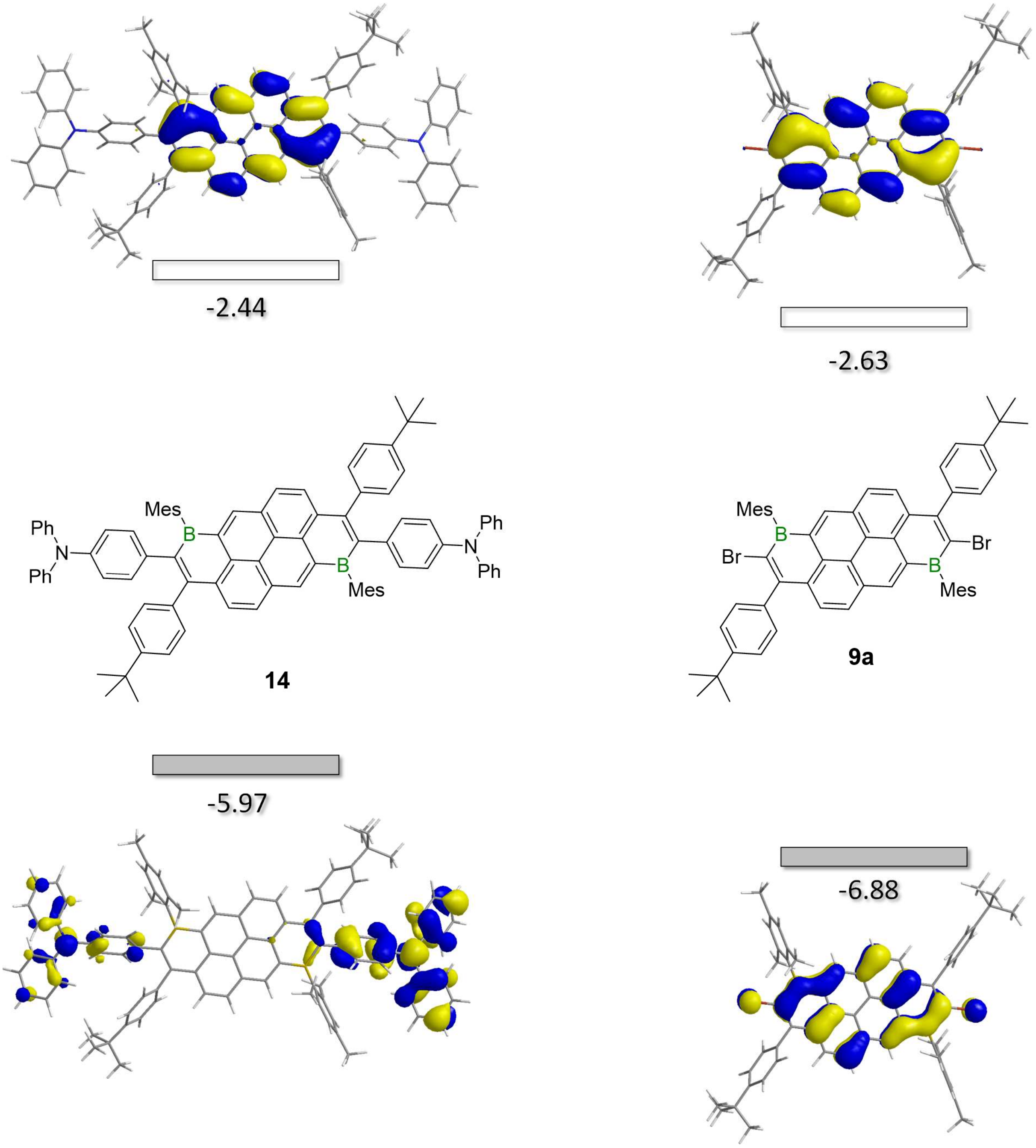

Figure S155. Calculated frontier orbitals (isovalue = 0.025) for **9a** and **14**. The structures were optimised at M06-2X/6-31G(d) level in the gas phase.

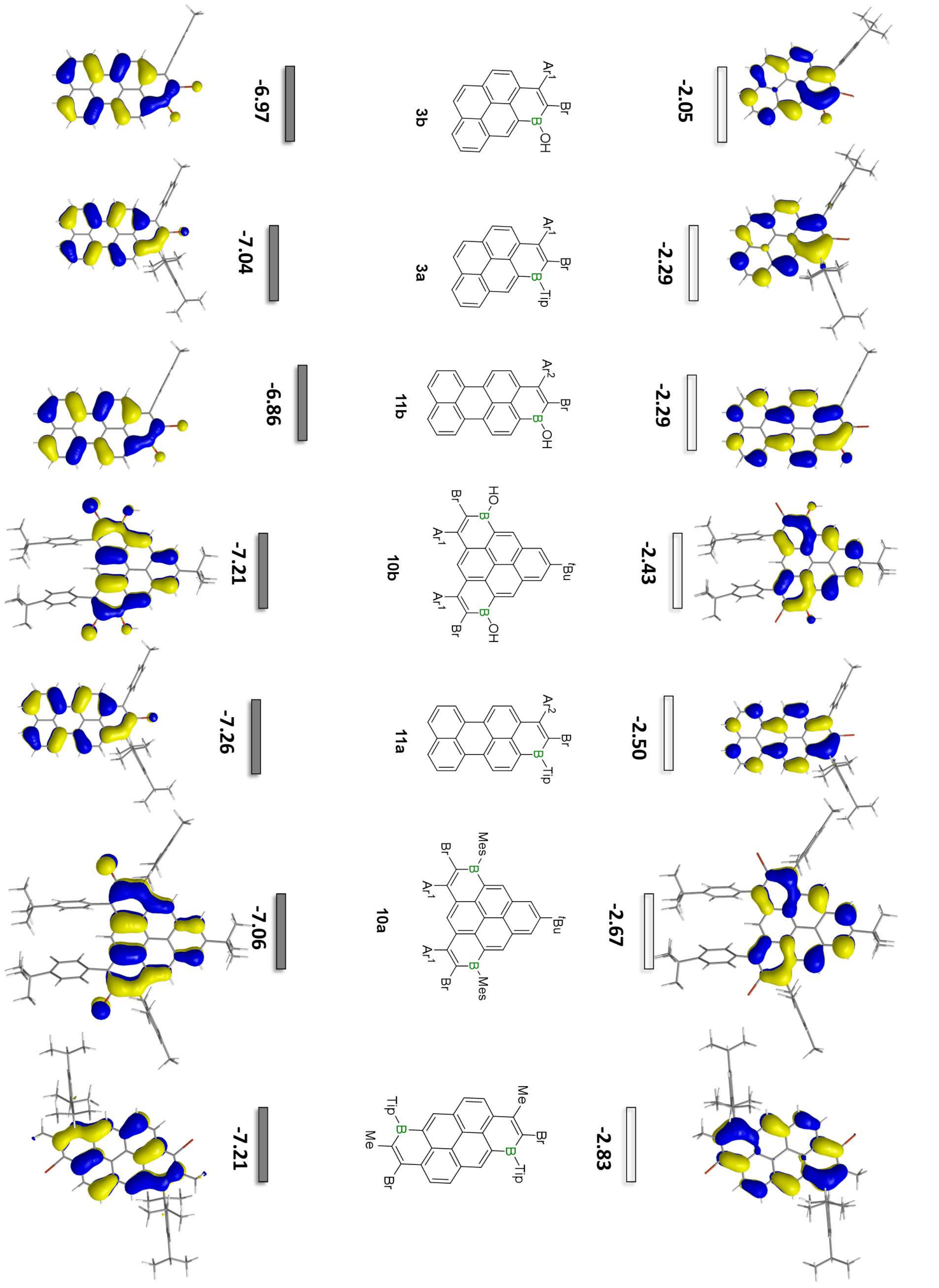

Figure S156. Calculated frontier orbitals (iso = 0.03) for several boron-doped PAHs at M06-2X/6-311G(d,p) level with a polarisable continuum model (PCM) of DCM. (Ar^1 = 4-tert-butylphenyl; Ar^2 = tolyl)

Optimised structure coordinates

2c

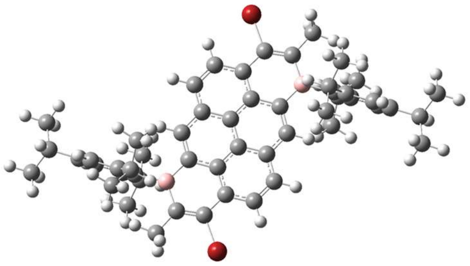

E (RM062X) = -7214.272255 Hartree

C	-0.38968400	-3.46062500	-0.40485900
C	-1.53902200	-2.66925400	-0.25045300
C	-1.38742800	-1.27044300	-0.09471500
C	-0.08695200	-0.70428700	-0.08344800
C	1.05325100	-1.52872200	-0.23922000
C	0.87608700	-2.90711700	-0.40229200
C	-2.53976000	-0.41640400	0.05097800
C	0.08696000	0.70428600	0.08342400
C	-1.05324200	1.52872000	0.23920600
C	-2.35139000	0.92954700	0.21828800
C	-0.87607800	2.90711500	0.40227500
H	-1.74475500	3.54439700	0.52325400
C	0.38969100	3.46062700	0.40480700
C	1.53902600	2.66925800	0.25037800
C	1.38743500	1.27044300	0.09467500
C	2.53976900	0.41640300	-0.05099500
C	2.35140000	-0.92955000	-0.21828900
H	3.21447000	-1.57977100	-0.33959600
H	-0.48670000	-4.52957700	-0.52720500
H	1.74476400	-3.54440200	-0.52324600
H	-3.21446000	1.57976500	0.33961700
H	0.48670900	4.52958000	0.52714300
C	-2.89482500	-3.24933600	-0.22901700
C	-4.05927200	-2.57480200	-0.09029700
C	2.89482400	3.24934600	0.22889000

C	4.05927600	2.57480900	0.09022600
B	3.96009200	1.02753900	-0.01475600
B	-3.96008300	-1.02753700	0.01474200
C	5.62115800	-0.61891100	1.08500300
C	5.95432300	-0.06557000	-1.25296600
C	6.70635300	-1.49117600	1.01665600
C	7.02960900	-0.95463500	-1.29196600
C	7.41792900	-1.67957700	-0.16792800
H	7.00550700	-2.03693000	1.90762700
H	7.57797300	-1.09652700	-2.21876400
C	-5.22864700	-0.09704600	0.06102500
C	-5.95424200	0.06556600	1.25307300
C	-5.62120800	0.61892000	-1.08491100
C	-7.02951300	0.95464900	1.29214300
C	-6.70639000	1.49119300	-1.01649700
C	-7.41789100	1.67960100	0.16813400
H	-7.57781500	1.09654600	2.21897700
H	-7.00559900	2.03694500	-1.90745000
C	5.39915700	3.26235600	0.04943800
H	5.58730500	3.81763300	0.97283100
H	6.19835300	2.53308300	-0.08258000
H	5.44579500	3.98328000	-0.77210800
C	4.90012800	-0.42637900	2.41150500
H	4.09280800	0.29931700	2.26051500
C	5.53173100	0.67220600	-2.51406800
H	4.84792700	1.47797700	-2.21909500
C	5.22865700	0.09704500	-0.06095800
C	5.84242100	0.16337000	3.46785900
H	6.65875700	-0.52878500	3.69096500
H	6.27971200	1.10128800	3.11728300
H	5.30173900	0.35929600	4.39692600
C	4.26002000	-1.72584500	2.90921900
H	3.54764300	-2.11975700	2.17996900
H	5.02068000	-2.49099100	3.08685100

H	3.72930500	-1.55414100	3.84880800
C	6.70810800	1.32563800	-3.24471000
H	7.27554400	1.97635600	-2.57539600
H	7.39125500	0.57485400	-3.64921600
H	6.34429300	1.92530100	-4.08225600
C	4.76121300	-0.26805200	-3.44917000
H	5.40406100	-1.09348100	-3.76835400
H	3.88849600	-0.69274900	-2.94678200
H	4.42159300	0.26594500	-4.34020000
C	8.58537000	-2.64565200	-0.22927500
H	8.96867500	-2.62642600	-1.25454300
C	9.71883900	-2.21381000	0.70778000
H	10.57054000	-2.89234300	0.61648400
H	10.05661800	-1.20180300	0.47493300
H	9.38693700	-2.22908900	1.74958600
C	8.13972700	-4.07871500	0.08257100
H	8.97832900	-4.77177700	-0.01970200
H	7.76506400	-4.15366600	1.10713600
H	7.34342500	-4.39826000	-0.59300200
C	-4.90025500	0.42638400	-2.41145300
H	-4.09294700	-0.29933800	-2.26051700
C	-4.26013300	1.72583800	-2.90918200
H	-5.02078000	2.49100900	-3.08676800
H	-3.54771300	2.11972000	-2.17995700
H	-3.72946600	1.55413200	-3.84879600
C	-5.84262300	-0.16332200	-3.46776400
H	-6.27991800	-1.10123600	-3.11718100
H	-6.65895400	0.52885900	-3.69081100
H	-5.30199900	-0.35924500	-4.39686600
C	-5.53158800	-0.67223500	2.51414100
H	-4.84775700	-1.47796700	2.21911700
C	-4.76108400	0.26801100	3.44926500
H	-3.88839600	0.69276400	2.94687500
H	-5.40395400	1.09340100	3.76850400

H	-4.42142200	-0.26601300	4.34026300
C	-6.70792200	-1.32575000	3.24478300
H	-7.39109100	-0.57501000	3.64933100
H	-7.27534400	-1.97646400	2.57545300
H	-6.34406600	-1.92543100	4.08229700
C	-8.58532900	2.64567400	0.22954900
H	-8.96851100	2.62652400	1.25486400
C	-8.13975400	4.07872200	-0.08246300
H	-8.97835400	4.77177500	0.01988000
H	-7.34336200	4.39832800	0.59297500
H	-7.76523800	4.15361200	-1.10708600
C	-9.71890300	2.21373400	-0.70733700
H	-9.38711500	2.22892300	-1.74918100
H	-10.05664000	1.20174400	-0.47436000
H	-10.57060300	2.89226500	-0.61600500
C	-5.39915700	-3.26234300	-0.04956900
H	-5.58735800	-3.81742100	-0.97307200
H	-5.44576700	-3.98343900	0.77182600
H	-6.19833800	-2.53308600	0.08263000
Br	-2.99589100	-5.16607000	-0.40457700
Br	2.99586400	5.16610100	0.40424400

3a

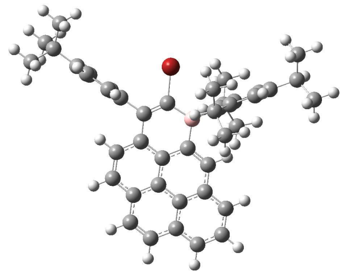

E (RM062X) = -4263.897194 Hartree

C	4.31735300	4.99545400	0.05173500
C	3.27145200	5.91212600	-0.00322300
C	1.93968600	5.48686700	-0.03999600
C	1.66074300	4.09600700	-0.01939200

C	2.73144100	3.16728600	0.03622200
C	4.05257200	3.63363300	0.07107000
C	0.84228100	6.41561900	-0.09864000
C	0.31307400	3.63102200	-0.05402800
C	-0.74647300	4.57346400	-0.11267500
C	-0.43819800	5.97840600	-0.13389700
C	-2.06355000	4.10052300	-0.14906500
H	-2.88091800	4.81089900	-0.19875500
C	-2.32883900	2.74385500	-0.12266200
C	-1.29767400	1.79029900	-0.05892800
C	0.04125300	2.23849100	-0.03016300
C	1.13580800	1.29867100	0.02237300
C	2.42294800	1.77203600	0.05352200
H	3.24994900	1.06694000	0.09257700
H	5.34071500	5.34848200	0.07934900
H	4.86432000	2.91589000	0.11403400
H	-1.26161100	6.68253100	-0.17872600
H	-3.35786100	2.40838600	-0.15191900
C	-1.62601800	0.35456000	-0.02830100
C	-0.64081600	-0.58178300	0.00708800
B	0.85375600	-0.22132000	0.03846700
C	2.59896900	-1.74782500	-1.09754100
C	2.53275400	-1.70032800	1.32561100
C	3.67925700	-2.62533800	-1.03760500
C	3.61516700	-2.58246300	1.35409300
C	4.20402300	-3.05211800	0.18346300
H	4.12478200	-2.98364700	-1.96175200
H	4.01421500	-2.91390000	2.30916700
C	2.06441500	-1.29213500	-2.44659500
H	1.14101200	-0.72607800	-2.27078800
C	1.92494000	-1.20576300	2.62907800
H	1.02721000	-0.62406000	2.38538900
C	2.02295900	-1.26974000	0.09245100
C	1.70005600	-2.47207500	-3.35336200

H	2.58748000	-3.05081200	-3.62298500
H	0.99431200	-3.14114800	-2.85635800
H	1.24420100	-2.11176600	-4.27881600
C	3.05785000	-0.35302000	-3.13980700
H	3.28080300	0.51534100	-2.51455600
H	3.99736300	-0.87545400	-3.34153000
H	2.65455700	0.00243800	-4.09139700
C	1.48064100	-2.36270700	3.52950700
H	0.79156900	-3.02411700	3.00010100
H	2.33842800	-2.95524600	3.85802800
H	0.97853600	-1.97902600	4.42092200
C	2.89133800	-0.27366100	3.36822000
H	3.80868900	-0.80701000	3.63313600
H	3.16409800	0.58282400	2.74633500
H	2.43797000	0.10050600	4.28957000
C	5.38130400	-4.00687900	0.23386500
H	5.60269200	-4.19293700	1.28982200
C	5.03968000	-5.35006400	-0.42070500
H	5.87489900	-6.04791100	-0.32206100
H	4.15764400	-5.79864500	0.04093900
H	4.83439500	-5.22040500	-1.48699000
C	6.62891600	-3.39191200	-0.41023500
H	7.47959500	-4.07247400	-0.32446300
H	6.45940600	-3.19776800	-1.47285700
H	6.89271400	-2.44671400	0.06896800
C	-3.06789500	-0.02868500	-0.02826700
C	-3.77903900	-0.07986700	1.16510400
C	-3.71843000	-0.36301800	-1.21591300
C	-5.11506600	-0.47416700	1.17625500
H	-3.28435700	0.18068200	2.09468000
C	-5.05022100	-0.75061100	-1.19702600
H	-3.17405800	-0.33013700	-2.15342400
C	-5.77780400	-0.82066500	-0.00198500
H	-5.63217600	-0.51178800	2.12626600

H	-5.52891400	-1.01085300	-2.13458200
Br	-1.10615500	-2.44399500	0.05213200
H	1.06533700	7.47667300	-0.11425600
H	3.48329100	6.97581700	-0.01837100
C	-7.23861000	-1.27587800	-0.03036100
C	-7.30981900	-2.70650900	-0.59029400
H	-8.34826200	-3.04898000	-0.60830900
H	-6.91895200	-2.75851800	-1.60874400
H	-6.73077600	-3.39296400	0.03262800
C	-7.87148500	-1.26966200	1.36422100
H	-7.86531100	-0.26874100	1.80365100
H	-8.91095500	-1.59764200	1.29059300
H	-7.35333100	-1.95042100	2.04440900
C	-8.05027400	-0.33296700	-0.93470300
H	-7.67698900	-0.33904300	-1.96082600
H	-9.09764400	-0.64670900	-0.95542300
H	-8.00546100	0.69308300	-0.56068100

3b

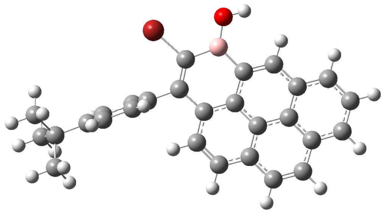

E (RM062X) = -3754.417040 Hartree

C	7.02618400	0.02136300	-0.05596100
C	6.66935500	-1.32247700	-0.12027200
C	5.32703500	-1.71159100	-0.11355200
C	4.31995600	-0.71394200	-0.04019800
C	4.69124400	0.65239100	0.02551600
C	6.04813100	1.00234000	0.01626500
C	4.93437100	-3.09476400	-0.17941100
C	2.94093000	-1.07927100	-0.03539800
C	2.58899100	-2.45393300	-0.10299600

C	3.62842900	-3.44660700	-0.17338300
C	1.23553900	-2.79950500	-0.10342900
H	0.95352500	-3.84515000	-0.15499200
C	0.25666100	-1.82284300	-0.04347000
C	0.57600600	-0.45866100	0.02633900
C	1.93802600	-0.07754300	0.03378000
C	2.32716000	1.31387500	0.10769100
C	3.65467600	1.63577500	0.10121200
H	3.97597400	2.67358400	0.15375500
H	8.07259600	0.30098300	-0.06271800
H	6.32451000	2.04984500	0.06638100
H	3.33601200	-4.48964400	-0.22374300
H	-0.78314900	-2.12381500	-0.05108800
C	-0.51445000	0.54022500	0.08674400
C	-0.23672700	1.85999800	0.16715000
B	1.21389300	2.39900600	0.19130300
C	-1.91501800	0.02772200	0.04792200
C	-2.57556600	-0.12224000	-1.16577600
C	-2.57942100	-0.32084400	1.22376000
C	-3.88059100	-0.60776500	-1.20659000
H	-2.06892600	0.14488500	-2.08697400
C	-3.87840800	-0.80601100	1.17513900
H	-2.07578500	-0.20949200	2.17806000
C	-4.55944700	-0.96008600	-0.03939200
H	-4.36176000	-0.70663300	-2.17105500
H	-4.36944900	-1.06889300	2.10554100
Br	-1.65776800	3.13836100	0.25758900
H	5.71188900	-3.84854700	-0.23618500
H	7.43767800	-2.08583600	-0.17799500
O	1.42797200	3.73465600	0.27861900
H	2.35333000	3.99468100	0.28931400
C	-5.98978100	-1.50396000	-0.04231800
C	-5.98958600	-2.92534900	0.54503600
H	-7.00653800	-3.32734700	0.55121000

H	-5.36050300	-3.58870500	-0.05419600
H	-5.61546100	-2.93467900	1.57090800
C	-6.58145500	-1.56418100	-1.45373700
H	-6.00235800	-2.22387900	-2.10495300
H	-7.59983800	-1.95609500	-1.40109600
H	-6.62558000	-0.57341400	-1.91320100
C	-6.88341200	-0.59568700	0.81904700
H	-6.54180100	-0.55987800	1.85564300
H	-6.88876600	0.42416800	0.42627800
H	-7.91006600	-0.97191600	0.81572200

9a

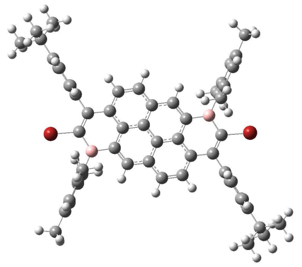

E (RM062X) = -7440.482286 Hartree

C	-2.86434100	-2.02660200	0.02059800
C	-3.02220600	-0.62942500	0.02349300
C	-1.87274100	0.19261700	0.02145300
C	-0.58793600	-0.40626100	0.02146400
C	-0.45711800	-1.81740500	0.01436000
C	-1.61228000	-2.61030400	0.01384100
C	-1.99239700	1.62959300	0.01335700
C	0.58670100	0.40596700	0.02704400
C	0.45588700	1.81712700	0.02817000
C	-0.85305500	2.39178400	0.01504800
C	1.61100900	2.61001800	0.03835800
H	1.51369000	3.68968300	0.03869000
C	2.86302300	2.02627900	0.04837900
C	3.02090500	0.62910500	0.04366600
C	1.87150200	-0.19290400	0.02995400

C	1.99121400	-1.62981700	0.01304900
C	0.85189500	-2.39199300	0.00426500
H	0.93564800	-3.47600700	-0.01051000
H	-3.74133200	-2.66045000	0.02185500
H	-1.51496500	-3.68995800	0.00841600
H	-0.93667500	3.47589100	0.00663600
H	3.73997600	2.66010600	0.05815600
C	-4.38248200	-0.05255600	0.03079800
C	-4.56290900	1.29258000	0.02628300
C	4.38114200	0.05220800	0.05414200
C	4.56165800	-1.29281700	0.03802500
B	3.38798100	-2.29334700	-0.00081800
B	-3.38911300	2.29325000	-0.00247000
C	3.60980700	-4.62501200	1.09512500
C	3.73187200	-4.47279500	-1.32050100
C	3.78589200	-6.00504900	1.00117300
C	3.90558800	-5.85359300	-1.38443900
C	3.93077800	-6.63792200	-0.23182100
H	3.81586400	-6.59886700	1.91064200
H	4.03044200	-6.32834000	-2.35397700
C	-3.57841300	3.84768400	-0.06986200
C	-3.60193400	4.62435600	1.09896700
C	-3.72794200	4.47559100	-1.31668700
C	-3.76751300	6.00546500	1.00680900
C	-3.89143500	5.85810300	-1.37882300
C	-3.91390400	6.64047500	-0.22533200
H	-3.78424000	6.59966700	1.91647600
H	-4.00616900	6.33564800	-2.34814900
C	3.46929800	-3.95944300	2.44306600
H	2.48906800	-3.48432000	2.54734700
C	3.71787600	-3.64187200	-2.58113900
H	4.47536100	-2.85262600	-2.53291900
C	3.57807100	-3.84748600	-0.07250300
C	4.08823000	-8.13445700	-0.32097900

H	4.71638700	-8.41409600	-1.16836700
C	-3.71159200	3.64771200	-2.57933500
H	-2.74147300	3.16016700	-2.71684600
C	-3.45529600	3.95723100	2.44550000
H	-2.47345100	3.48479100	2.54670000
C	-4.12483700	8.13089200	-0.30606900
H	-3.62337800	8.64470400	0.51571300
C	5.52667800	1.00657700	0.07773100
C	6.08166300	1.47186900	-1.11449400
C	6.04665600	1.45951900	1.28442400
C	7.13610400	2.37262500	-1.08882400
H	5.68507900	1.12425400	-2.06250900
C	7.10635200	2.36375400	1.30127000
H	5.62424000	1.10384900	2.21819000
C	7.67195400	2.84093800	0.11816400
H	7.54692700	2.71709700	-2.03150300
H	7.48450700	2.69293100	2.26047300
C	-5.52813000	-1.00704400	0.04034800
C	-6.05514200	-1.46934800	1.24632900
C	-6.07601700	-1.46309600	-1.15279900
C	-7.11012700	-2.36997700	1.24703700
H	-5.63698400	-1.11944300	2.18412100
C	-7.13519300	-2.36785400	-1.14314000
H	-5.67519600	-1.10953600	-2.09689400
C	-7.67333200	-2.84184800	0.05404800
H	-7.49966000	-2.71155700	2.19978400
H	-7.53493100	-2.69992000	-2.09255700
Br	6.34464500	-1.99747100	0.05860800
Br	-6.34585700	1.99725700	0.04816700
H	3.58557100	-4.67742500	3.25545900
H	4.22522900	-3.17771100	2.57132800
H	3.92030900	-4.25099300	-3.46264500
H	2.74753800	-3.15503000	-2.71950500
H	4.53345200	-8.53906700	0.58912600

H	3.11610900	-8.61689500	-0.45753900
H	-3.57169500	4.67365900	3.25924300
H	-4.20860000	3.17311100	2.57449000
H	-3.91150900	4.25919600	-3.45977800
H	-4.47003100	2.85917500	-2.53478200
H	-3.74604300	8.53167600	-1.24765200
H	-5.18971500	8.37344400	-0.24727700
C	-8.83173800	-3.84005100	0.10753700
C	-10.01152200	-3.20939400	0.86595100
H	-10.84537000	-3.91524800	0.91032900
H	-9.73777900	-2.94633400	1.88990100
H	-10.35355700	-2.30261100	0.36075900
C	-9.31391900	-4.24056500	-1.28971600
H	-8.52006500	-4.72258900	-1.86628500
H	-10.13825700	-4.95131000	-1.19643500
H	-9.67688400	-3.37662200	-1.85220500
C	-8.37161300	-5.10935700	0.84445700
H	-7.52988300	-5.57414700	0.32465900
H	-8.06100000	-4.88921900	1.86796100
H	-9.19070200	-5.83241800	0.88861600
C	8.83094100	3.83960100	0.09388200
C	9.28508100	4.23158700	1.50292500
H	9.63540800	3.36398000	2.06779900
H	10.11204700	4.94168600	1.43031600
H	8.48020300	4.71143100	2.06583400
C	8.38510800	5.11329200	-0.64425200
H	9.20512000	5.83626700	-0.66848500
H	8.09401100	4.89913900	-1.67477500
H	7.53376900	5.57528000	-0.13780700
C	10.02591400	3.21384400	-0.64459900
H	10.35662600	2.30299300	-0.13918100
H	9.77352000	2.95886100	-1.67607700
H	10.86109900	3.91916300	-0.66611200

9b

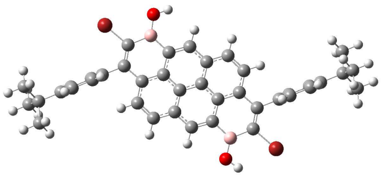

E (RM062X) = -3754.417040 Hartree

C	-2.88973400	-2.01008800	0.06473700
C	-3.04826400	-0.61542800	0.06711000
C	-1.89712500	0.20730900	0.07373000
C	-0.61200300	-0.39523700	0.07753500
C	-0.48300200	-1.80806500	0.07490000
C	-1.63809900	-2.59679000	0.06862400
C	-2.00809600	1.64859100	0.07662300
C	0.56613900	0.41248300	0.08390700
C	0.43980200	1.82393000	0.08643500
C	-0.86904000	2.40077500	0.08267300
C	1.59545500	2.61315500	0.09253500
H	1.50212600	3.69327700	0.09432900
C	2.84701800	2.02631800	0.09640900
C	3.00328400	0.63123000	0.09420900
C	1.85100500	-0.18962800	0.08775900
C	1.95929900	-1.62798600	0.08500500
C	0.82521600	-2.38695600	0.07877800
H	0.90588400	-3.47027800	0.07668800
H	-3.76700500	-2.64347100	0.05963000
H	-1.54363600	-3.67678300	0.06671000
H	-0.91835100	3.48694100	0.08476500
H	3.72486200	2.65917300	0.10136100
C	-4.41762400	-0.04714500	0.06225300
C	-4.60753600	1.28943000	0.06488700
C	4.37042300	0.05739200	0.09909800
C	4.55089500	-1.28033200	0.09670600
B	3.36607800	-2.27981100	0.08920100

B	-3.42647300	2.29265200	0.07263200
C	5.51785900	1.00987000	0.10532400
C	6.05988800	1.47134200	-1.09436700
C	6.05628000	1.46097400	1.30464200
C	7.11933700	2.36663400	-1.08319700
H	5.64899200	1.12515600	-2.03676000
C	7.12031300	2.36024600	1.30722100
H	5.64393300	1.10801600	2.24394100
C	7.67293100	2.83341600	0.11632300
H	7.52029200	2.70773500	-2.03135500
H	7.51251300	2.68835600	2.26114000
C	-5.55832200	-1.00837100	0.05586700
C	-6.09669900	-1.47507200	1.25512700
C	-6.09292800	-1.46462100	-1.14315700
C	-7.14883500	-2.37898900	1.24411300
H	-5.68909400	-1.12526100	2.19763800
C	-7.14964800	-2.37249100	-1.14611500
H	-5.68374100	-1.10797600	-2.08247200
C	-7.69877200	-2.85011600	0.04462400
H	-7.54712600	-2.72321700	2.19230700
H	-7.53913700	-2.70362400	-2.10013700
Br	6.31830800	-2.02032300	0.10263500
Br	-6.37826900	2.00661800	0.05944500
C	-8.85546100	-3.85099600	0.08464300
C	-10.04320700	-3.22552900	0.83491300
H	-10.87650600	-3.93265400	0.86861000
H	-9.77925300	-2.96648800	1.86247500
H	-10.38188800	-2.31702200	0.33051600
C	-9.32482700	-4.24763000	-1.31807600
H	-8.52514000	-4.72639800	-1.88925500
H	-10.14886300	-4.95994200	-1.23431400
H	-9.68412000	-3.38230800	-1.88081700
C	-8.39916800	-5.12191200	0.82113300
H	-7.55243500	-5.58349600	0.30664600

H	-8.09712500	-4.90439800	1.84777700
H	-9.21732800	-5.84656400	0.85638500
C	8.83751700	3.82505100	0.07611000
C	9.30811500	4.22023300	1.47884300
H	9.66010600	3.35314100	2.04346600
H	10.13767100	4.92606300	1.39508700
H	8.51140300	4.70606600	2.04820700
C	8.39219500	5.09829800	-0.66306200
H	9.21625400	5.81617100	-0.69821300
H	8.08992200	4.88173300	-1.68982800
H	7.54856900	5.56749700	-0.15038000
C	10.02114300	3.18874900	-0.67161400
H	10.35194000	2.27850300	-0.16512400
H	9.75659900	2.93016800	-1.69913900
H	10.86004600	3.88916300	-0.70522200
O	3.50095300	-3.62624400	0.08620300
H	4.42191500	-3.91360200	0.08946900
O	-3.68397200	3.62153100	0.07554800
H	-2.90477500	4.18482800	0.08131100

10a

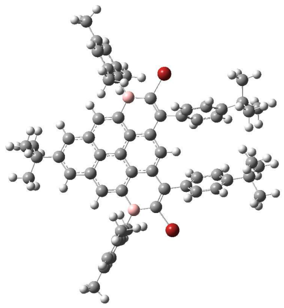

E (RM062X) = -7597.704566 Hartree

C	-4.19712100	4.53773600	-0.03388100
C	-4.60934500	3.20740500	-0.00013300
C	-3.69490400	2.14178900	0.00794600
C	-2.30625300	2.40724800	-0.01244900
C	-1.87404800	3.75558400	-0.04265600
C	-2.82062100	4.78530800	-0.05601000

C	-4.12793400	0.78339200	0.03939500
C	-1.36199100	1.34166300	-0.00053900
C	-1.82925600	0.00346200	0.00804400
C	-3.24396700	-0.26784300	0.03329100
C	-0.88744100	-1.04978700	0.00435400
C	0.47509500	-0.73582200	0.06693400
C	0.95588200	0.57707000	0.05578700
C	0.02476300	1.64055800	0.00113800
C	0.46315600	3.01310500	-0.03484600
C	-0.47090900	4.01891200	-0.05835800
H	-0.13866300	5.05394900	-0.08668200
H	-5.19607300	0.58169500	0.06755200
H	-5.66429900	2.96076400	0.01981800
H	-2.45515500	5.80718900	-0.08390600
H	1.19101100	-1.54443400	0.12606400
C	2.40959900	0.82596300	0.08828300
C	-1.31563800	-2.45979200	-0.05343200
C	2.88416200	2.10026400	-0.00005300
C	-2.63700100	-2.78231100	-0.00783900
B	-3.75133200	-1.72722500	0.06134500
B	1.97265800	3.33664700	-0.04992000
C	-0.24797400	-3.48588300	-0.19416400
C	0.04844600	-4.36020700	0.85120300
C	0.48038100	-3.57724900	-1.37581100
C	1.06101400	-5.29564600	0.71119400
H	-0.51494100	-4.30128600	1.77580400
C	1.49218200	-4.52391900	-1.51174500
H	0.26013500	-2.90107800	-2.19528900
C	1.80743000	-5.39608800	-0.47014100
H	1.27671800	-5.95586100	1.54374300
H	2.03731200	-4.56316900	-2.44587700
C	3.35414500	-0.32179600	0.23550900
C	3.63964500	-1.19109600	-0.81269300
C	4.07171600	-0.45675200	1.42629000

C	4.67798700	-2.11777000	-0.70165500
H	3.08210200	-1.11955400	-1.74111400
C	5.10002700	-1.37894000	1.53007600
H	3.85171300	0.20442200	2.25748200
C	5.45862400	-2.20152600	0.45328900
H	4.89088500	-2.75164600	-1.55381200
H	5.66262800	-1.42206200	2.45552200
C	-5.28119700	-2.06499400	0.14611700
C	-5.87767400	-2.26939200	1.40061600
C	-6.07404300	-2.13305500	-1.01032600
C	-7.24480800	-2.52646600	1.48354900
C	-7.43900100	-2.39358000	-0.89781000
C	-8.04305300	-2.59181400	0.34265700
H	-7.69814100	-2.67785400	2.45953700
H	-8.04564000	-2.44072000	-1.79821500
C	2.51866200	4.80651600	-0.09483400
C	2.81301400	5.41628800	-1.32452100
C	2.74236200	5.51572000	1.09512200
C	3.31957300	6.71390400	-1.34892100
C	3.25210300	6.81225100	1.04159000
C	3.54924500	7.42726300	-0.17302000
H	3.54459000	7.17821900	-2.30525600
H	3.42608800	7.35256600	1.96811400
C	2.94764300	-6.40917100	-0.55234000
C	6.73315100	-3.04661200	0.54247600
C	7.92904000	-2.07537600	0.57797700
H	7.88221700	-1.42116800	1.45156400
H	8.86603500	-2.63787600	0.62126400
H	7.94157100	-1.44824600	-0.31710900
C	6.90961900	-3.97016800	-0.66735300
H	6.99351400	-3.40207100	-1.59705300
H	7.83054600	-4.54582500	-0.54802400
H	6.08346500	-4.67755500	-0.76718300
C	6.75101100	-3.89580600	1.82424700

H	7.67135500	-4.48496600	1.85797400
H	6.72429700	-3.27583000	2.72226100
H	5.90478900	-4.58400500	1.86016000
C	3.95602300	-6.07366400	0.55839300
H	3.52668800	-6.22374300	1.55160800
H	4.84660000	-6.70391200	0.47386400
H	4.25795900	-5.02598200	0.47844700
C	3.67364600	-6.35160300	-1.89956400
H	4.48623500	-7.08193400	-1.90531700
H	3.00268500	-6.58889200	-2.72907000
H	4.10880200	-5.36433100	-2.07873400
C	2.40105400	-7.83095500	-0.35035100
H	3.22079400	-8.55386400	-0.38488100
H	1.89927000	-7.93529900	0.61389100
H	1.68535500	-8.08393500	-1.13676000
C	2.43870700	4.86614500	2.42400000
H	1.36823800	4.66399900	2.52737300
H	2.95964100	3.90740400	2.51603800
H	2.74584000	5.49928400	3.25700600
C	2.59237100	4.65259700	-2.60788400
H	3.18610100	3.73230800	-2.61436700
H	1.54277800	4.36379300	-2.71866700
H	2.87564500	5.24418600	-3.47910900
C	4.13808900	8.81427300	-0.21500400
H	3.84990500	9.39228500	0.66438600
H	5.23059900	8.76777400	-0.23764800
H	3.81341600	9.35414800	-1.10613400
C	-5.03251300	-2.20586700	2.65029300
H	-4.20583300	-2.92144400	2.59405400
H	-4.59335400	-1.21150700	2.77815700
H	-5.61895200	-2.43474200	3.54072800
C	-5.44281000	-1.92971800	-2.36689600
H	-5.01176700	-0.92798100	-2.45633000
H	-4.63221400	-2.64764100	-2.52798300

H	-6.17183800	-2.05815300	-3.16762400
C	-9.51549600	-2.89858000	0.44527000
H	-10.07239400	-2.42957800	-0.36762700
H	-9.69068000	-3.97655500	0.38770800
H	-9.92541300	-2.54710500	1.39354400
C	-5.17589100	5.71286100	-0.05296700
C	-6.63328100	5.24763000	0.01201500
H	-6.89685500	4.62403600	-0.84609100
H	-6.83688500	4.68695600	0.92800800
H	-7.28879300	6.12106400	0.00229900
C	-4.90022400	6.62272200	1.15599800
H	-5.02818900	6.07300600	2.09174600
H	-3.88702000	7.02972400	1.13455800
H	-5.59835800	7.46374000	1.15251300
C	-4.97452900	6.51026400	-1.35252000
H	-5.16879300	5.88153300	-2.22506800
H	-5.66438400	7.35782400	-1.37833100
H	-3.95809800	6.90179900	-1.43267600
Br	-3.17288700	-4.62366200	-0.12306900
Br	4.78045000	2.41478100	-0.06813100

10b

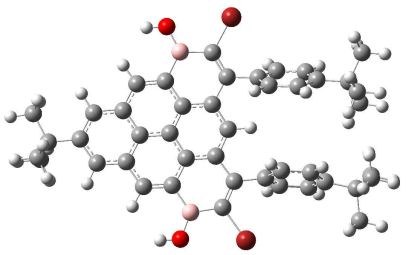

E (RM062X) = -7050.393508 Hartree

C	-6.94719600	-0.42413700	0.00139100
C	-6.33014900	0.82214700	-0.07944300
C	-4.93440000	0.96229100	-0.08681000
C	-4.11029600	-0.18336900	-0.01424800
C	-4.72746400	-1.45481800	0.07171000

C	-6.12120200	-1.55015000	0.07916800
C	-4.30859700	2.24406800	-0.16786300
C	-2.69020000	-0.06274400	-0.02658700
C	-2.10566700	1.22934400	-0.09281900
C	-2.95067900	2.39892100	-0.16870100
C	-0.69740500	1.34028900	-0.08642600
C	0.07111000	0.17211700	-0.08527400
C	-0.48434300	-1.10833000	-0.02337400
C	-1.89118300	-1.23579400	0.03141100
C	-2.52635400	-2.53010900	0.13653400
C	-3.88942500	-2.61047900	0.15257400
H	-4.39085500	-3.57237600	0.22758800
H	-4.96888900	3.10611600	-0.22687100
H	-6.92523400	1.72581200	-0.13798500
H	-6.56119400	-2.54018200	0.14868600
H	1.14825200	0.26108800	-0.12984900
C	0.41127400	-2.28872200	-0.00939300
C	-0.01945800	2.65761900	-0.07954500
C	-0.10691400	-3.53228800	0.12521600
C	-0.73636600	3.79986700	-0.16661100
B	-2.28047200	3.80198200	-0.24541300
B	-1.62726300	-3.79525400	0.22871100
C	1.46260700	2.66077400	0.06174100
C	2.28465400	2.95592900	-1.02512800
C	2.04782200	2.35970600	1.28670100
C	3.66340200	2.93967200	-0.88153200
H	1.83880300	3.19558900	-1.98428000
C	3.43326000	2.35032500	1.42507400
H	1.41835500	2.12586700	2.13905600
C	4.26822100	2.63178300	0.34365200
H	4.27792700	3.16642800	-1.74574700
H	3.85196700	2.11050000	2.39376900
C	1.88733500	-2.10750100	-0.15556500
C	2.68768800	-1.69354800	0.90453900

C	2.50752100	-2.50448300	-1.34170600
C	4.07840900	-1.75771000	0.80943200
H	2.22818200	-1.36235000	1.83033600
C	3.88921000	-2.56804500	-1.42906600
H	1.89765400	-2.81820400	-2.18200400
C	4.70794000	-2.23985400	-0.34037300
H	4.66535200	-1.46102700	1.67010300
H	4.33106500	-2.92986600	-2.35033500
C	5.79246100	2.57589800	0.43604700
C	6.21075700	-2.53124700	-0.40526400
C	6.38405500	-4.06231300	-0.40668400
H	5.89576700	-4.51395600	-1.27328100
H	7.44686300	-4.31910500	-0.43915500
H	5.94984700	-4.49950900	0.49602400
C	6.96767000	-1.97014100	0.80398500
H	6.63001100	-2.42923300	1.73632600
H	8.03235900	-2.18964500	0.69505100
H	6.85580300	-0.88698200	0.89183000
C	6.83679900	-1.96585400	-1.69087800
H	7.90818500	-2.18324800	-1.69999100
H	6.40364200	-2.41629600	-2.58575700
H	6.70779000	-0.88428300	-1.75767800
C	6.28960300	1.52814400	-0.57255200
H	6.09075800	1.83052400	-1.60333800
H	7.36760400	1.37329500	-0.46563500
H	5.77965800	0.57729800	-0.39757600
C	6.27175400	2.16530100	1.83132100
H	7.36352800	2.12715700	1.84221600
H	5.95317900	2.88040500	2.59411200
H	5.89760900	1.17522700	2.10699800
C	6.38752600	3.95134700	0.09702800
H	7.47951900	3.90615200	0.13646400
H	6.09894500	4.27679500	-0.90454600
H	6.04772600	4.70530200	0.81173000

C	-8.46659100	-0.60061400	0.01002400
C	-9.20125200	0.73798200	-0.10412600
H	-8.96826800	1.39766800	0.73565200
H	-8.95391900	1.25569900	-1.03448300
H	-10.27840900	0.55831500	-0.09795100
C	-8.87877000	-1.48600200	-1.17821600
H	-8.57791100	-1.02811600	-2.12381500
H	-8.42566800	-2.47795100	-1.11915100
H	-9.96441300	-1.61261800	-1.18620100
C	-8.88768800	-1.27713500	1.32511700
H	-8.60500100	-0.66259400	2.18345700
H	-9.97218200	-1.41349500	1.34120500
H	-8.42394100	-2.25921100	1.44064000
Br	0.13663000	5.50159100	-0.10198900
Br	1.03177200	-5.06867200	0.21191400
O	-2.94140200	4.98052600	-0.34810000
H	-3.89883400	4.90558700	-0.38391200
O	-2.07752000	-5.06391900	0.38599900
H	-3.03253000	-5.14804600	0.45434400

<u>11a</u>

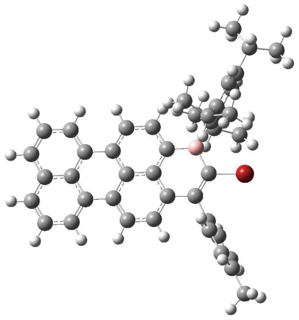

E (RM062X) = -4299.594512 Hartree

C	-0.02829000	2.16321800	-0.01821400
C	1.29154200	1.51733400	-0.03296500
C	1.43803700	0.09725000	0.02959900
C	2.74198000	-0.48236000	0.02908500
C	3.89847700	0.35615000	-0.03653000

C	3.71244900	1.72685000	-0.10228300
H	4.55799400	2.39750100	-0.15506100
C	2.43827800	2.29429200	-0.10008800
H	2.35372400	3.37257200	-0.15039500
C	5.24339200	-0.24540400	-0.03309300
C	5.37162300	-1.66393000	0.03320900
C	4.22384600	-2.50866100	0.09570800
C	2.87859700	-1.90406800	0.09513300
C	1.73318500	-2.68663500	0.15831400
H	1.80164900	-3.76372700	0.21136300
C	0.46573700	-2.10806600	0.15363700
H	-0.40726300	-2.75218500	0.20274600
C	0.28345600	-0.73253800	0.08827300
C	-1.15886800	1.41365500	0.04967900
C	-2.41734400	-1.02939500	0.09180600
C	-2.89193500	-1.59906700	-1.10293100
C	-3.99537700	-2.45040500	-1.07214000
H	-4.35704500	-2.88468300	-2.00056400
C	-4.64816300	-2.75517500	0.12217200
C	-4.17742500	-2.17343800	1.29654600
H	-4.68183600	-2.40399400	2.23074400
C	-3.07943000	-1.31145400	1.29700800
C	-2.57877300	-0.72196300	2.60651800
H	-1.85982500	0.07087200	2.36620100
C	-3.69928700	-0.07755900	3.42774600
H	-4.24025300	0.66278800	2.83481900
H	-3.28310000	0.41992700	4.30707600
H	-4.41442900	-0.82583600	3.77876100
C	-1.84019700	-1.78601100	3.42723000
H	-2.51969900	-2.60390200	3.68354500
H	-1.45238100	-1.35971300	4.35597300
H	-1.00266200	-2.20472600	2.86342000
C	-5.84164700	-3.69077500	0.14106700
H	-6.17312600	-3.77298300	1.18130900

C	-5.46083900	-5.09306600	-0.34661900
H	-5.13317800	-5.06453300	-1.38958200
H	-6.31824500	-5.76771700	-0.28252300
H	-4.64811800	-5.51002400	0.25197700
C	-7.00631000	-3.12907600	-0.68211100
H	-7.29553200	-2.13748000	-0.32802500
H	-7.87554100	-3.78808000	-0.61470000
H	-6.72989700	-3.04481200	-1.73682700
C	-2.22706100	-1.28333700	-2.43468300
H	-1.42020100	-0.56404000	-2.25173100
C	-3.20971500	-0.61981500	-3.40656700
H	-4.02535600	-1.30137900	-3.66276400
H	-2.70051100	-0.34134600	-4.33248600
H	-3.64542800	0.27981500	-2.96624500
C	-1.59522700	-2.53176500	-3.05908300
H	-0.86091600	-2.97768800	-2.38385900
H	-1.09278400	-2.28029600	-3.99640600
H	-2.35854100	-3.28433100	-3.27576400
C	4.40998700	-3.87874800	0.15586600
H	3.56217600	-4.54754200	0.20236300
C	5.69277900	-4.45351200	0.15821600
H	5.79116600	-5.53088100	0.20614400
C	6.80429400	-3.65590200	0.09974800
H	7.79981100	-4.08532500	0.10063200
C	6.66911400	-2.24814800	0.03642100
C	7.81292200	-1.41609300	-0.02353000
H	8.79485500	-1.87557900	-0.01884900
C	7.67291000	-0.05524200	-0.08598000
H	8.54505600	0.58520400	-0.13053200
C	6.39243100	0.52430400	-0.09113500
H	6.32991300	1.60252800	-0.14105600
B	-1.13276600	-0.12269500	0.08175600
Br	-2.86763000	2.28971800	0.10093600
C	-0.07003500	3.65313200	-0.08328600

C	-0.03552800	4.41642900	1.08176800
C	-0.15599300	4.29815700	-1.31576400
C	-0.09139700	5.80406000	1.01141100
H	0.02690100	3.92160300	2.04508100
C	-0.21094000	5.68527800	-1.37800600
H	-0.18869300	3.70925600	-2.22617200
C	-0.17896500	6.46014700	-0.21703200
H	-0.07221300	6.38669600	1.92654400
H	-0.28634300	6.17460800	-2.34360200
C	-0.21530300	7.96469200	-0.29174600
H	0.79254600	8.36887700	-0.42067300
H	-0.81685500	8.30048100	-1.13776000
H	-0.63084900	8.39339200	0.62107900

<u>11b</u>

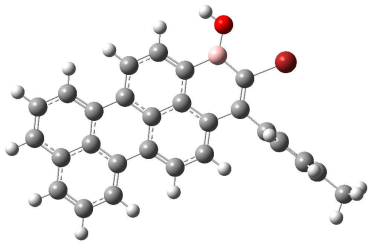

E (RM062X) = -3790.113885 Hartree

C	2.07252900	-0.19447700	0.00074600
C	0.65038000	0.19802400	0.00126600
C	-0.40796200	-0.76808500	0.00590700
C	-1.76604700	-0.32784400	0.00462000
C	-2.06773800	1.07225600	0.00738900
C	-1.01588100	1.96794700	-0.00051200
H	-1.19668700	3.03324900	-0.00533100
C	0.31468800	1.53938600	-0.00393400
H	1.09726000	2.28754800	-0.01021800
C	-3.47165400	1.52289300	0.01828100
C	-4.51548200	0.55076400	-0.00039000
C	-4.22509300	-0.84630700	-0.01572700

C	-2.81873400	-1.29418300	0.00039100
C	-2.49070900	-2.64100700	0.01112400
H	-3.26102700	-3.39861000	0.01614800
C	-1.16017400	-3.05807400	0.01666100
H	-0.97234300	-4.12783600	0.02430000
C	-0.10786000	-2.16009700	0.01142500
C	2.42386400	-1.49912400	0.00686200
C	-5.27799000	-1.74288600	-0.04505400
H	-5.09383500	-2.80767500	-0.06375800
C	-6.61718600	-1.31428300	-0.05320400
H	-7.40987100	-2.05176400	-0.07720900
C	-6.91274800	0.02217300	-0.03045800
H	-7.94077200	0.36691300	-0.03396400
C	-5.87184800	0.98166500	-0.00357400
C	-6.16734600	2.36617400	0.01940700
H	-7.20527000	2.67975300	0.01705300
C	-5.15390000	3.28655700	0.04540600
H	-5.37473500	4.34664100	0.06571500
C	-3.81303100	2.86369900	0.04472300
H	-3.04660000	3.62605800	0.06680000
B	1.37112600	-2.62958200	0.01267000
Br	4.26943200	-2.00195600	0.00686400
C	3.09546200	0.89145800	-0.00663100
C	3.58522500	1.39081800	-1.21112900
C	3.59458600	1.39860900	1.19237000
C	4.56566400	2.37699100	-1.21386200
H	3.20610600	0.99605900	-2.14778000
C	4.57403100	2.38371500	1.18184900
H	3.22206000	1.00917900	2.13389600
C	5.07589700	2.88797600	-0.02007000
H	4.94553500	2.75029100	-2.15921200
H	4.96148500	2.76329000	2.12186500
O	1.78020600	-3.92414500	0.01746500
H	1.06639500	-4.56745700	0.01960400

C	6.12511100	3.96919300	-0.02218800
H	6.66318900	3.99338300	-0.97047400
H	5.66765200	4.95094500	0.12797400
H	6.84771900	3.81597700	0.78107700

12a

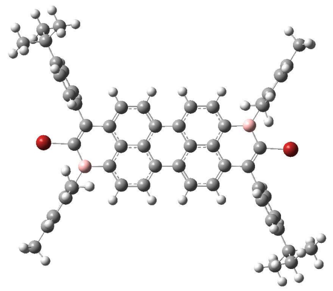

E (RM062X) = -7594.094332 Hartree

C	5.20667100	-0.36581100	-0.00719400
C	3.74854400	-0.57624000	-0.01336600
C	2.82549200	0.51475600	0.00109100
C	1.42421400	0.25626200	0.00104100
C	0.94209200	-1.08662200	-0.02118000
C	1.86763600	-2.11459900	-0.04840100
H	1.55114500	-3.14718700	-0.07413100
C	3.24216900	-1.86469600	-0.04473800
H	3.91895600	-2.70888700	-0.06818800
C	-0.50823500	-1.35073100	-0.01810900
C	-1.42424200	-0.25629000	-0.00117900
C	-0.94212000	1.08659000	0.02120900
C	0.50820500	1.35070300	0.01782200
C	1.00778500	2.64485500	0.02798200
H	0.34209700	3.49576100	0.03661300
C	2.38235900	2.88600800	0.02357800
H	2.73851100	3.91169200	0.02949100
C	3.30724600	1.85226600	0.01156900
C	5.71672100	0.89200800	-0.01417000
C	-1.86766100	2.11456100	0.04885200
H	-1.55116300	3.14713800	0.07491800

C	-3.24219400	1.86466000	0.04522400
H	-3.91898300	2.70884300	0.06894700
C	-3.74857000	0.57621300	0.01351100
C	-2.82552000	-0.51478000	-0.00124200
C	-3.30727800	-1.85228600	-0.01202300
C	-2.38239400	-2.88602800	-0.02435700
H	-2.73855000	-3.91170900	-0.03056200
C	-1.00781800	-2.64487800	-0.02870400
H	-0.34213400	-3.49578400	-0.03766300
B	4.82409900	2.14605200	-0.00005400
Br	7.61907800	1.13659400	0.00433600
C	6.07525800	-1.57746100	0.01085400
C	6.33641500	-2.23988500	1.20484000
C	6.63539400	-2.06792400	-1.16883900
C	7.14909700	-3.37161700	1.22280400
H	5.90600900	-1.86939800	2.12923500
C	7.43921500	-3.19791900	-1.14332100
H	6.44114400	-1.55896300	-2.10673500
C	7.71633800	-3.87516400	0.05162000
H	7.33196200	-3.85628700	2.17304800
H	7.85991900	-3.55638600	-2.07641100
C	-5.20669700	0.36578600	0.00729400
C	-5.71674800	-0.89203100	0.01400800
B	-4.82412600	-2.14607600	-0.00039100
Br	-7.61910700	-1.13658400	-0.00450300
C	-6.07526600	1.57745000	-0.01053200
C	-6.63529200	2.06780900	1.16925500
C	-6.33648400	2.24001300	-1.20442900
C	-7.43908200	3.19783200	1.14391600
H	-6.44098800	1.55874700	2.10708400
C	-7.14912900	3.37177400	-1.22221200
H	-5.90614100	1.86961800	-2.12888900
C	-7.71629100	3.87519900	-0.05093300
H	-7.85968400	3.55623200	2.07707800

H	-7.33202900	3.85655900	-2.17238900
C	-5.39671500	-3.60620800	-0.00834700
C	-5.60731100	-4.28288600	-1.21912200
C	-5.72964800	-4.23869900	1.20013200
C	-6.14025300	-5.57126700	-1.20720000
C	-6.25675900	-5.52783300	1.18314600
C	-6.46776900	-6.21157200	-0.01386500
H	-6.30979400	-6.08369800	-2.15017700
H	-6.51677800	-6.00808400	2.12257600
C	5.39666700	3.60619200	0.00784000
C	5.72959700	4.23866400	-1.20065800
C	5.60727100	4.28289100	1.21859700
C	6.25673500	5.52778400	-1.18369000
C	6.14022500	5.57126800	1.20665500
C	6.46775600	6.21154300	0.01331200
H	6.51675800	6.00802000	-2.12312600
H	6.30975100	6.08371900	2.14962500
C	-5.52846400	-3.50712900	2.50529500
H	-6.10691000	-2.57736700	2.51887700
H	-5.84163600	-4.11339200	3.35592700
H	-4.47700300	-3.23933600	2.64786700
C	-5.27185600	-3.60320400	-2.52506500
H	-5.54953900	-4.22089000	-3.37976900
H	-5.79834000	-2.64709200	-2.61229100
H	-4.20049300	-3.39069400	-2.59213100
C	-7.01748400	-7.61528900	-0.01254000
H	-6.21742300	-8.34459800	0.14274400
H	-7.74665900	-7.75148600	0.78801400
H	-7.50020300	-7.84961400	-0.96239200
C	5.27182500	3.60321900	2.52455000
H	5.79849500	2.64721700	2.61186200
H	5.54931600	4.22099600	3.37924800
H	4.20050000	3.39049500	2.59151600
C	5.52841100	3.50706800	-2.50580600

H	5.84145500	4.11336700	-3.35645900
H	6.10696600	2.57737500	-2.51943800
H	4.47697300	3.23914500	-2.64829500
C	7.01750000	7.61524800	0.01194900
H	7.74672500	7.75138500	-0.78857000
H	6.21746700	8.34456800	-0.14342300
H	7.50016900	7.84961400	0.96181700
C	8.61374800	-5.11411200	0.02694300
C	7.98323900	-6.18044700	-0.88469000
H	7.87210400	-5.81941100	-1.90927100
H	8.61617100	-7.07182600	-0.90786500
H	6.99593300	-6.46921300	-0.51519700
C	9.99701000	-4.72642800	-0.52204200
H	10.46285800	-3.96635500	0.11022500
H	10.64848500	-5.60442800	-0.54333000
H	9.93062300	-4.33067200	-1.53766800
C	8.80041100	-5.72036400	1.42120500
H	9.26785200	-5.01117900	2.10904700
H	7.84851400	-6.04457800	1.85001800
H	9.45032700	-6.59559700	1.35031400
C	-8.61364200	5.11418500	-0.02604200
C	-7.98309900	6.18031100	0.88580300
H	-8.61602800	7.07168800	0.90918900
H	-7.87194000	5.81905400	1.91030400
H	-6.99580200	6.46915700	0.51634800
C	-9.99692400	4.72645600	0.52286900
H	-10.64835800	5.60448300	0.54432200
H	-10.46280500	3.96652300	-0.10954100
H	-9.93055200	4.33051500	1.53842400
C	-8.80027400	5.72071900	-1.42018700
H	-7.84836500	6.04496800	-1.84894200
H	-9.26775900	5.01169300	-2.10816400
H	-9.45014400	6.59597200	-1.34912200

12b

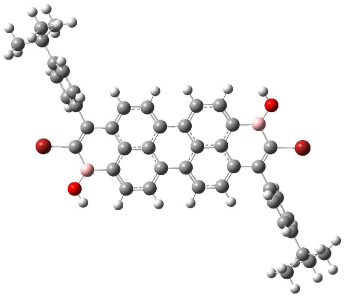

E (RM062X) = -7046.784607 Hartree

C	-5.11937500	1.04094900	-0.03353100
C	-3.76314700	0.45184400	-0.01394100
C	-2.57937700	1.25738500	-0.07896900
C	-1.30002300	0.62929700	-0.06495700
C	-1.19724700	-0.79482500	-0.02786700
C	-2.36101000	-1.53217700	0.06799800
H	-2.33089600	-2.61018700	0.13894300
C	-3.61908800	-0.91931300	0.07889300
H	-4.49694100	-1.54812300	0.15210200
C	0.12662000	-1.43930400	-0.09798000
C	1.30593000	-0.63787900	-0.06342800
C	1.20296700	0.78587200	-0.01409300
C	-0.12055300	1.43098100	-0.08513100
C	-0.24849100	2.80752900	-0.17436800
H	0.62447400	3.44278800	-0.22250100
C	-1.50649500	3.41155400	-0.21112800
H	-1.53958500	4.49472300	-0.28151000
C	-2.67570500	2.67497800	-0.15562900
C	-5.28117900	2.37925600	-0.10908100
C	2.36625400	1.52233500	0.09369300
H	2.33593800	2.59975600	0.17292400
C	3.62421700	0.90928600	0.10666900
H	4.50165400	1.53746600	0.18989200
C	3.76861500	-0.46112200	0.00407800
C	2.58529600	-1.26587400	-0.07589700
C	2.68221500	-2.68263800	-0.16645800

C	1.51337600	-3.41852900	-0.23653800
H	1.54683500	-4.50090100	-0.31819000
C	0.25511400	-2.81484800	-0.20069900
H	-0.61756000	-3.44952700	-0.26045300
B	-4.07714600	3.34772500	-0.18025400
Br	-7.03425900	3.13778700	-0.16735200
C	-6.27981400	0.10610900	0.03033000
C	-6.75040100	-0.52307300	-1.11645100
C	-6.91698600	-0.14540400	1.24542700
C	-7.84314300	-1.38527700	-1.05290000
H	-6.26459700	-0.33723000	-2.06854300
C	-8.00230800	-1.00725300	1.30160100
H	-6.56144800	0.34160900	2.14702000
C	-8.49233400	-1.64529700	0.15470400
H	-8.18246900	-1.85174100	-1.96870200
H	-8.47771100	-1.18026600	2.26083700
C	5.12491100	-1.05010900	-0.01124700
C	5.28755900	-2.38740000	-0.09993400
B	4.08394600	-3.35504300	-0.18987400
Br	7.04131300	-3.14473100	-0.15234100
C	6.28378600	-0.11444000	0.06730300
C	6.77102800	0.50967100	-1.08136100
C	6.89958700	0.14759900	1.28531800
C	7.85473300	1.37217000	-1.00266700
H	6.30007200	0.31483800	-2.03905000
C	7.98580700	1.01701900	1.35704400
H	6.53206300	-0.33345300	2.18535800
C	8.48735500	1.64614500	0.21702500
H	8.21354700	1.83835100	-1.91369500
H	8.43890700	1.19303100	2.32414700
O	4.30597400	-4.68844900	-0.28649600
H	3.50985400	-5.22377000	-0.34696800
O	-4.29878700	4.68214200	-0.26292700
H	-3.50242500	5.21842100	-0.31082600

C	9.67941900	2.60450900	0.25153500
C	9.23999300	3.98162900	-0.27330400
H	10.08614400	4.67398400	-0.25677900
H	8.87249000	3.92307700	-1.29994900
H	8.44420900	4.39567900	0.35120500
C	10.80054100	2.05093900	-0.64415400
H	11.66096400	2.72544300	-0.62423600
H	11.12488400	1.06833300	-0.29242900
H	10.47405000	1.95116200	-1.68138500
C	10.23714100	2.78287600	1.66690700
H	9.48701000	3.19475100	2.34685900
H	10.59800700	1.83693700	2.07906700
H	11.07968100	3.47762500	1.63758500
C	-9.69756900	-2.58125200	0.26655100
C	-10.90377000	-1.79149200	0.80192000
H	-11.77328400	-2.44958900	0.88335300
H	-10.70416300	-1.37210700	1.79025900
H	-11.15617300	-0.96938600	0.12746900
C	-9.36480900	-3.72542400	1.23863400
H	-10.21796800	-4.40432900	1.32041300
H	-8.50341800	-4.29651000	0.88271100
H	-9.13552700	-3.35011000	2.23815900
C	-10.08219500	-3.19105100	-1.08450800
H	-10.35675100	-2.42042900	-1.80936500
H	-9.26804400	-3.78798600	-1.50362300
H	-10.94489300	-3.84794900	-0.95129200

14

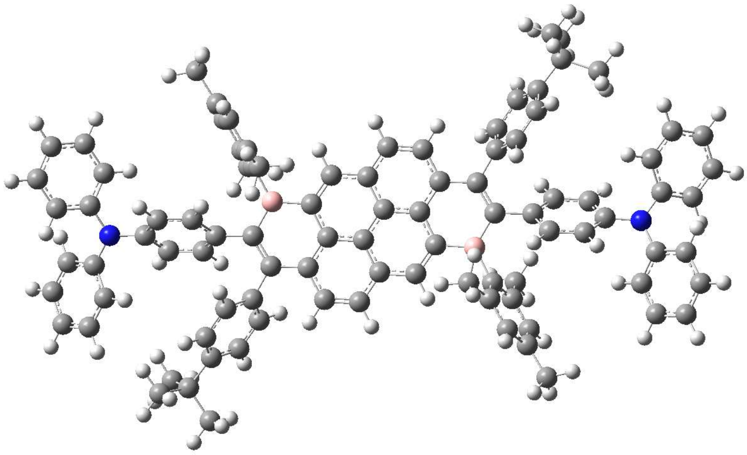

E (RM062X/6-31g(d)) = -3789.181331 Hartree

C	2.24745700	2.75471900	0.25317300
C	2.77373000	1.44876500	0.21612300
C	1.87380900	0.35667600	0.24934900
C	0.47450300	0.59572600	0.25200900
C	-0.02545700	1.92349800	0.26068700
C	0.88479000	2.98946900	0.27528300
C	2.36807300	-0.99720900	0.29971700
C	-0.44628700	-0.49724500	0.25428300
C	0.05293400	-1.82513400	0.28141100
C	1.46773600	-2.03193900	0.31338800
C	-0.85738900	-2.89124700	0.29920600
H	-0.48020600	-3.90941600	0.34121000
C	-2.21982900	-2.65653600	0.26545700
C	-2.74449600	-1.35071600	0.21239100
C	-1.84538000	-0.25803800	0.23786300
C	-2.34086100	1.09619000	0.26699900
C	-1.44049200	2.13087200	0.27729500
H	-1.81032900	3.15586600	0.30359800
H	2.92607100	3.59932200	0.27080200
H	0.50757800	4.00806800	0.30492400
H	1.83761900	-3.05638000	0.35564300
H	-2.89997900	-3.50011200	0.28647600

C	4.23539400	1.23850700	0.15993700
C	4.80817600	-0.00605600	0.22304600
C	-4.20459400	-1.14127100	0.14498200
C	-4.77866200	0.10453100	0.17129900
B	-3.86741900	1.35523600	0.29887100
B	3.89355800	-1.25549200	0.35478700
C	-4.46306300	3.44557300	1.70487200
C	-4.98177100	3.48893600	-0.66121100
C	-5.02593200	4.71545600	1.83646100
C	-5.52711700	4.76182700	-0.50284600
C	-5.56859500	5.38800200	0.74282500
H	-5.04354200	5.19030900	2.81609500
H	-5.93331700	5.27730700	-1.37186200
C	4.47312600	-2.71029300	0.52778900
C	4.47800600	-3.32755400	1.79176500
C	5.03166200	-3.39583000	-0.56562200
C	5.05681500	-4.58680200	1.94944700
C	5.59371600	-4.65845100	-0.38109700
C	5.62757800	-5.26570000	0.87368100
H	5.06639600	-5.04790600	2.93577800
H	6.01938500	-5.18018800	-1.23703000
C	-3.88287800	2.74926300	2.91532200
H	-2.78968900	2.69621600	2.85942500
C	-4.96716800	2.83215300	-2.02063000
H	-5.65069000	1.97471700	-2.04431700
C	-4.44704300	2.81188300	0.44971400
C	-6.20995700	6.74299100	0.90414500
H	-7.29399100	6.64896700	1.03707600
C	5.03119000	-2.75917600	-1.93466200
H	4.03831100	-2.37470500	-2.19529000
C	3.87334700	-2.62151300	2.98445600
H	2.78121600	-2.57296600	2.90860000
C	6.29036300	-6.60609800	1.06683200
H	6.20808300	-7.22344100	0.16772200

C	-5.07972200	-2.34906900	0.04641600
C	-5.05452600	-3.17550200	-1.08234200
C	-6.02749300	-2.61263900	1.03375300
C	-5.98817300	-4.19170700	-1.23570100
H	-4.31942800	-2.99198200	-1.86265300
C	-6.95473400	-3.63960700	0.88016600
H	-6.06548400	-1.97845000	1.91504400
C	-6.97237000	-4.43529900	-0.26778700
H	-5.96018500	-4.79421700	-2.13990600
H	-7.68297800	-3.79844100	1.66799700
C	5.10448600	2.44764800	0.03276400
C	6.05619500	2.74101700	1.01439300
C	5.05523800	3.26046900	-1.09986300
C	6.94323100	3.79404800	0.84795400
H	6.11487500	2.11469800	1.90010700
C	5.95613300	4.31078200	-1.26726600
H	4.32153300	3.05189600	-1.87513500
C	6.92635300	4.59365300	-0.30342500
H	7.67599500	3.98605600	1.62743500
H	5.89696300	4.90233200	-2.17431900
H	-4.14786900	3.27104600	3.83882300
H	-4.24390200	1.71618000	2.99669600
H	-5.27536900	3.52971800	-2.80416800
H	-3.96684900	2.46146900	-2.27304400
H	-5.81567600	7.26828900	1.77839000
H	-6.04119300	7.36985700	0.02372300
H	4.12387800	-3.13282600	3.91777700
H	4.22918800	-1.58622000	3.06177600
H	5.32879800	-3.47394600	-2.70678400
H	5.73014200	-1.91469200	-1.96968300
H	7.35737600	-6.48541400	1.28744600
H	5.84332000	-7.15496000	1.90044700
C	7.96570300	5.70407500	-0.46825400
C	9.36947500	5.07363100	-0.45712800

H	10.13379300	5.84674400	-0.59902800
H	9.57409400	4.56815500	0.49271700
H	9.47010000	4.33480300	-1.25948600
C	7.79149100	6.46855100	-1.78415100
H	6.80995100	6.95081900	-1.84265800
H	8.55324100	7.25141200	-1.85626500
H	7.90639500	5.80981300	-2.65130500
C	7.84337500	6.70430900	0.69298300
H	6.85124000	7.16715500	0.70303000
H	8.00012100	6.22054300	1.66176600
H	8.59219000	7.49739500	0.58840200
C	-8.02733900	-5.51522000	-0.51432600
C	-9.01991400	-5.62552300	0.64713800
H	-9.55420900	-4.68287100	0.81064600
H	-9.76294000	-6.39773000	0.42253200
H	-8.52084200	-5.90310800	1.58168100
C	-7.34294100	-6.87938400	-0.69880200
H	-8.09280000	-7.65566700	-0.88794600
H	-6.64847700	-6.87126800	-1.54438800
H	-6.77998300	-7.15604700	0.19855400
C	-8.81256100	-5.15675400	-1.78810200
H	-9.28055700	-4.17098500	-1.69118000
H	-8.16278000	-5.13524500	-2.66843600
H	-9.59964800	-5.89786500	-1.96954600
C	6.28218800	-0.18879500	0.10608000
C	6.98521700	-0.92712000	1.06513200
C	7.00510000	0.31346800	-0.98526400
C	8.35353700	-1.13598200	0.95793500
H	6.45402800	-1.33558900	1.92004900
C	8.36642400	0.08255000	-1.12000500
H	6.48408300	0.87949300	-1.75238700
C	9.05796300	-0.64417400	-0.14451500
H	8.88279100	-1.69906000	1.72036500
H	8.90425700	0.46116800	-1.98386100

C	-6.25400400	0.26815600	0.04225600
C	-6.98045600	0.98439700	0.99970500
C	-6.96092000	-0.26749300	-1.04371900
C	-8.36028700	1.11710000	0.91007500
H	-6.46036900	1.42505100	1.84569000
C	-8.33391300	-0.11276200	-1.16118400
H	-6.42005900	-0.81102100	-1.81330700
C	-9.05294500	0.57015700	-0.17374900
H	-8.90919000	1.65395100	1.67750700
H	-8.86159900	-0.52621500	-2.01534200
N	-10.45860500	0.71353800	-0.27694100
N	10.44564500	-0.89510200	-0.27863200
C	11.30508400	0.12462100	-0.74885100
C	12.37019900	-0.18122100	-1.60517900
C	11.10099400	1.45695600	-0.36640300
C	13.22088000	0.82306800	-2.05220900
H	12.52537800	-1.21055000	-1.91253100
C	11.95023800	2.45514500	-0.83080100
H	10.27231800	1.70127400	0.29191600
C	13.01775500	2.14710300	-1.67106600
H	14.04213200	0.56659000	-2.71470200
H	11.78219900	3.48193200	-0.51867200
H	13.68177500	2.92895900	-2.02480200
C	10.95425400	-2.17856200	0.03412600
C	12.20132900	-2.31775000	0.65519000
C	10.21047800	-3.32703000	-0.26518600
C	12.69609200	-3.58151300	0.95624400
H	12.77664700	-1.42951300	0.89670300
C	10.70667000	-4.58462600	0.05772600
H	9.23977400	-3.22534800	-0.74063200
C	11.95299400	-4.72286400	0.66433800
H	13.66565000	-3.67091800	1.43696100
H	10.11527100	-5.46397300	-0.18005800
H	12.33945800	-5.70702500	0.90824000

C	-11.06137800	1.94770400	0.06628100
C	-12.28729200	1.97881100	0.74158800
C	-10.43088000	3.15535400	-0.25763500
C	-12.87310500	3.19544800	1.07212200
H	-12.77453800	1.04436600	1.00178500
C	-11.01608100	4.36576800	0.09503900
H	-9.47772700	3.13559100	-0.77693600
C	-12.24188900	4.39616400	0.75601200
H	-13.82482500	3.20151900	1.59488500
H	-10.51168400	5.29277000	-0.16110900
H	-12.69868900	5.34346000	1.02316700
C	-11.24902100	-0.37358500	-0.71584700
C	-12.36819100	-0.15319800	-1.52858300
C	-10.92550200	-1.68646200	-0.34670700
C	-13.15397900	-1.22090400	-1.94627700
H	-12.61620700	0.86085800	-1.82561900
C	-11.71194500	-2.74788000	-0.78179100
H	-10.05415600	-1.86589800	0.27707800
C	-12.83234800	-2.52508100	-1.57902300
H	-14.01810400	-1.02918500	-2.57525400
H	-11.45192300	-3.75966200	-0.48285500
H	-13.44526100	-3.35696200	-1.90995900

9a

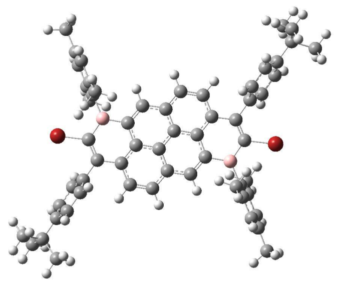

E (RM062X/6-31g(d)) = -7435.152893 Hartree

C	-2.85758900	2.04227500	-0.04742400
C	-3.02893400	0.64566000	-0.07208400
C	-1.88210300	-0.18427200	-0.09293100

C	-0.59079200	0.40555400	-0.09999300
C	-0.44912500	1.81700500	-0.08319200
C	-1.60008800	2.61670000	-0.05643900
C	-2.01077000	-1.62169800	-0.08401400
C	0.58046500	-0.41469100	-0.10637000
C	0.43878000	-1.82621400	-0.09842400
C	-0.87477200	-2.39086000	-0.08448100
C	1.58987500	-2.62608900	-0.08431600
H	1.48370000	-3.70731400	-0.06825500
C	2.84747900	-2.05175500	-0.07920900
C	3.01877900	-0.65505000	-0.09509600
C	1.87180200	0.17505200	-0.10306700
C	2.00032600	1.61243000	-0.08474300
C	0.86444200	2.38161600	-0.07337100
H	0.96023100	3.46689900	-0.04854200
H	-3.73130200	2.68250600	-0.01211800
H	-1.49386100	3.69780000	-0.03354300
H	-0.97025400	-3.47632600	-0.06638400
H	3.72152800	-2.69194700	-0.05405400
C	-4.39415000	0.07812000	-0.04076000
C	-4.57965100	-1.26840000	-0.00158100
C	4.38400300	-0.08745000	-0.06835100
C	4.56920800	1.25886500	-0.02151400
B	3.40262100	2.26665900	-0.03553000
B	-3.41299000	-2.27623500	-0.03090400
C	3.57904800	4.59746400	-1.15146400
C	3.85668700	4.44461800	1.25144000
C	3.75667700	5.97800300	-1.06875200
C	4.02520000	5.82667300	1.30592600
C	3.97088300	6.61203700	0.15448900
H	3.73620000	6.57245200	-1.98069700
H	4.20999800	6.30226900	2.26776900
C	-3.62107600	-3.82937400	0.01182400
C	-3.57399200	-4.60313900	-1.15897000

C	-3.86365400	-4.46188600	1.24337100
C	-3.73960600	-5.98581300	-1.08186200
C	-4.01994500	-5.84536200	1.29201700
C	-3.95955000	-6.62541500	0.13707100
H	-3.70211400	-6.57763500	-1.99499900
H	-4.19666300	-6.32702800	2.25238700
C	3.40279600	3.92330300	-2.49112400
H	2.48497700	3.32503900	-2.52187200
C	3.96755700	3.60540100	2.50168000
H	4.82913200	2.93039500	2.42265700
C	3.61240400	3.81924400	0.01703400
C	4.12313400	8.11005400	0.23271900
H	4.75650400	8.40065300	1.07556800
C	-3.97415600	-3.63029300	2.49878100
H	-3.08245300	-3.01035800	2.65161200
C	-3.38561900	-3.92553200	-2.49534100
H	-2.46575100	-3.33021300	-2.51848000
C	-4.16370200	-8.11791400	0.20278500
H	-3.65790000	-8.62487300	-0.62371700
C	5.53441500	-1.03612100	-0.07183200
C	6.28993500	-1.22836400	1.08772000
C	5.87941500	-1.74611400	-1.21901700
C	7.35476900	-2.11681900	1.09210100
H	6.03855800	-0.67007600	1.98532900
C	6.95831400	-2.62932500	-1.20965500
H	5.30241700	-1.60650400	-2.12968300
C	7.71605500	-2.83656100	-0.05584400
H	7.91975200	-2.24879100	2.01119100
H	7.19923700	-3.15860800	-2.12499300
C	-5.54243700	1.02926600	-0.03428700
C	-5.88781700	1.74732300	-1.18188200
C	-6.29085700	1.22152800	1.12435400
C	-6.95664100	2.63362700	-1.15966200
H	-5.31506500	1.60681800	-2.09502100

C	-7.35476100	2.11969000	1.14154000
H	-6.04014000	0.65834800	2.01913500
C	-7.71135400	2.84462100	0.00170300
H	-7.20549600	3.17379800	-2.06933900
H	-7.90870000	2.24532500	2.06539500
Br	6.33419200	1.96683300	0.02811000
Br	-6.34474700	-1.97584800	0.05424200
H	3.36154500	4.65084300	-3.30604400
H	4.24103700	3.24332300	-2.68834800
H	4.10206800	4.22470600	3.39253300
H	3.07498400	2.98602000	2.65163000
H	4.56611100	8.51214800	-0.68279500
H	3.15067700	8.59708100	0.36887600
H	-3.34108700	-4.65126800	-3.31169700
H	-4.21992200	-3.24226100	-2.69736900
H	-4.10641000	-4.25550500	3.38583700
H	-4.83696000	-2.95636400	2.42524800
H	-3.78188300	-8.52958200	1.14149700
H	-5.22845100	-8.37069000	0.14246500
C	-8.87482500	3.83837800	-0.02246000
C	-9.91194600	3.38001300	-1.06123200
H	-10.75082100	4.08442400	-1.09416800
H	-9.47965300	3.32454200	-2.06511100
H	-10.30305200	2.38940000	-0.80761600
C	-9.56932500	3.94761200	1.33836100
H	-8.87923600	4.29218900	2.11602200
H	-10.38771200	4.67179800	1.27347000
H	-9.99534300	2.98908900	1.65295000
C	-8.34796100	5.23077200	-0.40778100
H	-7.60404800	5.57889100	0.31635300
H	-7.88065900	5.22654600	-1.39737600
H	-9.17198900	5.95284300	-0.42879000
C	8.90321000	-3.80079800	-0.00032700
C	9.15394100	-4.48683300	-1.34670600

H	9.37933500	-3.75900600	-2.13340400
H	10.01179900	-5.16142500	-1.25967200
H	8.29138400	-5.08318000	-1.66278900
C	8.62566800	-4.88695400	1.05270600
H	9.46967600	-5.58367400	1.10751800
H	8.48038700	-4.45558000	2.04780500
H	7.72604300	-5.45579800	0.79585900
C	10.17133200	-3.02354100	0.39049100
H	10.39014200	-2.24368300	-0.34603800
H	10.06470500	-2.54384900	1.36809700
H	11.02968400	-3.70283500	0.44021500

PDI

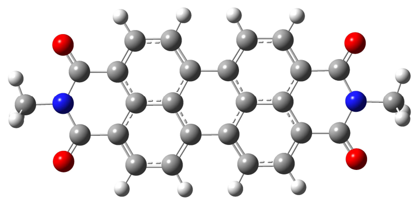

E (RM062X) = -1409.56048625 Hartree

C	-1.47250000	2.42323900	-0.03806400
C	-0.73691500	1.24925200	-0.00992100
C	0.73691800	1.24925300	0.01005100
C	1.47250300	2.42324000	0.03821400
C	-1.42891800	0.00168100	0.00066300
C	1.42892000	0.00168400	-0.00057400
C	0.73697700	-1.24614600	-0.01065100
C	-0.73697400	-1.24614700	0.01074500
C	1.47228200	-2.42042100	-0.03974300
C	2.87208100	-2.40987200	-0.04626200
C	3.55610000	-1.21812300	-0.02347300
C	2.84801500	0.00181500	-0.00094200
C	3.55769600	1.22131100	0.02101200
C	2.87227000	2.41257100	0.04376400
H	3.42828600	3.34131900	0.06474200
H	3.42889700	-3.33823300	-0.06767800

C	-2.87226800	2.41256400	-0.04365800
C	-3.55769400	1.22130300	-0.02097300
C	-2.84801300	0.00180800	0.00097600
H	-3.42828400	3.34131200	-0.06462600
C	-2.87207500	-2.40987900	0.04632900
C	-1.47227600	-2.42042400	0.03983600
H	-3.42889100	-3.33823900	0.06774900
H	-0.97713800	3.38327400	-0.05873700
H	0.97713900	3.38327400	0.05893300
H	0.97664000	-3.38030100	-0.06023700
H	-0.97663100	-3.38030100	0.06034200
C	-5.04223500	1.24114500	-0.02340400
C	-3.55609800	-1.21813300	0.02350300
C	-5.03839000	-1.22959900	0.02471100
C	5.04223600	1.24114800	0.02334700
C	5.03838600	-1.22960000	-0.02475000
N	5.68473900	0.00348300	0.00120900
N	-5.68474200	0.00347900	-0.00142600
C	7.14872600	-0.03616500	0.00365700
H	7.49565600	-0.58921300	0.87569300
H	7.50127700	-0.54019300	-0.89548000
H	7.51316400	0.98447100	0.03281400
C	-7.14872300	-0.03615100	-0.00404500
H	-7.49544100	-0.59069700	-0.87519700
H	-7.50152600	-0.53861000	0.89588700
H	-7.51316300	0.98443300	-0.03498400
O	-5.67065000	2.27718600	-0.04342800
O	-5.68357200	-2.25588400	0.04611800
O	5.67064400	2.27719400	0.04337900
O	5.68356200	-2.25589100	-0.04604400

Anthracene-diborinic acid

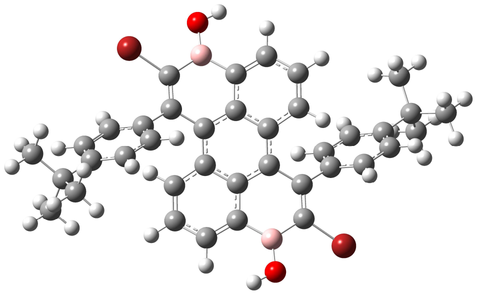

E (RM062X) = -6816.91080344 Hartree

C	3.46734100	2.79409200	1.01609400
C	2.76622100	1.62898500	1.09407800
C	1.49589200	1.45389200	0.44945100
C	0.88795400	2.62922100	-0.10728500
C	1.68775100	3.81503800	-0.27307400
C	2.95115000	3.86926100	0.25814900
C	0.81451300	0.20790800	0.43482400
C	-0.46971700	2.62077100	-0.50730200
C	-1.15206100	1.37646300	-0.52094800
C	-0.54213700	0.19840100	0.02747100
C	-1.34110600	-0.99005800	0.17770200
C	-2.60509400	-1.03904100	-0.35329100
C	-3.12642600	0.04492600	-1.09453700
C	-2.42544700	1.21063400	-1.16097600
H	4.41605800	2.89239500	1.52903200
H	3.16864900	0.82849400	1.69237900
H	3.55872000	4.76216000	0.14315300
H	-3.21053900	-1.93475000	-0.24991100
H	-4.07753100	-0.04758200	-1.60415200
H	-2.82974000	2.01895100	-1.74760200
C	1.52045700	-1.07803900	0.73208000
C	-1.17587900	3.90693500	-0.80161700
C	-0.45490700	5.00619300	-1.12286300
C	0.79936000	-2.18590400	1.02463100
B	1.08820300	5.02052600	-1.03774700
B	-0.74200300	-2.20653100	0.92394800
C	-2.64562800	3.98943800	-0.55561600

C	-3.14409400	3.65176600	0.69846200
C	-3.54634400	4.39597300	-1.54302300
C	-4.51044100	3.71084000	0.96188200
H	-2.46137800	3.32897700	1.47739500
C	-4.90603000	4.43332000	-1.28108500
H	-3.17957200	4.65584200	-2.52948300
C	-5.42179300	4.09121700	-0.02282800
H	-4.85345400	3.44207700	1.95244800
H	-5.57828400	4.73467900	-2.07681400
C	2.99684700	-1.15221800	0.52829000
C	3.86404600	-1.56399200	1.53613200
C	3.53549600	-0.79724900	-0.70980800
C	5.23829300	-1.59559700	1.32237000
H	3.46656700	-1.83556100	2.50741000
C	4.90496000	-0.84673100	-0.92189100
H	2.87653500	-0.47020300	-1.50711500
C	5.78985900	-1.23698100	0.09076700
H	5.87689400	-1.90598800	2.13891500
H	5.28971800	-0.56461200	-1.89557400
Br	-1.27209900	6.71322600	-1.40005800
Br	1.61819700	-3.89385100	1.29398100
O	-1.44671800	-3.26399300	1.39297000
H	-2.39913000	-3.18183500	1.28666600
O	1.79457500	6.06563400	-1.53084500
H	2.74736000	5.97874200	-1.43130000
C	7.29451700	-1.25329000	-0.18326500
C	-6.93213500	4.13800000	0.21336800
C	-7.62055900	3.15072500	-0.74441500
H	-8.70259800	3.17108700	-0.58824100
H	-7.42469900	3.40224900	-1.78899100
H	-7.26569700	2.13211600	-0.56641500
C	-7.30481700	3.75919100	1.64938200
H	-6.85510500	4.44245900	2.37436000
H	-8.38969200	3.81312500	1.76586500

H	-6.99225000	2.74016600	1.89094400
C	-7.44758100	5.56072900	-0.06177400
H	-6.96529600	6.28073400	0.60424800
H	-7.25515500	5.86718100	-1.09187000
H	-8.52704100	5.60498800	0.10698800
C	7.75429100	0.16445100	-0.56363400
H	8.83001800	0.16743500	-0.75935900
H	7.24651200	0.52401100	-1.46113300
H	7.54981100	0.86682500	0.24866100
C	8.09907900	-1.71196200	1.03677800
H	7.82758900	-2.72660000	1.33911100
H	9.16311500	-1.70938700	0.78893200
H	7.95222000	-1.04413400	1.88956300
C	7.58770600	-2.21515400	-1.34700600
H	7.26700100	-3.22970400	-1.09745200
H	7.07347100	-1.91028200	-2.26077400
H	8.66133300	-2.23370700	-1.55338100

Diphenylacetylene-1,2-cis-BBr_3

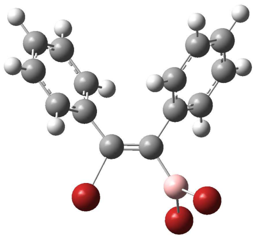

E (RM062X) = -8286.843665 Hartree

C	-4.24977300	0.08799600	-1.14161100
C	-2.86424900	0.01359700	-1.08038300
C	-2.24594800	-0.77758700	-0.10749900
C	-3.03310400	-1.50236300	0.79038300
C	-4.41827700	-1.41639600	0.73228500
C	-5.02939800	-0.62174400	-0.23302800

H	-4.72066200	0.69843800	-1.90249400
H	-2.25530700	0.56546100	-1.78667200
H	-2.55849100	-2.12743800	1.53770700
H	-5.02025600	-1.97442300	1.43902000
C	-0.76997300	-0.82024600	-0.03882400
C	0.07750200	0.21371700	-0.06655200
C	-0.38328200	1.63319800	-0.02880800
C	-1.24515500	2.07222600	0.98073200
C	0.08937000	2.55010200	-0.97150100
C	-1.64295500	3.40134100	1.03150900
H	-1.60056800	1.36464900	1.72148200
C	-0.31515500	3.88036300	-0.92013300
H	0.75835400	2.21964200	-1.75877800
C	-1.18153800	4.30802400	0.07953500
H	-2.31148300	3.73190200	1.81737100
H	0.04931000	4.58116300	-1.66157800
B	1.62087500	0.02795200	-0.01082000
Br	-0.02008600	-2.58427300	0.11919500
Br	2.64930400	-0.32974900	-1.58496200
Br	2.52592900	0.28213800	1.65580400
H	-1.49385600	5.34457500	0.12138900
H	-6.10998400	-0.56117100	-0.28129600

Diphenylacetylene-1,1-BBr_3

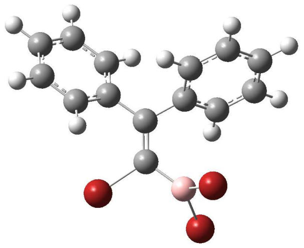

E (RM062X) = -8286.845310 Hartree

C	-4.36193100	-1.00987600	1.02295000
C	-2.98954700	-0.79555300	0.98008700
C	-2.42461700	-0.06416600	-0.06773100

C	-3.25474300	0.46162000	-1.06177600
C	-4.62389700	0.22858200	-1.02776600
C	-5.17949200	-0.50672000	0.01533100
H	-4.79361100	-1.56796700	1.84499000
H	-2.35274800	-1.17959300	1.76792800
H	-2.82033800	1.04810300	-1.86395600
H	-5.25807600	0.62687600	-1.81038000
C	-0.96854500	0.21870800	-0.11102000
C	0.01395500	-0.69323500	0.00301100
B	1.53162300	-0.39476800	0.12934000
Br	2.19010300	0.65182900	1.59477800
Br	2.80457500	-1.14692200	-1.09103400
Br	-0.34495600	-2.57406800	0.06032700
C	-0.57294400	1.63826600	-0.31024500
C	-1.14407000	2.63913200	0.48322000
C	0.38373600	1.98622300	-1.26741300
C	-0.72218600	3.95423300	0.35561900
H	-1.90083600	2.37483900	1.21346100
C	0.79668500	3.30842800	-1.40101300
H	0.78125500	1.22489600	-1.93011200
C	0.25316700	4.29088600	-0.58269800
H	-1.15219300	4.72068900	0.98896700
H	1.53636400	3.56775700	-2.14867600
H	0.57678300	5.31993300	-0.68189200
H	-6.24842300	-0.68022200	0.04712900

Ditolylacetylene-1,2-cis-BBr_3

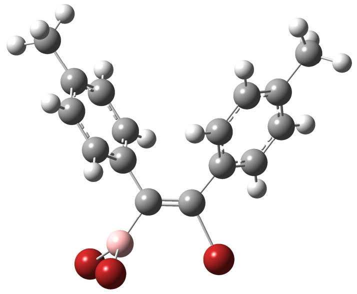

E (RM062X) = -8365.45800774 Hartree

C	3.93881600	-0.38103900	1.06319300
C	2.55083100	-0.35428400	1.02151600
C	1.85869800	-1.11676000	0.07811100
C	2.58556000	-1.91412800	-0.81068100
C	3.97140200	-1.92608400	-0.76791100
C	4.67175900	-1.16181600	0.16945400
H	4.46047700	0.21289700	1.80565300
H	1.99808400	0.25878800	1.72357800
H	2.06192000	-2.51781200	-1.54303400
H	4.52229000	-2.54003300	-1.47262700
C	0.38417100	-1.05111900	0.02654900
C	-0.38786400	0.03956400	0.06140100
C	0.17474600	1.42156000	0.01807400
C	1.01969700	1.81037200	-1.02535000
C	-0.16416600	2.35817800	0.99583700
C	1.52751600	3.10006900	-1.07221800
H	1.28115900	1.09107300	-1.79374100
C	0.35497300	3.64740100	0.94413600
H	-0.81377500	2.07234000	1.81607300
C	1.20564000	4.03961200	-0.08873600
H	2.18595300	3.38548600	-1.88587600
H	0.09572900	4.35839200	1.72137300
B	-1.93951500	-0.02898600	0.02325000
Br	-0.49825000	-2.75725900	-0.11123600
Br	-2.97403000	-0.21437000	1.62473600
Br	-2.84363700	0.21561800	-1.64619100
C	6.17591000	-1.20417200	0.21951100

H	6.52102500	-2.17232500	0.59157200
H	6.60307500	-1.06494300	-0.77563600
H	6.57062300	-0.42940200	0.87698600
C	1.74030400	5.44555700	-0.16230800
H	1.10032800	6.06609400	-0.79576800
H	1.77607600	5.90507700	0.82612300
H	2.74372200	5.46219800	-0.59020600

Ditolylacetylene-1,1-BBr_3

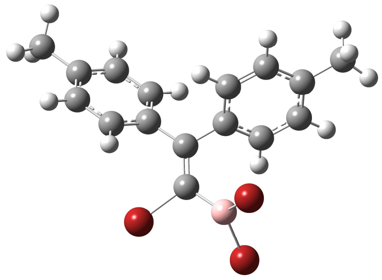

E (RM062X) = -8365.46002031 Hartree

C	-4.31450700	-0.51458100	0.96501000
C	-2.92480300	-0.54019800	0.94880400
C	-2.21789600	0.14441300	-0.04065000
C	-2.93371800	0.86842400	-1.00074100
C	-4.31983100	0.87091400	-0.99087800
C	-5.03380300	0.17962200	-0.00747300
H	-4.84791500	-1.04280100	1.74790500
H	-2.38608100	-1.07949700	1.71866000
H	-2.39519500	1.42075400	-1.76317900
H	-4.86020000	1.42155400	-1.75385200
C	-0.73637800	0.16739500	-0.06778500
C	0.07316600	-0.90678600	0.00894800
B	1.61724500	-0.87859800	0.13794700
Br	2.46448400	0.02055300	1.60586900
Br	2.74108700	-1.84327700	-1.08440300
Br	-0.60724400	-2.69961400	-0.01822800
C	-0.09292400	1.49734800	-0.22045200
C	-0.46502200	2.55982300	0.61229000

C	0.91133600	1.70401700	-1.16768900
C	0.19600100	3.77329500	0.53047400
H	-1.25502600	2.41564100	1.34111300
C	1.56208500	2.93141300	-1.25217900
H	1.16680700	0.90862500	-1.86011000
C	1.22424100	3.97847000	-0.39928900
H	-0.08286800	4.58178800	1.19819700
H	2.33830900	3.07451900	-1.99594200
C	1.91490200	5.31351000	-0.48155400
H	1.23299700	6.07430900	-0.87009000
H	2.24230700	5.64186900	0.50694400
H	2.78456100	5.26987200	-1.13708700
C	-6.53937800	0.19979200	-0.00454900
H	-6.91283600	1.22130100	0.09868900
H	-6.93228000	-0.19828500	-0.94304700
H	-6.94110000	-0.39603600	0.81498200

Bis-(*p*-fluorophenyl)acetylene-1,2-BBr_3

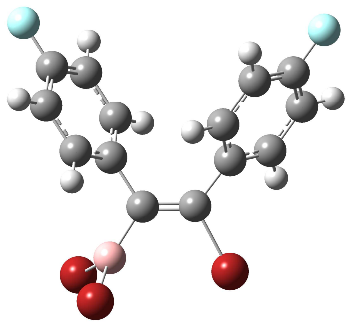

E (RM062X) = -8485.32004345 Hartree

C	3.94195700	-0.35615000	1.08089500
C	2.55562600	-0.33959600	1.03233400
C	1.86705700	-1.10460800	0.08529600
C	2.58911300	-1.90217200	-0.80609400
C	3.97632600	-1.92121800	-0.77282000
C	4.62529900	-1.14506200	0.17163300
H	4.49245900	0.22278900	1.81102900
H	2.00139200	0.26751700	1.73751900

H	2.06424100	-2.50682600	-1.53589400
H	4.55437300	-2.52562700	-1.45977800
C	0.39141900	-1.04128600	0.03438800
C	-0.37524200	0.05313500	0.05829500
C	0.20139700	1.42908500	0.00703100
C	1.04187700	1.80035500	-1.04700800
C	-0.11928400	2.36856600	0.98956800
C	1.57600500	3.07904200	-1.10824700
H	1.28011400	1.07775200	-1.81898200
C	0.41325800	3.65175500	0.94265800
H	-0.76936500	2.09205000	1.81179100
C	1.25327700	3.97891100	-0.10605100
H	2.22989600	3.38532300	-1.91457300
H	0.18932700	4.38974100	1.70219200
B	-1.92860100	-0.00849000	0.01803100
Br	-0.49072300	-2.74445300	-0.08724500
Br	-2.96250000	-0.16622300	1.62037900
Br	-2.82408400	0.22121500	-1.65615000
F	5.96740500	-1.16475000	0.21306800
F	1.77457200	5.21656300	-0.15631000

Bis-(*p*-fluorophenyl)acetylene-1,1-BBr_3

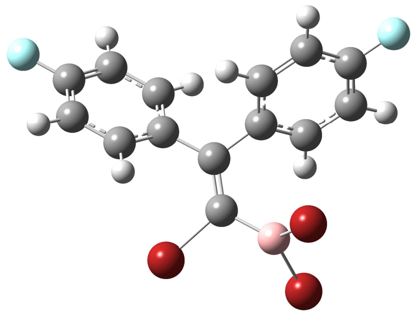

E (RM062X) = -8485.32183890 Hartree

C	-4.30512900	-0.58843500	0.97536100
C	-2.91732600	-0.57790700	0.95161600
C	-2.22770400	0.11431900	-0.04787100
C	-2.95249300	0.81493500	-1.01713000
C	-4.34009300	0.79710300	-1.01706500
C	-4.98846200	0.09266700	-0.01728200
H	-4.85710900	-1.10949400	1.74693500
H	-2.36651800	-1.09717000	1.72579300
H	-2.42414200	1.36980900	-1.78398000
H	-4.91746300	1.32003600	-1.76840400
C	-0.74637700	0.16708600	-0.07272400
C	0.08188100	-0.89080000	0.00990200
B	1.62734900	-0.83739800	0.13794500
Br	2.45362200	0.06500900	1.61265500
Br	2.76304100	-1.75439900	-1.10632900
Br	-0.56310400	-2.69466100	0.00189900
C	-0.13073700	1.51118700	-0.22448800
C	-0.52792700	2.56082600	0.61225500
C	0.86891900	1.73616900	-1.17498600
C	0.10083200	3.79315600	0.54061700
H	-1.31532400	2.39903500	1.33935500
C	1.50104200	2.97100700	-1.26500100
H	1.13868700	0.94729800	-1.86826600
C	1.11040400	3.97062000	-0.39485600
H	-0.17341200	4.61312100	1.19185000
H	2.27427000	3.16362000	-1.99741900
F	1.71695000	5.16575600	-0.46428300
F	-6.33153800	0.07768200	-0.00389100

S10: detailed Studies on Compound 14:

Theoretical calculations:

All ground state optimizations have been carried using in Gaussian 09[11] with the PBE0 functional and the Pople 6-31G(d,p) basis set.[14] Excited state calculations have been performed using Time-Dependent DFT (TD-DFT) within the Tamm-Dancoff approximation (TDA)[15] using the same functional and basis set for ground state geometry optimization.

Photophysical measurements:

The sample solutions for the emission spectra were prepared in spectroscopy grade solvents. Absorption spectra were recorded at room temperature using a Shimadzu UV-1800 double beam spectrophotometer. The sample solutions for the emission spectra were prepared in DCM and degassed via three freeze-pump-thaw cycles using a quartz cuvette designed in-house. Steady-state emission and time-resolved emission spectra were recorded at 298 K using an Edinburgh Instruments FLS980 fluorimeter. All samples for steady-state measurements were excited at 370 nm using a xenon lamp, while samples for time-resolved measurements were excited at 378 nm using a PicoQuant, LDH-D-C-375 pulsed diode laser. The thin films were prepared by spin-coating the samples from a solution of chlorobenzene of the desired sample on a quartz substrate. Solid-state FPL measurements of thin films were performed in an integrating sphere [16] under a nitrogen atmosphere in a Hamamatsu C9920-02 luminescence measurement system. Molar absorptivity determination was verified by linear least-squares fit of values obtained from at least five independent solutions at varying concentrations with absorbance ranging from 1.1×10^{-6} to 8.0×10^{-1} M^{-1} cm^{-1}.

---

[14] C. Adamo, V. Barone, J. Chem. Phys. 1999, 110, 6158; b) A. J. Pople, J. S. Binkley, R. Seeger, J. Quant. Chem. Symp. 1976, 10, 1.

[15] S. Grimme, Chem. Phys. Lett. 1996, 259, 128; b) S. Hirata, M. Head-Gordon, Chem. Phys. Lett. 1999, 314, 291.

[16] N. C. Greenham, I. D. W. Samuel, G. R. Hayes, R. T. Phillips, Y. A. R. R. Kessener, S. C. Moratti, A. B. Holmes, R. H. Friend, Chem. Phys. Lett. 1995, 241, 89.

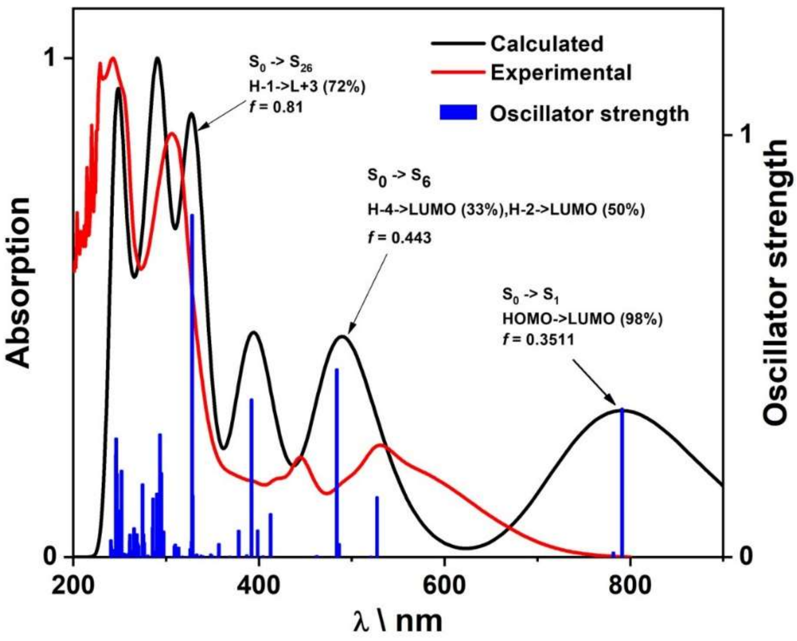

Figure S157. TDA simulation and experimental UV-Vis spectra of Compound **14.**

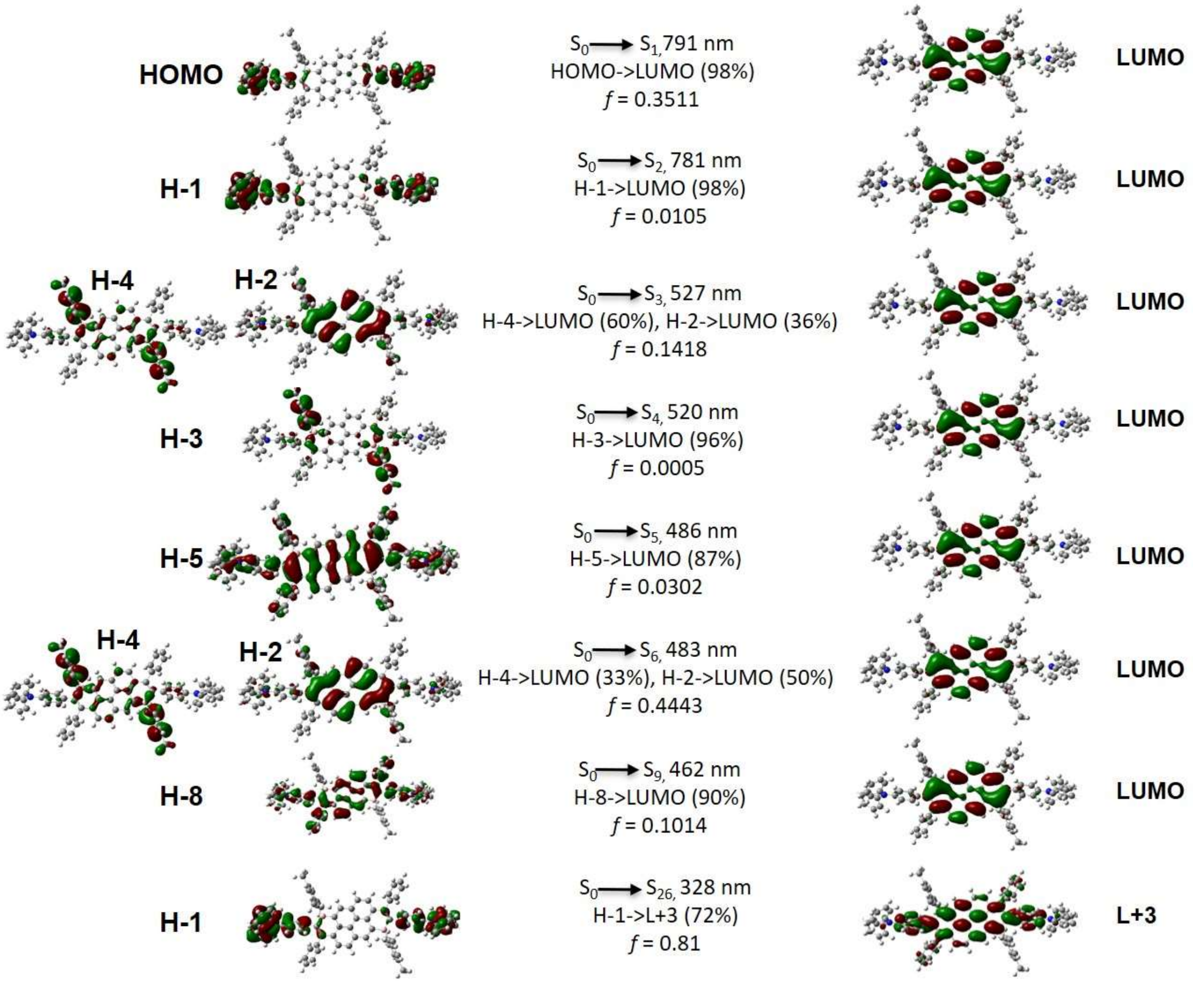

Figure S158. The frontier molecular orbitals of Compound **14** involved in the dominant electronic transitions.

Table S8 Summary of solution and solid state photophysical properties of Compound 14

Compound	$\lambda_{abs}{}^{a}$ (nm)	λ_{ex} (nm)	$\lambda_{em}{}^{a}$ (nm)	$\lambda_{em}{}^{b}$ (nm)	$\lambda_{em}{}^{c}$ (nm)	$\lambda_{em}{}^{d}$ (nm)	$\lambda_{em}{}^{e}$ (nm)
**14**	241, 312, 444, 530	370	512,558	442,638	472,726	434, 793	824

[a]Measured in DCM; [b]0.1 wt%, [c]1 wt% and [d]10 wt% emitter in PMMA film; [e]Neat film.

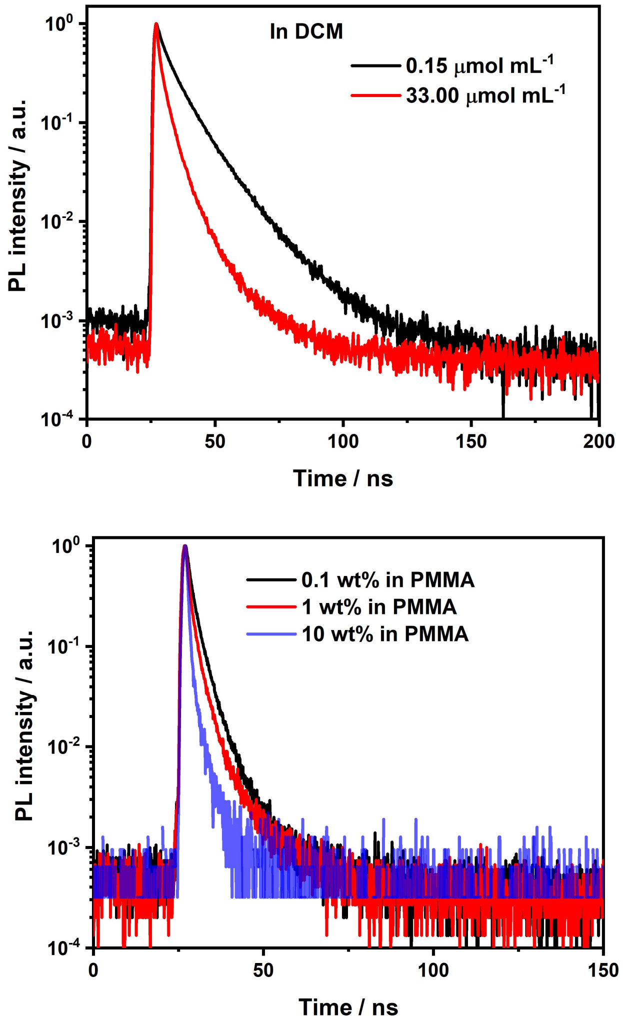

Figure S159. Time-resolved PL spectra of Compound **14** (a) in DCM with different concentration and (b) doped films with different doping concentrations.

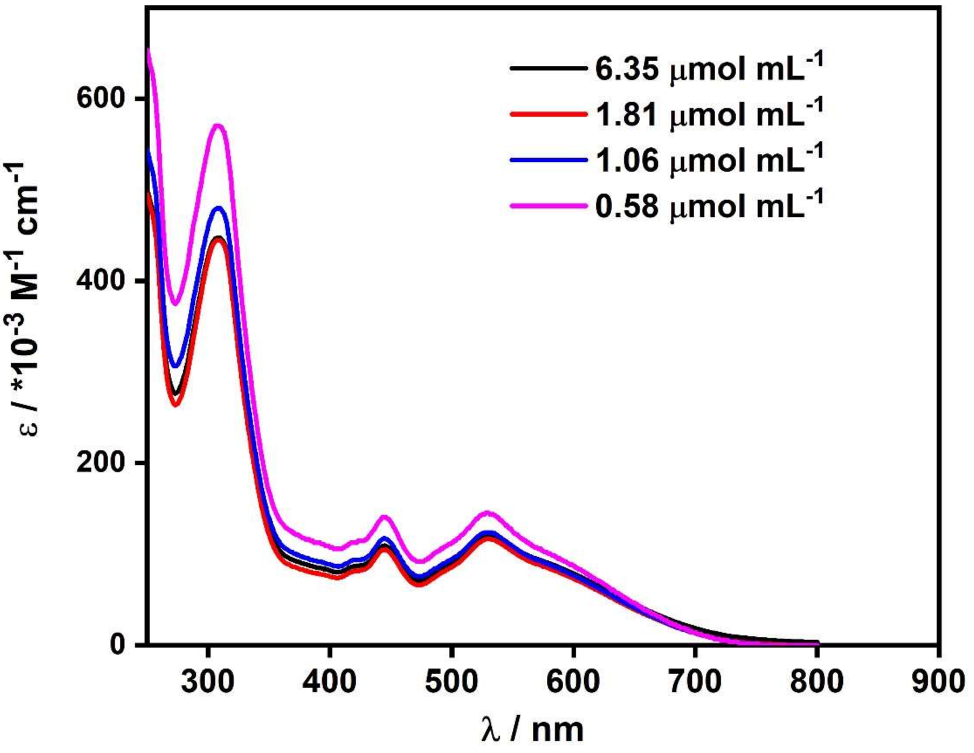

Figure S160. Absorption spectra of Compound **14** in DCM solution with different concentrations**.**

Table **S9**. Emission lifetimes of **14** in DCM solution and in the solid-state.

**Samples**	0.15 μmol mL^{-1} in DCM[a]	33 μmol mL^{-1} in DCM[b]	0.1wt% in PMMA[b]	1wt% in PMMA[b]	10 wt% in PMMA[b]	Neat film[b]
**τ_p(ns)**	4.0(34%), 12.1(66%)	0.9(20%), 3.7(64%), 12.0(14%)	1.7(65%), 5.1%(35%)	1.9(67%), 5.0%(33%)	0.6(84%), 3.4(16%)	<1

[a]The detected wavelength is 480 nm; [b]The detected wavelength is 730 nm; all the samples were measured with an excitation wavelength of 370 nm under an oxygen-free atmosphere at room temperature.
